# Nickel-catalyzed difunctionalization of allyl moieties using organoboronic acids and halides with divergent regioselectivities†
†Electronic supplementary information (ESI) available. CCDC 1563066. For ESI and crystallographic data in CIF or other electronic format see DOI: 10.1039/c7sc03149a


**DOI:** 10.1039/c7sc03149a

**Published:** 2017-10-26

**Authors:** Wanfang Li, Jie Kang Boon, Yu Zhao

**Affiliations:** a Department of Chemistry , National University of Singapore , 3 Science Drive 3 , 117543 , Republic of Singapore . Email: zhaoyu@nus.edu.sg

## Abstract

Efficient difunctionalization of alkenes allows the rapid construction of molecular complexity from simple building blocks in organic synthesis. We present herein a nickel-catalyzed dicarbofunctionalization of alkenes using readily available organoboronic acids and organic halides in a three-component fashion. In particular, an unprecedented regioselectivity of the 1,3-dicarbofunctionalization of *N*-allylpyrimidin-2-amine is achieved when aryl and methyl iodides are utilized. In contrast, the use of alkyl bromides with β-hydrogens results in 1,3-hydroarylation or oxidative 1,3-diarylation. Preliminary mechanistic studies suggest an isomerization involving nickel hydride in the 1,3-difunctionalization reactions. On the other hand, the use of alkenyl or alkynyl halides promotes alternative regioselectivities to deliver 1,2-alkenylcarbonation or intriguing 2,1-alkynylcarbonation products. Such 2,1-alkynylarylation is also applicable to *N*-allylbenzamide as a different class of substrates. Overall, this nickel-catalyzed process proves to be powerful in delivering versatile difunctionalized compounds using readily available reagents/catalysts and a simple procedure.

## Abstract 2

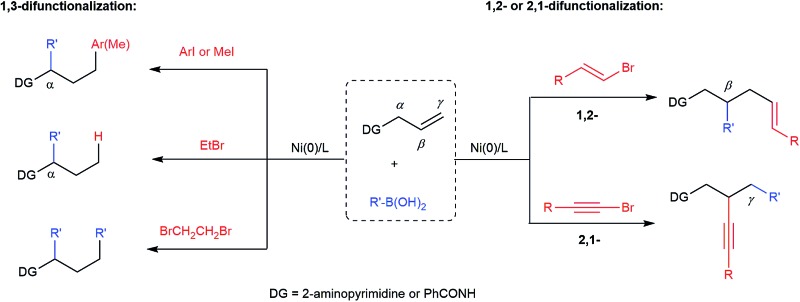
We present herein a nickel-catalyzed dicarbofunctionalization of alkenes using readily available organoboronic acids and organic halides in a three-component fashion.

## 1. Introduction

**Scheme 1:**
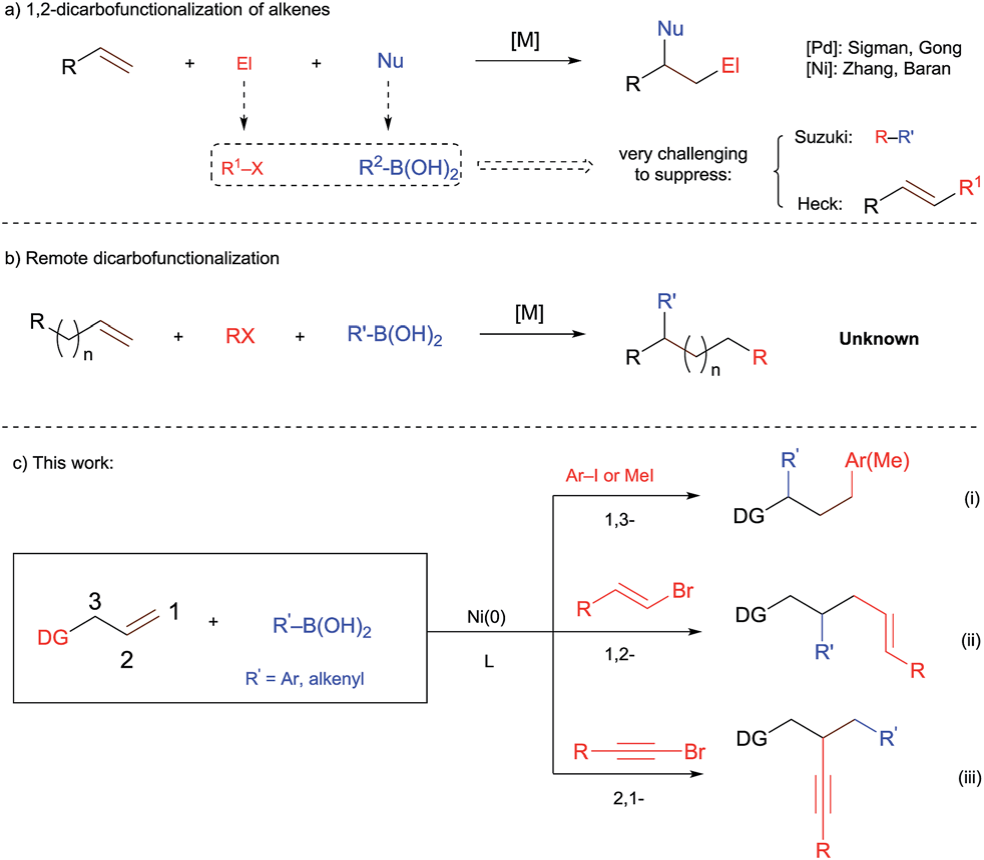
TM-catalyzed difunctionalization of alkenes.

The identification of new strategies for building up molecular complexity from simple chemical feedstocks represents an important goal in organic synthesis and application to medicinal and materials chemistry. To this end, the transition-metal-catalyzed dicarbofunctionalization of alkenes^1^–^3^ and alkynes^4^ by the introduction of one carbon electrophile and one carbon nucleophile has attracted much attention. In particular, the three component reaction using readily available and easy-to-handle organic boronic acids and organic halides for these transformations provides great practical advantages. A few elegant examples of alkyne difunctionalization using these reagents have been documented.^5^ For the corresponding difunctionalization of alkenes, on the other hand, suppressing the classical Heck and Suzuki couplings poses a formidable challenge (Scheme 1a). The few successful examples along these lines either require the use of strained alkene substrates1a,b or are limited to special alkyl halide electrophiles.3*b* The development of a dicarbofunctionalization of alkenes that tolerates a variety of organic boronic acids and halides will be highly desired.

In a related area of research, remote alkene functionalization, which selectively installs a functionality distal to the initial alkene moiety *via* metal catalyzed C=C bond isomerization (“chain walking”), has also attracted much attention.^6^ Such a process greatly expands the chemical space accessible from readily available alkenes. However, a remote dicarbofunctionalization of alkenes using boronic acids has never been documented before (Scheme 1b). We report herein the first example of dicarbofunctionalization of terminal alkenes substituted with a 2-aminopyrimidine unit using readily available organoboronic acids and halides. This nickel-catalyzed process turns out to be versatile. Depending on the choice of organic halides, divergent regioselectivities are achieved, including the unprecedented 1,3-diarylation and 2,1-alkynylcarbonation (Scheme 1c).

Our group has been previously interested in the carbofunctionalization of alkenes under base metal catalysis.^7^ We became particularly interested in the development of a general alkene dicarbofunctionalization reaction using organic boronic acids and halides. Compared to palladium catalysis, we envisioned that the use of a nickel catalyst might be better suited for these challenges considering that nickel has distinct reactivities in the elementary steps of cross-coupling.^8^ Indeed, nickel catalysis has shown great promise for the related functionalization of alkenes^9^ and alkynes,^4^ and the use of a nickel catalyst is also attractive for the promotion of economical and sustainable chemical synthesis.

## 2. Results and discussion

### 2.1 Initial discovery and optimization

We initiated our investigation with the dicarbofunctionalization of simple alkenes such as allylbenzene (**1a**) and *N*-allyl aniline (**1b**) using iodobenzene and phenylboronic acid. Under various nickel-catalyzed conditions, unfortunately, no diarylation product was detected at all (entries 1 and 2, Table 1). This prompted us to turn our attention to alkenes with a coordinative directing group. While the use of amides (**1c** and **1d**) or an ester (**1e**) as the directing group proved futile (entries 3–5), we were excited to observe the formation of a desired diarylation product in 23% isolated yield from the use of **1f** (entry 6), together with 42% yield of a Suzuki coupling side product. A screening of common phosphine ligands was carried out, from which dppm provided the optimal 47% isolated yield (entry 9 *vs.* 7 and 8). Here again, Suzuki cross-coupling consumed the reagents to a large extent (40% yield of biphenyl). Intriguingly, the product was determined to be a mixture of **2f** and an unexpected 1,3-difunctionalization product **2f′**, which represents an unprecedented regioselectivity in alkene difunctionalization. Gratifyingly, when **1g** bearing 2-aminopyrimidine was examined next, the 1,3-diarylation product **2g′** was isolated exclusively with an improved yield of 68% (entry 10). Moreover, the undesired Heck and Suzuki products were minimized to 5% and 15% respectively. Other substrates, bearing related directing groups (**1h–j**), were also examined and suffered from either low conversion or deallylation of the substrate (entries 11–13). Different metal precursors were also examined under the optimized conditions. Ni(ii) species in the presence of a reductant proved to be much less effective (entries 14 and 15). Importantly, the use of a palladium catalyst resulted in the deallylation of the substrate and undesired Suzuki coupling without any difunctionalization (entry 16).

**Table 1:** Optimization of Ni-catalyzed diarylation^*a*^

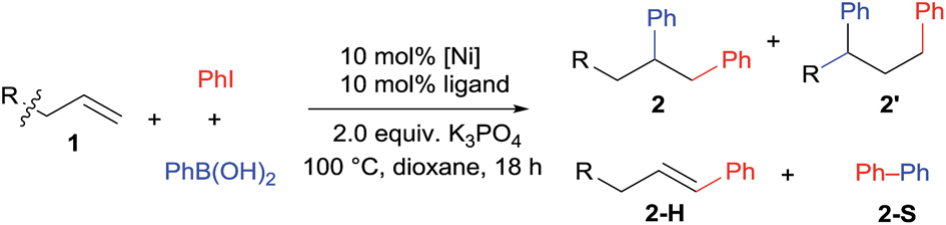							
Entry	**1**	[M]	Ligand	Yield (%)			r.r.
				**2H**	**2S**	**2′** ^*a*^	**2**/**2′**^*b*^
1	**1a**	Ni(COD)_2_	PPh_3_	<2	<2	n.r.	—
2	**1b**	Ni(COD)_2_	PPh_3_	<5	<5	Trace	—
3	**1c**	Ni(COD)_2_	PPh_3_	<2	<2	n.r.	—
4	**1d**	Ni(COD)_2_	PPh_3_	<2	<2	n.r.	—
5	**1e**	Ni(COD)_2_	PPh_3_	<2	<2	n.r.	—
6	**1f**	Ni(COD)_2_	PPh_3_	13	42	23^*c*^	n.d.
7	**1f**	Ni(COD)_2_	PEt_3_	5	12	10	n.d.
8	**1f**	Ni(COD)_2_	dppf	5	27	28	n.d.
9	**1f**	Ni(COD)_2_	dppm	5	40	47^*c*^	3/2
10	**1g**	Ni(COD)_2_	dppm	<5	15	68^*c*^	1/>20
11	**1h**	Ni(COD)_2_	dppm	<5	<5	<1^*d*^	—
12	**1i**	Ni(COD)_2_	dppm	<2	<2	<1^*d*^	—
13	**1j**	Ni(COD)_2_	dppm	<2	<2	n.r.	—
14^*e*^	**1g**	Ni(acac)_2_	dppm	<5	17	29	1/6
15^*e*^	**1g**	NiCl_2_·glyme	dppm	<5	41	16	n.d.
16	**1g**	Pd_2_(dba)_3_	dppm	<5	25	0^*f*^	—
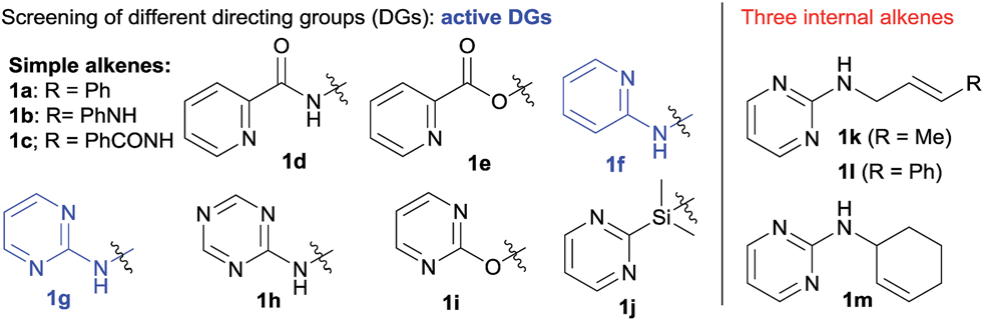							

^*a*^ **1** (0.2 mmol), PhI (34 μL, 0.3 mmol), PhB(OH)_2_ (36.5 mg, 0.3 mmol). GC-yield determined using *n*-hexadecane as the internal standard. The yield of biphenyl was based on PhI.

^*b*^Determined by ^1^H NMR.

^*c*^Isolated yield.

^*d*^Deallylation was the major side reaction.

^*e*^AlMe_3_ (20 mol%) was added for the reduction of Ni(ii) to Ni(0).

^*f*^The Suzuki product (biphenyl) and the deallylation product (2-aminopyrimidine) were the major side products. n.r. = no reaction. n.d. = not determined.

### 2.2 Scope of 1,3-dicarbofunctionalization

**Scheme 2:**
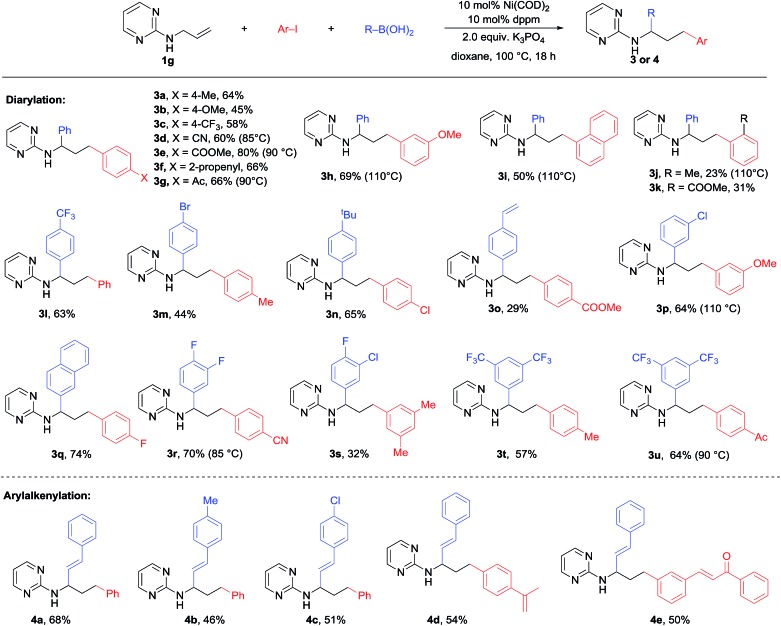
Scope of 1,3-diarylation/arylalkenylation.

With the optimal conditions in hand, we moved on to examining the substrate scope of this 1,3-difunctionalization reaction. Initial efforts were focused on the variation in the substitution pattern of the alkene substrate. Unfortunately, various internal alkenes including **1k**, **1l** and **1m** (Table 1) all failed to participate in this three-component difunctionalization reaction. Even under more forcing conditions (15% catalyst loading, 110 °C, 36 h), only Heck and Suzuki products were obtained in moderate yield. This was a serious limitation and prompted us to focus on the reaction with terminal alkenes. On the other hand, as shown in Scheme 2, various aryl iodides bearing *para*-, *meta*- and *ortho*-substituents of electron-withdrawing or electron-donating character successfully participated in this reaction to produce **3a–k** in moderate to high yields. This method showed a high tolerance to functional groups including cyano (**3e**), alkene (**3g**), ester (**3f**) and ketone (**3h**) groups. A higher temperature was required for the *ortho*-substituted aryl iodides probably due to steric hindrance (**3i** and **3j**). In the case of 4-iodo-benzonitrile, a lower temperature of 85 °C proved to be important to produce **3d** in a good 65% yield, while higher temperature led to much decomposition. Unfortunately, heteroaryl iodides like 2-iodo thiophene, pyridine, and pyrimidine gave only trace amounts of the desired product. A wide range of aryl boronic acids bearing bromo, vinyl, fluoro and trifluoromethyl substituents could also be used to deliver a range of diarylation products **3l–u** with good efficiency. Notably, alkenyl boronic acids could also be employed under the same conditions to afford the arylalkenylation products **4a–e** in good to high yields, which dramatically expanded the scope of this 1,3-dicarbofunctionalization of alkenes. However, heteroaryl boronic acids (2-thienyl, 2-furyl and 5-quinolinyl boronic acids) led to only trace amounts of the products owing to the decomposition of these boronic acids under the diarylation conditions.

### 2.3 Mechanistic studies

**Scheme 3:**
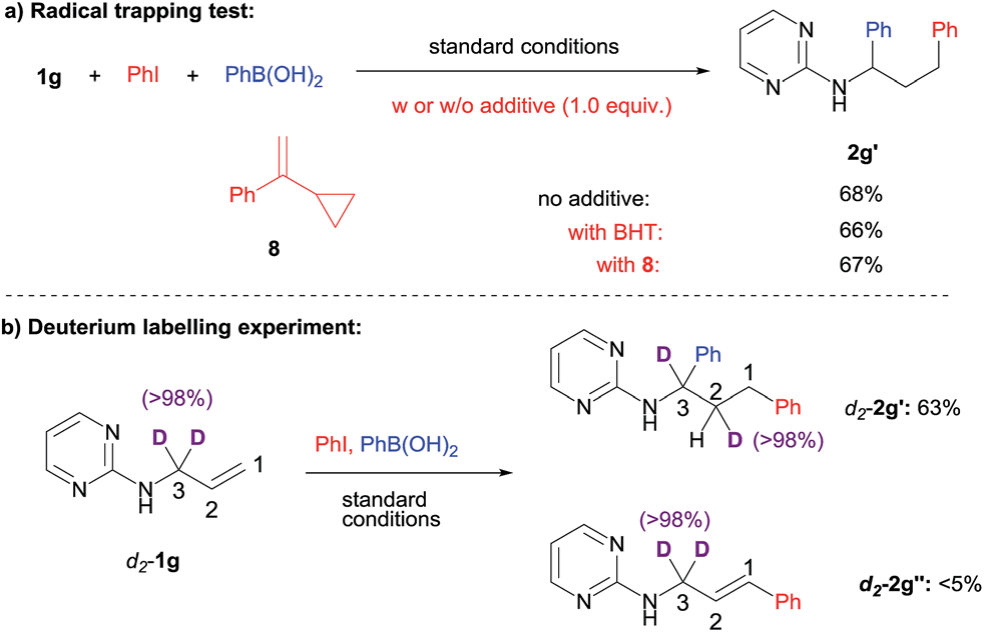
Mechanistic investigations.

To shed some light on the reaction mechanism, we first examined the effect of radical trapping reagents such as BHT (2,6-di-*tert*-butyl-*p*-cresol) or α-cyclopropylstyrene **8** on the difunctionalization reaction (Scheme 3a). As it turned out, the efficiency of the diarylation remained constant in the presence of these additives. In addition, the additives also remained unchanged after the reaction. This suggested that the diarylation likely did not involve a radical process. Although at this point we can not rule out the possibility of radical formation followed by a very fast trapping by Ni within the solvent cage.

We were curious whether a chain-walking mechanism was operating in this 1,3-difunctionalization process. To probe that possibility, deuterium labelling of the substrate was examined (Scheme 3b). When 3,3′-deuterium-labeled **1g** was subjected to the standard conditions, a clean transfer of one deuterium to the 2-position was observed, which is consistent with the chain-walking mechanism. For the small amount of Heck side product **2g′′**, on the other hand, the deuterium labelling remained at the 3-position, suggesting that the transfer proceeded after the proposed carbometallation step.

**Scheme 4:**
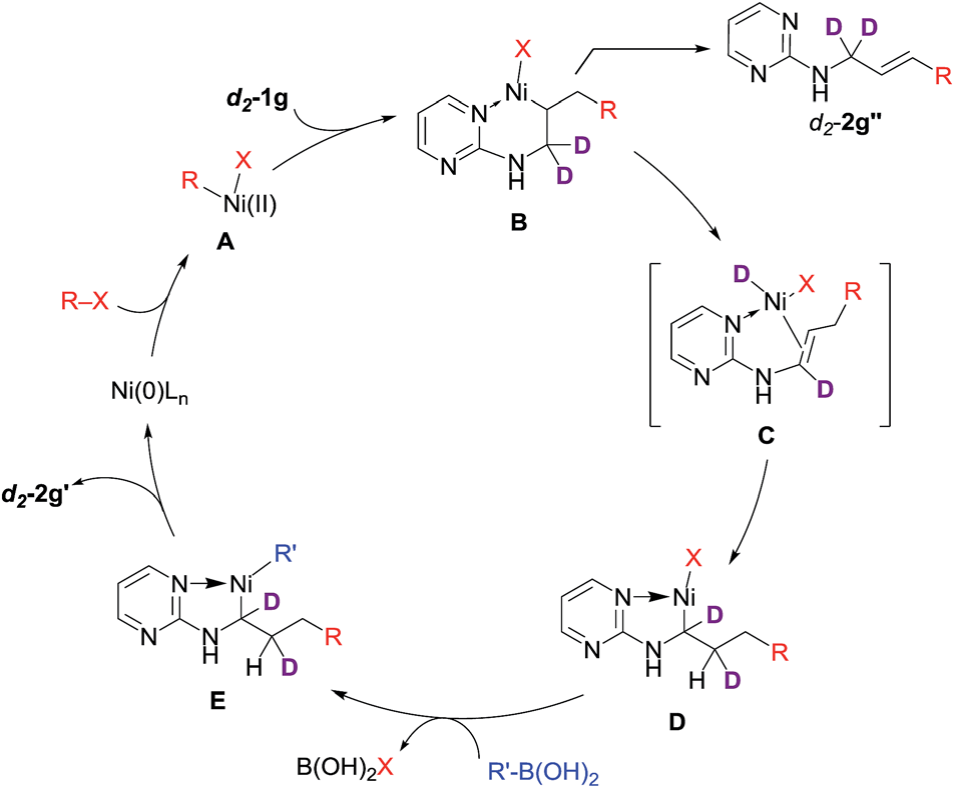
Plausible mechanism for the 1,3-dicarbofunctionalization.

Based on these data, we proposed a domino Heck–isomerization–Suzuki reaction pathway for the 1,3-diarylation as shown in Scheme 4. Oxidative addition of Ni(0) to aryl iodide followed by carbonickelation of the substrate produces intermediate **B**. Chelation from the substrate presumably reduced the rate of the β-hydride elimination to deliver the Heck side product. On the other hand, **B** could undergo transmetalation directly to yield a 1,2-difunctionalization product. Instead of that, **B** is isomerized to **D** through β-hydride elimination and reinsertion to form the five-membered chelation complex. Transmetalation of **D** with organoboronic acid then yields **E**, which undergoes reductive elimination to produce the diarylation product and regenerate the Ni(0) catalyst.

### 2.4 1,2-Alkenylcarbonation

**Scheme 5:**
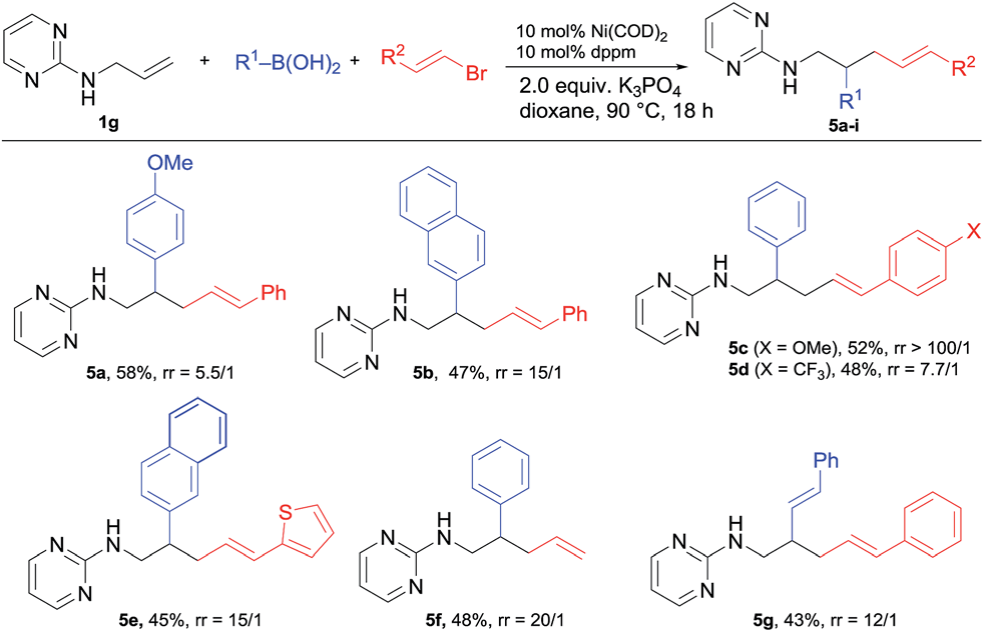
Scope of 1,2-alkenylcarbonation.

When alkenyl bromides were examined under the same conditions, the related alkenylarylation proceeded smoothly, but with an interesting switch to the 1,2-regioselectivity (Scheme 5). Under the standard conditions, a range of alkenyl bromides bearing various substituted arenes including heterocycles such as thiophene as well as unsubstituted vinyl bromide were all applicable for this 1,2-alkenylation (**5a–f**). Besides, dialkenylation could also be realized to deliver **5g**.

**Scheme 6:**
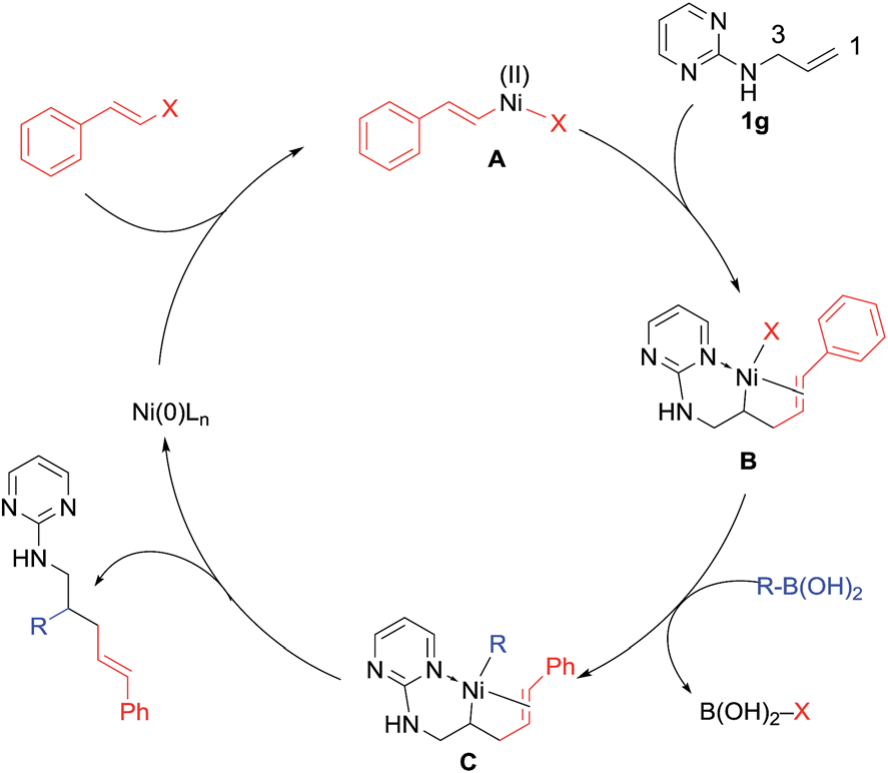
Proposed mechanism of 1,2-alkenyl carbonalization.

It is worth noting that while the ratio of 1,2 *vs.* 1,3-difunctionalization (r.r.) for most **5** products was >10/1 (as determined by GC-MS), the electronic property of the styryl halides had a significant influence on the regioselectivity. As shown by the comparison of **5c** and **5d**, the change of the electron-withdrawing CF_3_ to the electron-donating OMe increased the ratio from 7.7/1 to >100/1. We assume that this 1,2-regioselectivity for alkenylarylation could be attributed to the coordinating effect of the alkenyl unit on nickel (Scheme 6). Once intermediate **B** is formed from the alkenyl nickelation of **1g** with **A**, the C=C bond from the electrophile would coordinate to nickel and suppress the isomerization. When an electron-deficient alkenyl halide is used, it presumably does not coordinate to nickel so effectively, leading to a higher ratio of isomerization and in turn 1,3-difunctionalization.^10^

### 2.5 Alkynyl carbonation

**Scheme 7:**
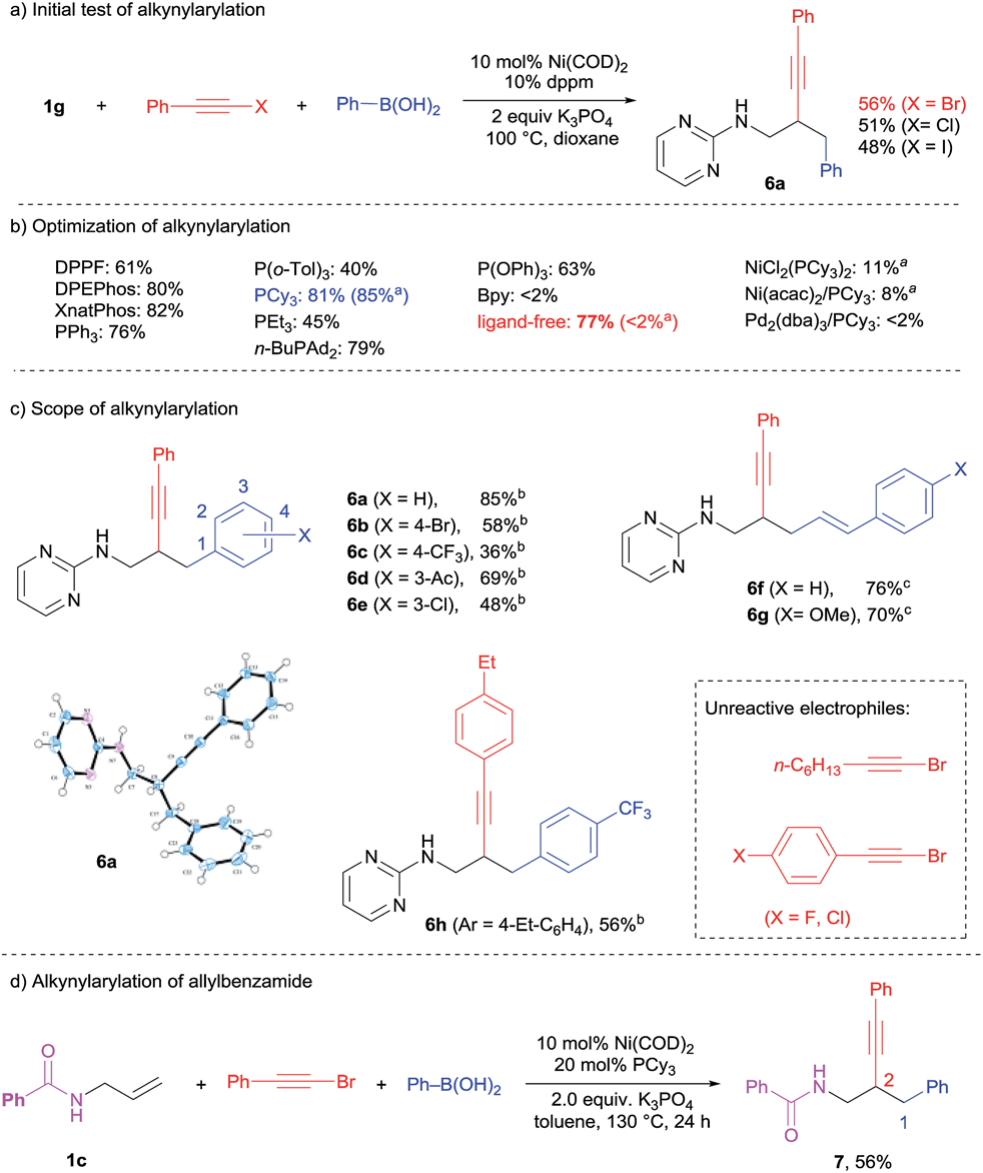
2,1-Alkynylcarbonation. ^a^Reaction in toluene. ^b^Reactions in toluene with PCy_3_. ^c^Reactions in dioxane with dppm.

Alkynyl halides are versatile and powerful reagents for the introduction of alkynyl groups into organic molecules.^11^ Based on the success in the dicarbofunctionalization of **1g** with various aryl and alkenyl halides, a three-component reaction between **1g**, (bromoethynyl)benzene and PhB(OH)_2_ was tested under the previously optimized conditions (Scheme 7a). A moderate isolated yield of 56% was obtained for the difunctionalization product **6a** using Ni(COD)_2_/dppm. The use of alkynyl chloride and iodide also yielded **6a**, albeit in lower yields. To our great surprise, both NMR and X-ray structure analysis confirmed an unexpected reverse regioselectivity. In contrast to the 1,2-alkenylarylation in Scheme 5, the alkynyl group was added on to the internal position of the C=C bond while the phenyl groups were added on to the terminals. To further optimize this intriguing transformation, various bisphosphine, monophosphine, phosphite and bipyridine ligands were screened (Scheme 7b). The yield of **6a** could be improved to 85% by using PCy_3_ as the ligand and toluene as the solvent. It is noteworthy that a good yield of 77% could be obtained for **6a** even in the absence of a ligand in dioxane (<2% in toluene). The coordinative solvent presumably played an important role in stabilizing the nickel species in this case. On the other hand, the combination of Ni(ii) precursors with PCy_3_ or the use of a Pd complex were much less effective for this 2,1-alkynylarylation (<11% for **6a**).

The scope of this 2,1-alkynylation was extended to various alkynyl bromides and boronic acids under optimized conditions (Scheme 7c). Bromo (**6b**), trifluoromethyl (**6c**), acetyl (**6d**) and chloro (**6e**) substituents on the phenylboronic acids could be well-tolerated. Alkenylboronic acids also worked well to yield **6f** and **6g** in good yield.

This catalytic system, however, showed a narrow scope for the alkynyl reagents. Other than electron-neutral aryl-substituted alkynyl bromides (59% yield for **6h**), the perturbation of the electronic property of aryl-alkynyl bromides and the use of alkyl-substituted alkynyl bromide all resulted in no conversion to the desired product. While the previous 1,3- and 1,2-dicarbofunctionalizations necessitate the use of pyrimidine directing groups, we were curious whether the 2,1-alkynylarylation could overcome this limitation. To our excitement, simple *N*-allylbenzamide **1c** was identified to be a suitable substrate for this reaction. As shown in Scheme 7d, the 2,1-difunctionalized product **7** was obtained in a good yield of 56% at a slightly higher temperature. Further exploration of the scope of this transformation is ongoing.

**Scheme 8:**
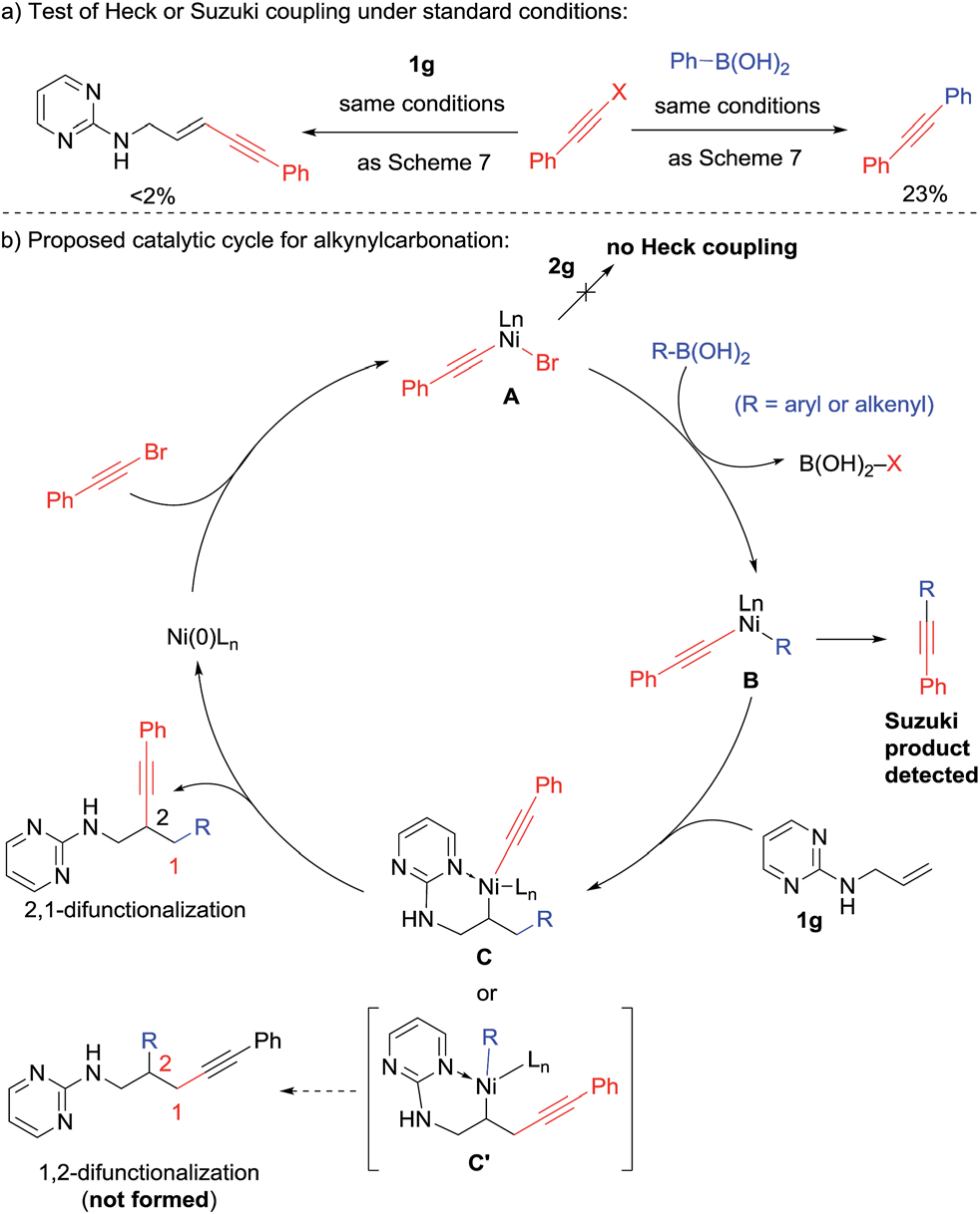
Proposed mechanism for alkynylarylation.

It is noteworthy that in the previous 1,3-diarylation and 1,2-alkenylcarbonation reactions, a small amount of Heck product was isolated as the side product. In contrast, no Heck product was observed in the 2,1-alkynylcarbonation. Instead, a small amount of Suzuki product was detected. This observation was further confirmed in the attempted two-component couplings under standard conditions (Scheme 8a); the alkynyl bromide underwent Suzuki coupling but not the Heck reaction. Taking this observation and the intriguing reverse regioselectivity into consideration, we propose the reaction mechanism shown in Scheme 8b. Instead of reacting with the alkene, the oxidative addition intermediate **A** preferentially underwent transmetalation with R–B(OH)_2_ to form **B**, which featured two distinct carbanion ligands on nickel. Selective insertion of **1g** into the aryl/alkenyl–nickel bond led to the formation of intermediate **C**, which delivered the 2,1-alkynylarylation products through reductive elimination. The key chemo-selectivity in the carbonickelation step was likely due to the fact that nickel–alkynyl bonds are much stronger than the Ni–aryl and Ni–alkenyl counterparts.^12^

### 2.6 Reaction with alkyl halides

**Scheme 9:**
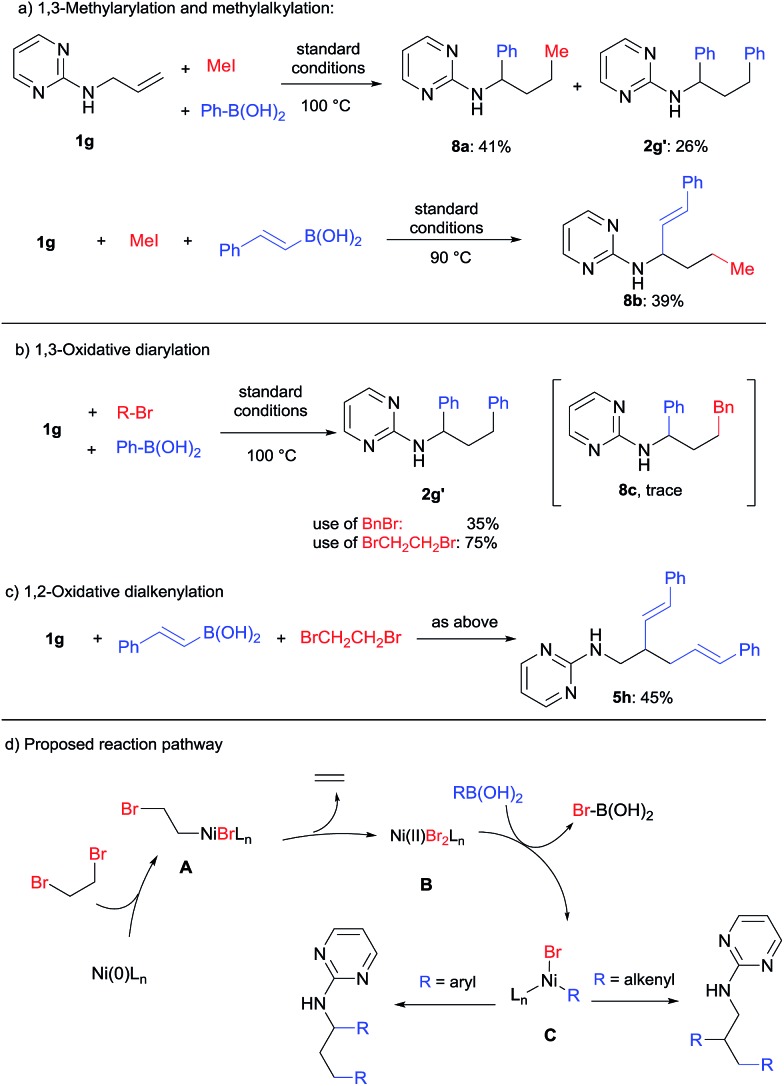
Difunctionalization using alkyl halides.

Alkyl halides are usually poor electrophiles in transition-metal catalyzed cross-coupling reactions due to their slow oxidative addition and facile β-hydride elimination.^13^ As shown in Scheme 9a, when methyl iodide was used for dicarbofunctionalization of **1g**, the 1,3-methylarylation (**8a**) or 1,3-methyl alkylation (**8b**) products could be obtained in 41% and 39% yield by using phenyl or styryl boronic acid, respectively. In addition, an unexpected oxidative diarylation product (**2g′**) was also obtained.

When methyl iodide was replaced by benzyl bromide, interestingly, only a trace amount of the desired product (**8c**) was detected using GC-MS, while **2g′** was obtained as the major product in 35% yield (Scheme 9b). By the use of a known oxidant, 1,2-dibromoethane, the yield of **2g′** could be improved to 75%. This transformation thus represents a novel oxidative 1,3-diarylation of this class of terminal alkenes.

An oxidative dialkenylation was also attempted under the same conditions (Scheme 9c). It is intriguing to note that 1,2-dialkenylation product **5h** was formed selectively instead of 1,3-difunctionalization. This also provided an important piece of evidence for the reaction mechanism (Scheme 9d). Starting from the Ni(0) catalyst, oxidative addition/β-bromide elimination could produce Ni(ii) dibromide (**B**) by the release of ethylene gas.^14^ Transmetalation of **B** with organoboronic acid then generated the key organo-nickel intermediate **C**. The next steps will then follow the pathways for either 1,3-diarylation (Scheme 4) or 1,2-dialkenylation (Scheme 6) as discussed in previous sections.

**Scheme 10:**
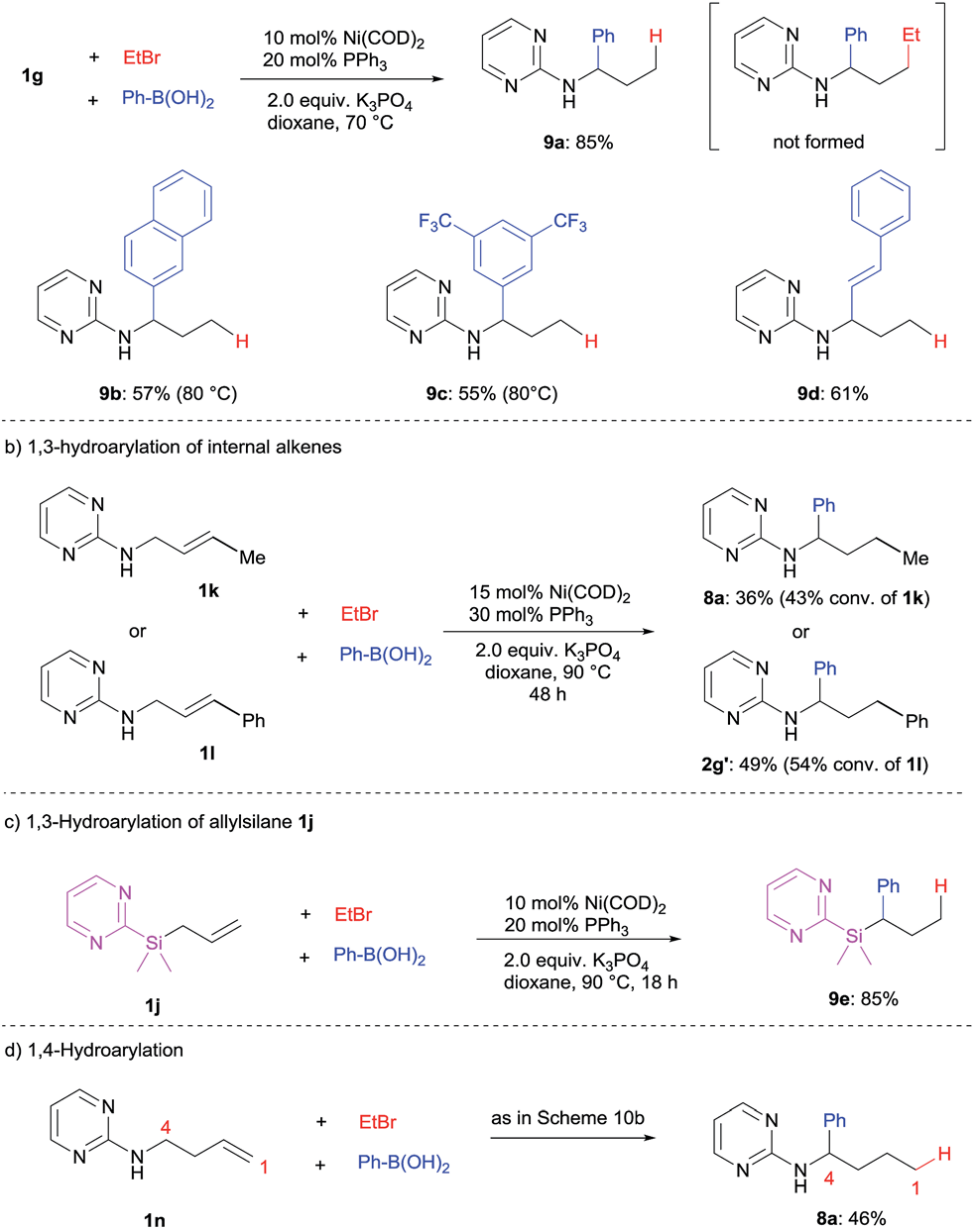
1,3- and 1,4-hydrocarbonation using alkyl halides bearing β-hydrogen.

When alkyl halides bearing β-hydrogen such as ethyl bromide were employed (see ESI† for other alkyl bromides), a complete switch of reactivity was observed. 1,3-Hydroarylation of the terminal alkene **1g** proceeded smoothly to yield **9a** in a high yield of 85% (Scheme 10a). In this transformation, the nickel hydride intermediate is presumably generated from nickel(0) precursor/alkyl bromide and undergoes the following hydrofunctionalization.4*j* This 1,3-hydroarylation or hydroalkenylation proved to be general to deliver **9b–d** in good yields. In this case, previously unreactive internal alkenes **1k** and **1l** were able to participate in the 1,3-hydroarylation, albeit with moderate reactivity (Scheme 10b).

Importantly, other substrates such as allylsilane **1j** could also undergo 1,3-hydroarylation under the optimized conditions to produce silane **9e** in high yield (Scheme 10c). This greatly expands the scope of this catalytic transformation.

To push the limit of this remote functionalization, a 1,4-hydroarylation of *N*-homoallyl pyrimidineamine (**1n**) was attempted under similar conditions. To our excitement, the desired product **8a** was successfully obtained, albeit in a moderate yield (Scheme 10d). Unfortunately, the diarylation of **1n** and other internal alkene substrates was unsuccessful due to their low reactivity (see ESI for details†).

### 2.7 Application of the methodology

**Scheme 11:**
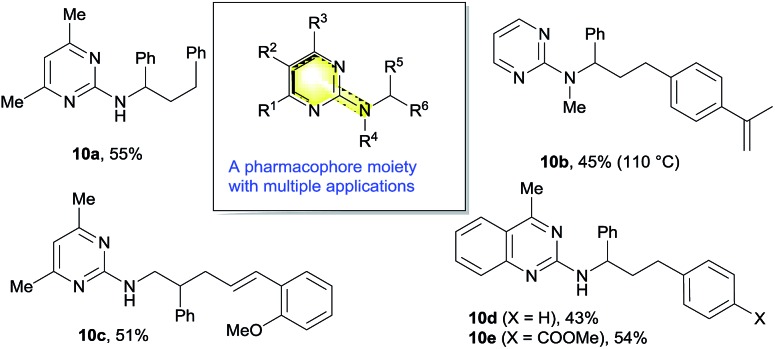
Synthesis of various aminopyrimidine pharmacophores.

The 2-aminopyrimidine motif represents a universal pharmacophore in bioorganic and medicinal chemistry (Scheme 11), and has found many applications including hypertension and congestive heart failure treatment as well as in inhibitors of protein kinases.^15^ This nickel-catalyzed difunctionalization reaction could tolerate various substitution patterns on the nitrogen or pyrimidine ring to deliver a range of heterocycles that can be applicable for biological screening (**10a–e**). On the other hand, the removal of the pyrimidine moiety was also attempted under various conditions, which only led to the cleavage of the aminopyrimidine completely to deliver hydrocarbon products (see ESI for details†).

## 3. Conclusions

In summary, we have realized the first nickel-catalyzed dicarbofunctionalization of *N*-allyl 2-aminopyrimidine using readily available organic boronic acids and halides. Divergent regioselectivities were obtained based on the nature of different hybridized organic halides. Aryl and methyl iodides led to an intriguing 1,3-regioselectivity for the reaction through a domino Heck–isomerization–Suzuki pathway. For alkenyl halides, the C=C bond coordination helped to anchor the Ni(ii) intermediate, resulting in less of a tendency towards isomerization and the formation of 1,2-difunctionalization instead. The reaction with alkynyl halides produced a reversed 2,1-regioselectivity, presumably resulting from an alternative sequence of transmetalation followed by selective C=C insertion into the weaker Ni(ii)-carbon bond. Alkyl halides with β-hydrogen furnished the 1,3- or 1,4-hydrofunctionalization product. In addition, dihaloethane worked as an oxidant under the same conditions to form the oxidative dicarbofunctionalization products. These methodologies can be used for the synthesis of 2-aminopyrimidine motifs as attractive pharmacophores. Current efforts in our laboratory are directed towards the dicarbofunctionalization of more diverse alkenes and enantioselective variants of these transformations.

## Conflicts of interest

There are no conflicts to declare.

## Supplementary Material

Supplementary information

Crystal structure data
