# Supplementary material for: Nickel-catalyzed difunctionalization of allyl moieties using organoboronic acids and halides with divergent regioselectivities
Source: Chem Sci. 2017 Oct 26;9(3):600–7. doi: 10.1039/c7sc03149a (PMC5869318; doi:10.1039/c7sc03149a)
Supplement: Supplementary file 1 [file SC-009-C7SC03149A-s001.pdf]

Electronic Supplementary Material (ESI) for Chemical Science.
This journal is © The Royal Society of Chemistry 2017

Electronic Supplementary Information

Nickel-catalyzed Difunctionalization of Allyl Moieties Using Organoboronic Acids and Halides with Divergent Regioselectivities

Wanfang Li, Jie Kang Boon, and Yu Zhao*
zhaoyu@nus.edu.sg
Department of Chemistry, National University of Singapore
3 Science Drive 3, Singapore 117543

Table of Contents

1. General Information **S3**

2. Preparation of the starting materials. **S3**

3. General procedures and reaction optimization. **S7**

3.1 General procedure for the 1,3-dicarbofunctionalization. *S8*

3.2 General procedure for the alkynylcarbonation. *S8*

3.3 General procedure for the hydrocarbonation. *S8*

4. Characterization data of the difunctionalization products. **S10**

*4.1 Diarylation and arylalkenylation products **2-4**.* *S10*

*4.2 Alkenylcarbonation products **5**.* *S16*

*4.3 Alkynylcarbonation product **6** and **7**.* *S18*

*4.4 Functionalization products with alkyl halides **8** and **9*** *S21*

*4.5 Application of the method for pharmacophores **10**.* *S22*

5. Mechanistic studies. **S24**

5.1 Radical trapping experiment. *S24*

5.2 Deuterium-labelling experiments. *S24*

6. Initial attempts on removing of the directing group **S26**

7. References **27**

8. NMR spectra of new compounds **27**

1. General Information

Chemicals and solvents were purchased from commercial suppliers and used as received. **^{1}H, ^{13}C** and **^{19}F NMR** spectra were recorded on a Bruker ACF300 (300 MHz) or AMX (500 MHz or 400 MHz) spectrometer. Chemical shifts were reported in parts per million (ppm), and the residual solvent peak was used as an internal reference: proton (chloroform δ 7.26), carbon (chloroform δ 77.0) or tetramethylsilane (TMS δ 0.00) was used as a reference. Multiplicity was indicated as follows: s (singlet), d (doublet), t (triplet), q (quartet), p (pentet), m (multiplet), dd (doublet of doublet), bs (broad singlet). Coupling constants were reported in Hertz (Hz). Low resolution mass spectra were obtained on a Finnigan/MAT LCQ spectrometer in ESI mode, and a Finnigan/MAT 95XL-T mass spectrometer in EI mode. All high resolution mass spectra (**HRMS**) were obtained on a Finnigan/MAT 95XL-T spectrometer. **GC-MS** were obtained on a Shimdazu QP-2000 spectrometer in EI mode.

Materials and Methods. Unless otherwise stated, starting materials were purchased from Aldrich and/or Fluka. Solvents were purchased in ACS reagent grade quality. For thin layer chromatography (TLC), Merck pre-coated TLC plates (Merck 60 F254) were used, and compounds were visualized with a UV light at 254 nm. Further visualization was achieved by staining with iodine. Flash chromatography separations were performed on 300 – 400 mesh silica gel.

2. Preparation of the starting materials.

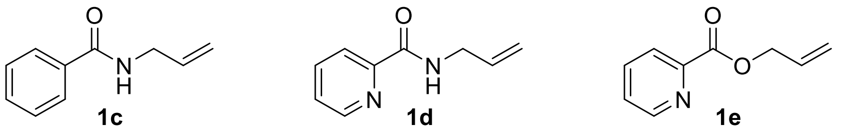

1c[1] and **1d**[2] are known compounds and were prepared according to the reported procedures.

1e is a known compound[3] and was prepared as the following procedure: Picolinic acid (2.5 g, 20 mmol) and Cs_2CO_3 (10.0 g, 32.0 mmol) were dissolved in anhydrous DMF (30 mL), to which allyl bromide (2.1 mL, 24 mmol) was added dropwise. The mixture was allowed to stir at room temperature overnight. Then 30 mL of water was added and the aqueous layer was extracted with diethyl ether (30 mL × 3). The combined organic phase was dried over anhydrous Na_2SO_4 and the desired product was obtained as pale yellow oil (1.3 g, 40%) by flash column chromatography (Hexane/EA = 6). **^{1}H NMR** (500 MHz, $CDCl_3$) δ 8.75 (d, *J* = 3.3 Hz, 1H), 8.12 (d, *J* = 7.9 Hz, 1H), 7.83 (td, *J* = 7.8, 1.7 Hz, 1H), 7.46 (ddd, *J* = 7.7, 4.8, 1.2 Hz, 1H), 6.12 – 5.98 (m, 1H), 5.41 (dd, *J* = 17.1, 1.5 Hz, 1H), 5.29 (dd, *J* = 10.4, 1.2 Hz, 1H), 4.89 (dt, *J* = 5.9, 1.3 Hz, 2H).

(1f) *N*-Allylpyridin-2-amine

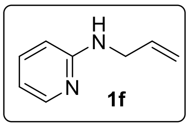

1f is a known compound[4] and was prepared by the reported procedure: Under the protection of N_2, *t*BuOK (3.37 g, 30.0 mmol) was added in one portion into a solution of 2-aminopyridine (1.88 g, 20.0 mmol) in anhydrous THF (60 mL). After stirring at room temperature for 1 h, allyl bromide (2.3 mL, 26.0 mmol) in THF (10 mL) was added dropwise to the resultant dark greenish solution. After stirring for another 2 hours, the reaction was quenched with 20 mL of saturated NH_4Cl aqueous solution. THF was then removed under reduced pressure. The residue was extracted with EtOAc (20 mL × 3) and the combined organic phase was washed with brine and dried over anhydrous Na_2SO_4. The desired product (1.46 g, 55%) was obtained as light yellow liquid by flash column chromatography (Hexane/EA = 5). **^{1}H NMR** (300 MHz, $CDCl_3$) δ 8.06 (d, *J* = 5.8 Hz, 1H), 7.44 – 7.30 (m, 1H), 6.53 (q, *J* = 7.1, 6.7 Hz, 1H), 6.36 (d, *J* = 8.0 Hz, 1H), 6.03 – 5.81 (m, 1H), 5.24 (d, *J* = 17.3 Hz, 1H), 5.12 (d,

J = 9.5 Hz, 1H), 4.87 (s, 1H), 3.90 (t, J = 5.9 Hz, 2H). **^{13}C NMR** (75 MHz, $CDCl_3$) δ 158.6, 148.0, 137.3, 135.0, 115.8, 112.9, 106.6, 44.5.

(1g) *N*-Allylpyrimidin-2-amine

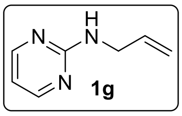

1g is a known compound and was prepared by reported procedure:[5] A solution of 2-chloropyrimidine (4.58 g, 40.0 mmol) in allylamine (10.0 mL, 134 mmol) was heated at 70 °C for 3 hours in a seal tube. After cooling to room temperature, the mixture was washed with 10% aq. NaOH (20 mL) and extracted with ether (30 mL × 3). The combined organic phase was washed with brine and dried over anhydrous Na_2SO_4. The desired product (4.8 g, 88%) was obtained as light yellow oil by flash column chromatography (Hexane/EA = 5). **^{1}H NMR** (300 MHz, $CDCl_3$) δ 8.26 (d, J = 4.8 Hz, 2H), 6.50 (t, J = 4.8 Hz, 1H), 6.17 (s, 1H), 6.06 – 5.81 (m, 1H), 5.37 – 5.03 (m, 2H), 4.07 (tt, J = 5.5, 1.8 Hz, 2H). **^{13}C NMR** (75 MHz, $CDCl_3$) δ 162.2, 157.9, 135.0, 115.5, 110.3, 43.6.

Preparation of dideuterated 1g (*d*$_2$-1g)

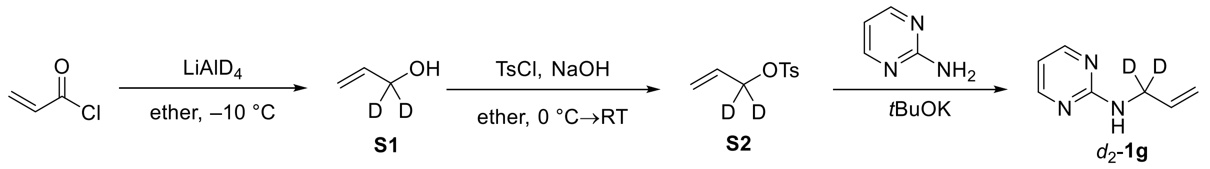

Step 1: Dideuterioallyl alcohol (**S1**) was synthesized by the reported procedure[6] and was used without isolation. 1,1-Dideuterioallyl tolsylate (**S2**) was prepared from **S1** by the reported procedure.[6] **^{1}H NMR** (500 MHz, $CDCl_3$) δ 7.80 (dd, J = 8.4, 1.9 Hz, 1H), 7.35 (d, J = 8.3 Hz, 1H), 5.82 (dd, J = 17.1, 10.3 Hz, 1H), 5.32 (d, J = 17.2 Hz, 1H), 5.26 (d, J = 10.6 Hz, 1H), 2.45 (s, 1H). **^{13}C NMR** (75 MHz, $CDCl_3$) δ 162.1, 158.0, 134.7, 115.9, 110.7, 43.3 (lost). **GC/MS** (EI) (m/z, rel intensity): 55 (100), 107 (8), 110 (82), 135 (49), 214 (1).

Step 2: To a solution of 2-aminopyrimidine (3.33 g, 35.0 mmol) in anhydrous THF (25 mL) was added ^{t}BuOK (3.93 g, 35.0 mmol) in one portion under N_2 at room temperature. After stirring for 1 h, a solution of **S2** (1.50 g, 7.0 mmol) in THF (10 mL) was added dropwise and stirred for 24 h. Then 10 mL of water was added to the mixture and extracted with EtOAc (20 mL × 3). Then the organic phase was dried over anhydrous Na_2SO_4 and concentrated. ***d*$_2$-1g** was obtained as a light yellow liquid (0.46 g, 48%) by column chromatography (Hexane/EA = 5). **^{1}H NMR** (500 MHz, $CDCl_3$) δ 8.29 (d, J = 4.9 Hz, 2H), 6.55 (t, J = 4.8 Hz, 1H), 5.96 (dd, J = 17.2, 10.3 Hz, 1H), 5.34 (s, 1H), 5.26 (dd, J = 17.2, 1.4 Hz, 1H), 5.15 (dd, J = 10.4, 1.4 Hz, 1H). **^{13}C NMR** (75 MHz, $CDCl_3$) δ 162.1, 158.0, 134.7, 115.9, 110.7, 43.6 (very weak). **GC/MS** (EI) (m/z, rel intensity): 53 (58), 79 (72), 110 (82), 120 (68), 121 (100), 136 (83), 137 (92). **HRMS (ESI):** Calc. for $C_7H_8D_2N_3$ $(M+H)^+$: 138.0995; found: 138.0997.

(1h) *N*-Allyl-1,3,5-triazin-2-amine

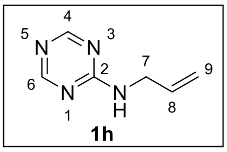

1h is a known compound[7] and was prepared by the allylation of 1,3,5-triazin-2-amine following the same procedure as **1f**. **^{1}H NMR** (400 MHz, $CDCl_3$) δ 8.56(s, 1H), 8.44 (s, 1H), 6.71 (s, 1H), 5.99 – 5.78 (m, 1H), 5.30 – 5.09 (m, 2H), 4.07 (ddd, J = 5.7, 4.1, 1.6 Hz, 2H). **^{13}C NMR** (100 MHz, $CDCl_3$) δ 166.3(C4), 165.5(C6), 164.4(C2), 133.4(C8), 116.7(C9), 43.1(C7). C4 and C6 are inequivalent due to the rotational isomerism of the 1,3,5-triazine system.[8]

(1i) 2-(Allyloxy)pyrimidine

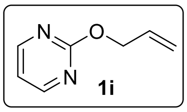

1i is a known compound[9] and was prepared according to the literature procedure: To a cold solution (ice-water bath) of NaOEt (1.63 g, 24 mmol) in allyl alcohol (6.8 mL, 100 mmol) was slowly added 2-chloropyrimidine (2.29 g, 20 mmol). Then the mixture was warmed to room temperature and stirred for 12 h. Diluted with 40 mL of water and extracted with diethyl ether (20 mL×3). The combined organic phase was dried over anhydrous Na_2SO_4. The desired product was obtained as colorless liquid (2.15 g, 79%) by column chromatography (hexane/EA = 10). **^{1}H NMR** (400 MHz, $CDCl_3$) δ 8.46 (d, *J* = 4.8 Hz, 2H), 6.88 (td, *J* = 4.8, 1.1 Hz, 1H), 6.11 – 5.96 (m, 1H), 5.37 (dq, *J* = 17.3, 1.5 Hz, 1H), 5.21 (dq, *J* = 10.6, 1.4 Hz, 1H), 4.88 – 4.81 (m, 2H). **^{13}C NMR** (100 MHz, $CDCl_3$) δ 164.8, 159.1, 132.6, 117.6, 114.9, 67.8.

(1j) 2-(Allyldimethylsilyl)pyrimidine

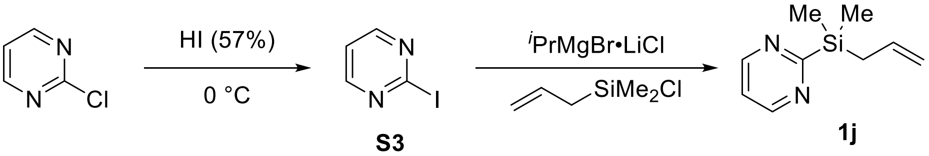

1j was synthesized by a similar procedure reported by Gevorgyan group:[10] To an oven-dried 100 mL Schlenk tube was added 2-iodopyrimidine (**S3**)[11] (1.0 g, 4.85 mmol) and THF (15 mL) under N_2. To this mixture, ^{i}PrMgCl•LiCl (4.2 mL, 1.3 M in THF, 5.34 mmol) was added dropwise at 0 °C. After stirring at 0 °C for 30 min, a solution of allylchlorodimethylsilane (6.0 mL, 5.34 mmol) in THF (10 mL) was added. The reaction was warmed to room temperature and stirred overnight. Quenched with saturated NH_4Cl (aq.) and THF was removed under reduced pressure. The resultant mixture was extracted with EtOAc (10 mL × 3) and dried over anhydrous Na_2SO_4. The desired product was obtained as light yellow liquid (610 mg, 70%) by column chromatography (hexane). **^{1}H NMR** (500 MHz, $CDCl_3$) δ 8.73 (dd, *J* = 5.0, 0.7 Hz, 2H), 7.16 (td, *J* = 5.0, 0.7 Hz, 1H), 5.85 – 5.74 (m, 1H), 4.89 – 4.79 (m, 2H), 1.90 – 1.85 (m, 2H), 0.35 (s, 6H). **^{13}C NMR** (126 MHz, $CDCl_3$) δ 180.1, 155.0, 134.1, 119.8, 113.7, 22.3, -4.3. **HRMS (ESI):** Calc. for $C_9H_{15}N_2Si$ $(M+H)^+$: 179.0999; found: 179.1000.

(1k) *N*-Crotylpyrimidin-2-amine

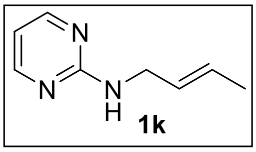

1k was prepared by the similar procedure as **1f** from 2-aminopyrimidine and *trans*-crotyl bromide. **^{1}H NMR** (400 MHz, $CDCl_3$) δ 8.21 (dd, *J* = 4.7, 2.0 Hz, 2H), 6.50 – 6.41 (m, 1H), 5.94 (s, 1H), 5.71 – 5.44 (m, 2H), 4.02 – 3.91(m, 2H), 1.65 (s, 3H). **^{13}C NMR** (126 MHz, $CDCl_3$) δ 162.3, 157.9, 127.6, 110.2, 43.2, 38.3, 17.7. **HRMS (ESI)**: Calc. for $C_8H_{12}N_3$ $(M+H)^+$: 150.1026; found: 150.1019.

(1l) *N*-Cinnamylpyrimidin-2-amine

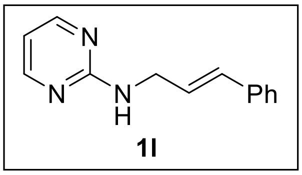

1l was prepared by alkylation of 2-aminopyridine with cinnamyl bromide (1.3 equiv.) in the presence K_2CO_3 (2.0 equiv.) as the base. **^{1}H NMR** (500 MHz, $CDCl_3$) δ 8.31 (d, *J* = 4.9 Hz, 2H), 7.49 – 7.20 (m, 5H), 6.62 (d, *J* = 15.8 Hz, 1H), 6.55 (t, *J* = 4.8 Hz, 1H), 6.42 – 6.30 (m, 1H), 5.77 (s, 1H), 4.26 (t, *J* = 6.0 Hz, 2H). **^{13}C NMR** (126 MHz, $CDCl_3$) δ 162.2, 158.0, 136.8, 131.1, 128.5, 127.4, 126.4, 126.3, 110.6, 43.3. **HRMS (ESI):** Calc. for $C_{13}H_{14}N_3$ $(M+H)^+$: 212.1182; found: 212.1188.

(1m) *N*-(Cyclohex-2-en-1-yl)pyrimidin-2-amine

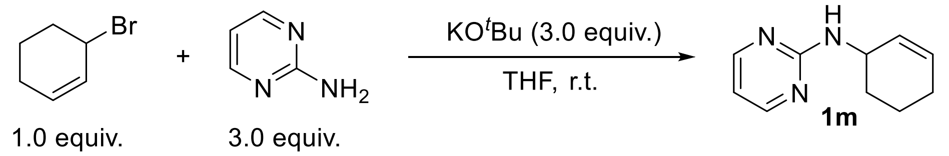

1m was prepared by the similar procedure as **1n** with cyclohexenyl bromide as the limiting reagent. **^{1}H NMR** (500 MHz, $CDCl_3$) δ 8.20 (d, *J* = 4.8 Hz, 2H), 6.43 (t, *J* = 4.8 Hz, 1H), 6.08 (d, *J* = 8.4 Hz, 1H), 5.82 (ddt, *J* = 9.6, 3.9, 2.1 Hz, 1H), 5.68 (dt, *J* = 10.6, 2.8 Hz, 1H), 4.51 (dq, *J* = 8.7, 2.9 Hz, 1H), 2.06 – 1.84 (m, 3H), 1.72 – 1.50 (m, 3H). **^{13}C NMR** (126 MHz, $CDCl_3$) δ 161.7, 157.9, 130.2, 128.4, 110.0, 46.0, 29.3, 24.9, 19.7. **HRMS (ESI):** Calc. for $C_{10}H_{14}N_3$ $(M+H)^+$: 176.1182; found: 176.1180.

(1n) *N*-(But-3-en-1-yl)pyrimidin-2-amine

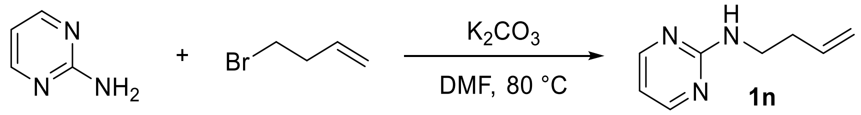

1n is a known compound (CAS 1594998-48-7) and was prepared as the following: To a solution of 2-aminopyrimidine (1.0 g, 10.5 mmol) and K_2CO_3 (2.9 g, 21.0 mmol) in DMF (15 mL) was slowly added 4-bromobut-1-ene (1.2 mL, 11.6 mmol). After addition, the mixture was allowed to stir at 80 °C for 15 h. Then 15 mL of water was added and extracted with ether (15 mL × 3). The combined organic phase was dried over Na_2SO_4 and concentrated. The desired product was purified by column chromatography (Hexane/EA = 6) as light yellow liquid. **^{1}H NMR** (500 MHz, $CDCl_3$) δ 8.24 (d, *J* = 4.8 Hz, 2H), 6.48 (t, *J* = 4.8 Hz, 1H), 5.85 – 5.70 (m, 1H), 5.38 (s, 1H), 5.15 – 4.99 (m, 2H), 3.46 (d, *J* = 6.5 Hz, 2H), 2.36 – 2.34 m, 2H). **^{13}C NMR** (126 MHz, $CDCl_3$) δ 162.3, 157.9, 135.5, 117.0, 110.4, 40.5, 33.7.

(1o) *N*-Allyl-*N*-methylpyrimidin-2-amine

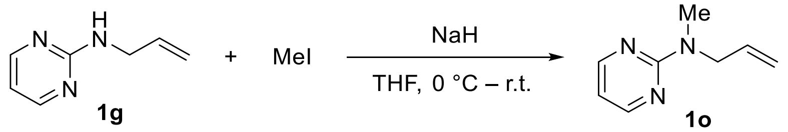

A solution of *N*-allylpyrimidin-2-amine (**1g**) (400 mg, 2.96 mmol) in THF (2 mL) was added slowly to 60% NaH (dispersed in mineral oil) (178 mg, 4.44 mmol) dispersed in THF (6 mL) at 0 °C. After stirring for 30 min, methyl iodide (332 μL, 5.33 mmol) was added through a microsyringe and stirred for another 2 hours. The reaction mixture was quenched with saturated NH_4Cl (15 mL) and THF was removed under reduced pressure. The resultant mixture was extracted with EtOAc (5 mL × 3) and dried over anhydrous Na_2SO_4. Column chromatography (Hexane/EA = 20) gave the pure product as light yellow liquid (411 mg, 93%). **^{1}H NMR** (400 MHz, $CDCl_3$) δ 8.29 (d, *J* = 4.7 Hz, 2H), 6.44 (t, *J* = 4.7 Hz, 1H), 5.90 – 5.81 (m, 1H), 5.19 – 5.08 (m, 2H), 4.26 (dt, *J* = 5.3, 1.6 Hz, 2H), 3.11 (s, 3H). **^{13}C NMR** (100 MHz, $CDCl_3$) δ 161.8, 157.6, 133.6, 116.0, 109.2, 51.4, 34.7. **RMS (ESI):** Calc. for $C_8H_{12}N_3$ $(M+H)^+$: 150.1026; found: 150.1021.

(1p) *N*-Allyl-4,6-dimethylpyrimidin-2-amine

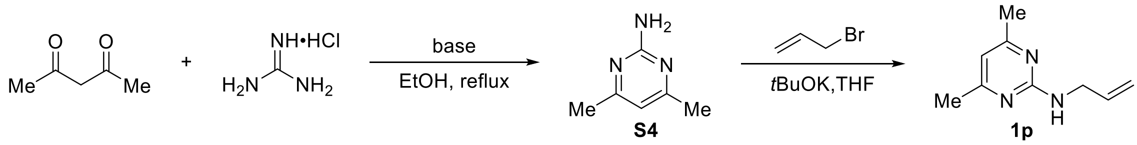

Step 1: 4,6-dimethylpyrimidin-2-amine (**S4**) was synthesized according to the literature: [12] To a solution of 2,4-pentadione (2.0 g, 20 mmol) in EtOH (100 mL) was added guanidine hydrochloride (2.3 g, 24 mmol) and K_2CO_3 (3.3 g, 24 mmol). The mixture was stirred under reflux overnight. Then cooled to room temperature and EtOH was removed under reduced pressure before extraction with EtOAc/H_2O and brine wash. The combined organic phase was dried over anhydrous Na_2SO_4. The crude product was obtained as yellow solid and used directly for the next step. **^{1}H NMR** (500 MHz, $CDCl_3$) δ 6.37 (s, 1H), 5.17 (s, 2H), 2.28 (s, 6H). **^{13}C NMR** (126 MHz, $CDCl_3$) δ 167.8, 162.8, 110.6, 23.7.
Step 2: Performed with the same procedure as **1f**. **1m** was isolated as a yellow solid in 56% yield. **^{1}H NMR** (300 MHz, $CDCl_3$) δ 6.28 (s, 1H), 6.02 – 5.82 (m, 1H), 5.36 – 5.13 (m, 2H), 5.08 (d, *J* = 9.8, 1H), 4.06 (s, 2H), 2.25 (s, 6H). **^{13}C NMR** (75 MHz, $CDCl_3$) δ 167.3, 162.2, 135.4, 115.3, 109.7, 43.7, 23.8. **HRMS (ESI)**: Calc. for $C_9H_{14}N_3$ $(M+H)^+$: 164.1182; found: 164.1186.

(1q) *N*-Allyl-4-methylquinazolin-2-amine

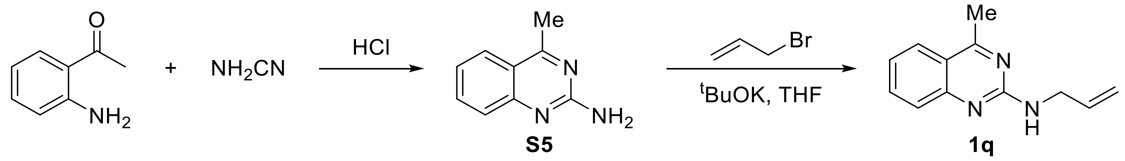

Step 1: 4-Methylquinazolin-2-amine (**S5**) was synthesized according to the known procedure:[13] 2-Aminoacetophenone (2.7 g, 20 mmol) was dissolved in diethyl ether (100 mL) with stirring. Then 4 M HCl in dioxane (12 mL, 48 mmol) was added slowly by using a Pasteur pipette at room temperature. After stirring for 10 minutes, the solvent was rotoevaporated. To the resultant white salt was added cyanamide (5.0 g, 120 mmol) dissolved in 40 mL of diethyl ether and stirred for another 10 minutes. Afterwards, the ether solvent was rotoevaporated again and the resultant brown liquid was heated at 50 °C for 1 h. The reaction mixture was diluted with 100 mL of chloroform and washed sequentially with 5% aq. NaOH (30 mL) and brine (30 mL). Finally, the solvent was rotoevaporated to give light yellow solid which was subjected to the next step directly. The NMR data was in consistent with the literature: [14] **^{1}H NMR** (300 MHz, $CDCl_3$) δ 7.91 (d, *J* = 8.3, 1H), 7.73 – 7.64 (m, 1H), 7.61 – 7.53 (m, 1H), 7.33 – 7.21 (m, 1H), 5.51 (s, 2H), 2.82 (s, 3H). **^{13}C NMR** (75 MHz, $CDCl_3$) δ 170.2, 159.4, 151.6, 133.8, 125.8, 125.3, 122.8, 119.6, 21.5.

Step 2: Performed with the same procedure as **1f**. The desired product was purified by column chromatography (Hexane/EA = 12) as light yellow solid. **^{1}H NMR** (500 MHz, $CDCl_3$) δ 7.82 (dd, *J* = 8.1, 1.4 Hz, 1H), 7.65 – 7.55 (m, 2H), 7.22 – 7.15 (m, 1H), 6.07 – 5.95 (m, 1H), 5.46 (s, 1H), 5.29 (dd, *J* = 17.2, 1.7 Hz, 1H), 5.13 (dd, *J* = 10.3, 1.6 Hz, 1H), 4.20 (s, 1H), 2.74 (s, 3H). **^{13}C NMR** (126 MHz, $CDCl_3$) δ 169.4, 158.7, 151.8, 135.2, 133.5, 126.1, 125.2, 122.2, 119.6, 115.6, 43.8, 21.5. **HRMS (ESI)**: Calc. for $C_{12}H_{14}N_3$ $(M+H)^+$: 200.1182; found: 200.1189.

3. General procedures and reaction optimization.

3.1 General procedure for the 1,3-dicarbofunctionalization.

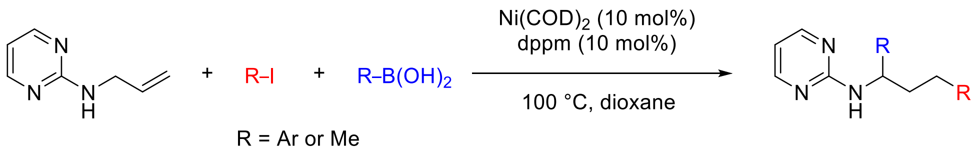

To an oven-dried 10 mL dram vial was added aryl iodide (0.3 mmol), boronic acid (0.3 mmol) and K_3PO_4 (85 mg, 0.400 mmol). Then the vial was taken into glovebox and charged with $Ni(COD)_2$ (5.5 mg, 0.020 mmol) and dppm (7.7 mg, 0.020 mmol). After that, anhydrous dioxane (1.5 mL) was added to the mixture before *N*-allylpyrimidin-2-amine (27 µL, 0.20 mmol) was added through a microsyringe. Finally, the vial was capped and transferred into preheated oil bath at 100 °C and allowed to stir for 18 h. After the reaction was completed, the mixture was diluted with water (0.5 mL) and extracted with EtOAc (2 mL × 3). To the combined organic phase was added 200 mg of silica gel and concentrated under reduced pressure. The desired product was then purified by column chromatography with hexane and EtOAc as the eluent.

3.2 General procedure for the alkynylcarbonation.

To an oven-dried 10 mL dram vial was added boronic acid (0.3 mmol) and K_3PO_4 (85 mg, 0.400 mmol). Then the vial was taken into glovebox and charged with $Ni(COD)_2$ (5.5 mg, 0.020 mmol) and monophosphine ligand (0.020 mmol). After that, anhydrous toluene (1.5 mL) was added to the mixture before *N*-allylpyrimidin-2-amine (27 µL, 0.20 mmol) and alkynyl halides (0.3 mmol) was added through a microsyringe. Finally, the vial was capped and transferred into preheated oil bath at 100 °C and allowed to stir for 18 h. After the reaction was completed, the mixture was diluted with water (0.5 mL) and extracted with EtOAc (2 mL × 3). To the combined organic phase was added 200 mg of silica gel and concentrated under reduced pressure. The desired product was then purified by column chromatography with hexane and EtOAc as the eluent.

3.3 General procedure for the hydrocarbonation.

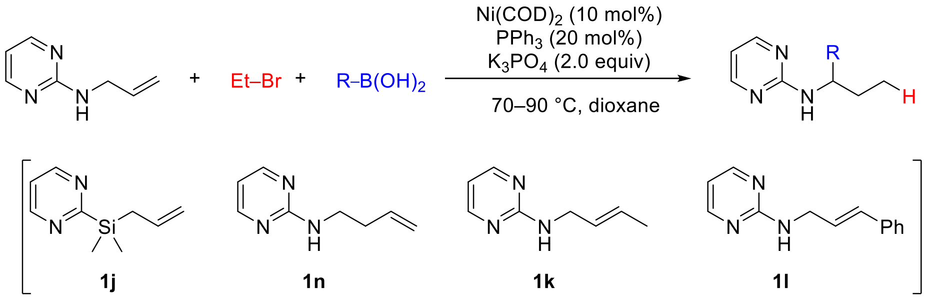

To an oven-dried 10 mL dram vial was added boronic acid (0.3 mmol) and K_3PO_4 (85 mg, 0.400 mmol). Then the vial was taken into glovebox and charged with $Ni(COD)_2$ (5.5 mg, 0.020 mmol) and PPh_3 (10.5, 0.040 mmol). After that, anhydrous dioxane (1.5 mL) was added to the mixture before *N*-allylpyrimidin-2-amine (27 µL, 0.20 mmol) and ethyl bromide (30 µL,0.4 mmol) was added through microsyringes. Finally, the vial was capped and transferred into preheated oil bath at 100 °C and allowed to stir for 18 h. After the reaction was completed, the mixture was diluted with water (0.5 mL) and extracted with EtOAc (2 mL × 3). To the combined organic phase was added 200 mg of silica gel and concentrated under reduced pressure. The desired product was then purified by column chromatography with hexane and EtOAc as the eluent. The optimization was shown in Table S1.

Table S1. Optimization of the hydroarylation of **1j.**

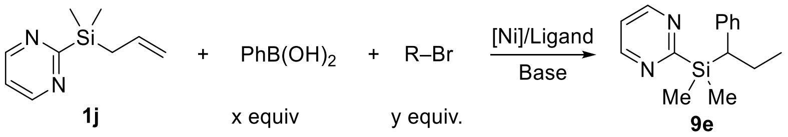

Entry	[Ni] (10 mol%)	Ligand (20/10 mol%)	solvent	T (°C)	$PhB(OH)_2$ (x)	Base (equiv)	R-Br (y)	Yield[b] (%)
1	$Ni(COD)_2$	PPh_3	dioxane	90	1.5	K_3PO_4 (2)	^{i}PrBr (1.5)	60
2	$Ni(COD)_2$	PPh_3	dioxane	90	1.5	0	^{i}PrBr (1.5)	9
3	$Ni(COD)_2$	PPh_3	dioxane	90	1.5	K_3PO_4 (2)	^{i}PrBr (1.5)	52
4	$Ni(COD)_2$	PPh_3	dioxane	90	1.5	K_3PO_4 (1)	^{i}PrBr (1.5)	49
5	$Ni(COD)_2$	PPh_3	THF	90	1.5	K_3PO_4 (2)	^{i}PrBr (1.5)	40
6	$Ni(COD)_2$	PPh_3	dioxane	90	2	K_3PO_4 (2)	^{i}PrBr (3)	83
7	$Ni(COD)_2$	PCy_3	Dioxane	70	1.5	K_3PO_4 (2)	^{i}PrBr (2)	33
8	$Ni(COD)_2$	dppm	Dioxane	70	1.5	K_3PO_4 (2)	^{i}PrBr (2)	15
9	$Ni(COD)_2$	PPh_3	toluene	70	1.5	K_3PO_4 (2)	^{i}PrBr (2)	76
10	$Ni(COD)_2$	PPh_3	MeCN	70	1.5	K_3PO_4 (2)	^{i}PrBr (2)	52
11	$Ni(COD)_2$	PPh_3	Dioxane	70	1.5	K_3PO_4 (2)	CyBr (2)	79
12	$Ni(COD)_2$	PPh_3	Dioxane	70	1.5	K_3PO_4 (2)	^{t}BuBr (2)	69
13	$Ni(COD)_2$	PPh_3	Dioxane	70	1.5	K_3PO_4 (2)	EtBr (2)	86(81)
14	$Ni(COD)_2$	PPh_3	Dioxane	70	1.5	K_3PO_4 (2)	EtBr (0)	0
15	$Ni(COD)_2$	PCy_3	Dioxane	70	1.5	K_3PO_4 (2)	EtBr (2)	55
16	$Ni(COD)_2$	P(*o*-Anisol)$_3$	Dioxane	70	1.5	K_3PO_4 (2)	EtBr (2)	2
17	$Ni(COD)_2$	PEt_3	Dioxane	70	1.5	K_3PO_4 (2)	EtBr (2)	28
18	$Ni(COD)_2$	dppe	Dioxane	70	1.5	K_3PO_4 (2)	EtBr (2)	10
19	$Ni(COD)_2$	dppp	Dioxane	70	1.5	K_3PO_4 (2)	EtBr (2)	9
20	$Ni(COD)_2$	dppb	Dioxane	70	1.5	K_3PO_4 (2)	EtBr (2)	78
21	$Ni(COD)_2$	dppf	Dioxane	70	1.5	K_3PO_4 (2)	EtBr (2)	46
22	$Ni(COD)_2$	IPr	Dioxane	70	1.5	K_3PO_4 (2)	EtBr (2)	3
23	$Ni(COD)_2$	Bpy	Dioxane	70	1.5	K_3PO_4 (2)	EtBr (2)	9
24	$Ni(COD)_2$	*n*-BuPAd$_2$	Dioxane	70	1.5	K_3PO_4 (2)	EtBr (2)	21
25	$Ni(COD)_2$	$P(OPh)_3$	Dioxane	70	1.5	K_3PO_4 (2)	EtBr (2)	3
26	$Ni(COD)_2$	PPh_3	Dioxane	70	1.5	Na_3CO_3(2)	EtBr (2)	61
27	$Ni(COD)_2$	PPh_3	Dioxane	70	1.5	$KHCO_3$ (2)	EtBr (2)	34
28	$Ni(COD)_2$	PPh_3	Dioxane	70	1.5	^{t}BuOK (2)	EtBr (2)	2
29	$Ni(COD)_2$	PPh_3	Dioxane	70	1.5	KOAc (2)	EtBr (2)	0
30	$Ni(COD)_2$	PPh_3	Dioxane	70	1.5	DBU (2)	EtBr (2)	14
31	$Co_2(CO)_8$	PPh_3	Dioxane	70	1.5	K_3PO_4 (2)	EtBr (2)	0

(a) Conditions**: 1j** (0.2 mmol), R-Br (0.4 mmol), $PhB(OH)_2$ (0.3 mmol), dioxane (1.5 mL), 18 h.

(b) Determined by GC using *n*-hexadecane as the internal standard. Isolated yield was given in the brackets.

4. Characterization data of the difunctionalization products.

4.1 Diarylation and arylalkenylation products 2-4.

(2g') *N*-(1,3-Diphenylpropyl)pyrimidin-2-amine

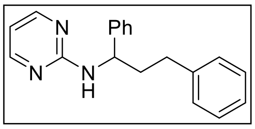

2g' was synthesized according to the general procedure. Yield 39.5 mg (68%) of a colorless oil (hexane/EtOAc = 8/1).

^{1}H NMR (400 MHz, $CDCl_3$) δ 8.17 (d, *J* = 4.8 Hz, 2H), 7.50 – 7.41 (m, 2H), 7.31 – 7.20 (m, 4H), 7.20 – 7.11 (m, 3H), 6.45 (t, *J* = 4.8 Hz, 1H), 5.77 (d, *J* = 8.5 Hz, 1H), 5.02 (td, *J* = 8.2, 6.5 Hz, 1H), 2.78 – 2.56 (m, 2H), 2.21 – 2.00 (m, 2H). **^{13}C NMR** (126 MHz, $CDCl_3$) δ 161.9, 158.0, 143.3, 141.5, 128.5, 128.3, 128.3, 127.0, 126.4, 125.9, 110.7, 54.8, 39.0, 32.7. **HRMS (ESI):** Calc. for $C_{19}H_{20}N_3$ $(M+H)^+$: 290.1652; found: 290.1657.

(3a) *N*-(1-Phenyl-3-(*p*-tolyl)propyl)pyrimidin-2-amine

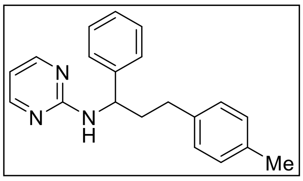

3a was synthesized according to the general procedure. Yield 38.9 mg (64%) of a light yellow oil (hexane/EtOAc = 8/1).

^{1}H NMR (300 MHz, $CDCl_3$) δ 8.24 (d, *J* = 4.8 Hz, 2H), 7.39 – 7.23 (m, 5H), 7.11 – 7.00 (m, 4H), 6.50 (t, *J* = 4.8 Hz, 1H), 5.58 (d, *J* = 8.4 Hz, 1H), 5.10 (q, *J* = 7.7 Hz, 1H), 2.78 – 2.50 (m, 2H), 2.31 (s, 3H), 2.21 – 2.08 (m, 2H). **^{13}C NMR** (75 MHz, $CDCl_3$) δ 161.9, 158.0, 143.3, 138.5, 135.3, 129.1, 128.5, 128.2, 127.0, 126.4, 110.8, 54.8, 39.2, 32.2, 21.0. **HRMS (ESI):** Calc. for $C_{19}H_{22}N_3$ $(M+H)^+$: 340.1808; found: 340.1812.

(3b) *N*-(3-(4-Methoxyphenyl)-1-phenylpropyl)pyrimidin-2-amine

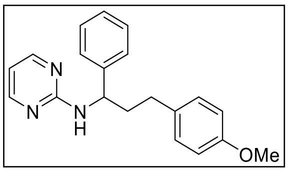

3b was synthesized according to the general procedure. Yield 28.8 mg (45%) of a colorless oil (hexane/EtOAc = 6/1).

^{1}H NMR (400 MHz, $CDCl_3$) δ 8.19 (d, *J* = 4.8 Hz, 2H), 7.36 – 7.17 (m, 5H), 7.06 – 6.99 (m, 2H), 6.81 – 6.73 (m, 2H), 6.45 (t, *J* = 4.8 Hz, 1H), 5.85 (d, *J* = 8.2 Hz, 1H), 5.07 (td, *J* = 8.1, 6.4 Hz, 1H), 3.74 (s, 3H), 2.72 – 2.51 (m, 2H), 2.17 – 2.03 (m, 2H). **^{1}H NMR** (400 MHz, $CDCl_3$) δ 8.26 (d, *J* = 5.0 Hz, 2H), 7.40 – 7.24 (m, 5H), 7.07 (d, *J* = 8.7 Hz, 1H), 6.80 (d, *J* = 8.7 Hz, 1H), 6.55 (t, *J* = 4.9 Hz, 1H), 6.50 (s, 1H), 5.09 (q, *J* = 8.1 Hz, 1H), 3.78 (s, 3H), 2.76 – 2.53 (m, 2H), 2.21 – 2.10 (m, 2H). **^{13}C NMR** (100 MHz, $CDCl_3$) δ 160.2, 157.9, 142.7, 133.4, 129.3, 128.6, 128.5, 127.2, 126.5, 113.8, 110.3, 55.2, 55.0, 39.1, 31.6. **HRMS (ESI):** Calc. for $C_{20}H_{22}N_3O$ $(M+H)^+$: 320.1757; found: 320.1761.

(3c) *N*-(1-Phenyl-3-(4-(trifluoromethyl)phenyl)propyl)pyrimidin-2-amine

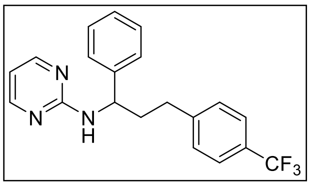

3c was synthesized according to the general procedure. Yield 41.5 mg (58%) of a pale white wax (hexane/EtOAc = 7/1).

^{1}H NMR (400 MHz, $CDCl_3$) δ 8.19 (d, *J* = 4.9 Hz, 2H), 7.46 (d, *J* = 7.9 Hz, 2H), 7.39 – 7.08 (m, 7H), 6.46 (t, *J* = 4.7 Hz, 1H), 5.82 – 5.67 (m, 1H), 5.15 – 4.96 (m, 1H), 2.84 – 2.56 (m, 2H), 2.27 – 2.02 (m, 2H). **^{13}C NMR** (126 MHz, $CDCl_3$) δ 161.7, 158.0, 145.6, 142.9, 128.7, 128.6, 128.3 (d, $J_{F\text{-}C}$ = 32.4 Hz), 127.3, 126.4, 125.3 (q, $J_{F\text{-}C}$ = 3.7 Hz), 124.3 (q, $J_{F\text{-}C}$ = 272 Hz) 110.9, 54.6, 38.5, 32.5. **^{19}F NMR** (376 MHz, $CDCl_3$) δ -62.3. **HRMS (ESI):** Calc. for $C_{20}H_{19}F_3N_3$ $(M+H)^+$: 358.1526; found: 358.1526.

(3d) 4-(3-Phenyl-3-(pyrimidin-2-ylamino)propyl)benzonitrile

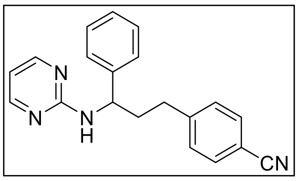

3d was synthesized according to the general procedure, reaction temperature was 85 °C. Yield 37.7 mg (60%) of a light yellow oil (hexane/EtOAc = 4/1).
^{1}H NMR (500 MHz, CDCl$_3$) δ 8.23 (d, *J* = 4.7, 2H), 7.54 (dd, *J* = 8.2, 1.8 Hz, 2H), 7.37 – 7.30 (m, 4H), 7.29 – 7.22 (m, 3H), 6.52 (td, *J* = 4.7, 1.8 Hz, 1H), 5.84 (d, *J* = 8.4 Hz, 1H), 5.09 (td, *J* = 8.3, 6.4 Hz, 1H), 2.82 - 2.59 (m, 2H), 2.26 – 2.10 (m, 2H). **^{13}C NMR (125 MHz, CDCl$_3$)** δ 161.7, 158.0, 147.2, 142.6, 132.2, 129.2, 128.7, 127.3, 126.4, 119.0, 110.9, 109.8, 54.6, 38.2, 32.8. **HRMS (ESI):** Calc. for $C_{20}H_{19}N_4$ $(M+H)^+$: 315.1604; found: 315.1610.

(3e) Methyl 4-(3-phenyl-3-(pyrimidin-2-ylamino)propyl)benzoate

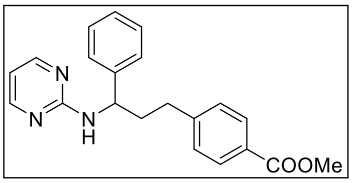

3e was synthesized according to the general procedure, reaction temperature was 90 °C. Yield 55.7 mg (80%) of a pale yellow oil (hexane/EtOAc = 5/1).
^{1}H NMR (400 MHz, CDCl$_3$) δ 8.20 (d, *J* = 4.9 Hz, 2H), 7.91 – 7.80 (m, 2H), 7.29 – 7.27 (m, 4H), 7.23 – 7.10 (m, 5H), 6.51 (d, *J* = 4.9 Hz, 1H), 6.43 (s, 1H), 5.03 (q, *J* = 7.8 Hz, 1H), 3.83 (s, 3H), 2.76 – 2.60 (m, 2H), 2.21 – 2.13 (m, 2H). **^{13}C NMR** (100 MHz, CDCl$_3$) δ 167.1, 161.8, 158.0, 147.1, 142.9, 129.8, 128.6, 128.41, 128.37, 127.2, 126.4, 110.9, 54.7, 51.9, 38.4, 32.7. **HRMS (ESI):** Calc. for $C_{21}H_{22}N_3O_2$ $(M+H)^+$: 348.1707; found: 348.1711.

(3f) *N*-(1-Phenyl-3-(4-(prop-1-en-2-yl)phenyl)propyl)pyrimidin-2-amine

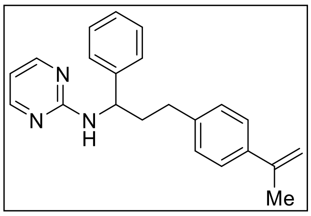

3f was synthesized according to the general procedure. Yield 43.5 mg (66%) of a colorless oil (hexane/EtOAc = 8/1).
^{1}H NMR (500 MHz, CDCl$_3$) δ 8.26 (d, *J* = 4.7 Hz, 2H), 7.45 – 7.37 (m, 4H), 7.36 (t, *J* = 7.5 Hz, 2H), 7.32 – 7.23 (m, 1H), 7.14 (d, *J* = 7.9 Hz, 2H), 6.52 (t, *J* = 4.8 Hz, 1H), 6.12 (d, *J* = 8.4 Hz, 1H), 5.38 (d, *J* = 1.5 Hz, 1H), 5.17 (q, *J* = 7.9 Hz, 1H), 5.08 (q, *J* = 1.4 Hz, 1H), 2.85 – 2.64 (m, 2H), 2.22 (td, *J* = 9.6, 4.2 Hz, 2H), 2.17 (s, 3H). **^{13}C NMR** (75 MHz, CDCl$_3$) δ 161.9, 157.9, 143.3, 143.0, 140.8, 138.8, 128.5, 128.4, 128.2, 127.0, 125.5, 111.7, 110.7, 54.7, 38.9, 32.3, 21.8. **HRMS (ESI):** Calc. for $C_{22}H_{24}N_3$ $(M+H)^+$: 330.1965; found: 330.1974.

(3g) 1-(4-(3-Phenyl-3-(pyrimidin-2-ylamino)propyl)phenyl)ethan-1-one

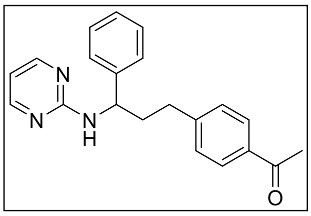

3g was synthesized according to the general procedure. Yield 43.8 mg (66%) of a colorless oil (hexane/EtOAc = 5/1).
^{1}H NMR (400 MHz, CDCl$_3$) δ 8.17 (d, *J* = 4.8 Hz, 2H), 7.78 (d, *J* = 8.2 Hz, 2H), 7.33 – 7.17 (m, 4H), 7.22 – 7.11 (m, 3H), 6.45 (t, *J* = 4.8 Hz, 1H), 5.80 (d, *J* = 8.4 Hz, 1H), 5.03 (q, *J* = 7.7 Hz, 1H), 2.79 – 2.55 (m, 2H), 2.50 (s, 3H), 2.22 – 2.02 (m, 2H). **^{13}C NMR** (100 MHz, CDCl$_3$) δ 197.8, 161.4, 157.9, 147.3, 142.8, 135.1, 128.6, 128.5, 127.2, 126.4, 115.5, 110.8, 54.7, 38.4, 32.6, 26.5. **HRMS (ESI):** Calc. for $C_{21}H_{22}N_3O$ $(M+H)^+$: 332.1757; found: 332.1761.

(3h) *N*-(3-(3-Methoxyphenyl)-1-phenylpropyl)pyrimidin-2-amine

3h was synthesized according to the general procedure. Reaction temperature was 110 °C. Yield 44.0 mg (69%) of a colorless oil (hexane/EtOAc = 10/1).

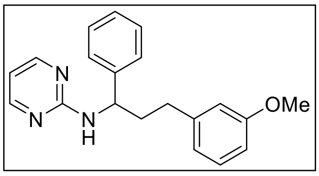

^{1}H NMR (400 MHz, $CDCl_3$) δ 8.24 (d, *J* = 4.9 Hz, 2H), 7.39 – 7.28 (m, 4H), 7.25 – 7.14 (m, 2H), 6.78 – 6.66 (m, 3H), 6.50 (t, *J* = 4.8 Hz, 1H), 5.82 (d, *J* = 8.4 Hz, 1H), 5.11 (td, *J* = 8.2, 6.4 Hz, 1H), 3.77 (s, 3H), 2.79 – 2.56 (m, 2H), 2.24 – 2.12 (m, 2H). **^{13}C NMR** (100 MHz, $CDCl_3$) δ 161.6, 159.6, 157.9, 143.16, 143.14, 129.3, 128.5, 127.1, 126.4, 120.7, 114.1, 111.3, 110.7, 55.1, 54.8, 38.9, 32.7. **HRMS (ESI):** Calc. for $C_{20}H_{22}N_3O$ $(M+H)^+$: 320.1757; found: 320.1757.

(3i) *N*-(3-(Naphthalen-1-yl)-1-phenylpropyl)pyrimidin-2-amine

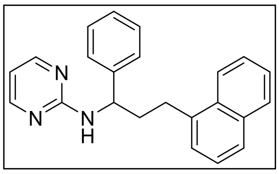

3i was synthesized according to the general procedure. Reaction temperature was 110 °C. Yield 33.9 mg (50%) of a colorless oil (hexane/EtOAc = 10/1).

^{1}H NMR (400 MHz, $CDCl_3$) δ 8.19 (d, *J* = 4.9 Hz, 2H), 7.86 – 7.70 (m, 2H), 7.62 (d, *J* = 8.1 Hz, 1H), 7.43 – 7.14 (m, 9H), 6.45 (t, *J* = 4.8 Hz, 1H), 5.80 (d, *J* = 8.4 Hz, 1H), 5.21 – 5.08 (m, 1H), 3.22 – 2.94 (m, 2H), 2.32 – 2.15 (m, 2H). **^{13}C NMR** (100 MHz, $CDCl_3$) δ 161.6, 157.9, 143.0, 137.7, 133.9, 131.7, 128.8, 128.6, 127.2, 126.7, 126.5, 125.9, 125.8, 125.5, 125.4, 123.6, 110.8, 55.3, 38.3, 29.8. **HRMS (ESI):** Calc. for $C_{21}H_{22}N_3O_2$ $(M+H)^+$: 348.1707; found: 348.1716.

(3j) *N*-(1-Phenyl-3-(o-tolyl)propyl)pyrimidin-2-amine

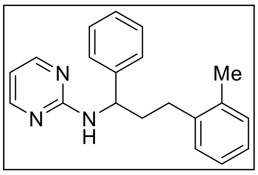

3j was synthesized according to the general procedure. Reaction temperature was 110 °C. Yield 15.4 mg (25%) of a colorless oil (hexane/EtOAc = 12/1).

^{1}H NMR (400 MHz, $CDCl_3$) δ 8.16 (d, *J* = 4.8 Hz, 2H), 7.35 – 6.99 (m, 9H), 6.41 (dt, *J* = 9.4, 4.8 Hz, 1H), 5.68 (d, *J* = 8.4 Hz, 1H), 5.12 – 4.99 (m, 1H), 2.74 – 2.46 (m, 2H), 2.14 (s, 3H), 2.12 – 1.94 (m, 1H). **^{13}C NMR** (100 MHz, $CDCl_3$) δ 161.8, 157.9, 143.2, 139.8, 135.8, 130.2, 128.7, 128.5, 127.1, 126.5, 126.4, 126.0, 110.8, 55.2, 37.8, 30.1, 19.1. **HRMS (ESI):** Calc. for $C_{20}H_{22}N_3$ $(M+H)^+$: 304.1808; found: 304.1813.

(3k) Methyl 2-(3-phenyl-3-(pyrimidin-2-ylamino)propyl)benzoate

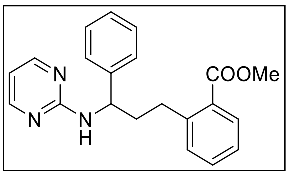

3k was synthesized according to the general procedure. Yield 21.6 mg (31%) of a colorless oil (hexane/EtOAc = 10/1).

^{1}H NMR (400 MHz, $CDCl_3$) δ 8.17 (d, *J* = 4.8 Hz, 2H), 7.82 (dd, *J* = 7.8, 1.5 Hz, 1H), 7.36 – 7.27 (m, 3H), 7.27 – 7.06 (m, 5H), 6.41 (t, *J* = 4.8 Hz, 1H), 5.93 (d, *J* = 7.8 Hz, 1H), 5.04 (q, *J* = 7.4 Hz, 1H), 3.80 (s, 3H), 3.18 – 3.06 (m, 1H), 2.90 – 2.80 (m, 1H), 2.11 – 2.01 (m, 2H). **^{13}C NMR** (100 MHz, $CDCl_3$) δ 167.8, 162.0, 157.9, 143.8, 143.7, 132.1, 131.1, 130.9, 129.2, 128.4, 126.9, 126.3, 126.1, 110.6, 55.3, 52.0, 39.6, 31.9. **HRMS (ESI):** Calc. for $C_{21}H_{22}N_3O_2$ $(M+H)^+$: 348.1707; found: 348.1716.

(3l) *N*-(3-Phenyl-1-(4-(trifluoromethyl)phenyl)propyl)pyrimidin-2-amine

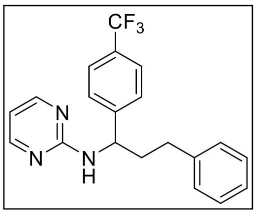

3l was synthesized according to the general procedure. Yield 45.1 mg (63%) of a colorless oil (hexane/EtOAc = 8/1).

^{1}H NMR (400 MHz, $CDCl_3$) δ 8.24 (d, *J* = 4.8 Hz, 2H), 7.59 – 7.54 (m, 2H), 7.47 (d, *J* = 8.1 Hz, 2H), 7.32 – 7.26 (m, 2H), 7.26 – 7.10 (m, 3H), 6.54 (t, *J* = 4.8 Hz, 1H), 5.76 (d, *J* = 8.1 Hz, 1H), 5.13 (q, *J* = 7.6 Hz, 1H), 2.83 – 2.61 (m, 2H), 2.25 – 2.11 (m, 2H). **^{13}C NMR** (100 MHz, $CDCl_3$) δ 161.5, 158.0, 147.6, 141.0, 128.66, 128.62, 128.6 (q, $J_{F\text{-}C}$ = 33 Hz), 126.7, 126.1, 125.5 (q, $J_{F\text{-}C}$ = 3.5 Hz), 124.3 (q, $J_{F\text{-}C}$ = 272 Hz), 111.1, 54.5, 39.0, 32.6. **^{19}F NMR** (376 MHz, $CDCl_3$) δ -62.4. **HRMS (ESI):**

Calc. for $C_{20}H_{19}F_3N_3$ $(M+H)^+$: 358.1526; found: 358.1530. The structure was confirmed by the 2D NMR (Figure S 15 and 16).

(3m) *N*-(1-(4-Bromophenyl)-3-(p-tolyl)propyl)pyrimidin-2-amine

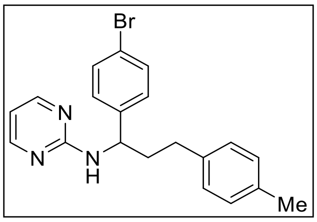

3m was synthesized according to the general procedure. Yield 33.7 mg (44%) of a colorless oil (hexane/EtOAc = 10/1).

^{1}H NMR (400 MHz, $CDCl_3$) δ 8.15 (d, *J* = 4.8 Hz, 2H), 7.57 – 6.90 (aromatic, 8H), 6.43 (t, *J* = 4.8 Hz, 1H), 5.62 (d, *J* = 8.2 Hz, 1H), 4.94 (q, *J* = 7.8, 1H), 2.72 – 2.44 (m, 2H), 2.23 (s, 3H), 2.11 – 1.96 (m, 2H). **^{13}C NMR** (100 MHz, $CDCl_3$) δ 161.7, 158.0, 142.6, 138.1, 137.5, 135.5, 131.5, 129.1, 128.2, 120.7, 111.0, 54.3, 39.0, 32.1, 21.0. **HRMS (ESI)**: Calc. for $C_{20}H_{21}BrN_3$ $(M+H)^+$: 382.0913 (^{79}Br) and 384.0898 (^{81}Br); found: 382.0920 (^{79}Br) and 384.0901 (^{81}Br).

(3n) *N*-(1-(4-(tert-Butyl)phenyl)-3-(4-chlorophenyl)propyl)pyrimidin-2-amine

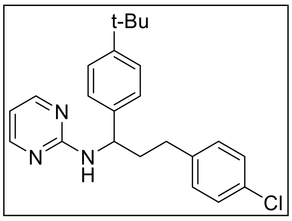

3n was synthesized according to the general procedure. Yield 49.4 mg (68%) of a light yellow oil (hexane/EtOAc = 6/1).

^{1}H NMR (400 MHz, $CDCl_3$) δ 8.24 (d, *J* = 4.8 Hz, 2H), 7.37 – 7.31 (m, 2H), 7.29 – 7.25 (m, 2H), 7.24 – 7.19 (m, 2H), 7.10 – 7.04 (m, 2H), 6.50 (t, *J* = 4.8 Hz, 1H), 5.51 (d, *J* = 8.7 Hz, 1H), 5.09 (td, *J* = 8.2, 6.4 Hz, 1H), 2.76 – 2.56 (m, 2H), 2.17 – 2.06 (m, 2H), 1.30 (s, 9H). **^{13}C NMR** (100 MHz, $CDCl_3$) δ 161.9, 158.0, 150.0, 140.1, 139.8, 131.5, 129.7, 128.4, 126.0, 125.5, 110.8, 54.2, 38.7, 34.4, 32.0, 31.3. **HRMS (ESI):** Calc. for $C_{23}H_{27}ClN_3$ $(M+H)^+$: 380.1888; found: 380.1890.

(3o) Methyl 4-(3-(pyrimidin-2-ylamino)-3-(4-vinylphenyl)propyl)benzoate

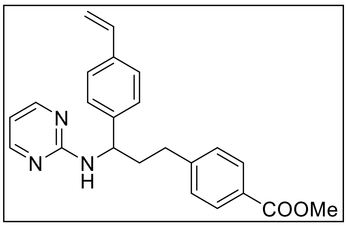

3o was synthesized according to the general procedure.Yield 22.0 mg (29%) of a colorless oil (hexane/EtOAc = 5/1).

^{1}H NMR (500 MHz, $CDCl_3$) δ 8.24 (d, *J* = 4.9 Hz, 2H), 7.93 (d, *J* = 8.0 Hz, 2H), 7.40 – 7.28 (m, 4H), 7.21 (d, *J* = 8.0 Hz, 2H), 6.69 (dd, *J* = 17.6, 10.9 Hz, 1H), 6.52 (t, *J* = 4.7 Hz, 1H), 5.78 (d, *J* = 8.4 Hz, 1H), 5.72 (d, *J* = 17.5 Hz, 1H), 5.22 (dd, *J* = 10.9, 0.9 Hz, 1H), 5.07 (q, *J* = 7.7 Hz, 1H), 3.89 (s, 2H), 2.83 – 2.65 (m, 2H), 2.26 – 2.10 (m, 2H). **^{13}C NMR** (126 MHz, $CDCl_3$) δ 167.1, 161.5, 157.9, 147.0, 142.5, 136.6, 136.4, 129.7, 128.4, 128.0, 126.6, 126.5, 113.7, 110.9, 54.5, 52.0, 38.3, 32.6. **HRMS (ESI):** Calc. for $C_{23}H_{24}N_3O_2$ $(M+H)^+$: 374.1863; found: 374.1863.

(3p) *N*-(1-(3-Chlorophenyl)-3-(3-methoxyphenyl)propyl)pyrimidin-2-amine

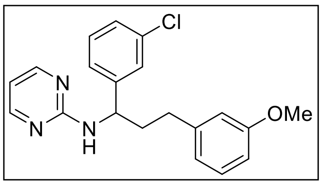

3p was synthesized according to the general procedure. Reaction temperature was 110 °C. Yield 45.3 mg (64%) of a colorless oil (hexane/EtOAc = 8/1).

^{1}H NMR (500 MHz, $CDCl_3$) δ 8.25 (d, *J* = 5.4 Hz, 2H), 7.36 – 7.30 (m, 1H), 7.25 – 7.15 (m, 4H), 6.78 – 6.63 (m, 3H), 6.53 (q, *J* = 6.1, 5.5 Hz, 1H), 5.75 (d, *J* = 8.5 Hz, 1H), 5.10 – 4.98 (m, 1H), 3.80 – 3.72 (m, 3H), 2.78 – 2.58 (m, 2H), 2.18 – 2.09 (m, 2H). **^{13}C NMR** (126 MHz, $CDCl_3$) δ 161.5, 159.7, 157.9, 145.5, 142.7, 134.4, 129.8, 129.4, 127.2, 126.7, 124.6, 120.7, 114.1, 111.5, 111.1, 55.1, 54.4, 38.8, 32.6. **HRMS (ESI):** Calc. for $C_{20}H_{21}ClN_3O$ $(M+H)^+$: 354.1368; found: 354.1368.

(3q) *N*-(3-(4-Fluorophenyl)-1-(naphthalen-2-yl)propyl)pyrimidin-2-amine

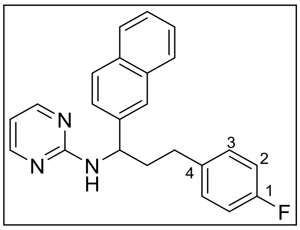

3q was synthesized according to the general procedure. Yield 52.8 mg (74%) of a colorless oil (hexane/EtOAc = 10/1).

^{1}H NMR (400 MHz, $CDCl_3$) δ 8.25 (d, *J* = 4.8 Hz, 2H), 7.85 – 7.76 (m, 4H), 7.51 – 7.40 (m, 3H), 7.11 (dd, *J* = 8.6, 5.5 Hz, 2H), 6.94 (t, *J* = 8.7 Hz, 2H), 6.52 (t, *J* = 4.8 Hz, 1H), 5.87 (d, *J* = 8.4 Hz, 1H), 5.24 (q, *J* = 7.6 Hz, 1H), 2.80 – 2.60 (m, 2H), 2.25 (tt, *J* = 9.5, 6.4 Hz, 2H). **^{13}C NMR** (100 MHz, $CDCl_3$) δ 162.0 (d, $J_{\text{F-C1}}$ = 247.3 Hz), 161.3, 157.8, 140.3, 136.9, 133.4, 132.7, 129.7 (d, $J_{\text{F-C3}}$ = 7.9 Hz), 128.5, 127.8, 127.6, 126.1, 125.7, 125.1, 124.7, 115.2 (d, $J_{\text{F-C2}}$ = 21.2 Hz), 110.8, 54.9, 39.0, 31.8. **^{19}F NMR** (376 MHz, $CDCl_3$) δ -117.5. **HRMS (ESI):** Calc. for $C_{23}H_{21}F_3N_3$ $(M+H)^+$: 358.1714; found: 358.1717.

(3r) 4-(3-(3,4-Difluorophenyl)-3-(pyrimidin-2-ylamino)propyl)benzonitrile

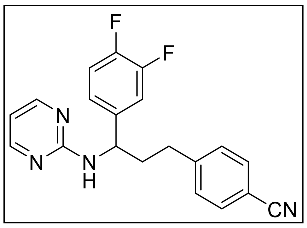

3r was synthesized according to the general procedure. Reaction temperature was 85 °C. Yield 48.9 mg (70%) of a colorless oil (hexane/EtOAc = 4/1).

^{1}H NMR (400 MHz, $CDCl_3$) δ 8.25 (d, *J* = 4.7 Hz, 2H), 7.55 (d, *J* = 8.2 Hz, 2H), 7.24 (d, *J* = 8.0 Hz, 2H), 7.20 – 7.00 (m, 3H), 6.57 (t, *J* = 4.8 Hz, 1H), 5.78 (d, *J* = 9.8 Hz, 1H), 5.02 (td, *J* = 8.2, 6.1 Hz, 1H), 2.87 – 2.66 (m, 2H), 2.24 – 2.04 (m, 2H). **^{13}C NMR** (100 MHz, $CDCl_3$) δ 161.4, 158.0, 151.1 (dd, $J_{\text{F-C}}$ = 91.2, 14.4 Hz), 149.4 (dd, $J_{\text{F-C}}$ = 91.2, 12.2 Hz), 146.6, 140.2 – 140.0 (m), 132.3, 129.1, 122.3 (dd, $J_{\text{F-C}}$ = 6.2, 3.6 Hz), 118.9, 117.4 (d, $J_{\text{F-C}}$ = 17.2 Hz), 115.4 (d, $J_{\text{F-C}}$ = 17.6 Hz), 111.4, 110.1, 53.7, 38.1, 32.7. **^{19}F NMR** (376 MHz, $CDCl_3$) δ -137.0 – -137.2 (m), -139.7 – -139.9 (m). **HRMS (ESI):** Calc. for $C_{20}H_{17}F_2N_4$ $(M+H)^+$: 351.1416; found: 351.1416.

(3s) *N*-(1-(3-Chloro-4-fluorophenyl)-3-(3,5-dimethylphenyl)propyl)pyrimidin-2-amine

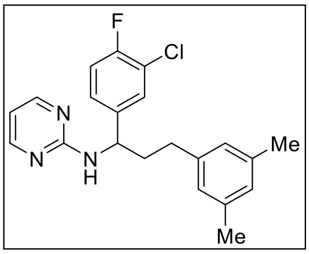

3s was synthesized according to the general procedure. Yield 23.7 mg (32%) of a colorless oil. (hexane/EtOAc = 8/1).

^{1}H NMR (400 MHz, $CDCl_3$) δ 8.28 (d, *J* = 4.8 Hz, 2H), 7.41 (dd, *J* = 7.0, 2.3 Hz, 1H), 7.29 (s, 1H), 7.26 – 7.22 (m, 1H), 7.09 (t, *J* = 8.7 Hz, 1H), 6.85 (s, 1H), 6.77 (s, 2H), 6.57 (t, *J* = 4.8 Hz, 1H), 5.56 (d, *J* = 8.0 Hz, 1H), 5.02 (q, *J* = 7.6 Hz, 1H), 2.76 – 2.54 (m, 2H), 2.29 (s, 6H), 2.07 – 2.14 (m, 2H). **^{13}C NMR** (100 MHz, $CDCl_3$) δ 161.7, 158.0, 157.1 (q, $J_{\text{F-C}}$ = 220.5 Hz), 140.9, 140.8, 137.9, 128.7, 127.7, 126.2, 126.1 (q, $J_{\text{F-C}}$ = 7.6 Hz), 120.8 (d, $J_{F\text{-}C}$ = 17.7 Hz), 116.4 (d, $J_{F\text{-}C}$ = 21.1 Hz), 111.2, 54.0, 39.1, 32.4, 21.2. **^{19}F NMR** (376 MHz, $CDCl_3$) δ -118.2. **HRMS (ESI):** Calc. for $C_{21}H_{22}ClFN_3$ $(M+H)^+$: 370.1481; found: 370.1482.

(3t) *N*-(1-(3,5-Bis(trifluoromethyl)phenyl)-3-(*p*-tolyl)propyl)pyrimidin-2-amine

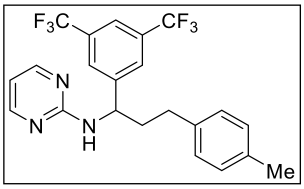

3t was synthesized according to the general procedure. Yield 50.1 mg (57%) of a yellow oil (hexane/EtOAc = 10/1).

^{1}H NMR (400 MHz, $CDCl_3$) δ 8.25 (d, *J* = 4.8 Hz, 2H), 7.80 (d, *J* = 1.6 Hz, 2H), 7.74 (d, *J* = 1.9 Hz, 1H), 7.12 – 6.98 (m, 4H), 6.57 (t, *J* = 4.8 Hz, 1H), 5.87 (d, *J* = 7.6 Hz, 1H), 5.13 (td, *J* = 8.1, 6.2 Hz, 1H), 2.80 – 2.62 (m, 2H), 2.31 (s, 3H), 2.23 – 2.10 (m, 2H). **^{13}C NMR** (100 MHz, $CDCl_3$) δ 161.5, 158.0, 146.6, 137.4, 135.8, 131.6 (q, *J* = 33.3 Hz), 129.3, 128.2, 126.8, 123.4 (q, *J* = 273.5 Hz),

121.1 – 120.9 (m), 111.6, 54.4, 39.0, 32.1, 21.0. **^{19}F NMR** (376 MHz, $CDCl_3$) δ -62.8. **HRMS (ESI):** Calc. for $C_{22}H_{20}F_6N_3$ $(M+H)^+$: 440.1556; found: 440.1554.

(3u) *N*-(1-(3,5-Bis(trifluoromethyl)phenyl)-3-(4-acetylphenyl)propyl)pyrimidin-2-amine

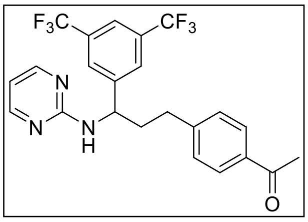

3u was synthesized according to the general procedure. Reaction temperature was 90 °C. Yield 59.8 mg (64%) of a colorless oil (hexane/EtOAc = 5/1).

^{1}H NMR (400 MHz, $CDCl_3$) δ 8.25 (d, *J* = 4.9 Hz, 2H), 7.91 – 7.83 (m, 2H), 7.84 – 7.71 (m, 3H), 7.25 – 7.21 (m, 2H), 6.59 (t, *J* = 4.8 Hz, 1H), 5.94 (d, *J* = 7.8 Hz, 1H), 5.14 (td, *J* = 8.3, 6.0 Hz, 1H), 2.92 – 2.72 (m, 2H), 2.58 (s, 3H), 2.26 – 2.15 (m, 2H). **^{13}C NMR** (100 MHz, $CDCl_3$) δ 197.7, 161.1, 158.0, 146.13, 146.10, 135.5, 131.8 (q, *J* = 33.2 Hz), 128.7, 128.6, 126.72(q, *J* = 4.0 Hz) 123.3 (q, *J* = 252.2 Hz) (121.4 – 121.0 (m), 111.7, 54.2, 38.3, 32.6, 26.5. **^{19}F NMR** (376 MHz, $CDCl_3$) δ -62.8. **HRMS (ESI):** Calc. for $C_{23}H_{20}F_6N_3O$ $(M+H)^+$: 468.1505; found: 468.1509.

(4a) (*E*)-*N*-(1,5-Diphenylpent-1-en-3-yl)pyrimidin-2-amine

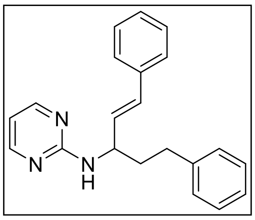

4a was synthesized according to the general procedure. Yield 42.9 mg (68%) of a yellowish oil. (hexane/EtOAc = 6/1).

^{1}H NMR (400 MHz, $CDCl_3$) δ 8.22 (d, *J* = 4.8 Hz, 2H), 7.32 – 7.20 (m, 6H), 7.17 – 7.09 (m, 4H), 6.56 – 6.50 (m, 1H), 6.48 (t, *J* = 4.8 Hz, 1H), 6.16 (dd, *J* = 15.9, 6.0 Hz, 1H), 5.20 (d, *J* = 9.0 Hz, 1H), 4.73 (t, *J* = 7.4 Hz, 1H), 2.74 – 2.68 (m, 2H), 2.03 – 1.87 (m, 2H). **^{13}C NMR** (100 MHz, $CDCl_3$) δ 162.1, 158.0, 141.6, 136.9, 130.6, 129.9, 128.5, 128.4, 128.4, 127.4, 126.4, 126.3, 125.9, 110.8, 52.3, 37.4, 32.3. **HRMS (ESI):** Calc. for $C_{21}H_{22}N_3$ $(M+H)^+$: 316.1808; found: 316.1810.

(4b) (*E*)-*N*-(5-Phenyl-1-(p-tolyl)pent-1-en-3-yl)pyrimidin-2-amine

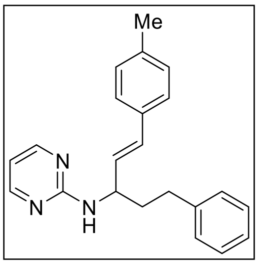

4b was synthesized according to the general procedure. Yield 30.4 mg (46%) of a colorless oil (hexane/EtOAc = 6/1).

^{1}H NMR (400 MHz, $CDCl_3$) δ 8.29 (d, *J* = 5.2 Hz, 2H), 7.30 – 7.26 (m, 3H), 7.26 – 7.23 (m, 2H), 7.19 (d, *J* = 6.9 Hz, 2H), 7.10 (d, *J* = 7.9 Hz, 2H), 6.57 (d, *J* = 8.2 Hz, 1H), 6.55 – 6.52 (m, 1H), 6.17 (dd, *J* = 15.9, 6.1 Hz, 1H), 5.43 (d, *J* = 8.6 Hz, 1H), 4.84 – 4.72 (m, 1H), 2.86 – 2.68 (m, 2H), 2.32 (s, 3H), 2.11 – 1.94 (m, 2H). **^{13}C NMR** (100 MHz, $CDCl_3$) δ 162.0, 158.0, 141.8, 137.2, 134.1, 129.9, 129.5, 129.2, 129.2, 128.4, 128.4, 126.3, 126.3, 125.9, 110.7, 52.4, 37.5, 32.4, 21.2. **HRMS (ESI):** Calc. for $C_{22}H_{24}N_3$ $(M+H)^+$: 330.1965; found: 330.1961.

(4c) (*E*)-*N*-(1-(4-Chlorophenyl)-5-phenylpent-1-en-3-yl)pyrimidin-2-amine

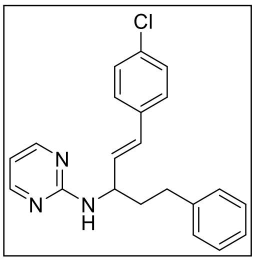

4c was synthesized according to the general procedure. Yield 35.7 mg (51%) of a yellowish oil (hexane/EtOAc = 6/1).

^{1}H NMR (400 MHz, $CDCl_3$) δ 8.29 (d, *J* = 4.8 Hz, 2H), 7.27 – 7.16 (m, 9H), 6.56 (d, *J* = 4.8 Hz, 1H), 6.21 (dd, *J* = 15.9, 5.9 Hz, 1H), 5.29 (d, *J* = 8.8 Hz, 1H), 4.81 – 4.74 (m, 1H), 2.78 (td, *J* = 9.6, 6.2 Hz, 2H), 2.09 – 1.97 (m, 2H). **^{13}C NMR** (100 MHz, $CDCl_3$) δ 161.9, 158.0, 158.0, 141.5, 135.4, 133.0, 131.3, 128.7, 128.6, 128.6, 128.4, 128.4, 127.6, 127.5, 125.9, 110.9, 52.3, 37.3, 32.3. **HRMS (ESI):** Calc. for $C_{21}H_{21}ClN_3$ $(M+H)^+$: 350.1419; found: 350.1417.

(4d) *N*-(1-Phenyl-5-(4-(*iso*-propenyl)phenyl)pent-1-en-3-yl)pyrimidin-2-amine

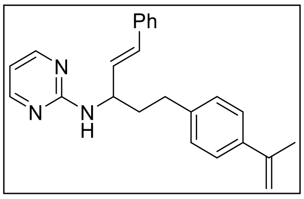

4d was synthesized according to the general procedure. Yield 38.5 mg (58%) of a colorless oil (hexane/EtOAc = 8/1).

^{1}H NMR (400 MHz, $CDCl_3$) δ 8.29 (d, *J* = 4.8 Hz, 2H), 7.43 – 7.31 (m, 5H), 7.30 (d, *J* = 7.4 Hz, 2H), 7.15 (d, *J* = 8.1 Hz, 2H), 6.64 – 6.54 (m, 1H), 6.55 (t, *J* = 4.8 Hz, 1H), 6.22 (dd, *J* = 15.9, 6.1 Hz, 1H), 5.34(s, 1H), 5.34 – 5.30 (m, 1H), 5.07 – 5.01 (m, 1H), 4.84 – 4.74 (m, 1H), 2.80 – 2.73 (m, 2H), 2.14 (s, 3H), 2.08 – 1.98 (m, 2H). **^{13}C NMR** (126 MHz, $CDCl_3$) δ 162.0, 158.0, 143.0, 140.9, 138.9, 136.9, 130.5, 130.0, 128.5, 128.3, 127.4, 126.4, 125.5, 111.7, 110.8, 52.3, 37.3, 31.9, 21.8. **HRMS (ESI):** Calc. for $C_{24}H_{26}N_3$ (M+H)$^+$: 356.2121; found: 356.2122.

(4e) 3-(1-Phenyl-3-(pyrimidin-2amino)-pent-1-en-5-yl)chalcone

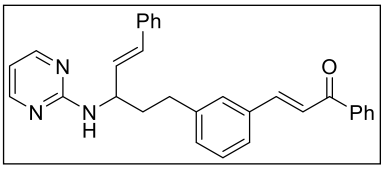

4e was synthesized according to the general procedure. Yield 44.7 mg (50%) of a light yellow oil. (hexane/EtOAc = 6/1).

^{1}H NMR (400 MHz, $CDCl_3$) δ 8.22 (d, *J* = 4.9 Hz, 2H), 7.94 (dt, *J* = 7.0, 1.4 Hz, 2H), 7.70 (d, *J* = 15.7 Hz, 1H), 7.55 – 7.47 (m, 1H), 7.48 – 7.36 (m, 5H), 7.32 – 7.24 (m, 3H), 7.27 – 7.19 (m, 2H), 7.20 – 7.10 (m, 2H), 6.53 (dd, *J* = 15.9, 1.4 Hz, 1H), 6.47 (t, *J* = 4.8 Hz, 1H), 6.16 (dd, *J* = 15.9, 6.1 Hz, 1H), 5.28 (d, *J* = 8.9 Hz, 1H), 4.80 – 4.68 (m, 1H), 2.82 – 2.68 (m, 2H), 2.06 – 1.93 (m, 2H). **^{13}C NMR** (126 MHz, $CDCl_3$) δ 190.5, 161.9, 158.1, 145.0, 142.4, 138.2, 136.8, 135.0, 132.7, 130.8, 130.4, 130.1, 129.0, 128.6, 128.5, 128.50, 128.46, 127.5, 126.4, 126.3, 122.0, 110.8, 52.1, 37.2, 32.1. **HRMS (ESI):** Calc. for $C_{30}H_{28}N_3O$ (M+H)$^+$: 446.2227; found: 446.2232.

4.2 Alkenylcarbonation products 5.

(5a) *N*-(2-(4-Methoxyphenyl)-5-phenylpent-4-en-1-yl)pyrimidin-2-amine

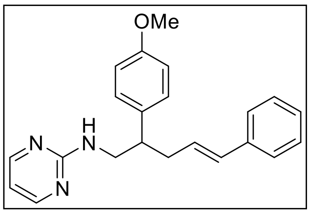

5a was synthesized according to the general procedure. Reaction temperature was at 90 °C. Yield 40.1 mg (58%) of a yellow liquid. (hexane/EtOAc = 4/1).

^{1}H NMR (400 MHz, $CDCl_3$) δ 8.23 (d, *J* = 4.9 Hz, 2H), 7.30 – 7.22 (m, 4H), 7.16 (d, *J* = 8.6 Hz, 2H), 6.86 (d, *J* = 8.7 Hz, 1H), 6.49 (t, *J* = 4.8 Hz, 1H), 6.38 (dt, *J* = 15.7, 1.4 Hz, 1H), 6.10 (ddd, *J* = 15.8, 7.6, 6.6 Hz, 1H), 5.07 (s, 1H), 3.93 – 3.82 (m, 1H), 3.79 (s, 3H), 3.55 – 3.40 (m, 1H), 3.08 – 2.92 (m, 1H), 2.68 – 2.47 (m, 2H). **^{13}C NMR** (100 MHz, $CDCl_3$) δ 162.2, 158.4, 157.9, 137.6, 134.1, 131.6, 128.8, 128.4, 128.0, 126.9, 126.0, 114.2, 110.5, 55.2, 46.4, 45.0, 37.8. **HRMS (ESI):** Calc. for $C_{21}H_{22}N_3$ (M+H)$^+$: 346.1914; found: 346.1923.

(5b) *N*-(2-(Naphthalen-2-yl)-5-phenylpent-4-en-1-yl)pyrimidin-2-amine

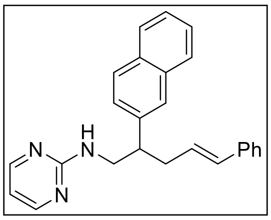

5b was synthesized according to the general procedure. Reaction temperature was at 90 °C. Yield 34.4 mg (47%) of a yellow liquid. (hexane/EtOAc = 6/1).

^{1}H NMR (500 MHz, $CDCl_3$) δ 8.23 (s, 2H), 7.86 – 7.68 (m, 4H), 7.51 – 7.36 (m, 3H), 7.27 – 7.12 (m, 4H), 6.56 – 6.47 (m, 1H), 6.43 (d, *J* = 15.7 Hz, 1H), 6.18 – 6.08 (m, 1H), 5.48 (s, 1H), 3.99 (dt, *J* = 13.0, 6.3 Hz, 1H), 3.73 – 3.62 (m, 1H), 3.25 (p, *J* = 7.2 Hz, 1H), 2.79 – 2.64 (m, 1H). **^{13}C NMR** (100 MHz, $CDCl_3$) δ 157.8, 139.6, 137.4, 133.6, 132.6, 131.8, 128.5, 128.4, 127.8,

127.7, 127.6, 127.0, 126.8, 126.1, 126.0, 125.8, 125.6, 110.0, 46.3, 46.1, 37.6. **HRMS (ESI):** Calc. for $C_{25}H_{24}N_3$ $(M+H)^+$: 366.1965; found: 366.1969.

(5c) *N*-(5-(4-Methoxyphenyl)-2-phenylpent-4-en-1-yl)pyrimidin-2-amine

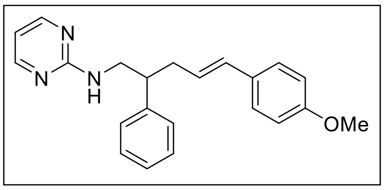

5c was synthesized according to the general procedure. Reaction temperature was at 90 °C. Yield 36.0 mg (52%) of a yellow liquid. (hexane/EtOAc = 4/1). **^{1}H NMR** (400 MHz, $CDCl_3$) δ 8.15 (d, *J* = 4.8 Hz, 2H), 7.24 (t, *J* = 7.5 Hz, 2H), 7.13 (dd, *J* = 17.3, 8.2 Hz, 5H), 6.72 (d, *J* = 8.3 Hz, 2H), 6.40 (t, *J* = 4.9 Hz, 1H), 6.25 (d, *J* = 15.8 Hz, 1H), 5.90 (dd, *J* = 15.3, 7.5 Hz, 1H), 5.01 (d, *J* = 7.6 Hz, 1H), 3.91 – 3.76 (m, 1H), 3.69 (s, 3H), 3.53 – 3.37 (m, 1H), 2.96 (t, *J* = 7.5 Hz, 1H), 2.51 (dt, *J* = 13.1, 7.2 Hz, 2H). **^{13}C NMR** (100 MHz, $CDCl_3$) δ 162.2, 158.8, 157.9, 142.3, 131.0, 130.4, 128.7, 127.9, 127.1, 126.8, 125.6, 113.8, 110.5, 55.2, 46.2, 46.0, 37.6. **HRMS (ESI):** Calc. for $C_{22}H_{24}N_3O$ $(M+H)^+$: 346.1914; found: 346.1925.

(5d) *N*-(2-Phenyl-5-(4-(trifluoromethyl)phenyl)pent-4-en-1-yl)pyrimidin-2-amine

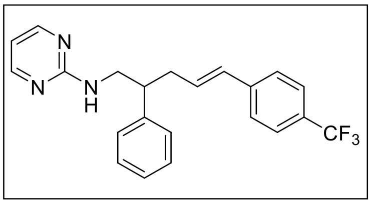

5d was synthesized according to the general procedure. Reaction temperature was at 90 °C. Yield 36.8 mg (48%) of a yellow oil. (hexane/EtOAc = 5/1). **^{1}H NMR** (400 MHz, $CDCl_3$) δ 8.20 (d, *J* = 4.9 Hz, 1H 2H), 7.44 (d, *J* = 8.1 Hz, 2H), 7.33 – 7.23 (m, 4H), 7.21 – 7.15 (m, 1H), 6.48 (t, *J* = 4.9 Hz, 1H), 6.36 (d, *J* = 15.8 Hz, 1H), 6.15 (dt, *J* = 15.3, 7.2 Hz, 1H), 5.41 (s, 1H), 3.86 (dt, *J* = 13.1, 6.4 Hz, 1H), 3.61 – 3.46 (m, 1H), 3.10 – 2.93 (m, 1H), 2.70 – 2.50 (m, 2H). **^{13}C NMR** (100 MHz, $CDCl_3$) δ 161.7, 157.8, 141.9, 140.9, 130.7, 130.5, 128.8, 127.8, 127.0, 126.1, 125.4 (q, 3.8 Hz), 125.3, 110.5, 46.3, 45.7, 37.5. **HRMS (ESI):** Calc. for $C_{22}H_{21}F_3N_3$ $(M+H)^+$: 284.1682; found: 184.1694.

(5e) (*E*)-*N*-(2-(naphthalen-2-yl)-5-(thiophen-2-yl)pent-4-en-1-yl)pyrimidin-2-amine

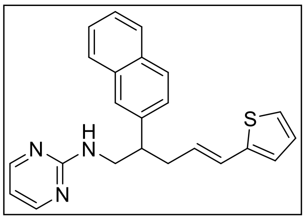

5e was synthesized according to the general procedure. Yield 33.5 mg (45%) of a yellow oil. (hexane/EtOAc = 6/1). **^{1}H NMR (400 MHz, $CDCl_3$)** δ 8.24 (d, *J* = 4.8 Hz, 2H), 7.81 (td, *J* = 8.6, 5.2 Hz, 3H), 7.68 (d, *J* = 1.7 Hz, 1H), 7.51 – 7.41 (m, 2H), 7.39 (dd, *J* = 8.5, 1.9 Hz, 1H), 7.05 (dt, *J* = 5.0, 0.9 Hz, 1H), 6.89 (dd, *J* = 5.1, 3.5 Hz, 1H), 6.81 (dt, *J* = 3.5, 0.8 Hz, 1H), 6.53 (ddd, *J* = 14.9, 1.5, 0.8 Hz, 1H), 6.50 (t, *J* = 4.8 Hz, 1H), 5.99 (dt, *J* = 15.5, 7.2 Hz, 1H), 5.01 – 4.92 (m, 1H), 4.03 – 3.92 (m, 1H), 3.70 – 3.58 (m, 1H), 3.27 – 3.18 (m, 1H), 2.73 – 2.61 (m, 2H). **^{13}C NMR** (100 MHz, $CDCl_3$) δ 162.3, 158.0, 142.6, 139.6, 133.6, 132.6, 128.5, 127.7, 127.6, 127.1, 126.7, 126.1, 125.8, 125.6, 125.0, 124.5, 123.4, 110.6, 46.2, 46.0, 37.5. **HRMS (ESI):** Calc. for $C_{23}H_{22}N_3S$ $(M+H)^+$: 372.1529; found: 372.1540.

(5f) *N*-(2-Phenylpent-4-en-1-yl)pyrimidin-2-amine

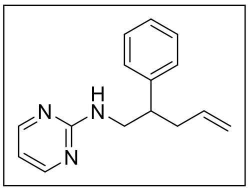

5h was synthesized according to the general procedure. Reaction temperature was at 90 °C. Two equivalent of vinyl bromide (1M solution in toluene) was used. Yield 23.1 mg (48%) of a yellow oil. (hexane/EtOAc = 6/1). **^{1}H NMR** (400 MHz, $CDCl_3$) δ 8.24 (d, *J* = 4.8 Hz, 2H), 7.39 – 7.13 (m, 5H), 6.49 (t, *J* = 4.8 Hz, 1H), 5.79 – 5.62 (m, 1H), 5.09 – 5.01 (m, 1H), 5.03 – 4.92 (m, 1H), 3.87 (ddd, *J* = 13.0, 7.2, 5.6 Hz, 1H), 3.48 (ddd, *J* = 13.4, 9.0, 4.6 Hz, 1H), 3.04 – 2.91 (m, 1H), 2.59 – 2.35 (m, 2H). **^{13}C NMR** (75

MHz, $CDCl_3$) δ 162.2, 157.9, 142.3, 136.1, 128.6, 127.9, 126.8, 116.5, 110.5, 46.4, 45.5, 38.3. **HRMS (ESI):** Calc. for $C_{15}H_{18}N_3$ $(M+H)^+$: 240.1495; found: 240.1496.

(5g) *N*-(5-Phenyl-2-((*E*)-styryl)pent-4-en-1-yl)pyrimidin-2-amine

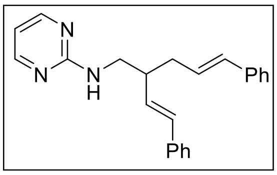

5g was synthesized according to the general procedure. Reaction temperature was at 90 °C. Yield 29.4 mg (43%) of a yellow oil. (hexane/EtOAc = 4.5/1)

^{1}H NMR (500 MHz, $CDCl_3$) δ 8.26 (d, *J* = 4.9 Hz, 2H), 7.51 – 6.96 (m, 11H), 6.51 (dd, *J* = 9.6, 5.2 Hz, 2H), 6.46 (d, *J* = 15.6 Hz, 1H), 6.26 (dd, *J* = 15.4, 7.7 Hz, 1H), 6.13 (dd, *J* = 15.9, 8.6 Hz, 1H), 5.24 (t, *J* = 5.8 Hz, 1H), 3.71 (dt, *J* = 12.4, 5.9 Hz, 1H), 3.47 – 3.31 (m, 1H), 2.68 (d, *J* = 7.4 Hz, 1H), 2.57 – 2.29 (m, 2H). **^{13}C NMR** (126 MHz, $CDCl_3$) δ 162.3, 158.0, 137.5, 137.1, 132.0, 131.9, 131.4, 128.5, 128.4, 127.6, 127.3, 127.0, 126.2, 126.1, 126.1, 110.6, 45.2, 43.6, 36.5. **HRMS (ESI):** Calc. for $C_{23}H_{24}N_3$ $(M+H)^+$: 342.1965; found: 342.1965.

4.3 Alkynylcarbonation product 6 and 7

(6a) *N*-(2-Benzyl-4-phenylbut-3-yn-1-yl)pyrimidin-2-amine

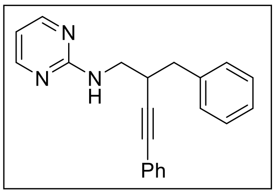

6a synthesized under the optimized conditions yield 53.3 mg (85%) of a white solid. (hexane/EtOAc = 5/1).

^{1}H NMR (400 MHz, $CDCl_3$) δ 8.21 (d, *J* = 4.8 Hz, 2H), 7.30 – 7.11 (m, 10H), 6.47 (t, *J* = 4.8 Hz, 1H), 5.57 (d, *J* = 6.8 Hz, 1H), 3.67 (ddd, *J* = 12.7, 6.8, 5.6 Hz, 1H), 3.47 (ddd, *J* = 13.1, 7.8, 5.6 Hz, 1H), 3.15 (tt, *J* = 8.0, 5.6 Hz, 1H), 2.95 – 2.77 (m, 2H). **^{13}C NMR** (100 MHz, $CDCl_3$) δ 162.2, 158.0, 138.8, 131.6, 129.3, 128.2, 128.1, 127.8, 126.4, 123.3, 110.8, 90.3, 84.0, 44.9, 38.8, 34.8. **HRMS (ESI):** Calc. for $C_{21}H_{20}N_3$ $(M+H)^+$: 314.1652; found: 314.1655.

Single crystal of **6a** (Table S1) was obtained by slow recrystallization from *n*-hexane/CH_2Cl_2. The measurement used a four circles goniometer Kappa geometry, Bruker AXS D8 Venture, equipped with a Photon 100 CMOS active pixel sensor detector. A Copper monochromatized (λ = 1.54178 Å) X-Ray radiation was selected for the measurement. Frames were integrated with the Bruker SAINT software using a narrow-frame algorithm. The integration of the data using a monoclinic unit cell yielded a total of 13513 reflections to a maximum θ angle of 72.38° (0.81 Å resolution), of which 3421 were independent (average redundancy 3.950, completeness = 99.2%, R_{int} = 5.14%, R_{sig} = 4.00%) and 2819 (82.40%) were greater than 2σ (F^2). Data were corrected for absorption effects using a multi-scan method with the use of the program (SADABS). The calculated minimum and maximum transmission coefficients (based on crystal size) are 0.7850 and 0.9680. The molecule was solved in the centrosymmetric space group C 1 2/c 1 with the aid of the software SHELXT , using a Dual Space method to solve the phase problem expanded in a triclinic unit cell P1.

**Table S2.** Crystallographic data of **6a.**			
Crystal size	(0.055 x 0.098 x 0.430) mm^3		
Crystal system	monoclinic		
Unit cell dimensions	a = 35.5176(9) Å	α = 90°	
	b = 4.65940(10) Å	β = 116.0890 (10)°	
	c = 23.3989(6) Å	γ = 90°	

Space group	C 1 2/c 1
Volume	3477.76(15) Å^3
Z	8
Density (calculated)	1.230 g/cm^3
Absorption coefficient	0.592 mm^{-1}
F(000)	1364

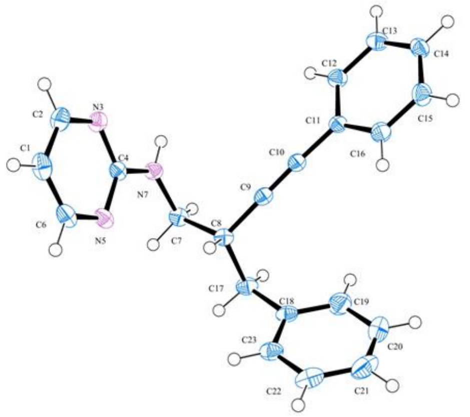

Figure S1. X-ray diffraction structure of compound **6a**.

(6b) *N*-(2-(4-Bromobenzyl)-4-phenylbut-3-yn-1-yl)pyrimidin-2-amine

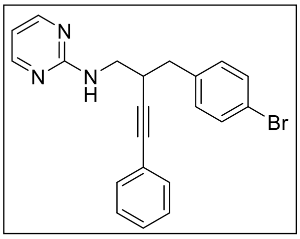

6b was synthesized according to the general procedure. Yield 45.7 mg (58%) of a yellow wax. (hexane/EtOAc = 5/1).

^{1}H NMR (500 MHz, $CDCl_3$) δ 8.29 (d, *J* = 4.7 Hz, 2H), 7.42 (d, *J* = 8.3 Hz, 2H), 7.36 – 7.30 (m, 2H), 7.31 – 7.25 (m, 3H), 7.19 (d, *J* = 8.4 Hz, 2H), 6.56 (t, *J* = 4.8 Hz, 1H), 5.48 (t, *J* = 6.1 Hz, 1H), 3.73 (ddd, *J* = 12.7, 6.8, 5.5 Hz, 1H), 3.53 (ddd, *J* = 13.1, 7.6, 5.5 Hz, 1H), 3.19 (ddd, *J* = 8.1, 5.5, 2.6 Hz, 1H), 2.94 (dd, *J* = 13.5, 5.5 Hz, 1H), 2.83 (dd, *J* = 13.5, 8.7 Hz, 1H). ^{13}C NMR (126 MHz, $CDCl_3$) δ 162.3, 158.0, 137.8, 131.6, 131.3, 131.0, 128.2, 128.0, 123.1, 120.3, 110.9, 89.9, 84.2, 44.9, 38.1, 34.7. **HRMS (ESI):** Calc. for $C_{21}H_{19}BrN_3$ $(M+H)^+$: 392.0757 (^{79}Br) and 394.0737 (^{81}Br); found: 392.0756 (^{79}Br) and 394.0743 (^{81}Br).

(6c) *N*-(4-Phenyl-2-(4-(trifluoromethyl)benzyl)but-3-yn-1-yl)pyrimidin-2-amine

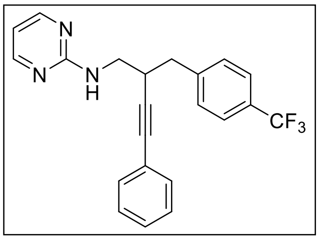

6c was synthesized according to the general procedure. Yield 27.5 mg (36%) of a yellow solid. (hexane/EtOAc = 5/1)

^{1}H NMR (500 MHz, $CDCl_3$) δ 8.29 (d, *J* = 4.9 Hz, 2H), 7.56 (d, *J* = 7.8 Hz, 2H), 7.43 (d, *J* = 7.7 Hz, 2H), 7.37 – 7.18 (m, 5H), 6.56 (t, *J* = 4.8 Hz, 1H), 5.62 (d, *J* = 6.3 Hz, 1H), 3.75 (dt, *J* = 12.8, 6.3 Hz, 1H), 3.57 (ddd, *J* = 13.2, 7.6, 5.6 Hz, 1H), 3.24 (td, *J* = 8.6, 4.1 Hz, 1H), 3.04 (dd, *J* = 13.4, 5.4 Hz, 1H), 2.92 (dd, *J* = 13.5, 8.7 Hz, 1H). **^{13}C NMR** (126 MHz, $CDCl_3$) δ 162.3, 158.1, 143.0, 131.6, 129.6, 128.2, 128.0, 123.0, 111.0, 89.6, 84.4, 125.11 (q, *J* = 3.8 Hz), 45.0, 38.5, 34.6. **HRMS (ESI):** Calc. for $C_{22}H_{19}F_3N_3$ $(M+H)^+$: 382.1526; found: 382.1533.

(6d) 1-(3-(4-Phenyl-2-((pyrimidin-2-ylamino)methyl)but-3-yn-1-yl)phenyl)ethan-1-one

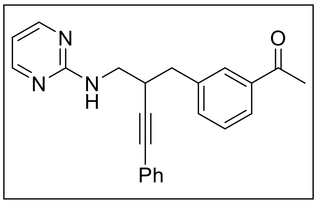

6d was synthesized according to the general procedure. Yield 48.9 mg (69%) of a yellow solid. (hexane/EtOAc = 4/1)

^{1}H NMR (400 MHz, $CDCl_3$) δ 8.32 (d, *J* = 4.8 Hz, 2H), 7.96 (d, *J* = 1.8 Hz, 1H), 7.86 (dt, *J* = 7.8, 1.5 Hz, 1H), 7.58 – 7.51 (m, 1H), 7.43 (t, *J* = 7.6 Hz, 1H), 7.39 – 7.32 (m, 2H), 7.31 – 7.25 (m, 4H), 6.58 (t, *J* = 4.8 Hz, 1H), 5.56 (t, *J* = 6.3 Hz, 1H),

3.79 (ddd, *J* = 12.7, 6.8, 5.6 Hz, 1H), 3.60 (ddd, *J* = 13.2, 7.5, 5.7 Hz, 1H), 3.27 (ddt, *J* = 8.7, 7.4, 5.5 Hz, 1H), 3.12 – 2.91 (m, 2H), 2.59 (s, 3H). **^{13}C NMR** (100 MHz, $CDCl_3$) δ 198.2, 162.3, 158.0, 139.4, 137.1, 134.1, 131.6, 129.2, 128.4, 128.2, 128.0, 126.6, 123.1, 110.9, 89.8, 84.4, 44.9, 38.5, 34.8, 26.6. **HRMS (ESI):** Calc. for $C_{23}H_{22}N_3O$ $(M+H)^+$: 356.1757; found: 356.1750.

(6e) *N*-(2-(3-Chlorobenzyl)-4-phenylbut-3-yn-1-yl)pyrimidin-2-amine

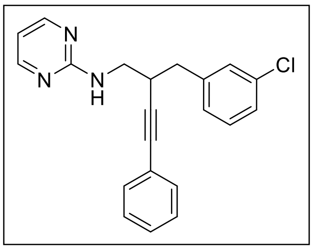

6e was synthesized according to the general procedure. Yield 33.4 mg (48%) of a yellow oil. (hexane/EtOAc = 5/1).

^{1}H NMR (500 MHz, $CDCl_3$) δ 8.32 (dd, *J* = 4.8, 1.5 Hz, 2H), 7.37 – 7.19 (m, 9H), 6.58 (t, *J* = 4.8 Hz, 1H), 5.73 (q, *J* = 7.2, 6.1 Hz, 1H), 3.82 – 3.70 (m, 1H), 3.55 – 3.61 (m, 1H), 3.24 (ddd, *J* = 8.1, 5.5, 2.6 Hz, 1H), 3.05 – 2.81 (m, 2H). **^{13}C NMR** (126 MHz, $CDCl_3$) δ 162.3, 158.0, 140.9, 133.9, 131.6, 129.5, 129.4, 128.2, 127.9, 127.5, 126.6, 123.1, 110.9, 89.8, 84.3, 44.9, 38.3, 34.7. **HRMS (ESI):** Calc. for $C_{21}H_{19}ClN_3$ $(M+H)^+$: 348.1262; found: 348.1267.

(6f) *N*-(5-Phenyl-2-(phenylethynyl)pent-4-en-1-yl)pyrimidin-2-amine

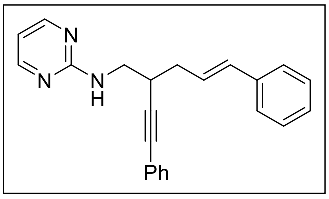

6f was synthesized according to the general procedure. Yield 51.8 mg (76%) of a yellow wax. (hexane/EtOAc = 4.5/1)

^{1}H NMR (400 MHz, $CDCl_3$) δ 8.20 (d, *J* = 4.8 Hz, 2H), 7.49 – 7.01 (m, 9H), 6.52 – 6.36 (m, 2H), 6.30 (dt, *J* = 15.7, 7.0 Hz, 1H), 5.65 (t, *J* = 6.3 Hz, 1H), 3.73 – 3.60 (m, 1H), 3.49 (ddd, *J* = 13.2, 7.7, 5.5 Hz, 1H), 3.03 (tt, *J* = 7.7, 5.7 Hz, 1H), 2.60 – 2.39 (m, 2H). **^{13}C NMR** (100 MHz, $CDCl_3$) δ 162.3, 158.0, 137.4, 132.3, 131.7, 128.5, 128.2, 127.8, 127.1, 126.9, 126.1, 123.3, 110.7, 90.3, 83.6, 44.8, 36.0, 33.0. **HRMS (ESI):** Calc. for $C_{23}H_{22}N_3$ $(M+H)^+$: 340.1808; found: 340.1815.

(6g) *N*-(5-(4-Methoxyphenyl)-2-(phenylethynyl)pent-4-en-1-yl)pyrimidin-2-amine

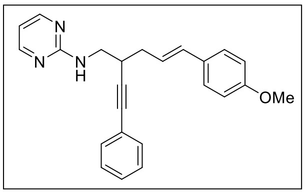

6g was synthesized according to the general procedure. Yield 51.6mg (70%) of a yellow wax. (hexane/EtOAc = 4/1)

^{1}H NMR (400 MHz, $CDCl_3$) δ 8.21 (d, *J* = 4.8 Hz, 2H), 7.36 – 7.28 (m, 2H), 7.27 – 7.15 (m, 5H), 6.81 – 6.73 (m, 2H), 6.46 (t, *J* = 4.8 Hz, 1H), 6.42 – 6.34 (m, 1H), 6.20 – 6.10 (m, 1H), 5.52 (t, *J* = 6.4 Hz, 1H), 3.72 (s, 3H), 3.67 (ddd, *J* = 12.8, 6.8, 5.7 Hz, 1H), 3.49 (ddd, *J* = 13.2, 7.8, 5.5 Hz, 1H), 3.02 (ddd, *J* = 7.7, 5.8, 1.9 Hz, 1H), 2.54 – 2.38 (m, 2H). ^{13}C NMR (100 MHz, $CDCl_3$) δ 162.3, 158.9, 158.0, 131.7, 130.3, 128.2, 127.8, 127.3, 124.7, 123.4, 113.9, 110.8, 90.4, 83.5, 55.3, 44.8, 36.0, 33.1. **HRMS (ESI):** Calc. for $C_{24}H_{24}N_3O(M+H)^+$: 370.1914; found: 370.1914.

(6h) *N*-(4-(4-Ethylphenyl)-2-(4-(trifluoromethyl)benzyl)but-3-yn-1-yl)pyrimidin-2-amine

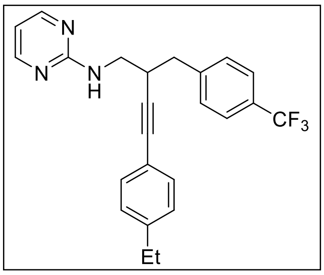

6h was synthesized according to the general procedure. Yield 45.9 mg (56%) of a yellow oil. (hexane/EtOAc = 5/1)

^{1}H NMR (500 MHz, $CDCl_3$) δ 8.31 (dd, *J* = 4.5, 1.9 Hz, 2H), 7.58 (d, *J* = 7.9 Hz, 2H), 7.45 (d, *J* = 7.9 Hz, 2H), 7.27 (dt, *J* = 8.1, 2.2 Hz, 2H), 7.14 (m, 2H), 6.58 (dp, *J* = 4.7, 2.4, 1.9 Hz, 1H), 5.74 (q, *J* = 5.9, 5.4 Hz, 1H), 3.77 (dt, *J* = 12.7, 6.2 Hz, 1H), 3.59 (ddd, *J* = 13.2, 7.4, 5.6 Hz, 1H), 3.26 (m, 1H), 2.99 (m, 2H), 2.65 (q, *J* =

7.6 Hz, 2H), 1.24 (m, 3H). **^{13}C NMR** (126 MHz, $CDCl_3$) δ 162.2, 158.0, 144.4, 143.1, 131.5, 129.6, 127.8, 125.1 (q, *J* = 3.7 Hz). 120.2, 110.9, 88.8, 84.5, 45.0, 38.5, 34.6, 28.7, 15.3. ^{19}F NMR (377 MHz, $CDCl_3$) δ -62.3. **HRMS (ESI):** Calc. for $C_{24}H_{23}F_3N_3$ $(M+H)^+$: 410.1839; found: 410.1843.

(7) *N*-(2-Benzyl-4-phenylbut-3-yn-1-yl)benzamide

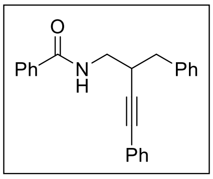

7 was synthesized according to the general procedure. Yield 38.1mg (56%) of a yellow oil. (hexane/EtOAc = 5/1)

^{1}H NMR (400 MHz, $CDCl_3$) δ 7.71 – 7.63 (m, 2H), 7.46 – 7.13 (m, 15H), 6.42 (s, 1H), 3.75 (ddd, *J* = 13.1, 6.6, 5.2 Hz, 1H), 3.42 (ddd, *J* = 13.2, 7.9, 5.4 Hz, 1H), 3.15 (tdd, *J* = 7.9, 6.4, 5.3 Hz, 1H), 2.89 (dd, *J* = 7.1, 2.3 Hz, 2H). **^{13}C NMR** (100 MHz, $CDCl_3$) δ 167.4, 138.5, 134.5, 131.6, 131.5, 129.3, 128.6, 128.4, 128.3, 128.1, 126.9, 126.6, 123.1, 89.9, 84.2, 43.2, 39.1, 35.0. **HRMS (ESI):** Calc. for $C_{24}H_{22}NO(M+H)^+$: 340.1696; found: 340.1704.

4.4 Functionalization products with alkyl halides 8 and 9

(8a) *N*-(1-Phenylbutyl)pyrimidin-2-amine

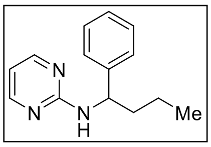

8a synthesized from methylarylation by general procedure 3.1: **1g** (27 μL, 0.2 mmol), $PhB(OH)_2$ (36.5 mg, 0.3 mmol), MeI (25 μL, 0.4 mmol). Yield 18.8 mg (41%) of a colorless oil (hexane/EtOAc = 10/1).

5a synthesized from hydroarylation by general procedure 3.2: **1k** (30 mg, 0.2 mmol), $PhB(OH)_2$ (36.5 mg, 0.3 mmol), EtBr (30 μL, 0.4 mmol). Yield 21.1 mg (46%) of a colorless oil (hexane/EtOAc = 10/1).

^{1}H NMR (400 MHz, $CDCl_3$) δ 8.13 (d, *J* = 4.8 Hz, 2H), 7.35 – 7.08 (m, 5H), 6.38 (t, *J* = 4.8 Hz, 1H), 5.73 (d, *J* = 8.4 Hz, 1H), 4.98 (q, *J* = 7.6 Hz, 1H), 1.73 (dq, *J* = 9.0, 7.1, 6.6 Hz, 2H), 1.47 – 1.15 (m, 2H), 0.84 (t, *J* = 7.4 Hz, 3H). **^{13}C NMR** (100 MHz, $CDCl_3$) δ 161.9, 157.9, 143.8, 128.4, 126.8, 126.3, 110.5, 54.8, 39.6, 19.5, 13.9. **HRMS (ESI):** Calc. for $C_{14}H_{18}N_3O$ $(M+H)^+$: 228.1495; found: 228.1499.

(8b) (*E*)-*N*-(1-Phenylhex-1-en-3-yl)pyrimidin-2-amine

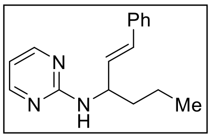

8b was synthesized from methylarylation by general procedure 3.1. Yield 18.5 mg (39%) of a colorless oil (hexane/EtOAc = 10/1).

^{1}H NMR (400 MHz, $CDCl_3$) δ 8.20 (d, *J* = 4.8 Hz, 2H), 7.27 (d, *J* = 7.9 Hz, 2H), 7.25 – 7.07 (m, 3H), 6.57 – 6.35 (m, 2H), 6.13 (ddd, *J* = 15.9, 5.9, 1.4 Hz, 1H), 5.17 (d, *J* = 8.8 Hz, 1H), 4.66 (p, *J* = 7.0 Hz, 1H), 1.61 (ddt, *J* = 11.1, 6.3, 4.5 Hz, 2H), 1.49 – 1.29 (m, 2H), 0.88 (t, *J* = 7.3 Hz, 3H). **^{13}C NMR** (100 MHz, $CDCl_3$) δ 162.0, 158.0, 137.0, 131.0, 129.5, 128.5, 127.3, 126.3, 110.6, 52.3, 37.9, 19.1, 14.0. **HRMS (ESI):** Calc. for $C_{16}H_{20}N_3$ $(M+H)^+$: 254.1652; found: 254.1653.

(9a) *N*-(1-Phenylpropyl)pyrimidin-2-amine

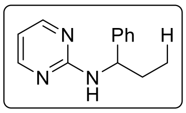

9a was synthesized according to the general procedure 3.2. Yield 36.4 mg (85%) of a white solid. (hexane/EtOAc = 8/1). **6** is a known compound (CAS: 1251300-40-9).

^{1}H NMR (400 MHz, $CDCl_3$) δ 8.15 (d, *J* = 4.8 Hz, 2H), 7.31 – 7.20 (m, 4H), 7.17 – 7.11 (m, 1H), 6.40 (t, *J* = 4.8 Hz, 1H), 5.47 (d, *J* = 8.2 Hz, 1H), 4.89 (q, *J* = 7.4 Hz, 1H), 1.80 (td, *J* = 7.3, 3.4 Hz, 2H), 0.87 (t, *J* = 7.4 Hz, 3H). **^{13}C NMR** (100 MHz, $CDCl_3$) δ 162.0, 157.9, 143.4, 128.4, 126.9, 126.4, 110.6, 56.5, 30.2, 10.7. **HRMS (ESI):** Calc. for $C_{13}H_{16}N_3$ $(M+H)^+$: 214.1339; found: 214.1346.

(9b) *N*-(1-(Naphthalen-2-yl)propyl)pyrimidin-2-amine

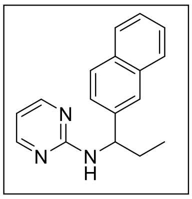

9b was synthesized according to the general procedure. Reaction temperature was 80 °C. Yield 30.1 mg (57%) of a colorless liquid (hexane/EtOAc = 6/1).

^{1}H NMR (400 MHz, $CDCl_3$) δ 8.23 (d, *J* = 4.8 Hz, 2H), 7.86 – 7.75 (m, 4H), 7.55 – 7.36 (m, 3H), 6.48 (s, 1H), 5.68 (d, *J* = 8.2 Hz, 1H), 5.13 (q, *J* = 7.4 Hz, 1H), 1.97 (t, *J* = 7.3 Hz, 2H), 0.99 (t, *J* = 7.4 Hz, 3H). **^{13}C NMR** (100 MHz, $CDCl_3$) δ 161.9, 158.0, 140.8, 133.4, 132.7, 128.2, 127.8, 127.6, 126.0, 125.5, 125.0, 124.9, 110.7, 56.7, 30.2, 10.8. **HRMS (ESI):** Calc. for $C_{17}H_{18}N_3$ $(M+H)^+$: 264.1459; found: 264.1502.

(9c) *N*-(1-(3,5-Bis(trifluoromethyl)phenyl)propyl)pyrimidin-2-amine

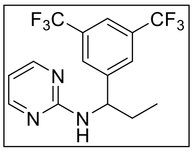

9c was synthesized according to the general procedure. Reaction temperature was 80 °C. Yield 38.4 mg (55%) of a white solid (hexane/EtOAc = 10/1).

^{1}H NMR (400 MHz, CDCl3) δ 8.24 (d, *J* = 4.8 Hz, 2H), 7.80 (d, *J* = 1.6 Hz, 2H), 7.74 (s, 1H), 6.56 (t, *J* = 4.8 Hz, 1H), 5.66 (d, *J* = 7.3 Hz, 1H), 5.03 (q, *J* = 7.2 Hz, 1H), 1.90 (p, *J* = 7.3 Hz, 2H), 1.00 (t, *J* = 7.4 Hz, 3H). **^{13}C NMR** (100 MHz, CDCl3) δ 161.5, 158.0, 146.6, 131.6 (q, *J* = 33.3 Hz), 126.7, 123.4 (q, *J* = 274.7 Hz), 124.7, 124.7, 121.0 – 120.8 (m), 111.5, 56.3, 30.2, 10.7. **HRMS (ESI):** Calc. for $C_{15}H_{14}F_6N_3$ $(M+H)^+$: 350.1086; found: 350.1098.

(9d) (*E*)-*N*-(1-phenylpent-1-en-3-yl)pyrimidin-2-amine

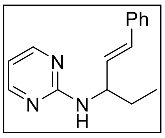

9d was synthesized according to the general procedure. Reaction temperature was 80 °C. Yield 29.2 mg (61%) of a colorless oil (hexane/EtOAc = 6/1).

^{1}H NMR (400 MHz, $CDCl_3$) δ 8.28 (d, *J* = 4.8 Hz, 2H), 7.35 (d, *J* = 7.1 Hz, 2H), 7.29 (d, *J* = 7.6 Hz, 2H), 7.24 – 7.16 (m, 1H), 6.58 (dd, *J* = 15.9, 1.4 Hz, 1H), 6.52 (t, *J* = 4.8 Hz, 1H), 6.21 (dd, *J* = 15.9, 5.9 Hz, 1H), 5.21 (d, *J* = 8.8 Hz, 1H), 4.67 (t, *J* = 7.3 Hz, 1H), 1.74 (dt, *J* = 14.1, 7.0 Hz, 2H), 1.02 (t, *J* = 7.4 Hz, 3H). **^{13}C NMR** (100 MHz, $CDCl_3$) δ 161.9, 158.0, 137.0, 130.6, 129.8, 128.5, 127.3, 126.3, 110.6, 53.9, 28.5, 10.3. **HRMS (ESI):** Calc. for $C_{15}H_{18}N_3$ $(M+H)^+$: 240.1495; found: 240.1094.

(9e) 2-(Dimethyl(1-phenylpropyl)silyl)pyrimidine

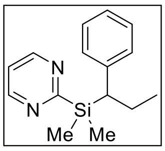

9e was synthesized according to the general procedure. Yield 53.9 mg (81%) of a colorless oil (hexane/EtOAc = 12/1).

^{1}H NMR (500 MHz, $CDCl_3$) δ 8.74 (d, *J* = 4.9 Hz, 2H), 7.23 – 7.14 (m, 3H), 7.07 (d, *J* = 7.4 Hz, 1H), 7.04 – 7.01 (m, 2H), 2.42 (dd, *J* = 11.3, 4.4 Hz, 1H), 1.90 – 1.79 (m, 2H), 0.80 (t, *J* = 7.2 Hz, 3H), 0.37 (s, 3H), 0.19 (s, 3H). **^{13}C NMR** (126 MHz, $CDCl_3$) δ 180.2, 154.9, 142.5, 128.0, 128.0, 124.5, 119.737.2, 22.6, 14.1, -4.5, -5.9. **HRMS (ESI):** Calc. for $C_{15}H_{21}N_2Si$ $(M+H)^+$: 257.1469; found: 257.1460.

4.5 Application of the method for pharmacophores 10.

(10a) *N*-(1,3-Diphenylpropyl)-4,6-dimethylpyrimidin-2-amine

10a was synthesized according to the general procedure from **1m** (33 mg, 0.2 mmol). Yield 35.0 mg (55%) of a colorless oil (hexane/EtOAc = 12/1).

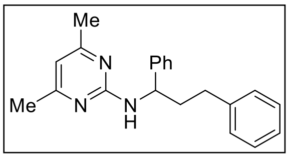

^{1}H NMR (500 MHz, $CDCl_3$) δ 7.49 – 7.12 (m, 10H), 6.30 (s, 1H), 5.52 (d, *J* = 8.6 Hz, 1H), 5.20 (td, *J* = 8.1, 6.3 Hz, 1H), 2.82 – 2.58 (m, 2H), 2.28 (s, 6H), 2.23 – 2.09 (m, 2H). **^{13}C NMR** (75 MHz, $CDCl_3$) δ 167.3, 161.8, 143.7, 141.9, 128.4, 128.4, 128.3, 126.9, 126.7, 125.8, 109.8, 54.6, 38.8, 32.7, 23.8. **HRMS (ESI):** Calc. for $C_{21}H_{24}N_3$ $(M+H)^+$: 318.1965; found: 318.1976.

(10b) *N*-Methyl-(1-phenyl-3-(4-(prop-1-en-2-yl)phenyl)propyl)pyrimidin-2-amine

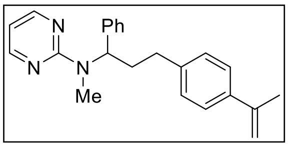

10b was synthesized according to the general procedure from **1l** (30 mg, 0.2 mmol), reaction temperature was 110 °C. Yield 30.3 mg (45%) of a colorless oil (hexane/EtOAc = 8/1).

^{1}H NMR (500 MHz, $CDCl_3$) δ 8.35 (d, *J* = 4.8 Hz, 2H), 7.41 – 7.36 (m, 2H), 7.36 – 7.28 (m, 4H), 7.25 (d, *J* = 6.7 Hz, 1H), 7.18 – 7.11 (m, 2H), 6.51 (t, *J* = 4.7 Hz, 1H), 6.30 (dd, *J* = 10.3, 5.4 Hz, 1H), 5.04 (q, *J* = 1.7 Hz, 1H), 2.91 (s, 3H), 2.66 (dd, *J* = 9.1, 6.9 Hz, 2H), 2.42 – 2.26 (m, 2H), 2.14 (s, 3H). **^{13}C NMR** (126 MHz, $CDCl_3$) δ 162.6, 157.6, 143.0, 141.2, 140.9, 138.9, 128.4, 128.2, 127.3, 127.1, 125.5, 111.7, 109.5, 56.1, 32.7, 32.5, 28.9, 21.8. **HRMS (ESI):** Calc. for $C_{23}H_{26}N_3O$ $(M+H)^+$: 344.2121; found: 344.2127.

(10c) *N*-(5-(2-Methoxyphenyl)-2-phenylpent-4-en-1-yl)-4,6-dimethylpyrimidin-2-amine

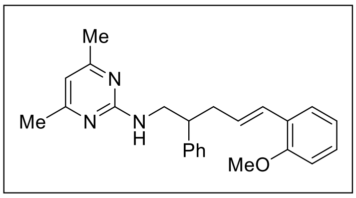

10c was synthesized according to the general procedure from **1m** (33 mg, 0.2 mmol), reaction temperature was 90 °C. Yield 38.1 mg (51%) of a colorless oil (hexane/EtOAc = 6/1).

^{1}H NMR (500 MHz, $CDCl_3$) δ 7.40 – 7.31 (m, 3H), 7.31 – 7.26 (m, 2H), 7.27 – 7.15 (m, 2H), 6.96 – 6.82 (m, 2H), 6.75 (d, *J* = 15.7 Hz, 1H), 6.30 (d, *J* = 7.1 Hz, 1H), 6.19 – 6.08 (m, 1H), 4.93 (d, *J* = 5.7 Hz, 1H), 4.02 – 3.91 (m, 1H), 3.83 (s, 3H), 3.67 – 3.55 (m, 1H), 3.13 – 3.01 (m, 1H), 2.79 – 2.56 (m, 2H), 2.28 (s, 6H). **^{13}C NMR** (126 MHz, $CDCl_3$) δ 167.3, 162.2, 156.3, 142.7, 128.9, 128.6, 127.9, 127.9, 126.6, 126.5, 126.1, 120.6, 110.8, 109.6, 55.4, 46.2, 46.24, 46.23, 38.1, 23.8. **HRMS (ESI):** Calc. for $C_{24}H_{28}N_3O$ $(M+H)^+$: 374.2227; found: 374.2230.

(10d) *N*-(1,3-Diphenylpropyl)-4,6-dimethylpyrimidin-2-amine

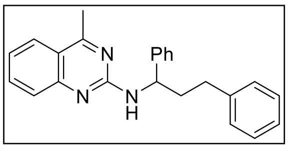

10d was synthesized according to the general procedure from **1n** (40.0 mg. 0.2 mmol), $PhB(OH)_2$ (48.8 mg, 0.4 mmol) and iodobenzene (43 µL, 0.4 mmol), K_3PO_4 (127 mg, 0.6 mmol). Yield 30.6 mg (43%) of a yellow wax (hexane/EtOAc = 10/1).

^{1}H NMR (400 MHz, $CDCl_3$) δ 7.87 – 7.81 (m, 1H), 7.67 – 7.55 (m, 2H), 7.50 – 7.42 (m, 2H), 7.39 – 7.20 (m, 8H), 5.60 (d, *J* = 8.5 Hz, 1H), 5.34 (q, *J* = 7.6 Hz, 1H), 2.88 – 2.63 (m, 2H), 2.77 (s, 3H), 2.40 – 2.19 (m, 2H). **^{13}C NMR** (126 MHz, $CDCl_3$) δ 169.5, 158.1, 151.6, 143.4, 141.7, 133.5, 128.4, 128.3, 128.3, 127.0, 126.7, 125.8, 125.2, 122.2, 119.6, 54.7, 38.7, 32.7, 21.5. **HRMS (ESI):** Calc. for $C_{24}H_{24}N_3$ $(M+H)^+$: 354.1965; found: 354.1957.

(10e) Methyl 4-(3-((4-methylquinazolin-2-yl)amino)-3-phenylpropyl)benzoate

10e was synthesized according to the general procedure from **1n** (40.0 mg. 0.2 mmol), $PhB(OH)_2$ (48.8 mg, 0.4 mmol) and methyl 4-iodobenzoate (104.8 mg, 0.4 mmol), K_3PO_4 (127 mg, 0.6 mmol). Yield 44.5 mg (54%) of a yellow wax (hexane/EtOAc = 5/1).

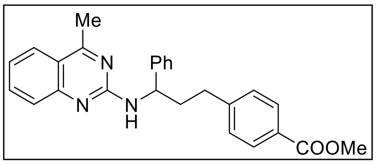

^{1}H NMR (500 MHz, $CDCl_3$) δ 7.94 (d, J = 8.3, 2H), 7.82 (dd, J = 8.2, 1.4 Hz, 1H), 7.67 – 7.51 (m, 2H), 7.47 – 7.38 (m, 2H), 7.37 – 7.29 (m, 2H), 7.29 – 7.16 (m, 4H), 5.72 (s, 1H), 5.37 – 5.22 (m, 1H), 3.90 (s, 1H), 2.76 (s, 3H), 2.87 – 2.70 (m, 2H), 2.38 – 2.26 (m, 1H), 2.26 – 2.15 (m, 1H). **^{13}C NMR** (126 MHz, $CDCl_3$) δ 167.1, 158.1, 151.6, 147.3, 143.1, 133.6, 129.7, 128.5, 128.5, 127.9, 127.1, 126.7, 125.2, 122.3, 119.7, 54.6, 51.9, 38.1, 32.7, 21.5. **HRMS (ESI):** Calc. for $C_{26}H_{26}N_3O_2$ $(M+H)^+$: 412.2020; found: 412.2022.

5. Mechanistic studies.

5.1 Radical trapping experiment.

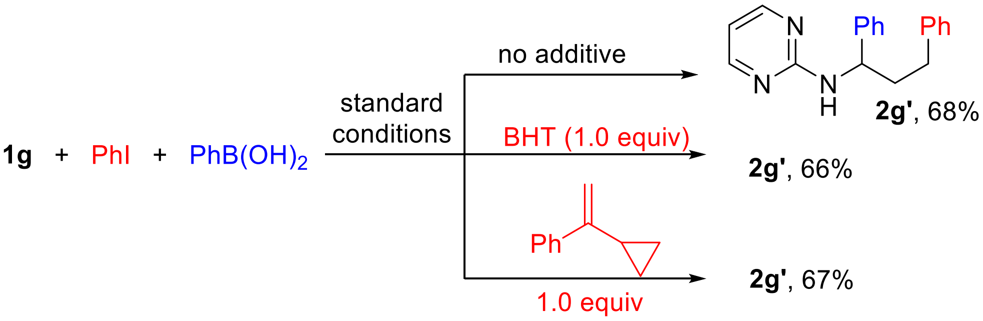

Two oven-dried 10 mL dram vials was loaded with dppm (8.5 mg, 0.022 mmol), $PhB(OH)_2$ (36.6 mg, 0.3 mmol), BHT (45.0 mg, 0.3 mmol) and (1-cyclopropylvinyl)benzene (30 μL, 0.3 mmol) respectively. Then the vials were taken into glovebox and charged with $Ni(COD)_2$ (5.5 mg, 0.02 mmol) and K_3PO_4 (85 mg, 0.4 mmol). After that, anhydrous dioxane (1.5 mL) was added. Then 1g (0.2 mmol, 26 μL) was added through a microsyringe. Finally, the vial was tightened and transferred into preheated oil bath at 100 °C and stirred for 18 h. After the reaction was completed, the mixture was diluted with water (1 mL) and extracted with EtOAc (2 mL × 3). To the combined organic phase was added 200 mg of silica gel and concentrated under reduced pressure. The desired product (2g') was purified by column chromatography (Hexane/EA = 8).

5.2 Deuterium-labelling experiments.

The reaction was carried out following the general procedure. ^{1}H NMR and ^{2}H NMR of **2g'** and ***d*₂-2g'** were compared in Figure S2. The ^{13}C NMR of **2g'** and ***d*₂-2g'** were showed in Figure S3.

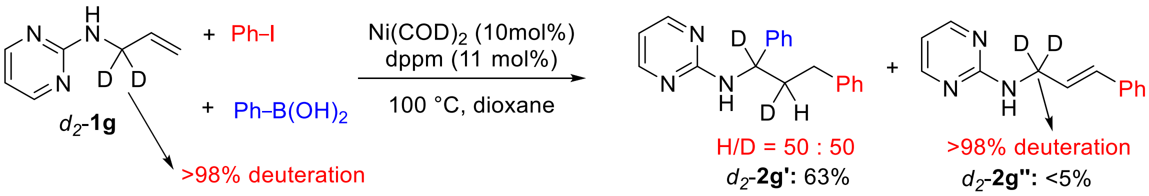

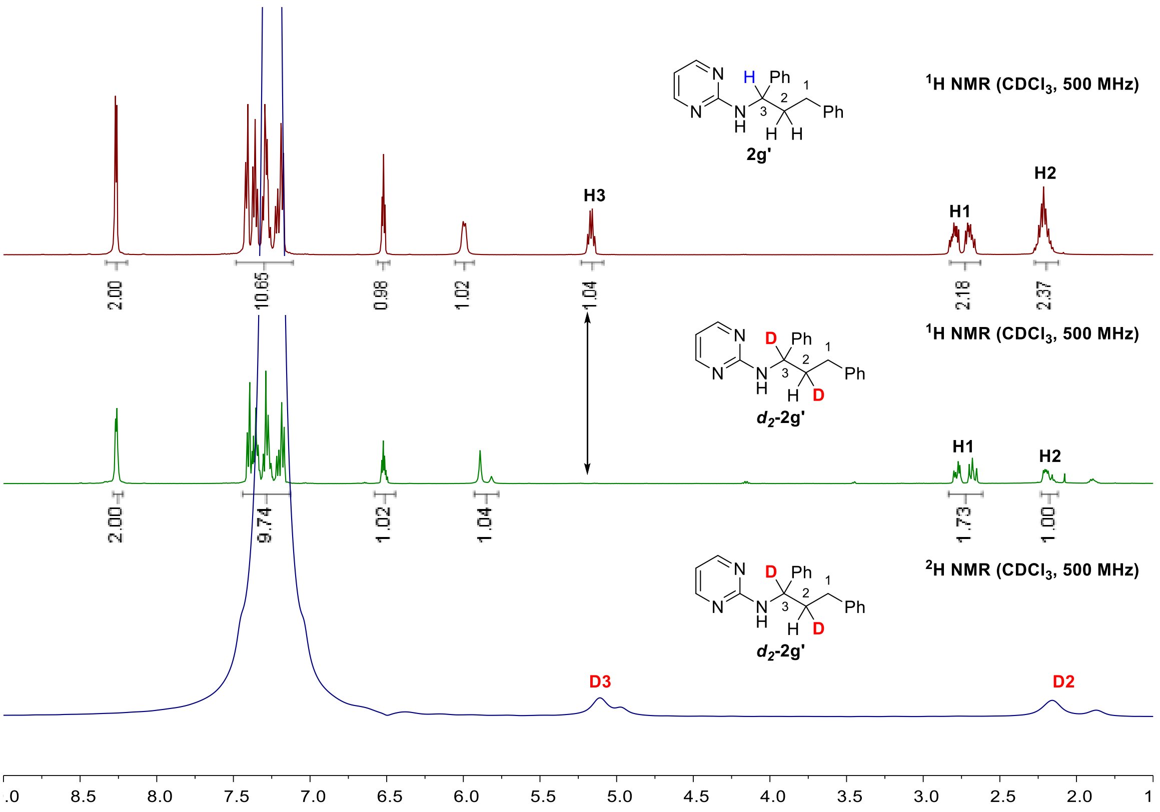

Figure S2. ^{1}H and ^{2}H NMR spectra of the deuterium labeled product *d*$_2$-2g'

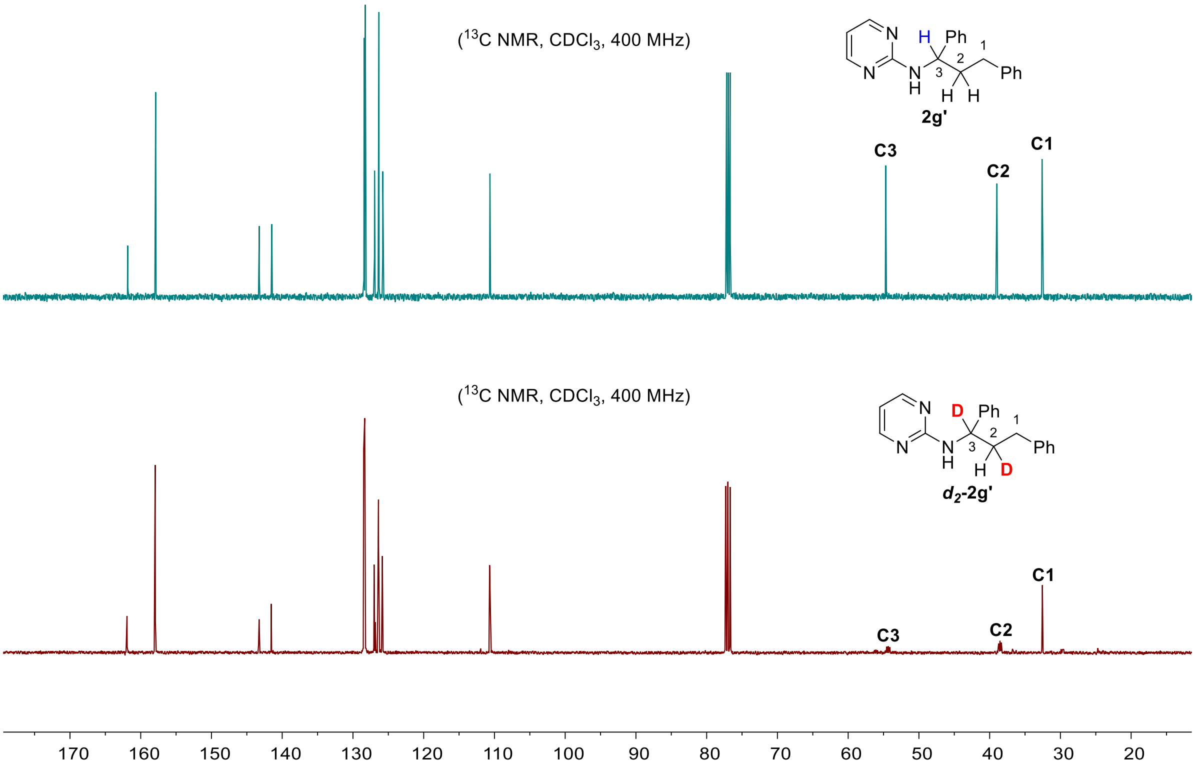

Figure S3. ^{13}C NMR spectra of the deuterium labeled product *d*$_2$-2g'

***N*-(1,3-diphenylpropyl-1,2-d_2)pyrimidin-2-amine (d_2-2g)**

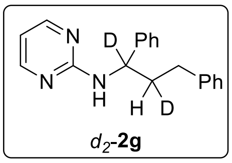

***d_2*-2g** was synthesized according to the general procedure from ***d_2*-1g** (27.5 mg. 0.2 mmol). Yield 73.8 mg (63%) of a colorless oil (hexane/EtOAc = 10/1).
^{1}H NMR (500 MHz, $CDCl_3$) δ 8.24 (d, *J* = 4.6 Hz, 2H), 7.42 – 7.10 (m, 10H), 6.49 (d, *J* = 1.3 Hz, 1H), 5.86 and 5.78 (s, 1H), 2.79 – 2.60 (m, 1.74H), 2.17 (dd, *J* = 10.5, 5.6 Hz, 1H). **^{13}C NMR** (100 MHz, $CDCl_3$) δ 161.8, 157.9, 143.2, 141.5, 128.5, 128.4, 128.3, 127.0, 126.4, 125.9, 110.7, 54.77 – 53.85 (m), 39.38 – 38.07 (m), 32.5. **GC/MS (EI)** (m/z, rel intensity): 79 (17), 91 (16), 185 (100), 186 (25), 291 (8). **HRMS (ESI):** Calc. for $C_{19}H_{18}D_2N_3$ $(M+H)^+$: 292.1777; found: 292.1786.

(*E*)-*N*-(3-phenylallyl-1,1-d_2)pyrimidin-2-amine (*d_2*-2g'')

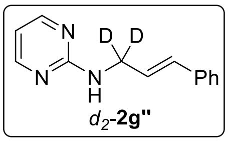

***d_2*-2g''** was isolated as a side product (*ca.* 4 mg, ≈5% yield).
^{1}H NMR (500 MHz, $CDCl_3$) δ 8.31 (d, *J* = 4.8 Hz, 2H), 7.37 (dd, *J* = 7.1, 1.4 Hz, 2H), 7.33 – 7.28 (m, 2H), 7.25 – 7.18 (m, 1H), 6.60 (d, *J* = 15.9 Hz, 1H), 6.56 (t, *J* = 4.8 Hz, 1H), 6.32 (d, *J* = 15.9 Hz, 1H), 5.41 (s, 1H). **^{13}C NMR** (126 MHz, $CDCl_3$) δ 162.0 (weak), 158.0, 136.8, 131.4, 128.5, 127.5, 126.4, 126.2, 110.8, 48.0 (weak), 45.0 (weak). **HRMS (ESI):** Calc. for $C_{13}H_{12}D_2N_3$ $(M+H)^+$: 214.1308; found: 214.1312.

6. Initial attempts on removing of the directing group.

In the recent literature, the following two procedures had been used to remove the *N*-aryl-*N*-pyrimidin-2-amines. However, when **2g** was subjected to condition **A**,[15] a mixture of 1,3-diphenylpropene (**S6**) and (1-chloropropane-1,3-diyl)dibenzene (**S7**) was formed. No desired product **S8** was detected from GC-MS. When **2g** was subjected to Condition **B**,[16] we found that it was reduced by Et_3SiH to 1,3-diphenylpropane (**S9**) in 75% isolated yield in the first step. The yield of **S29** was further improved to 86% when **2g** was heated in 48% HBr (aq.). Other conditions including oxidation, hydrogenation, substitution were also tried but proved to be futile.

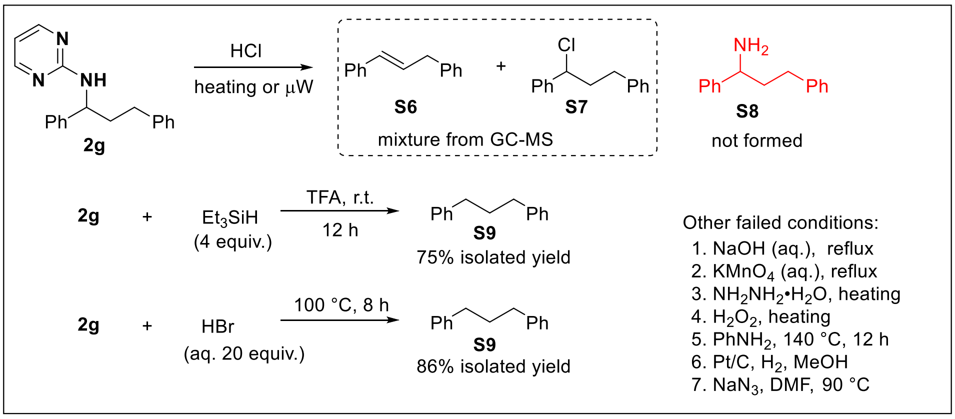

Procedure for the C-N bond cleavage in **2g** by hydrobromic acid: To a 10 mL dram vial was added **2g** (40 mg, 0.14 mmol), then 1 mL of 48% HBr (aq.) was added. The vial was capped and heated at 90 °C for 8 h. After cooling to room temperature, the mixture was neutralized by saturated aqueous $NaHCO_3$. Then extracted with EtOAc (2 mL × 3). The combined organic phase was dried over anhydrous Na_2SO_4. **S9** was obtained as colorless liquid (23.4 mg, 86%) by flash column chromatography (Hexane/EA = 50). **^{1}H NMR** (500 MHz, $CDCl_3$) δ 7.35 – 7.28 (m, 4H), 7.25 – 7.19 (m, 6H), 2.69 (t, *J* = 8.5, 4H), 2.05 – 1.95 (m, 2H). **^{13}C NMR** (126 MHz, $CDCl_3$) δ 142.3, 128.4, 128.3, 125.7, 35.4, 32.9. **S9** is a known compound (CAS: 1081-75-0) and its NMR data has been reported.[17]

7. References

1 J. Zheng, Z. Deng, Y. Zhang and S. Cui, *Adv. Synth. Catal.*, 2016, **358**, 746-751.

2 N. G. Moon and A. M. Harned, *Tetrahedron Lett.*, 2013, **54**, 2960-2963.

3 X. Frogneux, N. von Wolff, P. Thuéry, G. Lefèvre and T. Cantat, *Chem. Eur. J.*, 2016, **22**, 2930-2934.

4 H. Wang, Y. Wang, D. Liang, L. Liu, J. Zhang and Q. Zhu, *Angew. Chem. Int. Ed.*, 2011, **50**, 5678-5681.

5 S. Jaime-Figueroa, Y. Liu, J. M. Muchowski and D. G. Putman, *Tetrahedron Lett.*, 1998, **39**, 1313-1316.

6 D. S. Tsang, S. Yang, F.-A. Alphonse and A. K. Yudin, *Chem. Eur. J.*, 2008, **14**, 886-894.

7 S. Milanova and T. Konstantinova, *Rastenievud. Nauki*, 1995, **32**, 136-139.

8 A. V. Gulevskaya, B. U. W. Maes and C. Meyers, *Synlett*, 2007, **2007**, 71-74.

9 J. K. Elwood and J. W. Gates, Jr., *J. Org. Chem.*, 1967, **32**, 2956-2959.

10 A. V. Gulevich, F. S. Melkonyan, D. Sarkar and V. Gevorgyan, *J. Am. Chem. Soc.*, 2012, **134**, 5528-5531.

11 G. Vlád and I. T. Horváth, *J. Org. Chem.*, 2002, **67**, 6550-6552.

12 L. Xiao, A. Pöthig and L. Hintermann, *Monatsh. Chem.*, 2015, **146**, 1529-1539.

13 D. Kikelj, *Sci. Synth.*, 2004, **16**, 573-749.

14 X. Huang, H. Yang, H. Fu, R. Qiao and Y. Zhao, *Synthesis*, 2009, **2009**, 2679-2688.

15 Z. X. Ruan, S. Lackner and L. Ackermann, *Angew. Chem. Int. Ed.*, 2016, **55**, 3153-3157.

16 G. G. Pawar, A. Brahmanandan and M. Kapur, *Org. Lett.*, 2016, **18**, 448-451.

17 C.-T. Yang, Z.-Q. Zhang, Y.-C. Liu and L. Liu, *Angew. Chem., Int. Ed.*, 2011, **50**, 3904-3907.

8. NMR spectra of new compounds

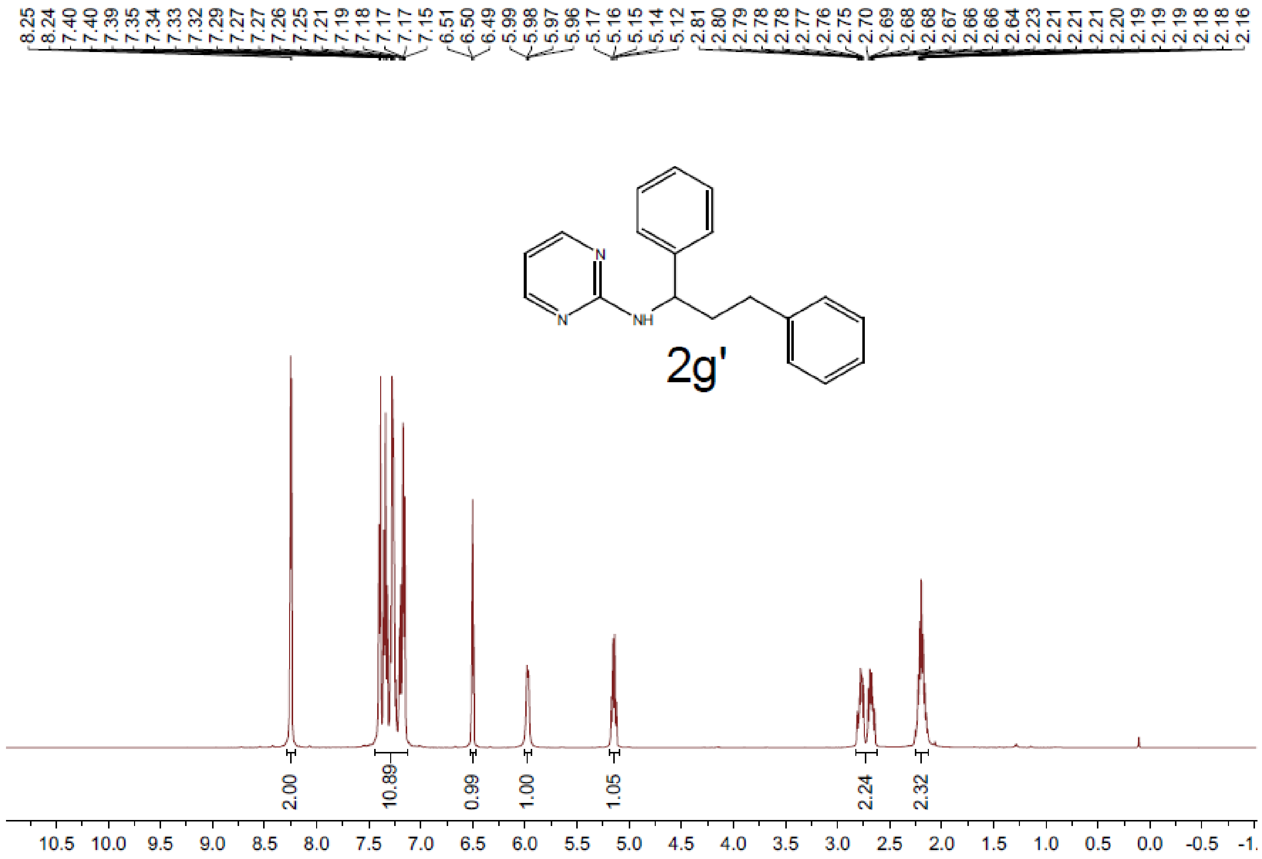
8.25
8.24
7.40
7.40
7.39
7.35
7.34
7.33
7.32
7.29
7.27
7.27
7.26
7.25
7.21
7.19
7.18
7.17
7.17
7.15
6.51
6.50
6.49
5.99
5.98
5.97
5.96
5.17
5.16
5.15
5.14
5.12
2.81
2.80
2.79
2.78
2.78
2.77
2.76
2.75
2.70
2.69
2.68
2.68
2.67
2.66
2.66
2.64
2.23
2.21
2.21
2.21
2.20
2.19
2.19
2.19
2.18
2.18
2.16
N
N
NH
2g'
2.00
10.89
0.99
1.00
1.05
2.24
2.32
10.5 10.0 9.5 9.0 8.5 8.0 7.5 7.0 6.5 6.0 5.5 5.0 4.5 4.0 3.5 3.0 2.5 2.0 1.5 1.0 0.5 0.0 -0.5

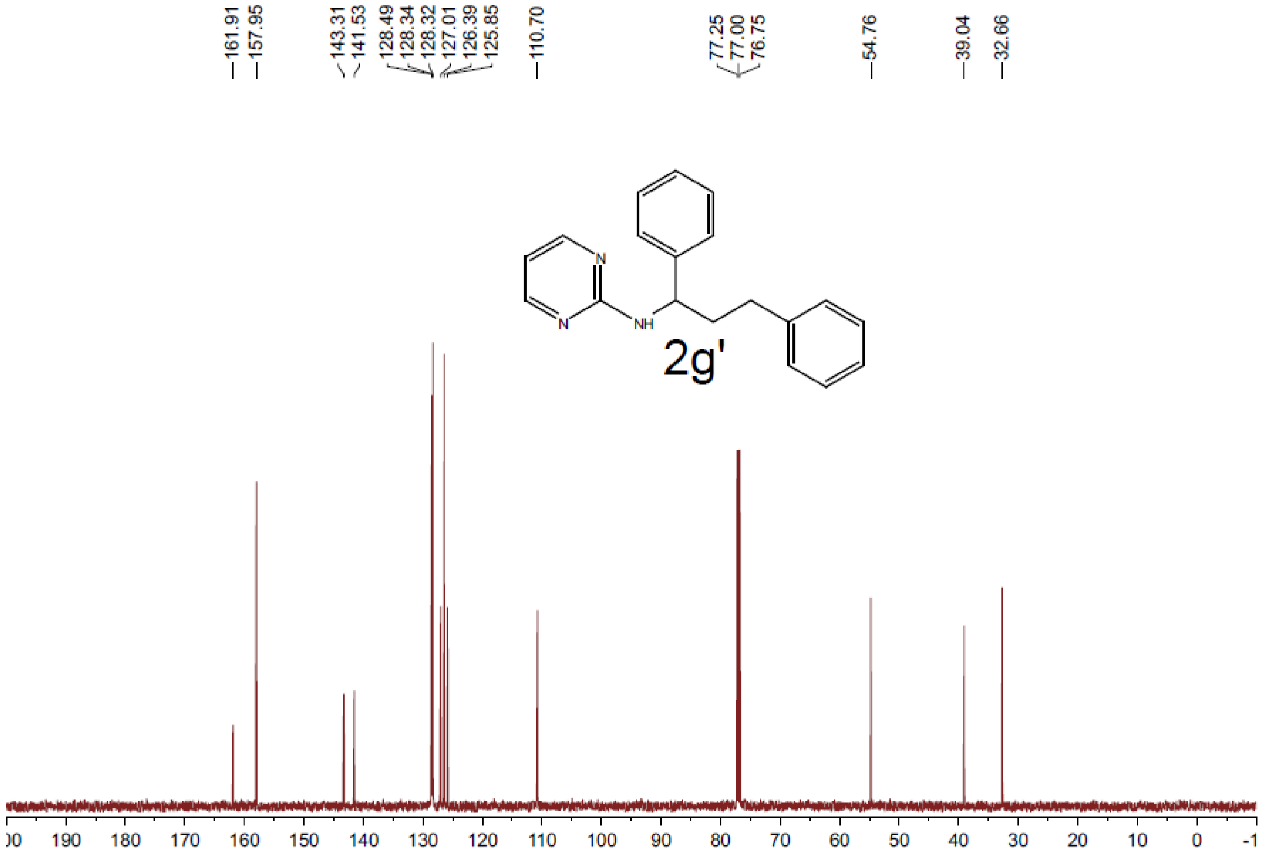
161.91
157.95
143.31
141.53
128.49
128.34
128.32
127.01
126.39
125.85
110.70
77.25
77.00
76.75
54.76
39.04
32.66
N
N
NH
2g'
190 180 170 160 150 140 130 120 110 100 90 80 70 60 50 40 30 20 10 0

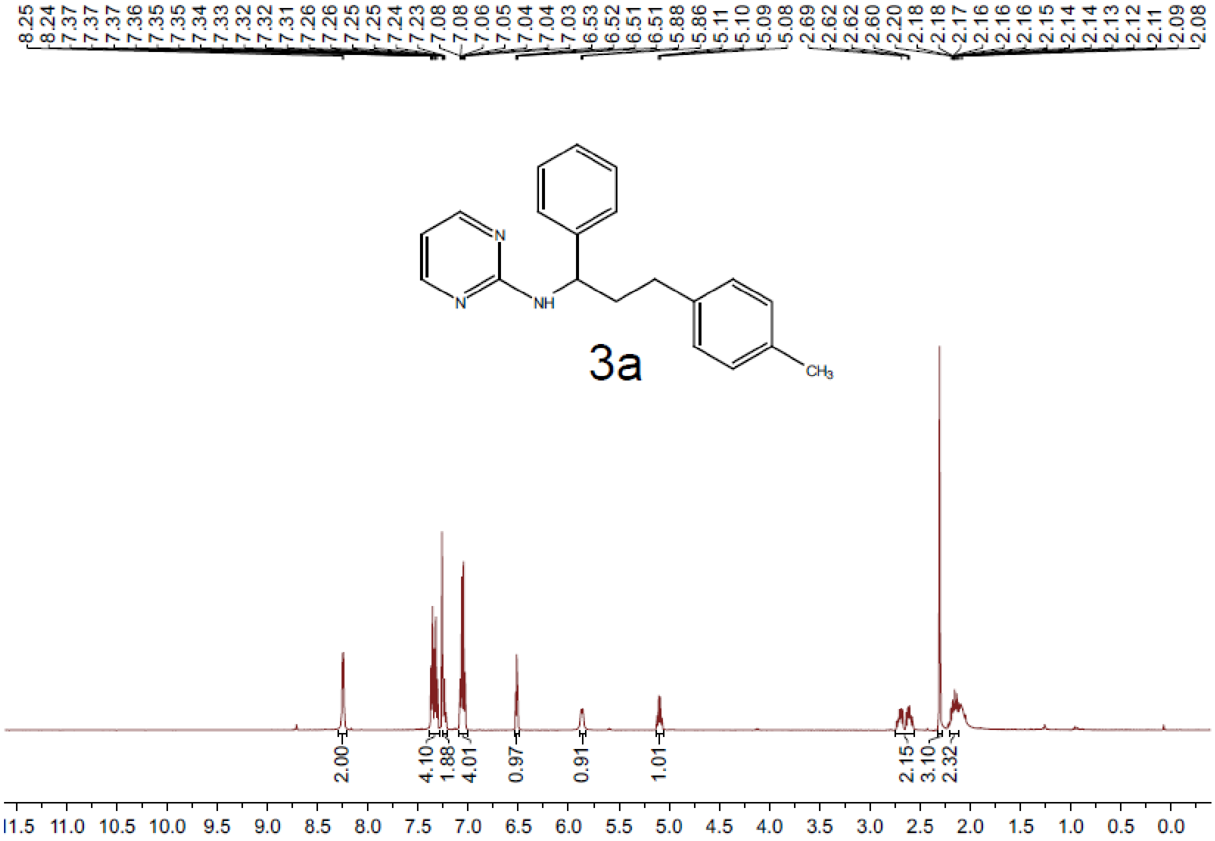
8.25
8.24
7.37
7.37
7.36
7.35
7.35
7.34
7.33
7.32
7.32
7.31
7.26
7.26
7.25
7.25
7.24
7.23
7.08
7.08
7.06
7.05
7.04
7.04
7.03
6.53
6.52
6.51
6.51
5.88
5.86
5.11
5.10
5.09
5.08
2.69
2.62
2.62
2.60
2.20
2.18
2.18
2.17
2.16
2.16
2.16
2.15
2.14
2.14
2.13
2.12
2.11
2.09
2.08
N
N
NH
CH_3
3a
2.00
4.10
1.88
4.01
0.97
0.91
1.01
2.15
3.10
2.32

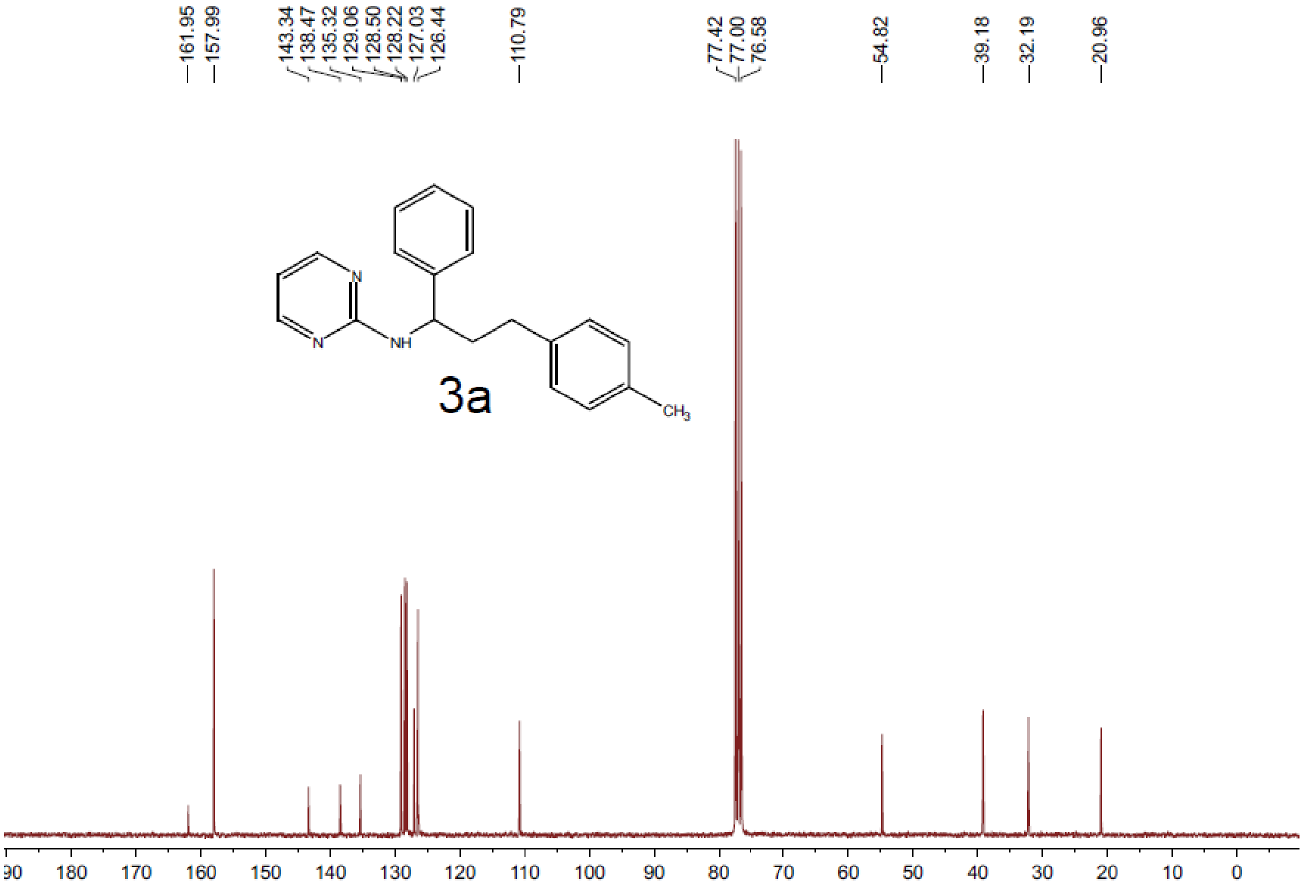
161.95
157.99
143.34
138.47
135.32
129.06
128.50
128.22
127.03
126.44
110.79
77.42
77.00
76.58
54.82
39.18
32.19
20.96
N
N
NH
CH_3
3a

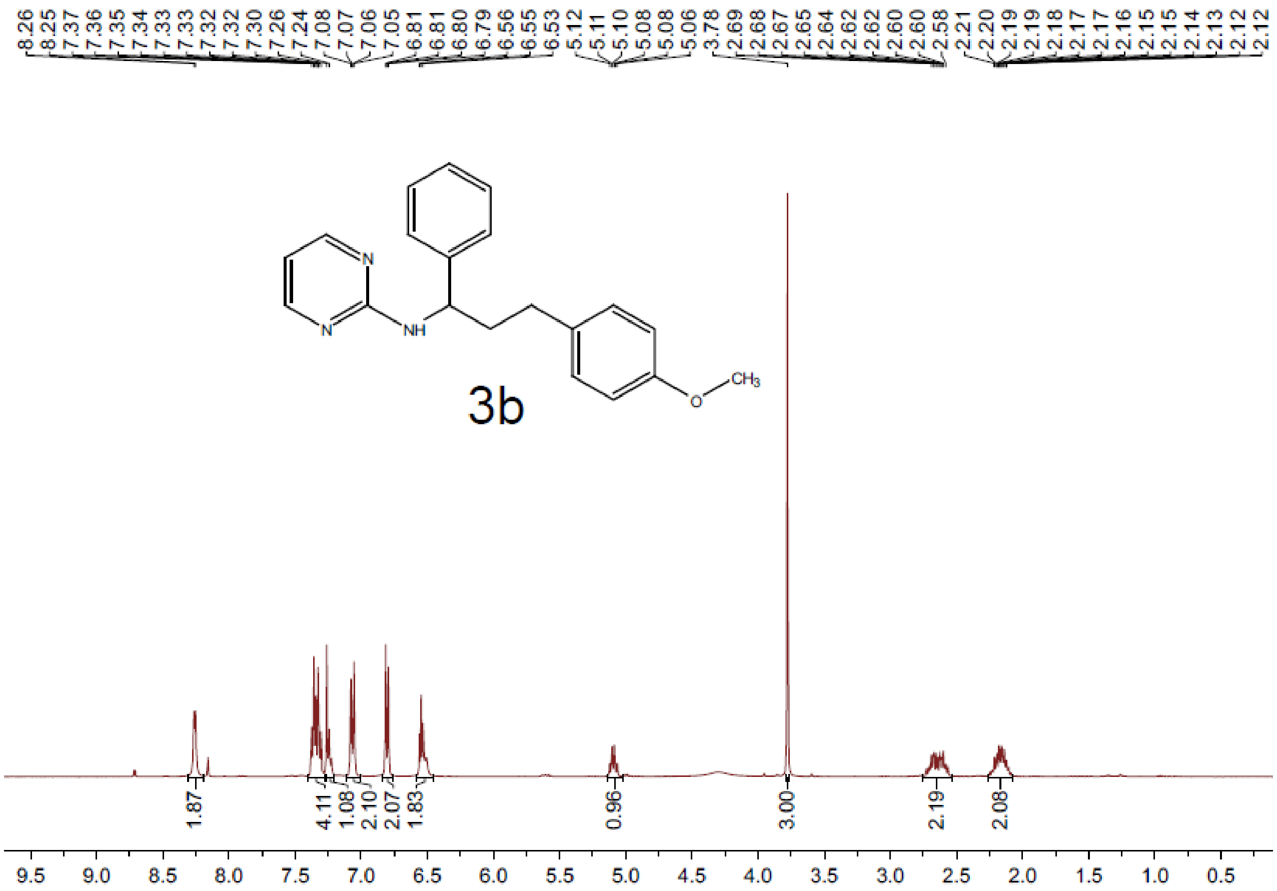
8.26
8.25
7.37
7.36
7.35
7.34
7.33
7.33
7.32
7.32
7.30
7.26
7.24
7.08
7.07
7.06
7.05
6.81
6.81
6.80
6.79
6.56
6.55
6.53
5.12
5.11
5.10
5.08
5.08
5.06
3.78
2.69
2.68
2.67
2.65
2.64
2.62
2.62
2.60
2.60
2.58
2.21
2.20
2.19
2.19
2.18
2.17
2.17
2.16
2.15
2.15
2.14
2.13
2.12
2.12
N
NH
CH3
O
3b
1.87
4.11
1.08
2.10
2.07
1.83
0.96
3.00
2.19
2.08
9.5
9.0
8.5
8.0
7.5
7.0
6.5
6.0
5.5
5.0
4.5
4.0
3.5
3.0
2.5
2.0
1.5
1.0
0.5

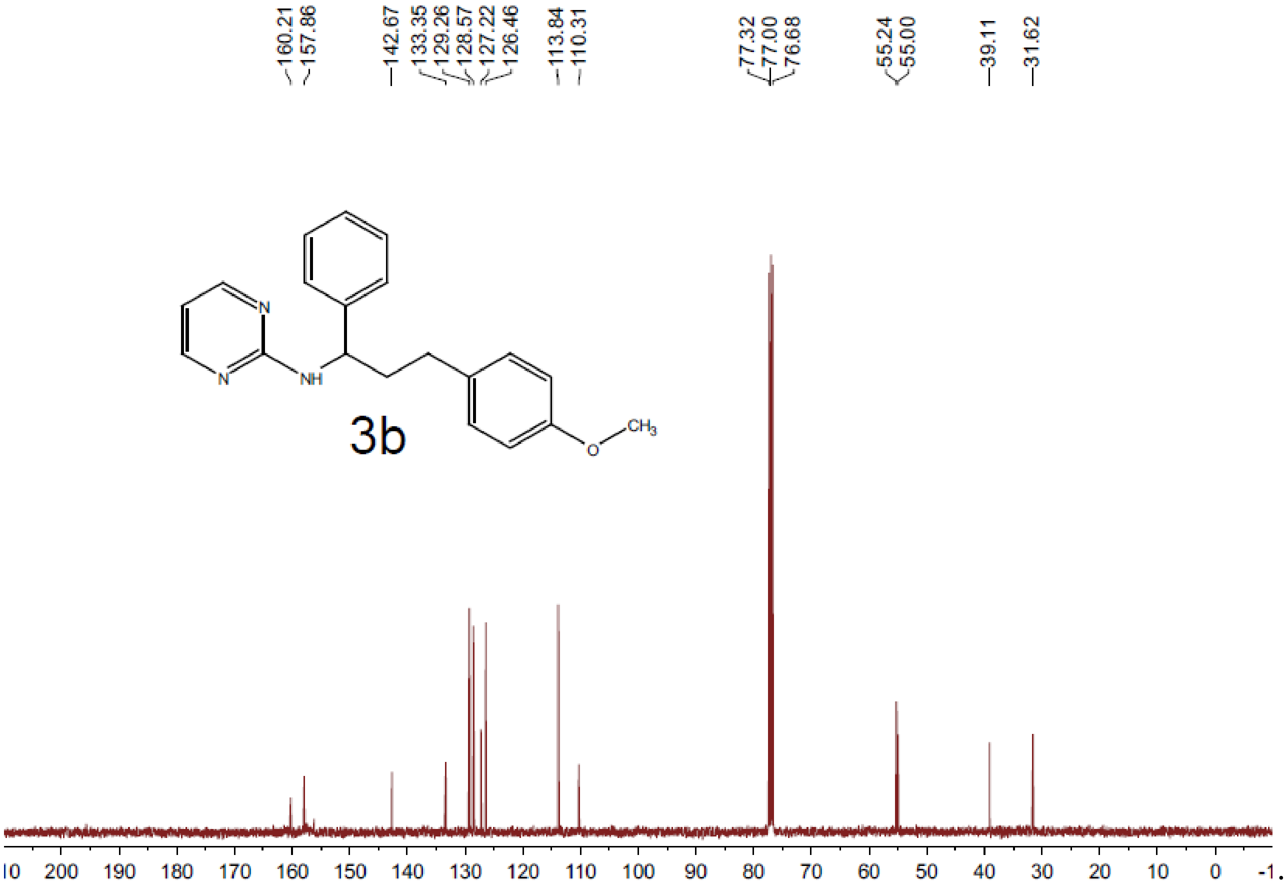
160.21
157.86
142.67
133.35
129.26
128.57
127.22
126.46
113.84
110.31
77.32
77.00
76.68
55.24
55.00
39.11
31.62
N
NH
CH3
O
3b
200
190
180
170
160
150
140
130
120
110
100
90
80
70
60
50
40
30
20
10
0

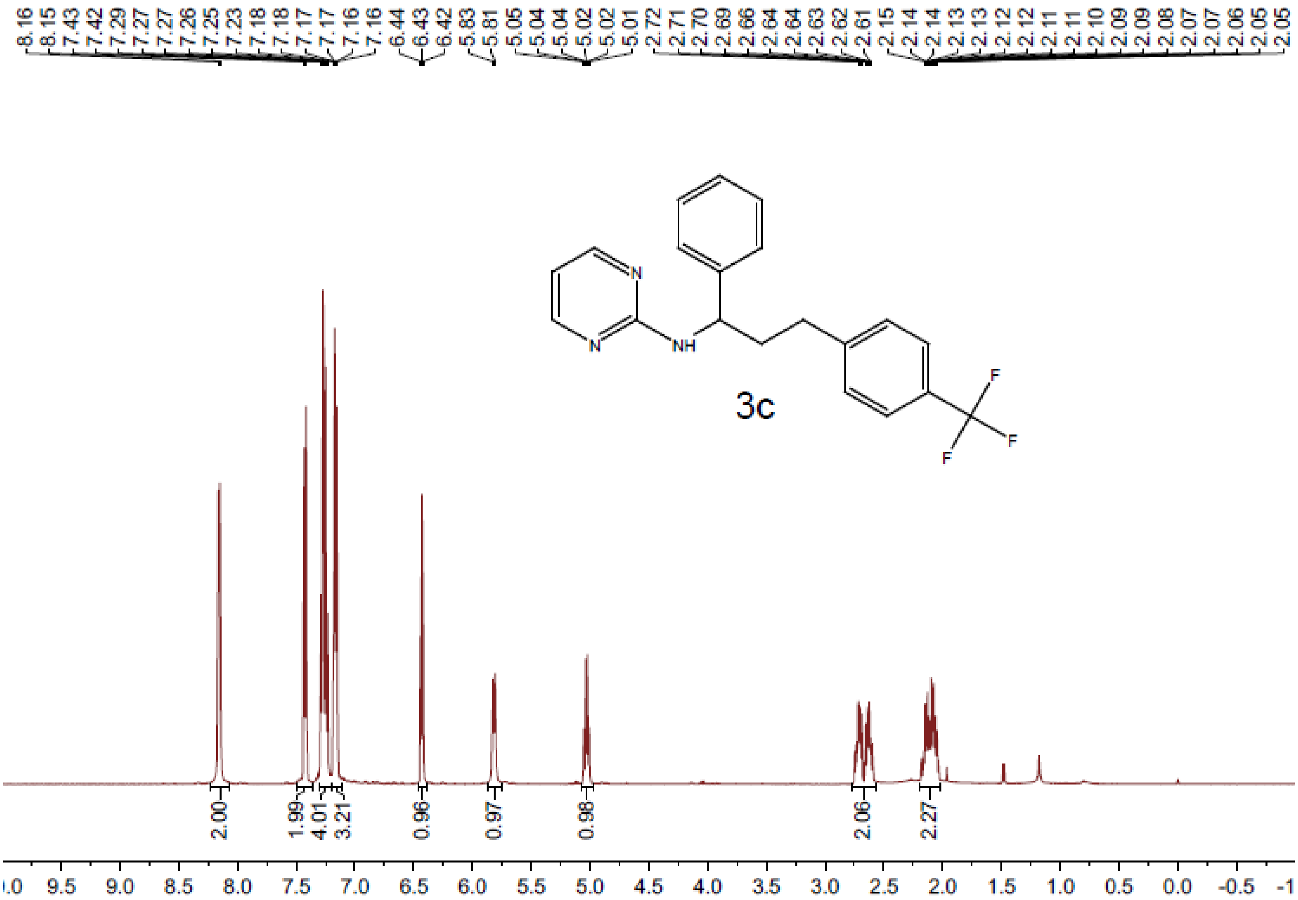
8.16 8.15 7.43 7.42 7.29 7.27 7.27 7.26 7.25 7.23 7.18 7.18 7.17 7.17 7.16 7.16 6.44 6.43 6.42 5.83 5.81 5.05 5.04 5.04 5.02 5.02 5.01 2.72 2.71 2.70 2.69 2.66 2.64 2.64 2.63 2.62 2.61 2.15 2.14 2.14 2.13 2.13 2.12 2.12 2.11 2.11 2.10 2.09 2.09 2.08 2.07 2.07 2.06 2.05 2.05
N
N
NH
F
F
F
3c
2.00
1.99
4.01
3.21
0.96
0.97
0.98
2.06
2.27
9.5 9.0 8.5 8.0 7.5 7.0 6.5 6.0 5.5 5.0 4.5 4.0 3.5 3.0 2.5 2.0 1.5 1.0 0.5 0.0 -0.5

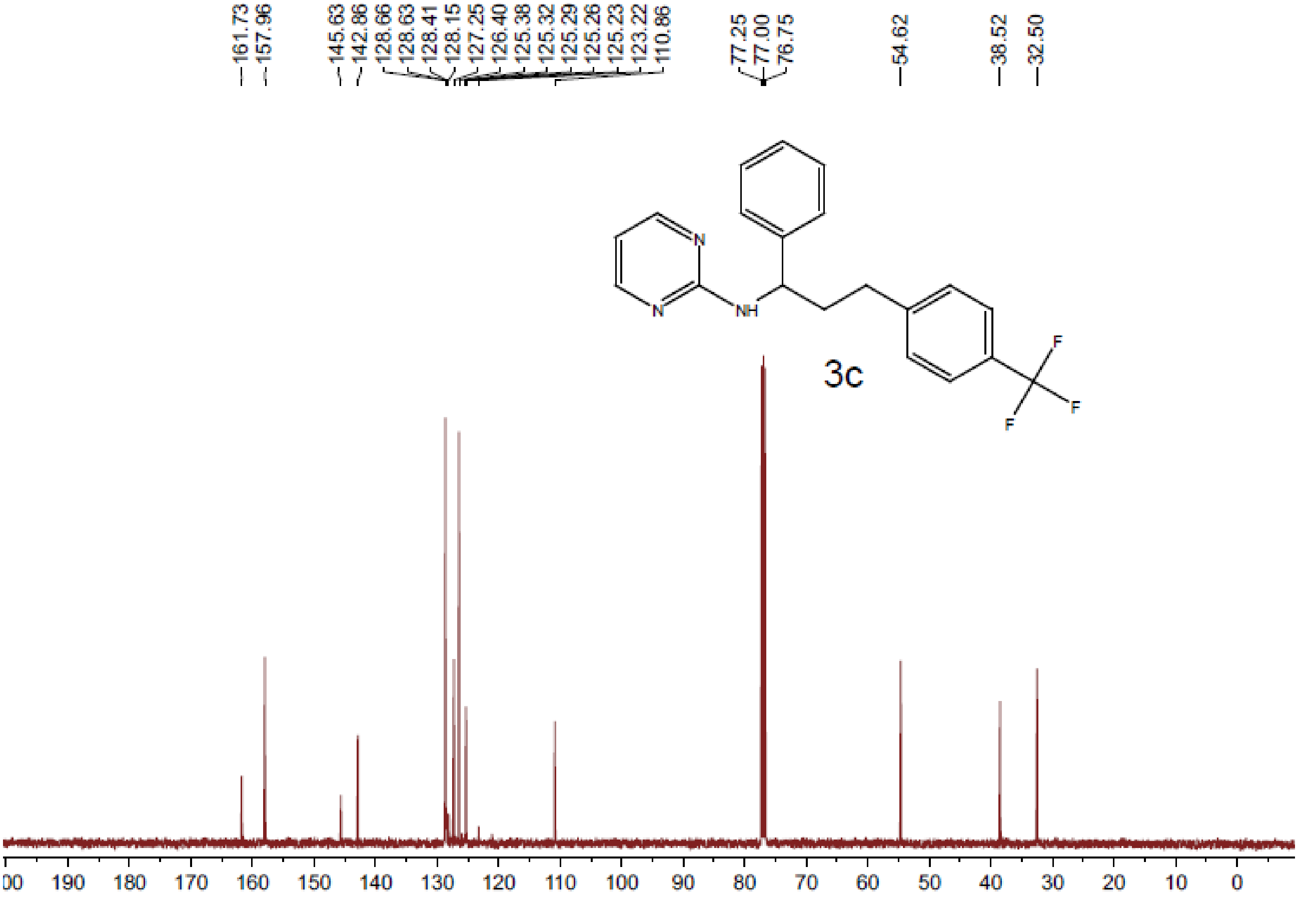
161.73 157.96 145.63 142.86 128.66 128.63 128.41 128.15 127.25 126.40 125.38 125.32 125.29 125.26 125.23 123.22 110.86 77.25 77.00 76.75 54.62 38.52 32.50
N
N
NH
F
F
F
3c
190 180 170 160 150 140 130 120 110 100 90 80 70 60 50 40 30 20 10 0

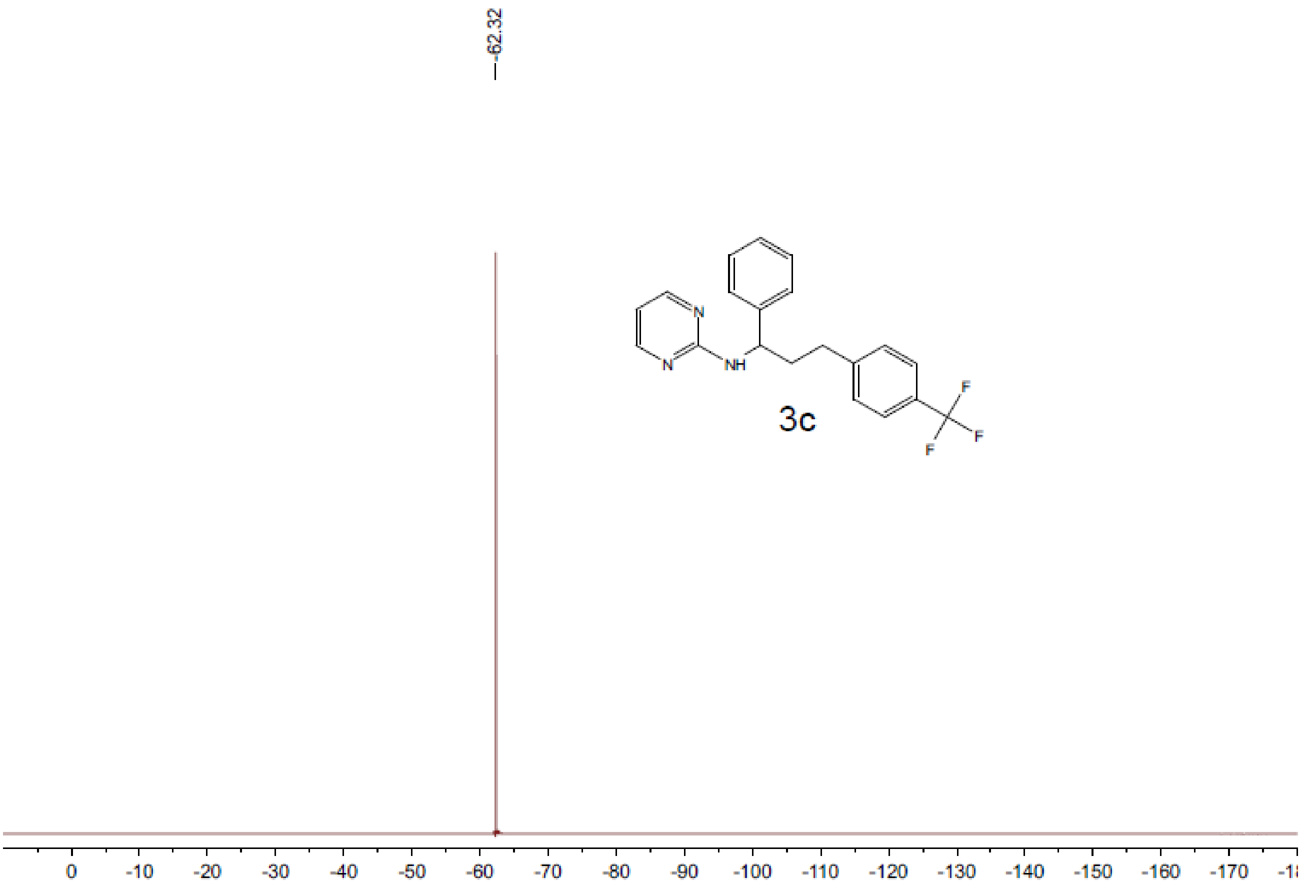
-62.32
N
N
NH
F
F
F
3c
0 -10 -20 -30 -40 -50 -60 -70 -80 -90 -100 -110 -120 -130 -140 -150 -160 -170

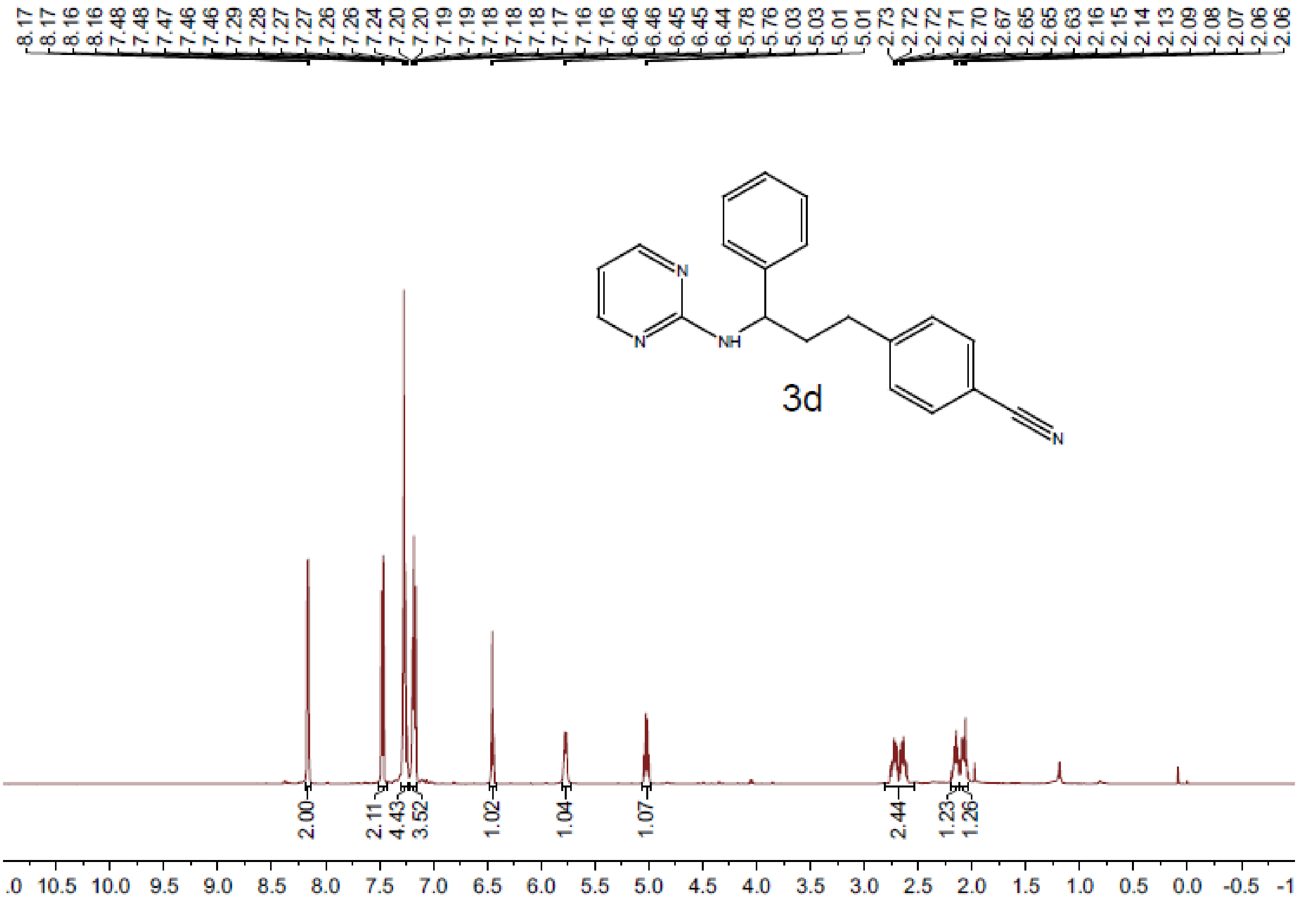
8.17 8.17 8.16 8.16 7.48 7.48 7.47 7.46 7.46 7.29 7.28 7.27 7.27 7.26 7.26 7.24 7.20 7.20 7.19 7.19 7.18 7.18 7.18 7.17 7.16 7.16 6.46 6.46 6.45 6.45 6.44 5.78 5.76 5.03 5.03 5.01 5.01 2.73 2.72 2.72 2.71 2.70 2.67 2.65 2.65 2.63 2.16 2.15 2.14 2.13 2.09 2.08 2.07 2.06 2.06
N
N
NH
N
3d
2.00
2.11
4.43
3.52
1.02
1.04
1.07
2.44
1.23
1.26
10.5 10.0 9.5 9.0 8.5 8.0 7.5 7.0 6.5 6.0 5.5 5.0 4.5 4.0 3.5 3.0 2.5 2.0 1.5 1.0 0.5 0.0 -0.5

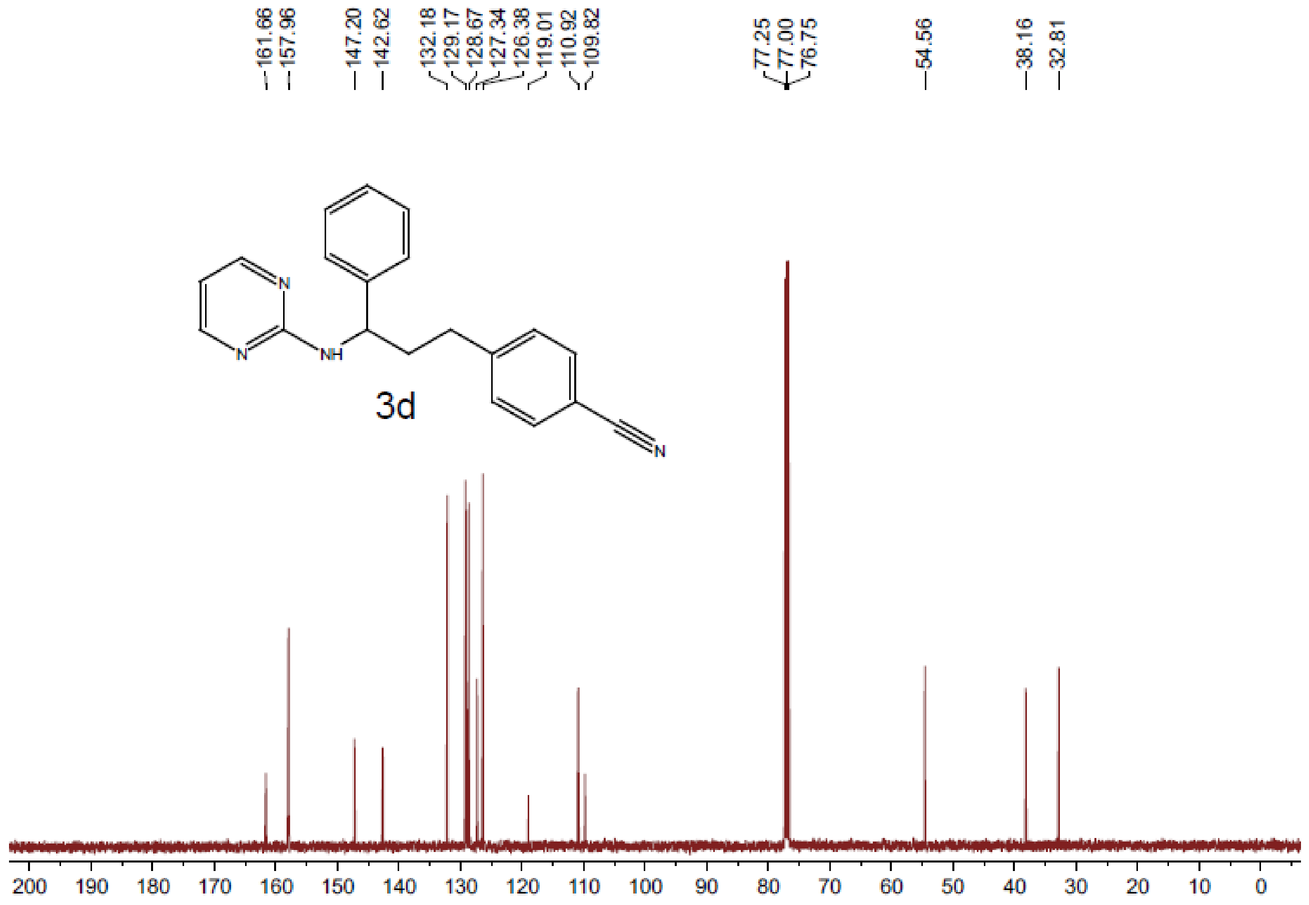
161.66
157.96
147.20
142.62
132.18
129.17
128.67
127.34
126.38
119.01
110.92
109.82
77.25
77.00
76.75
54.56
38.16
32.81
N
NH
3d
200 190 180 170 160 150 140 130 120 110 100 90 80 70 60 50 40 30 20 10 0

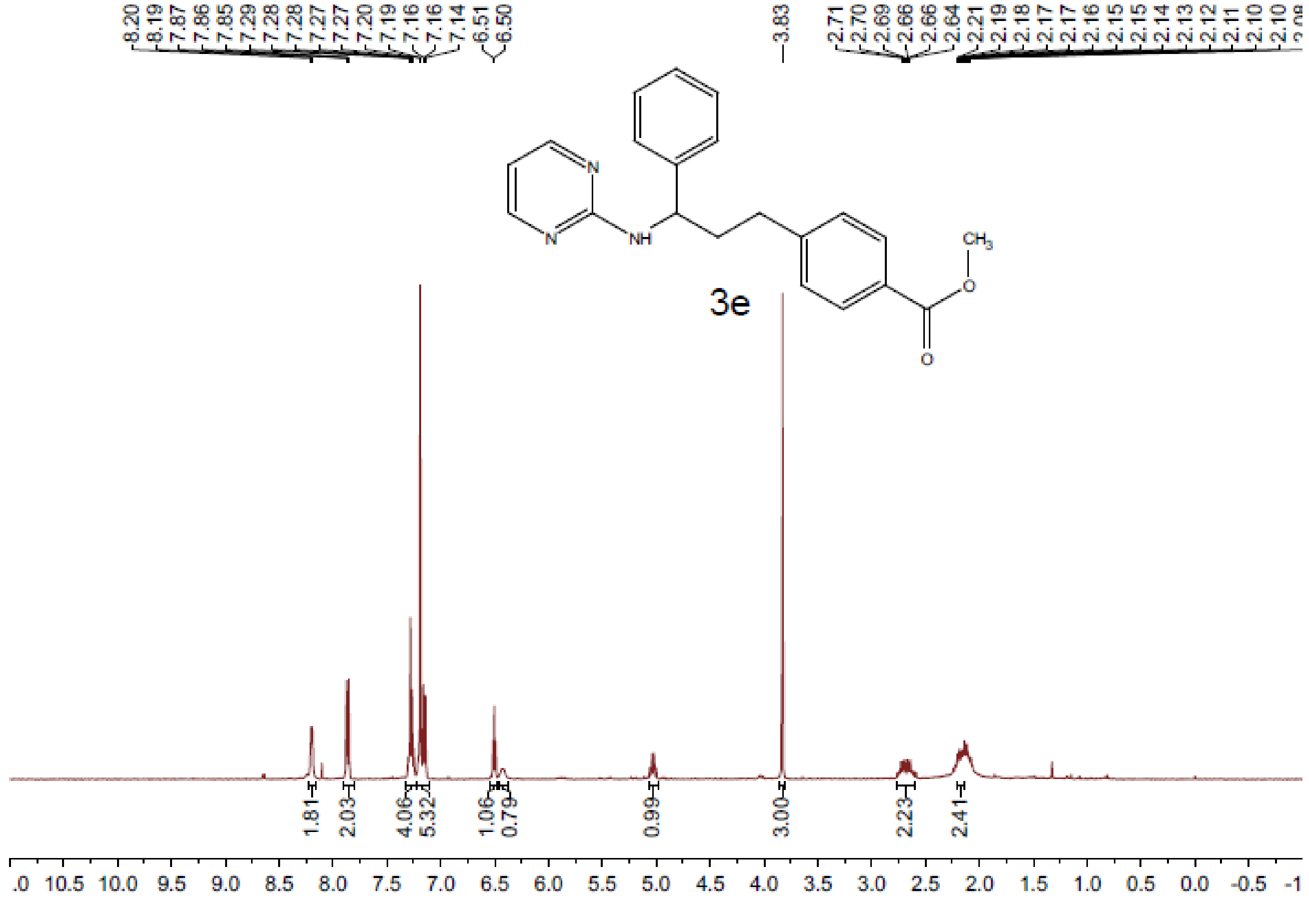
8.20
8.19
7.87
7.86
7.85
7.29
7.28
7.27
7.27
7.20
7.19
7.16
7.16
7.14
6.51
6.50
3.83
2.71
2.70
2.69
2.66
2.66
2.64
2.21
2.19
2.18
2.17
2.17
2.16
2.15
2.15
2.14
2.13
2.12
2.11
2.10
N
NH
CH3
O
3e
1.81
2.03
4.06
5.32
1.06
0.79
0.99
3.00
2.23
2.41
10.5 10.0 9.5 9.0 8.5 8.0 7.5 7.0 6.5 6.0 5.5 5.0 4.5 4.0 3.5 3.0 2.5 2.0 1.5 1.0 0.5 0.0 -0.5

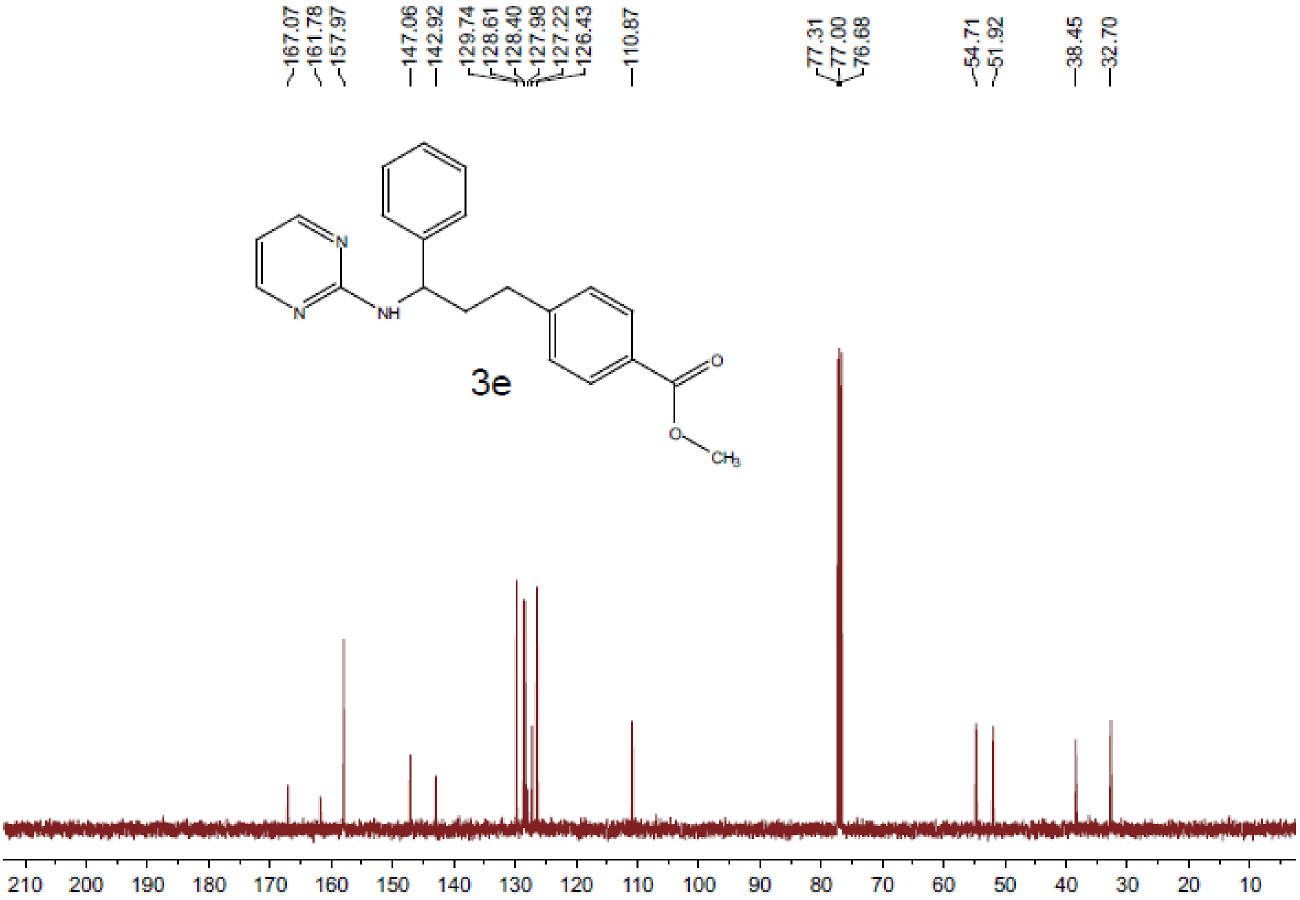
167.07
161.78
157.97
147.06
142.92
129.74
128.61
128.40
127.98
127.22
126.43
110.87
77.31
77.00
76.68
54.71
51.92
38.45
32.70
N
N
NH
O
O
CH3
3e
210 200 190 180 170 160 150 140 130 120 110 100 90 80 70 60 50 40 30 20 10

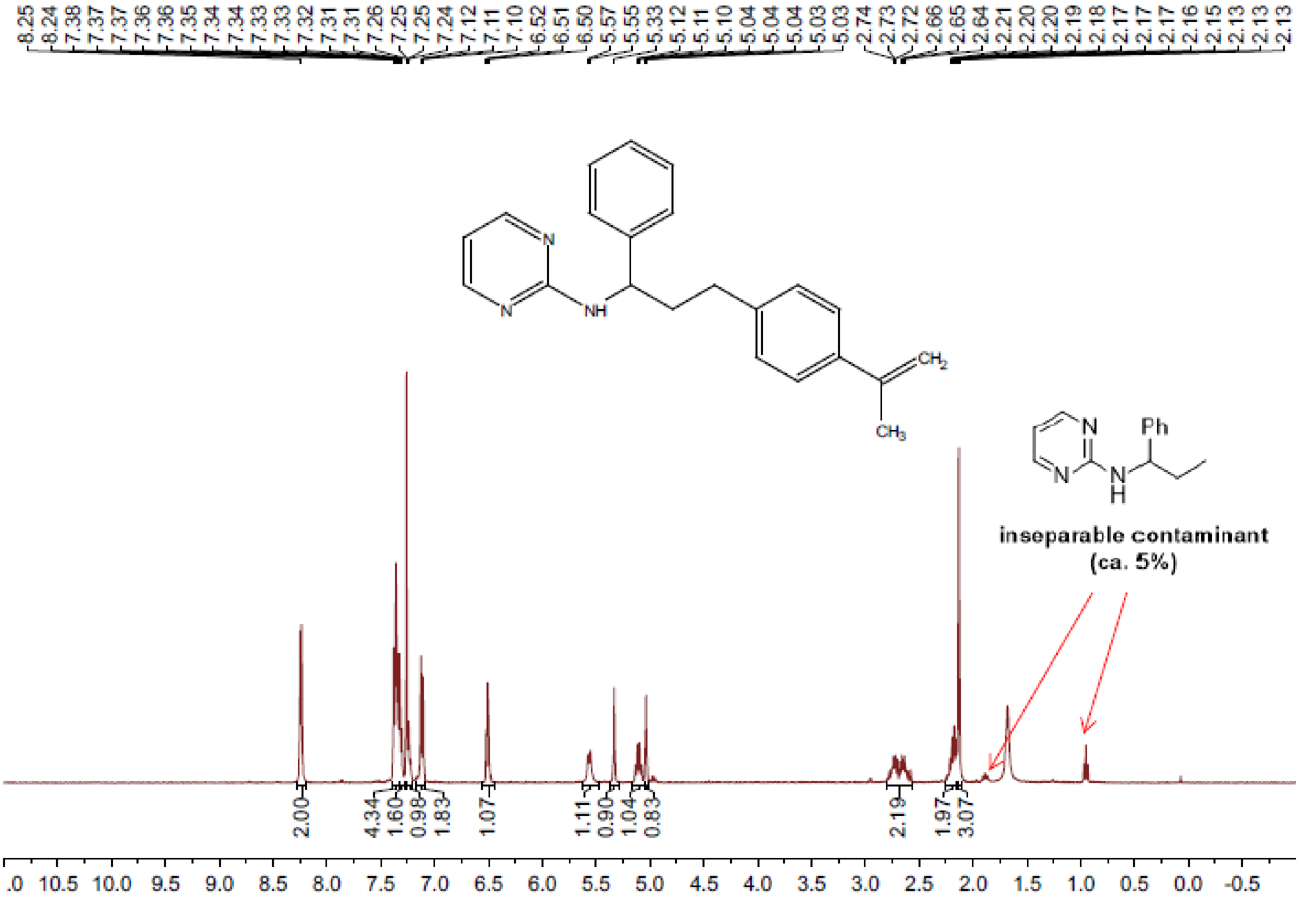
8.25
8.24
7.38
7.37
7.36
7.36
7.35
7.34
7.34
7.33
7.33
7.32
7.31
7.31
7.26
7.25
7.25
7.24
7.12
7.11
7.10
6.52
6.51
6.50
5.57
5.55
5.33
5.12
5.11
5.10
5.04
5.04
5.04
5.03
5.03
2.74
2.73
2.72
2.66
2.65
2.64
2.21
2.20
2.20
2.19
2.18
2.17
2.17
2.17
2.16
2.15
2.13
2.13
2.13
N
N
NH
CH2
CH3
N
N
N
H
Ph
inseparable contaminant
(ca. 5%)
2.00
4.34
1.60
0.98
1.83
1.07
1.11
0.90
1.04
0.83
2.19
1.97
3.07
.0 10.5 10.0 9.5 9.0 8.5 8.0 7.5 7.0 6.5 6.0 5.5 5.0 4.5 4.0 3.5 3.0 2.5 2.0 1.5 1.0 0.5 0.0 -0.5

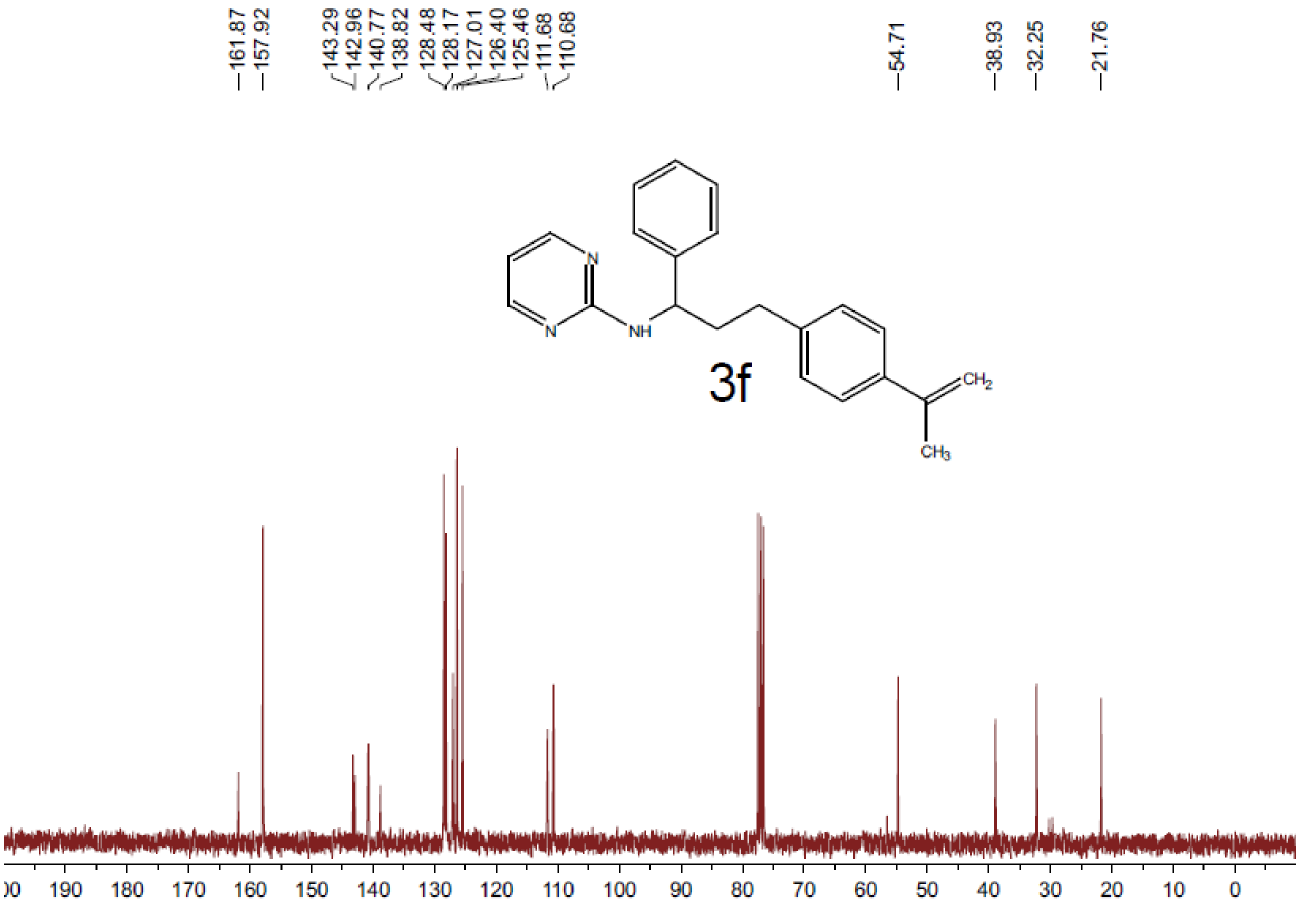
161.87
157.92
143.29
142.96
140.77
138.82
128.48
128.17
127.01
126.40
125.46
111.68
110.68
54.71
38.93
32.25
21.76
N
N
NH
3f
CH2
CH3
00 190 180 170 160 150 140 130 120 110 100 90 80 70 60 50 40 30 20 10 0

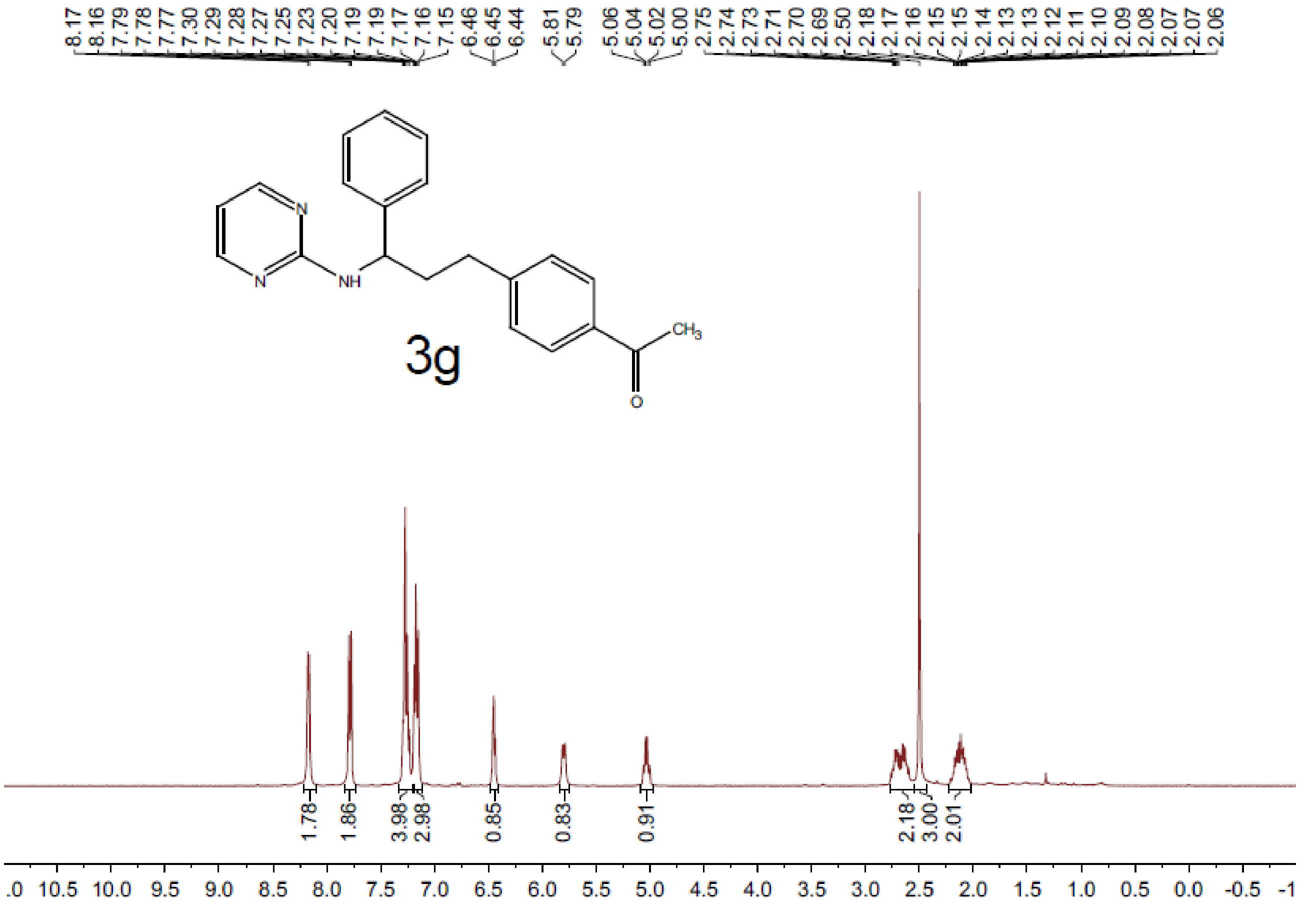
8.17
8.16
7.79
7.78
7.77
7.30
7.29
7.28
7.27
7.25
7.23
7.20
7.19
7.19
7.17
7.16
7.15
6.46
6.45
6.44
5.81
5.79
5.06
5.04
5.02
5.00
2.75
2.74
2.73
2.71
2.70
2.69
2.50
2.18
2.17
2.16
2.15
2.15
2.14
2.13
2.13
2.12
2.11
2.10
2.09
2.08
2.07
2.07
2.06
N
N
NH
3g
CH3
O
1.78
1.86
3.98
2.98
0.85
0.83
0.91
2.18
3.00
2.01
.0 10.5 10.0 9.5 9.0 8.5 8.0 7.5 7.0 6.5 6.0 5.5 5.0 4.5 4.0 3.5 3.0 2.5 2.0 1.5 1.0 0.5 0.0 -0.5 -1

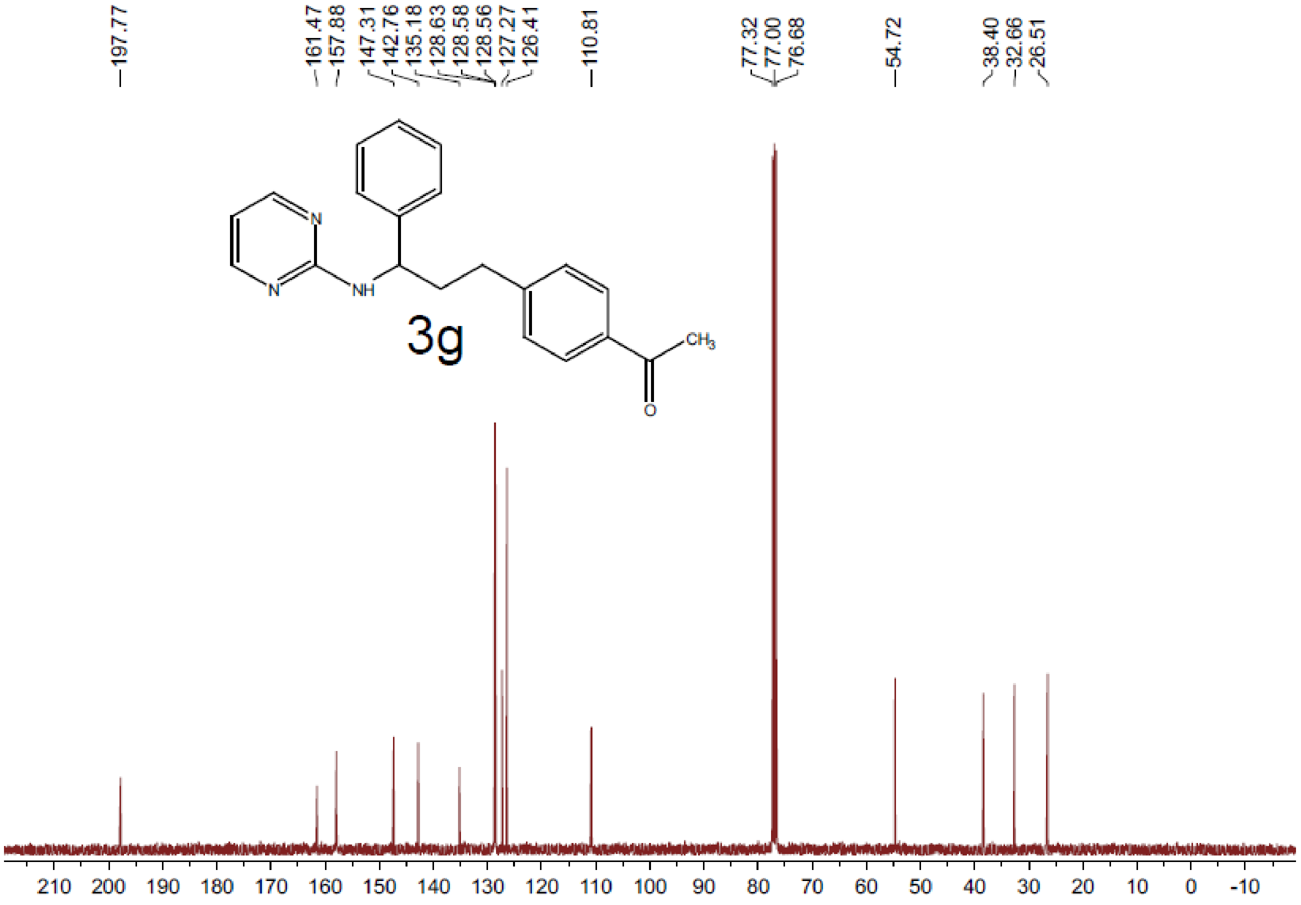
197.77
161.47
157.88
147.31
142.76
135.18
128.63
128.58
128.56
127.27
126.41
110.81
77.32
77.00
76.68
54.72
38.40
32.66
26.51
N
N
NH
CH3
O
3g
210 200 190 180 170 160 150 140 130 120 110 100 90 80 70 60 50 40 30 20 10 0 -10

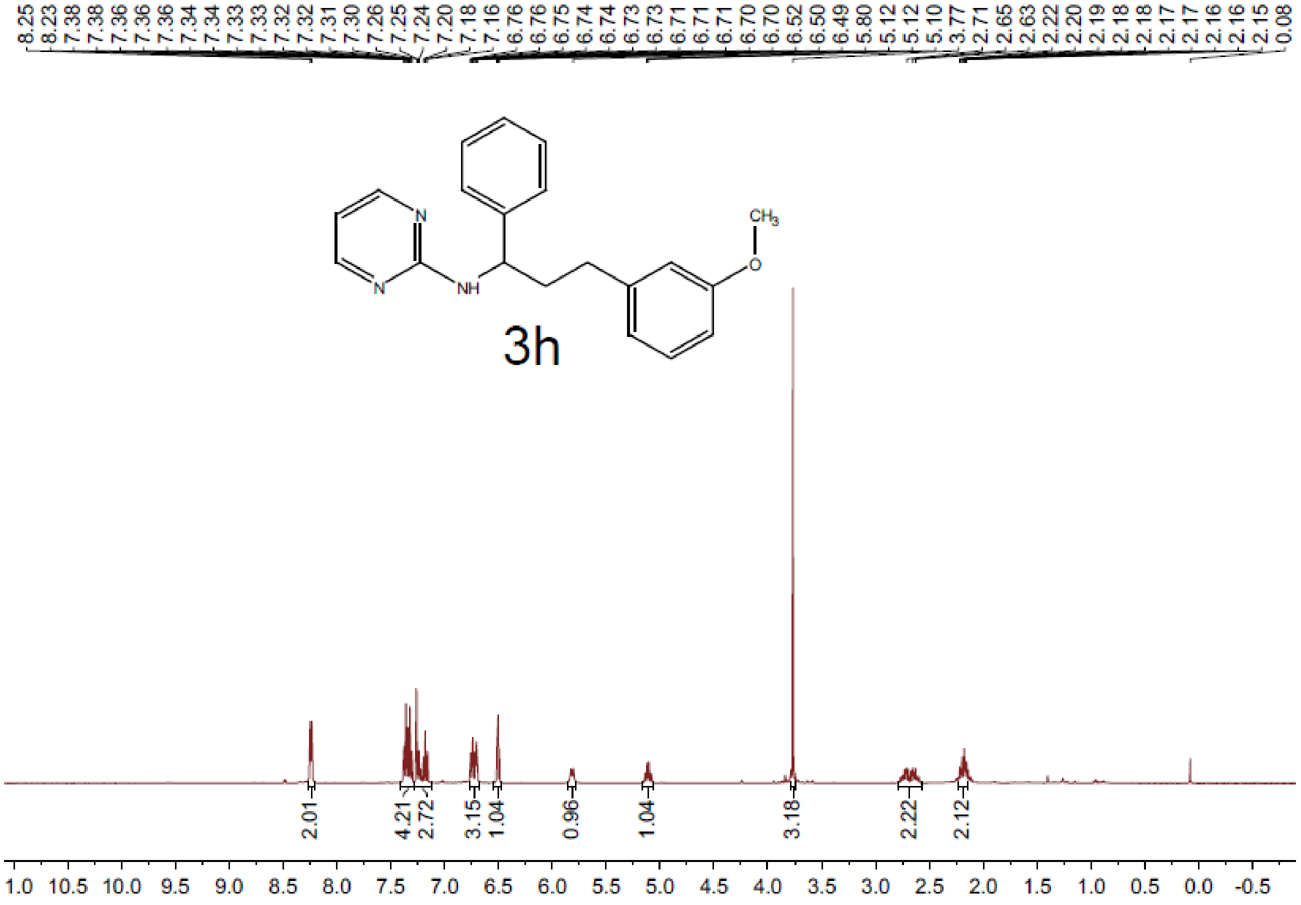
8.25
8.23
7.38
7.38
7.36
7.36
7.36
7.34
7.34
7.33
7.33
7.32
7.32
7.31
7.30
7.26
7.25
7.24
7.20
7.18
7.16
6.76
6.75
6.74
6.74
6.73
6.73
6.71
6.71
6.71
6.70
6.70
6.52
6.50
6.49
5.80
5.12
5.12
5.10
3.77
2.71
2.65
2.63
2.22
2.20
2.19
2.18
2.18
2.17
2.17
2.16
2.16
2.15
0.08
N
N
NH
CH3
O
3h
2.01
4.21
2.72
3.15
1.04
0.96
1.04
3.18
2.22
2.12
1.0 10.5 10.0 9.5 9.0 8.5 8.0 7.5 7.0 6.5 6.0 5.5 5.0 4.5 4.0 3.5 3.0 2.5 2.0 1.5 1.0 0.5 0.0 -0.5

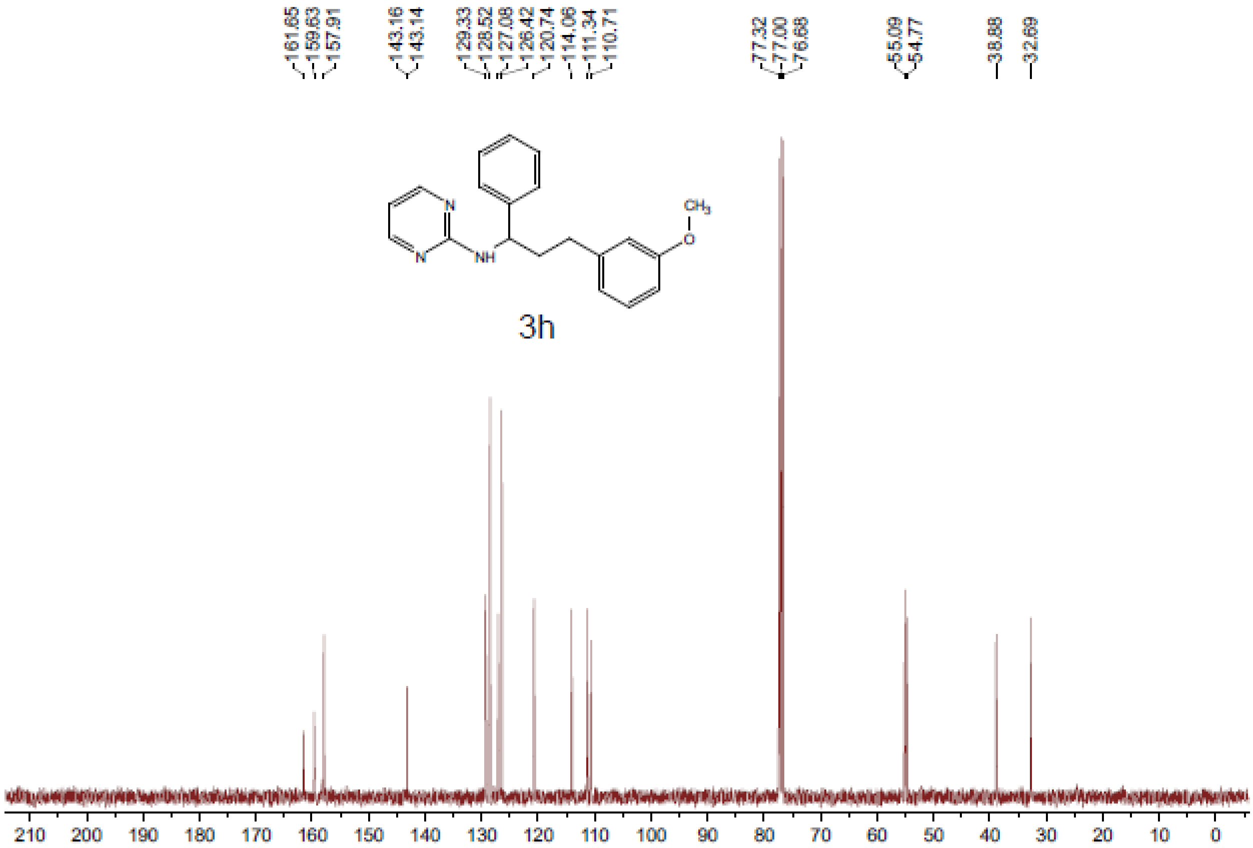
161.65
159.63
157.91
143.16
143.14
129.33
128.52
127.08
126.42
120.74
114.06
111.34
110.71
77.32
77.00
76.68
55.09
54.77
38.88
32.69
CH3
O
N
N
NH
3h
210 200 190 180 170 160 150 140 130 120 110 100 90 80 70 60 50 40 30 20 10 0

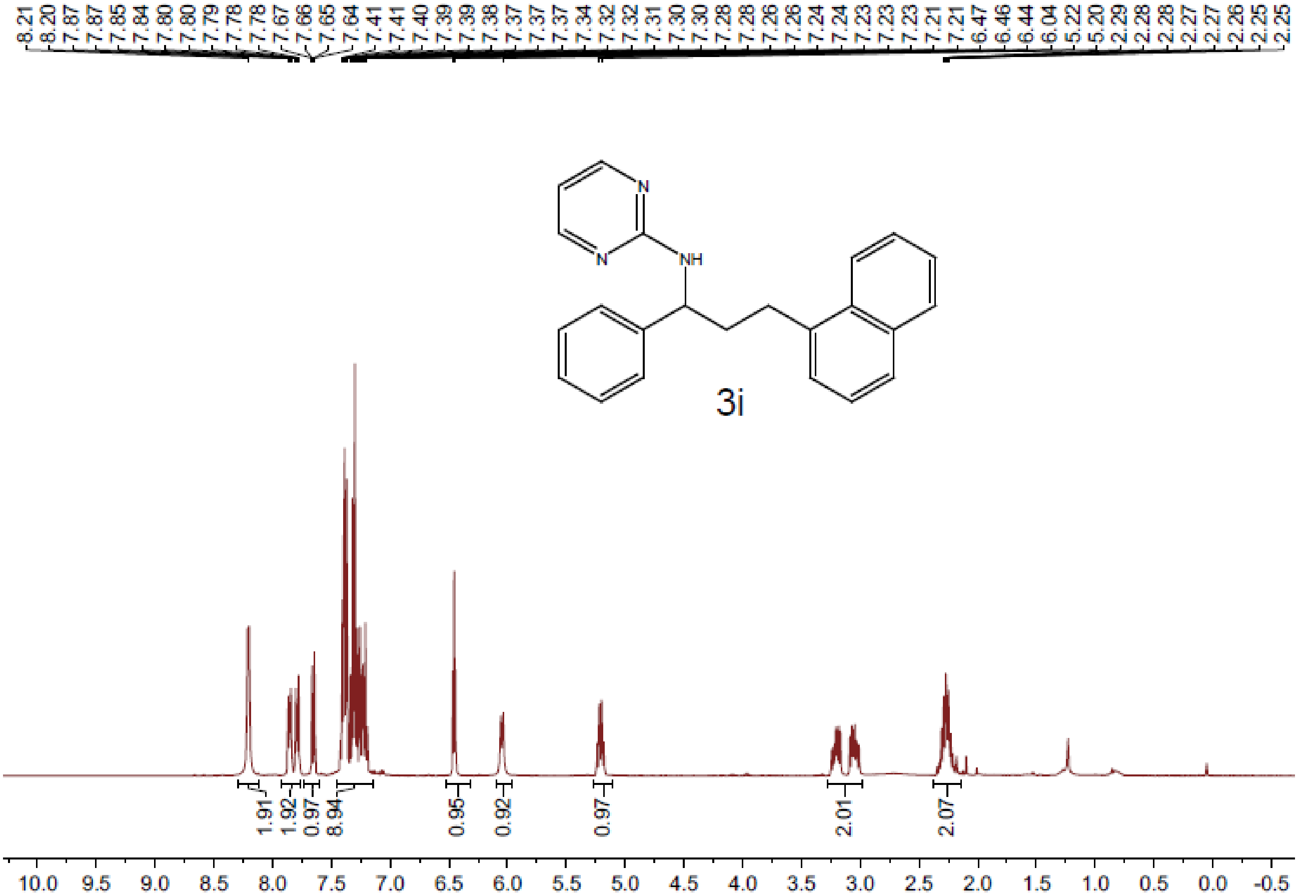
8.21 8.20 7.87 7.85 7.84 7.80 7.80 7.79 7.78 7.67 7.66 7.65 7.64 7.41 7.40 7.39 7.39 7.38 7.37 7.37 7.37 7.34 7.32 7.32 7.31 7.30 7.30 7.28 7.28 7.26 7.26 7.24 7.24 7.23 7.23 7.23 7.21 7.21 6.47 6.46 6.44 6.04 5.22 5.20 2.29 2.28 2.28 2.27 2.27 2.26 2.25 2.25
N
N
NH
3i
1.91
1.92
0.97
8.94
0.95
0.92
0.97
2.01
2.07
10.0 9.5 9.0 8.5 8.0 7.5 7.0 6.5 6.0 5.5 5.0 4.5 4.0 3.5 3.0 2.5 2.0 1.5 1.0 0.5 0.0 -0.5

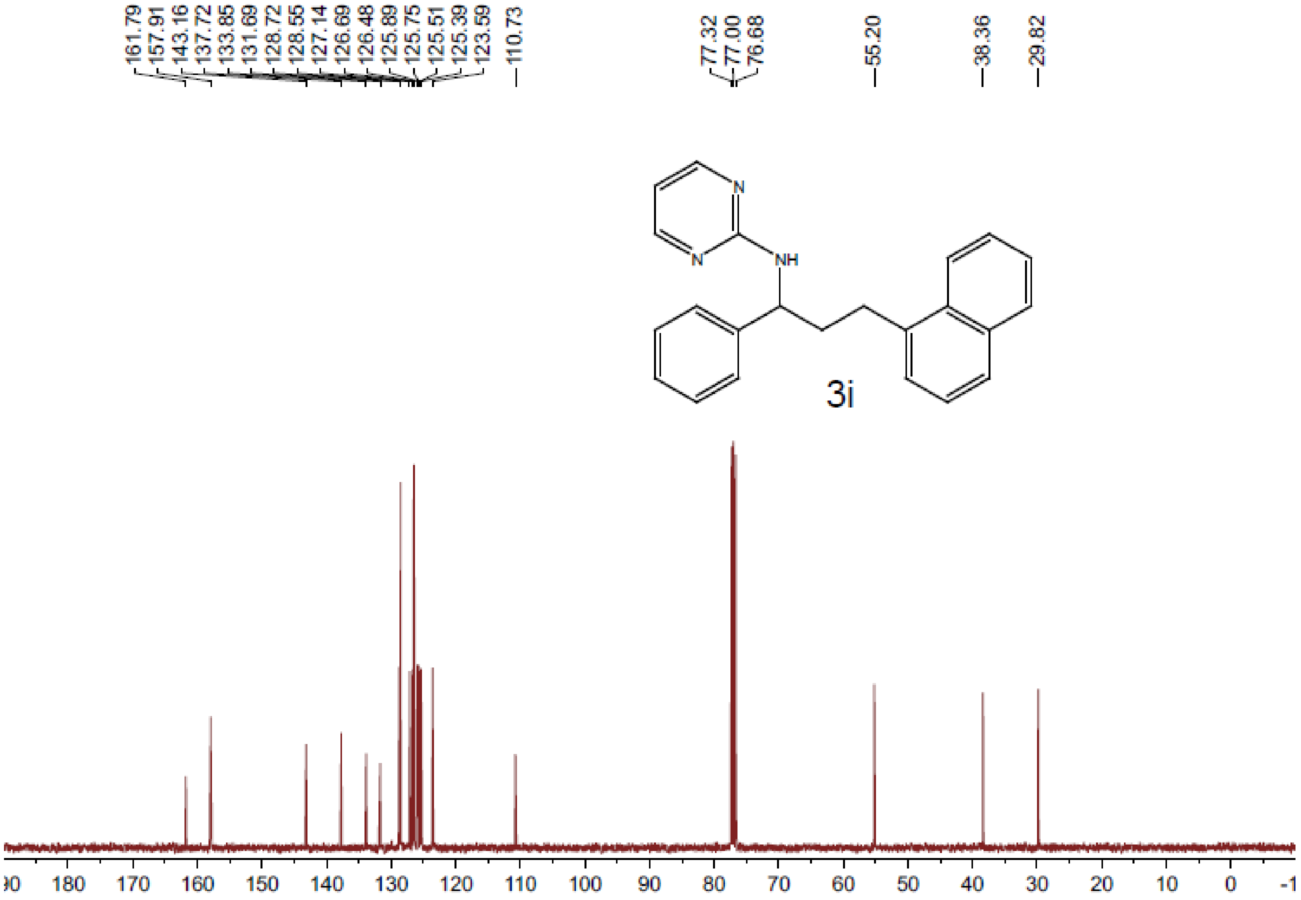
3i

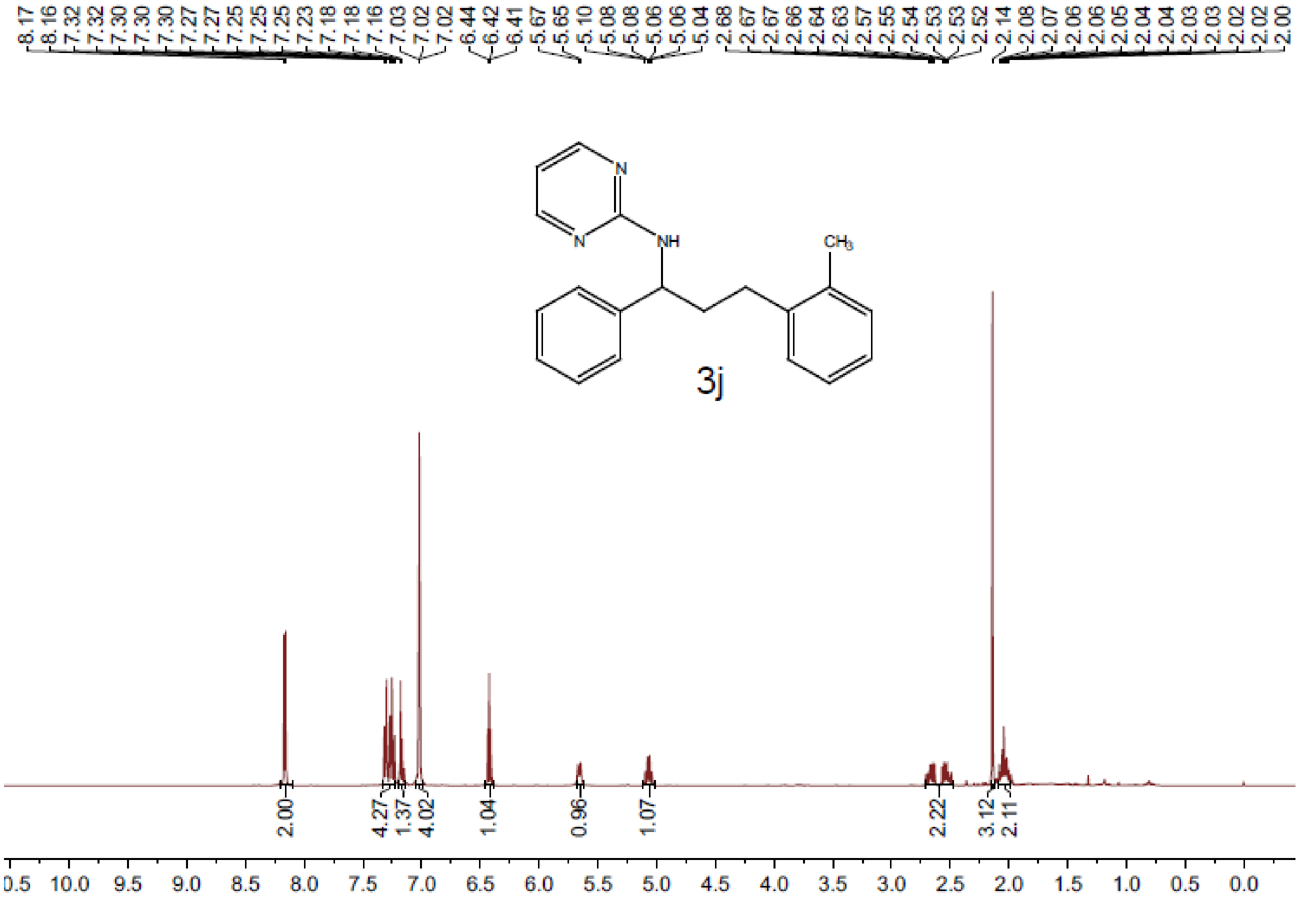
3j

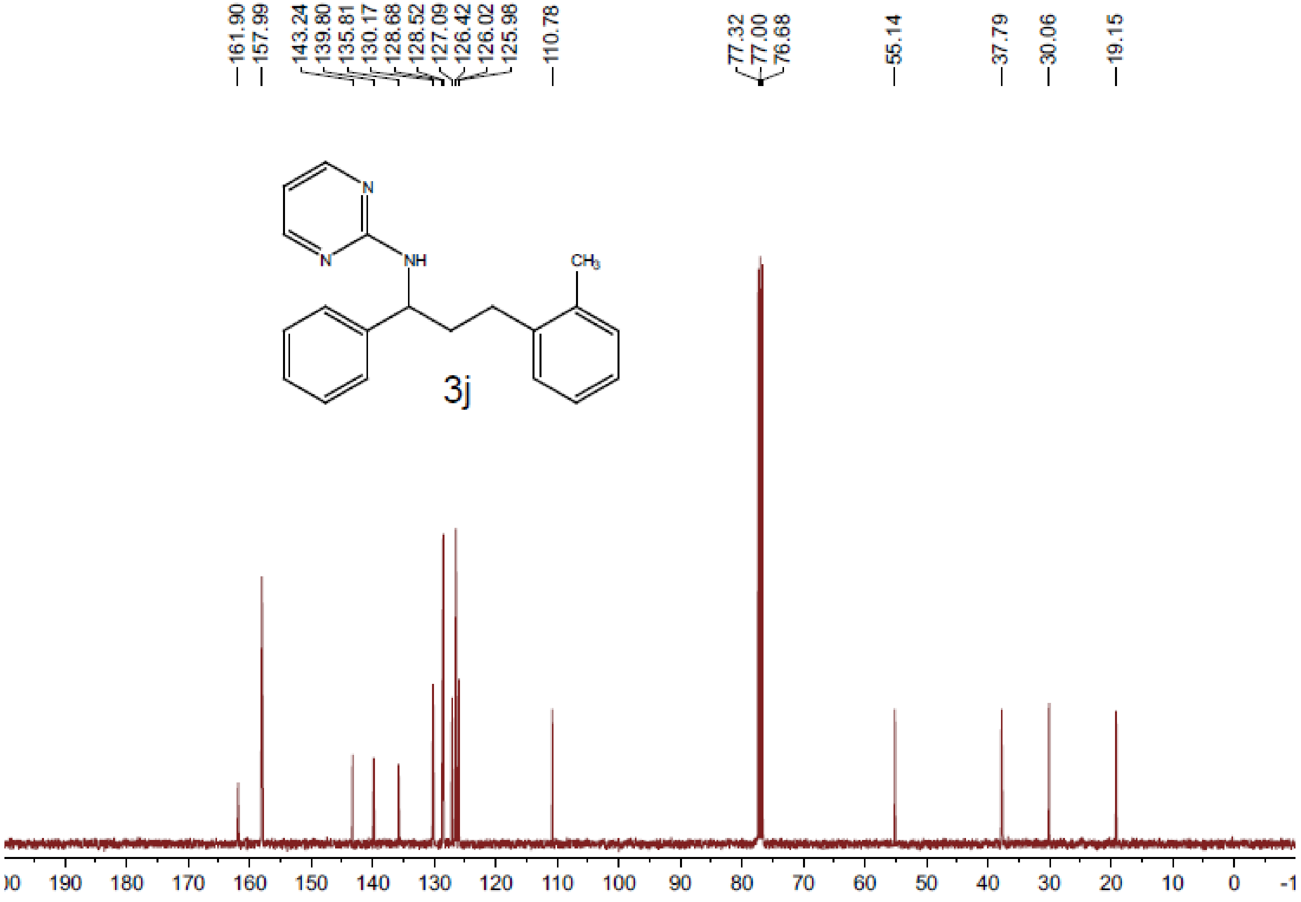
161.90
157.99
143.24
139.80
135.81
130.17
128.68
128.52
127.09
126.42
126.02
125.98
110.78
77.32
77.00
76.68
55.14
37.79
30.06
19.15
N
N
NH
CH3
3j
190 180 170 160 150 140 130 120 110 100 90 80 70 60 50 40 30 20 10 0

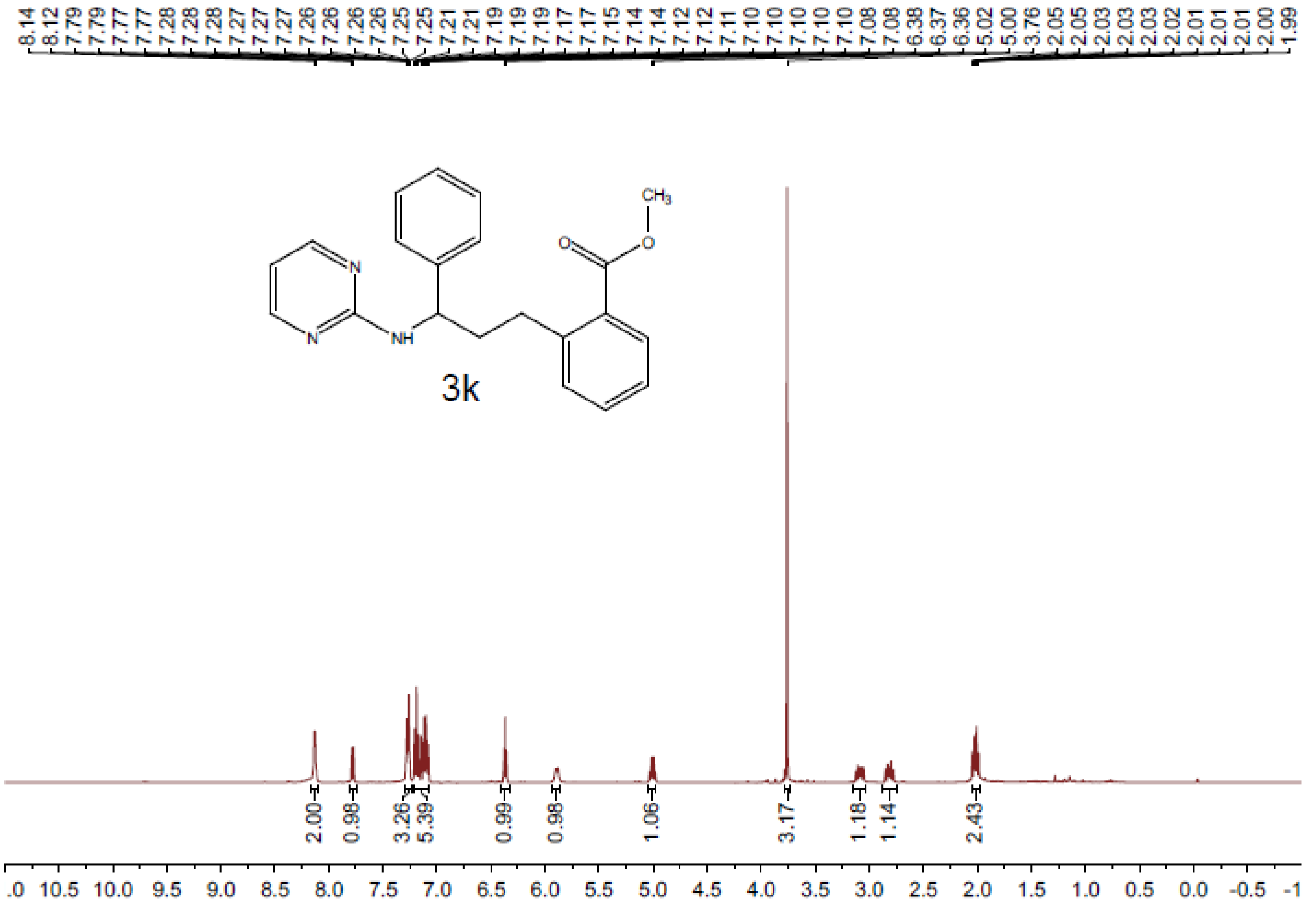
8.14 8.12 7.79 7.79 7.77 7.77 7.28 7.28 7.28 7.27 7.27 7.27 7.26 7.26 7.26 7.26 7.25 7.25 7.21 7.21 7.19 7.19 7.17 7.17 7.15 7.14 7.14 7.12 7.11 7.10 7.10 7.10 7.10 7.10 7.08 7.08 6.38 6.37 6.36 5.02 5.00 3.76 2.05 2.05 2.03 2.03 2.03 2.02 2.01 2.01 2.01 2.00 1.99
N
N
NH
O
O
CH3
3k
2.00
0.98
3.26
5.39
0.99
0.98
1.06
3.17
1.18
1.14
2.43
10.5 10.0 9.5 9.0 8.5 8.0 7.5 7.0 6.5 6.0 5.5 5.0 4.5 4.0 3.5 3.0 2.5 2.0 1.5 1.0 0.5 0.0 -0.5

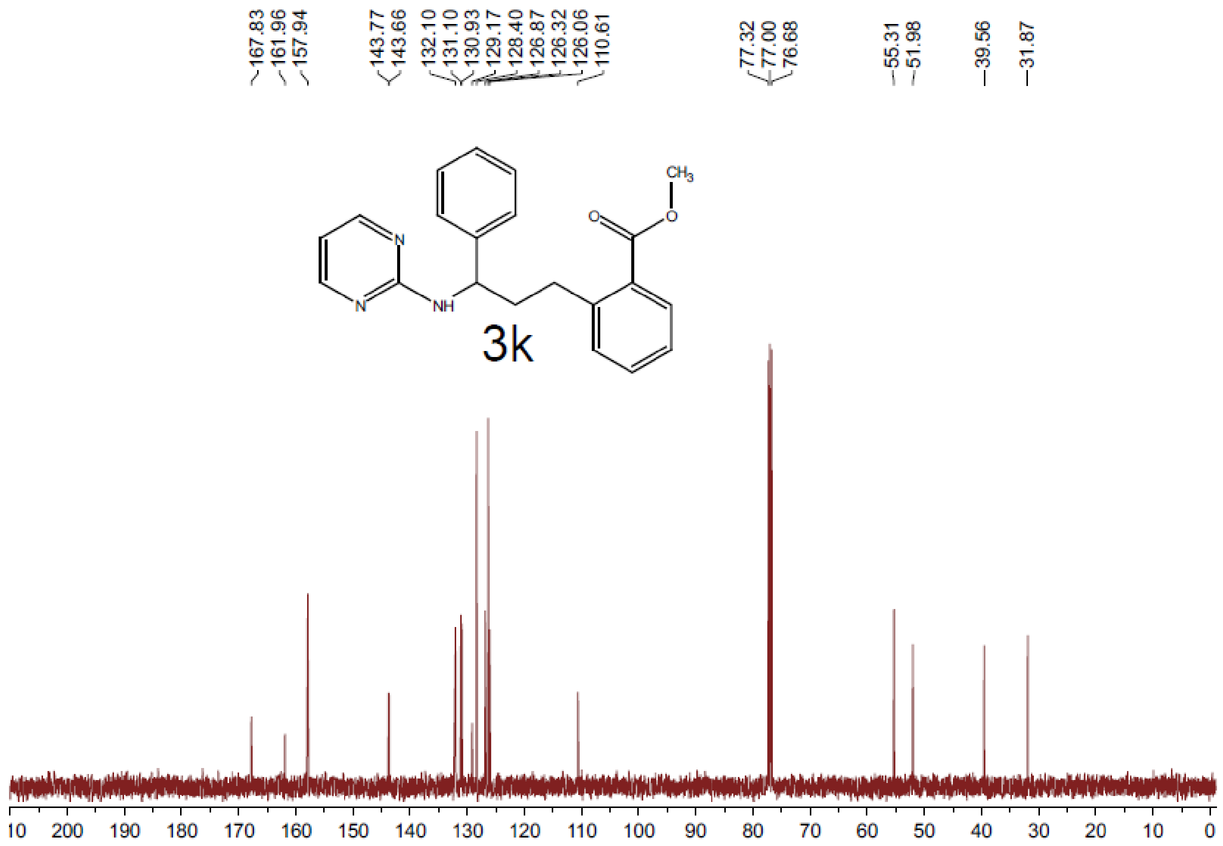

167.83
161.96
157.94
143.77
143.66
132.10
131.10
130.93
129.17
128.40
126.87
126.32
126.06
110.61
77.32
77.00
76.68
55.31
51.98
39.56
31.87
CH3
O
O
N
N
NH
3k
200 190 180 170 160 150 140 130 120 110 100 90 80 70 60 50 40 30 20 10 0

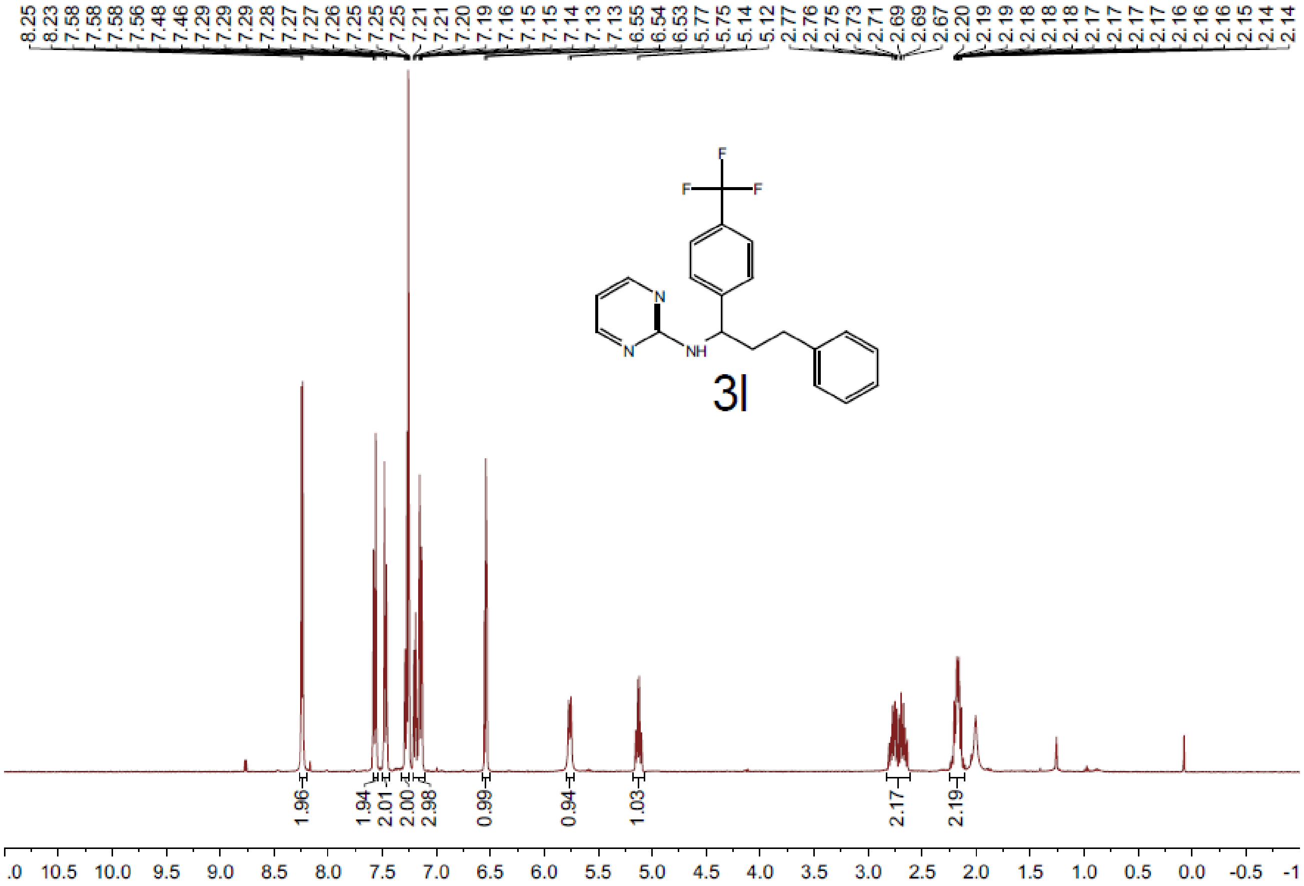

8.25 8.23 7.58 7.58 7.56 7.48 7.46 7.29 7.29 7.29 7.28 7.27 7.26 7.25 7.25 7.25 7.21 7.21 7.20 7.19 7.16 7.15 7.15 7.14 7.13 7.13 6.55 6.54 6.53 5.77 5.75 5.14 5.12 2.77 2.76 2.75 2.73 2.71 2.69 2.69 2.67 2.20 2.19 2.19 2.18 2.18 2.18 2.17 2.17 2.17 2.16 2.16 2.15 2.14 2.14
F
F
F
N
N
NH
3l
1.96
1.94
2.01
2.00
2.98
0.99
0.94
1.03
2.17
2.19
10.5 10.0 9.5 9.0 8.5 8.0 7.5 7.0 6.5 6.0 5.5 5.0 4.5 4.0 3.5 3.0 2.5 2.0 1.5 1.0 0.5 0.0 -0.5

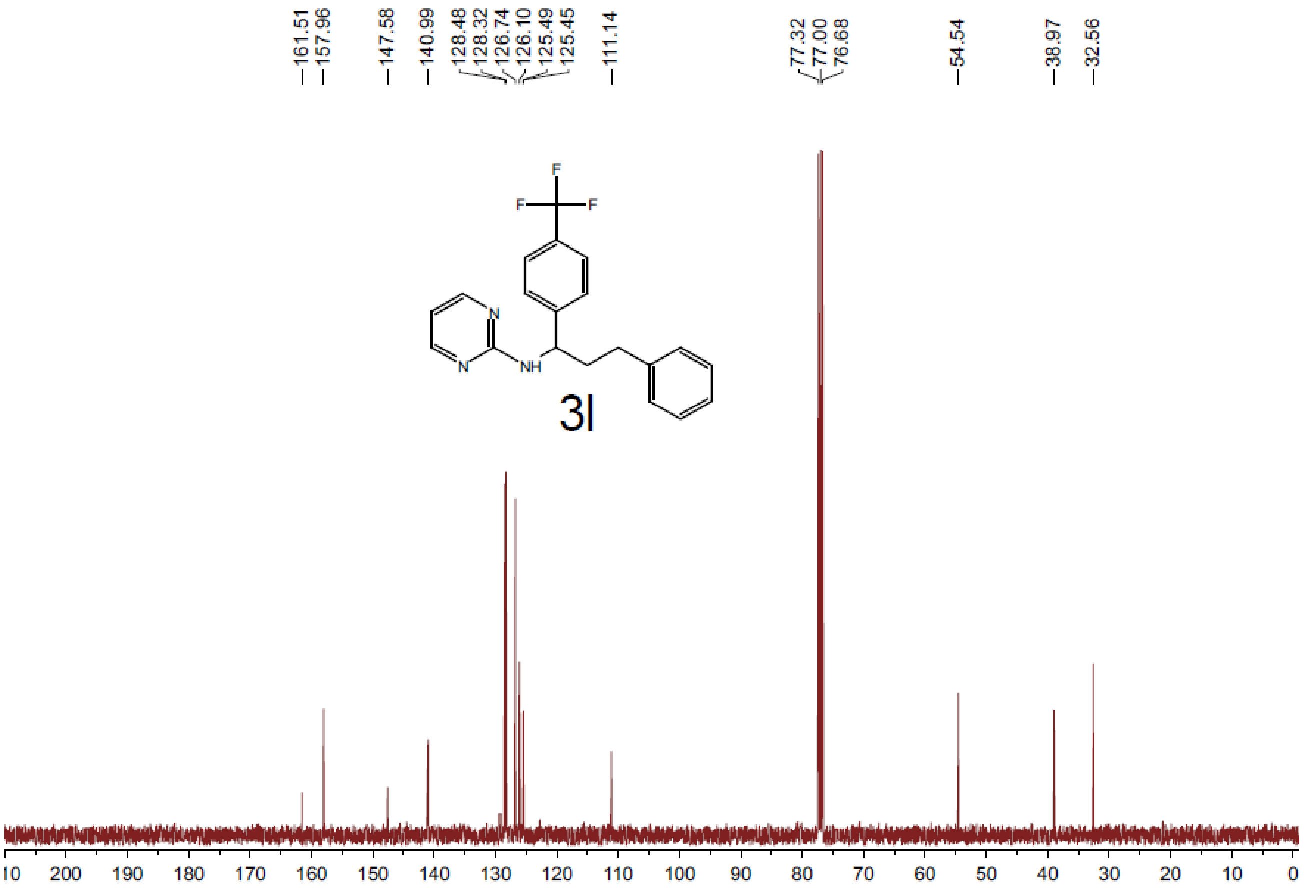

161.51
157.96
147.58
140.99
128.48
128.32
126.74
126.10
125.49
125.45
111.14
77.32
77.00
76.68
54.54
38.97
32.56
F
F
F
N
N
NH
3l
10
200
190
180
170
160
150
140
130
120
110
100
90
80
70
60
50
40
30
20
10
0

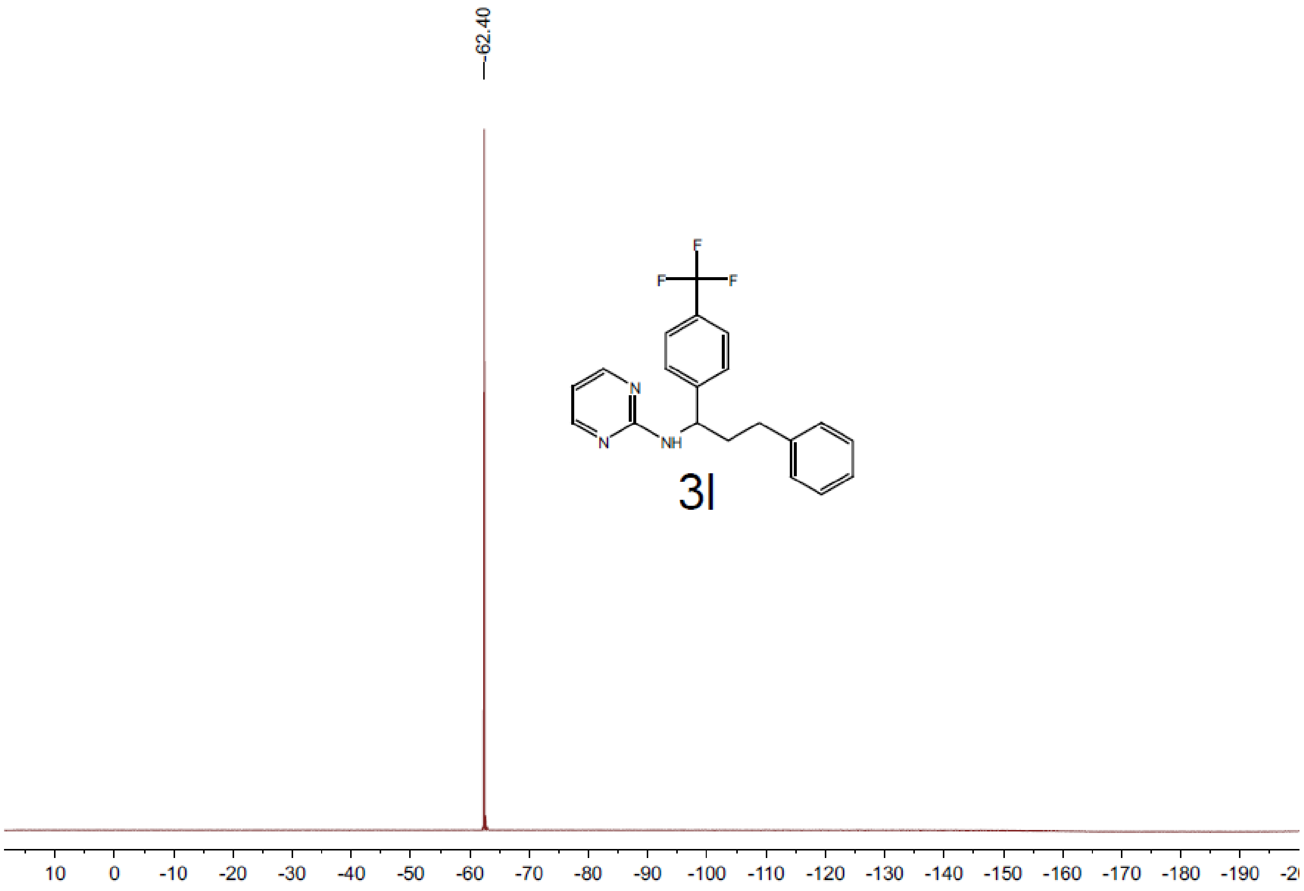

-62.40
F
F
F
N
N
NH
3l
10
0
-10
-20
-30
-40
-50
-60
-70
-80
-90
-100
-110
-120
-130
-140
-150
-160
-170
-180
-190

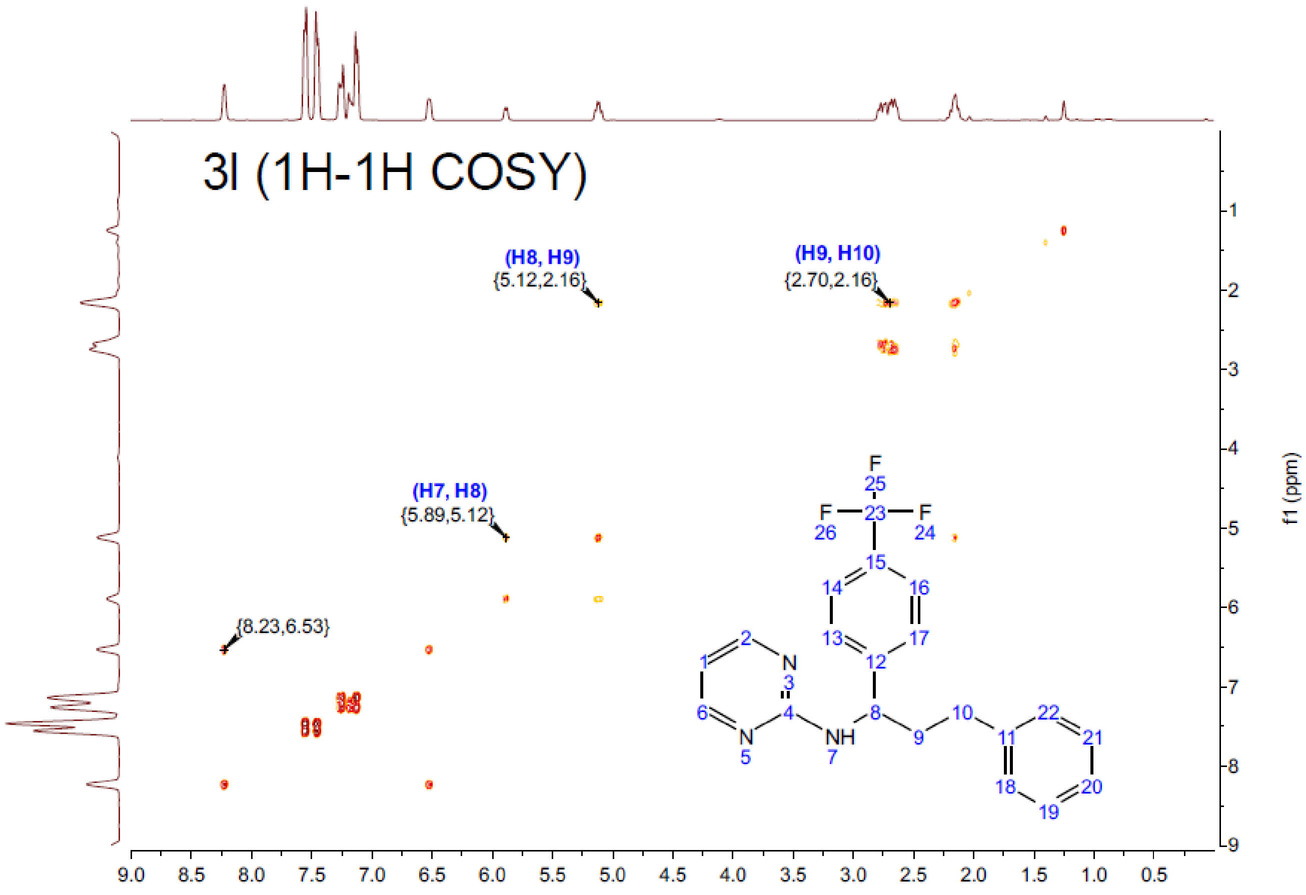
3l (1H-1H COSY)
(H8, H9)
{5.12,2.16}
(H9, H10)
{2.70,2.16}
(H7, H8)
{5.89,5.12}
{8.23,6.53}
f1 (ppm)

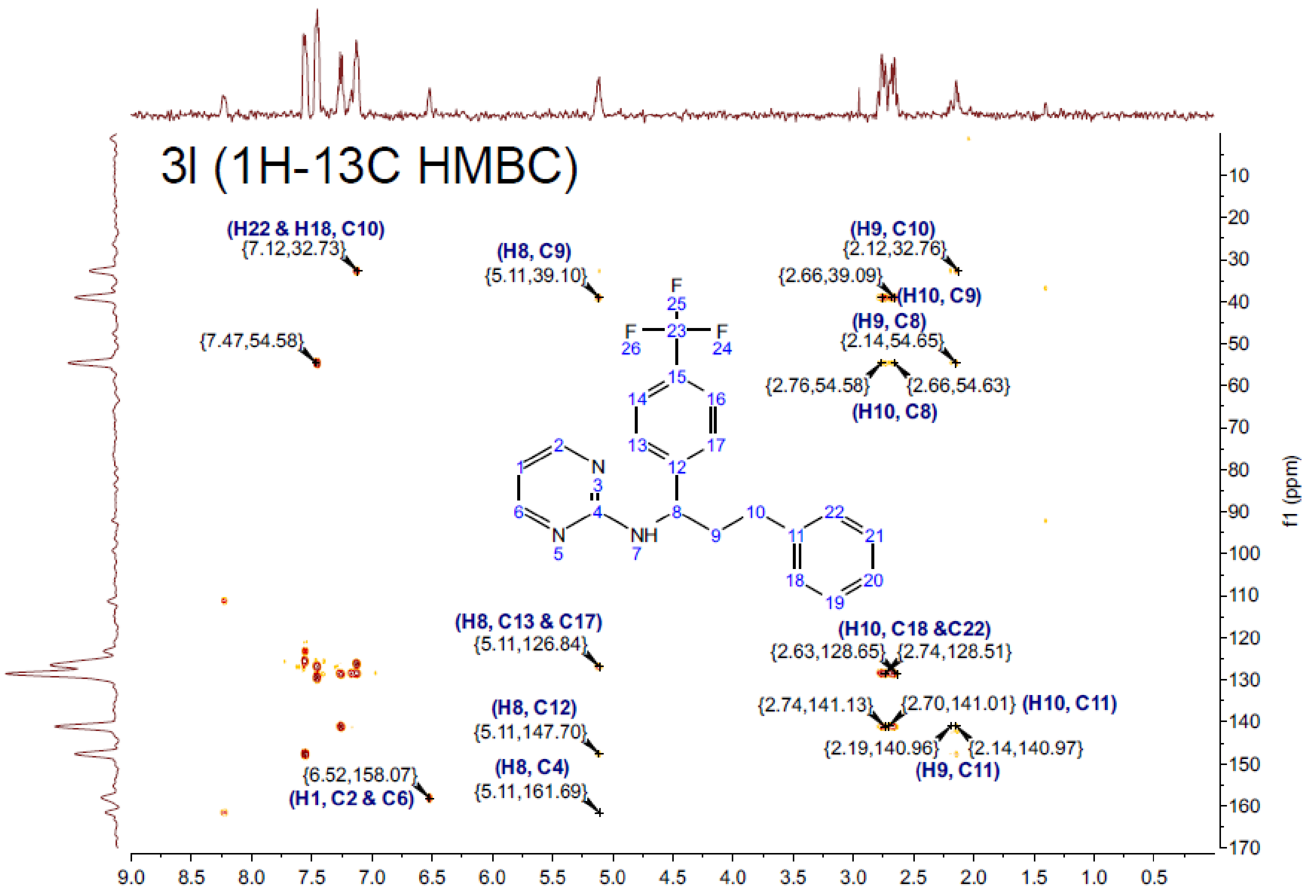
3l (1H-13C HMBC)
(H22 & H18, C10)
{7.12,32.73}
(H8, C9)
{5.11,39.10}
(H9, C10)
{2.12,32.76}
{2.66,39.09}
(H10, C9)
(H9, C8)
{2.14,54.65}
{7.47,54.58}
{2.76,54.58}
{2.66,54.63}
(H10, C8)
(H8, C13 & C17)
{5.11,126.84}
(H10, C18 &C22)
{2.63,128.65}
{2.74,128.51}
(H8, C12)
{5.11,147.70}
{2.74,141.13}
{2.70,141.01}
(H10, C11)
{2.19,140.96}
{2.14,140.97}
(H9, C11)
{6.52,158.07}
(H1, C2 & C6)
(H8, C4)
{5.11,161.69}
f1 (ppm)

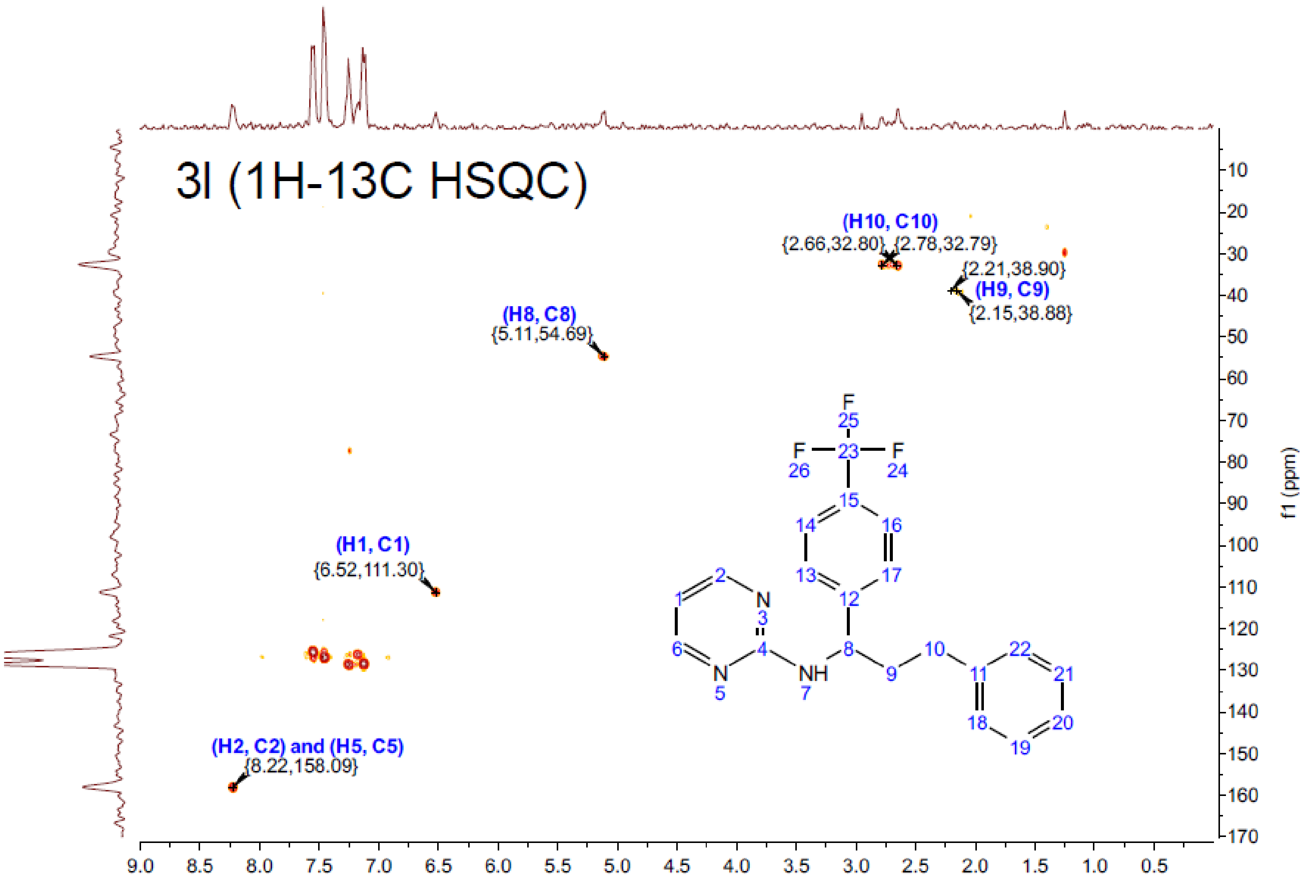
3l (1H-13C HSQC)
(H10, C10)
{2.66,32.80}
{2.78,32.79}
{2.21,38.90}
(H9, C9)
{2.15,38.88}
(H8, C8)
{5.11,54.69}
(H1, C1)
{6.52,111.30}
(H2, C2) and (H5, C5)
{8.22,158.09}
f1 (ppm)

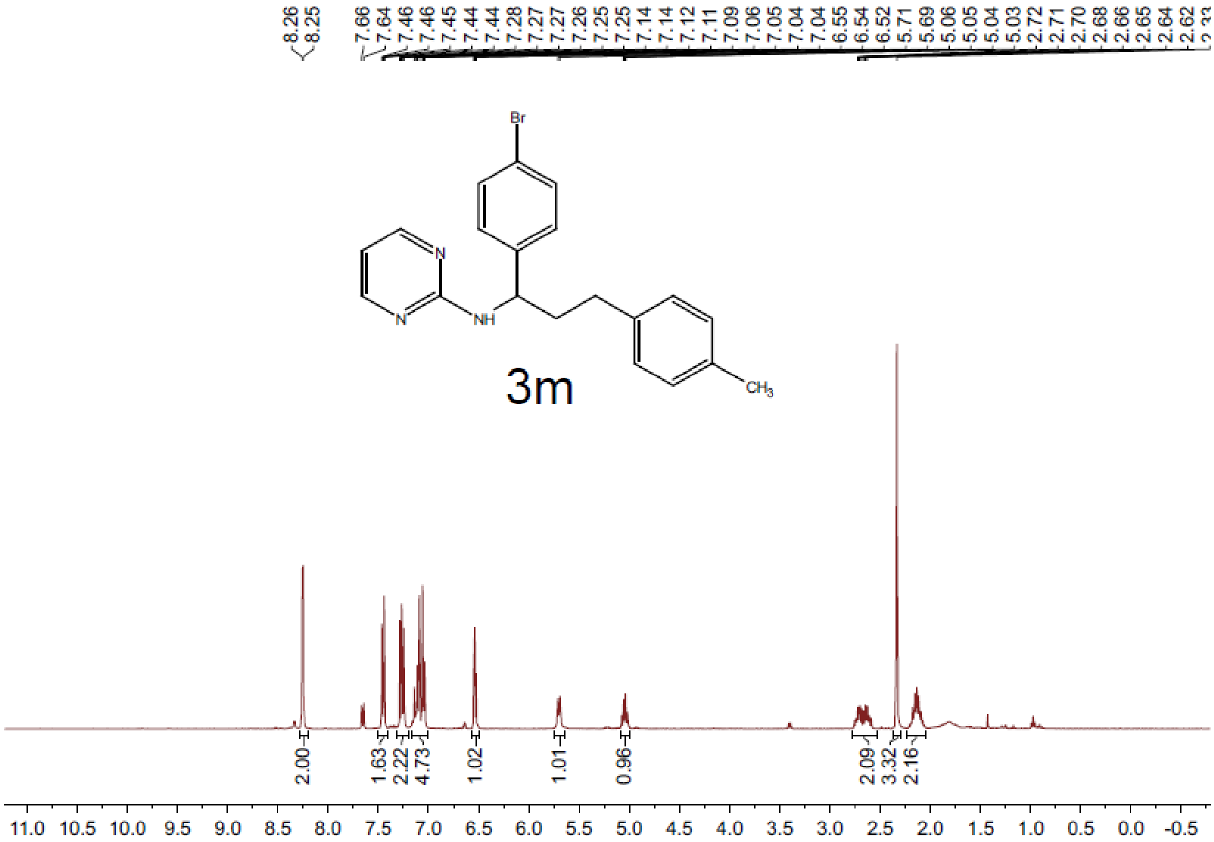
3m

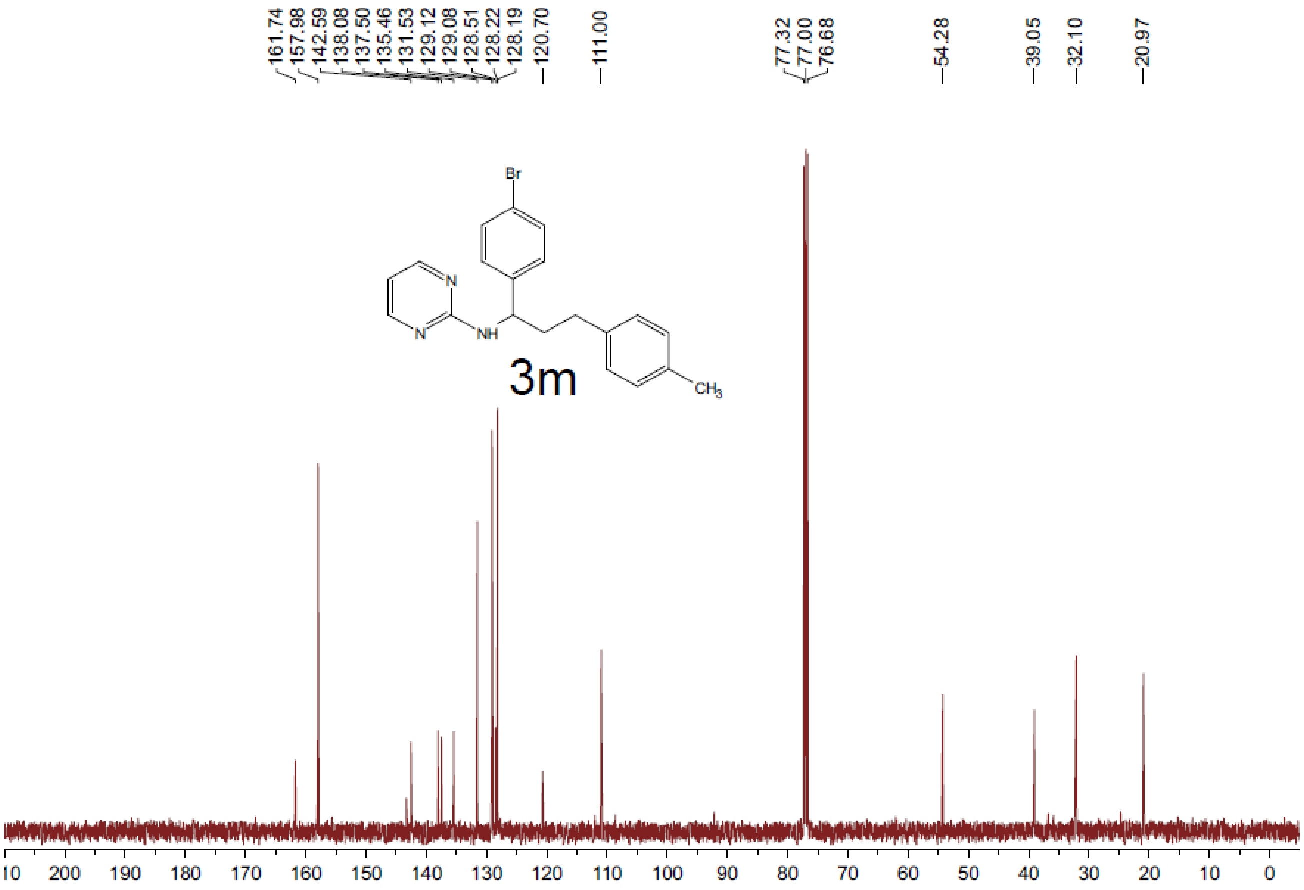
161.74
157.98
142.59
138.08
137.50
135.46
131.53
129.12
129.08
128.51
128.22
128.19
120.70
111.00
77.32
77.00
76.68
54.28
39.05
32.10
20.97
Br
N
N
NH
3m
CH3

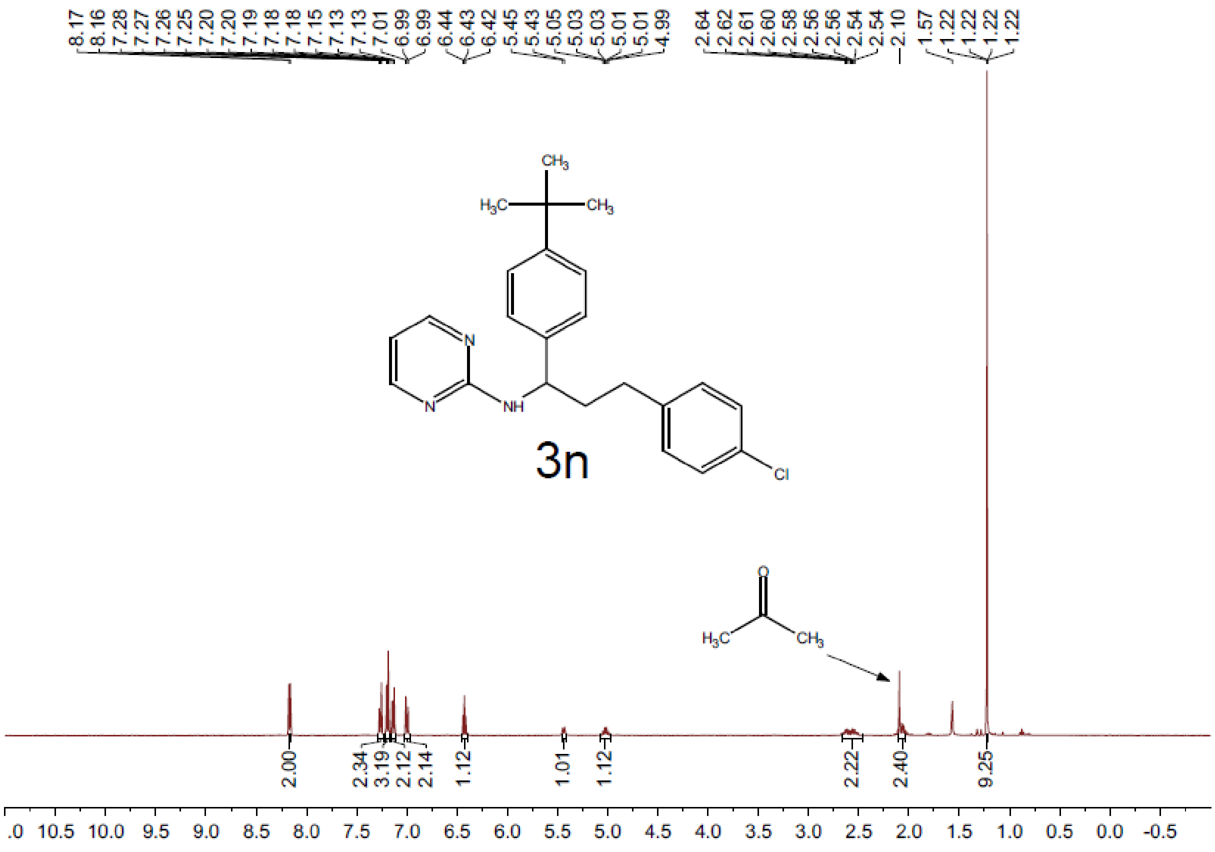
8.17
8.16
7.28
7.27
7.26
7.25
7.20
7.20
7.19
7.18
7.18
7.15
7.13
7.13
7.01
6.99
6.99
6.44
6.43
6.42
5.45
5.43
5.05
5.03
5.03
5.01
5.01
4.99
2.64
2.62
2.61
2.60
2.58
2.56
2.56
2.54
2.54
2.10
1.57
1.22
1.22
1.22
1.22
CH3
H3C
CH3
N
N
NH
3n
Cl
O
H3C
CH3
2.00
2.34
3.19
2.12
2.14
1.12
1.01
1.12
2.22
2.40
9.25

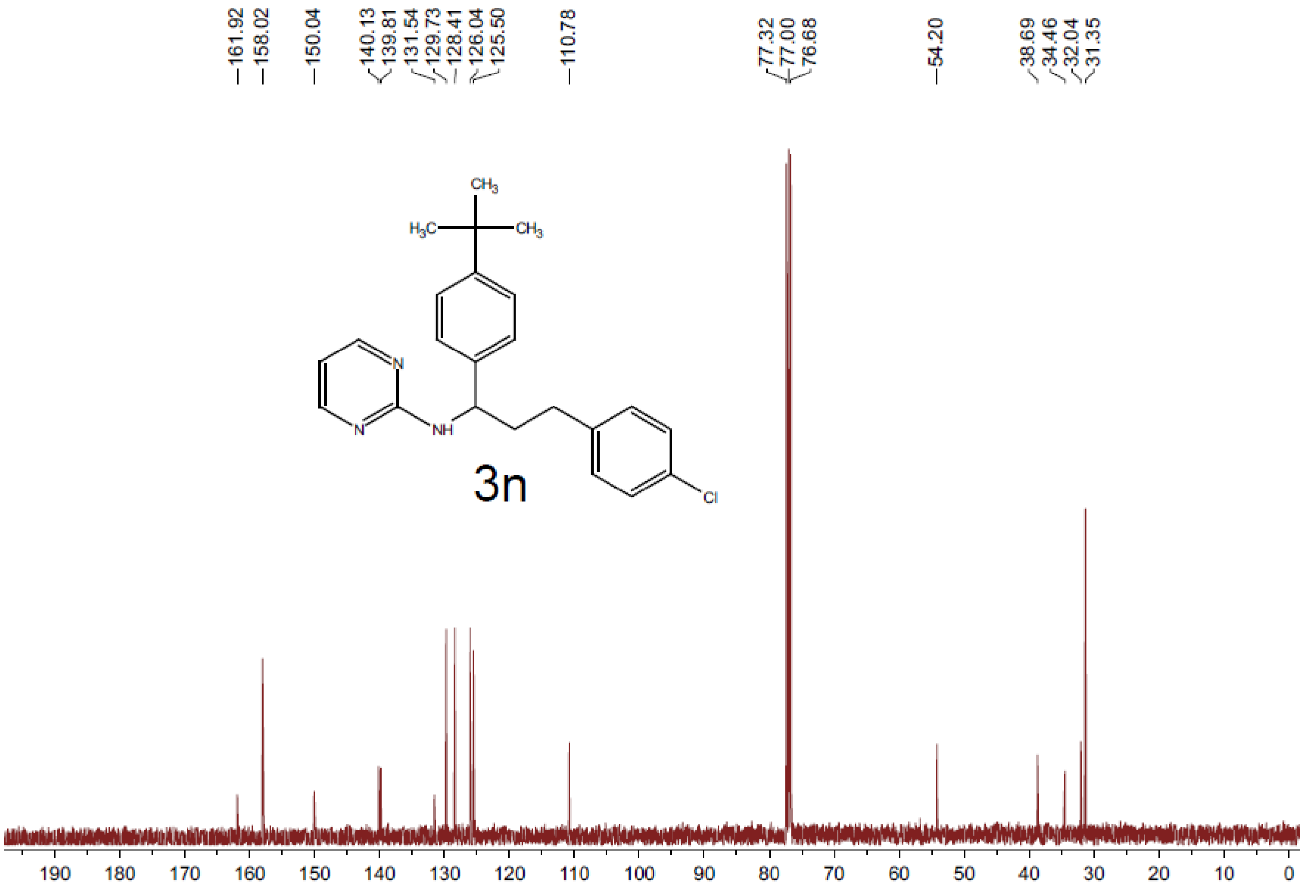
161.92
158.02
150.04
140.13
139.81
131.54
129.73
128.41
126.04
125.50
110.78
77.32
77.00
76.68
54.20
38.69
34.46
32.04
31.35
3n

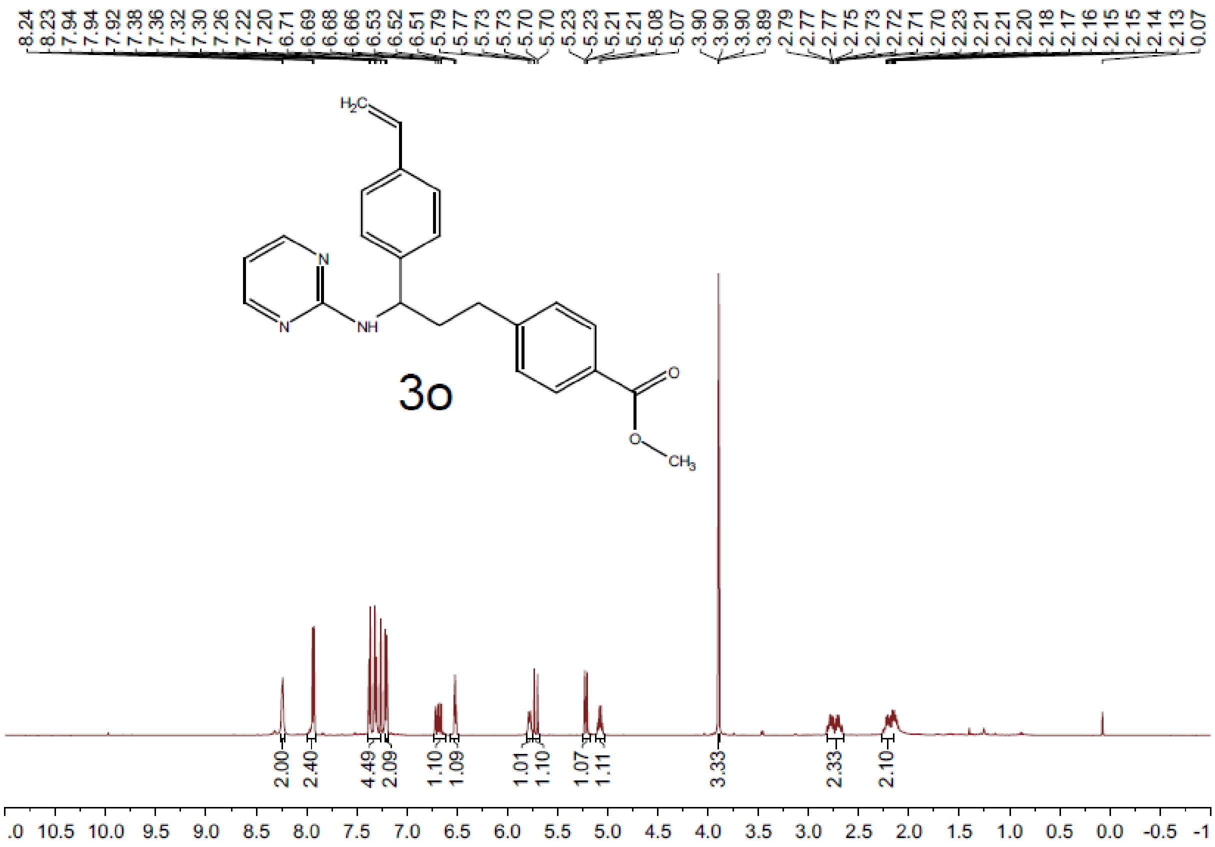
8.24 8.23 7.94 7.94 7.92 7.38 7.36 7.32 7.30 7.26 7.22 7.20 6.71 6.69 6.68 6.66 6.53 6.52 6.51 5.79 5.77 5.73 5.73 5.70 5.70 5.23 5.23 5.21 5.21 5.08 5.07 3.90 3.90 3.89 2.79 2.77 2.77 2.75 2.73 2.72 2.71 2.70 2.23 2.21 2.21 2.20 2.18 2.17 2.16 2.15 2.15 2.14 2.13 0.07
3o
2.00 2.40 4.49 2.09 1.10 1.09 1.01 1.10 1.07 1.11 3.33 2.33 2.10

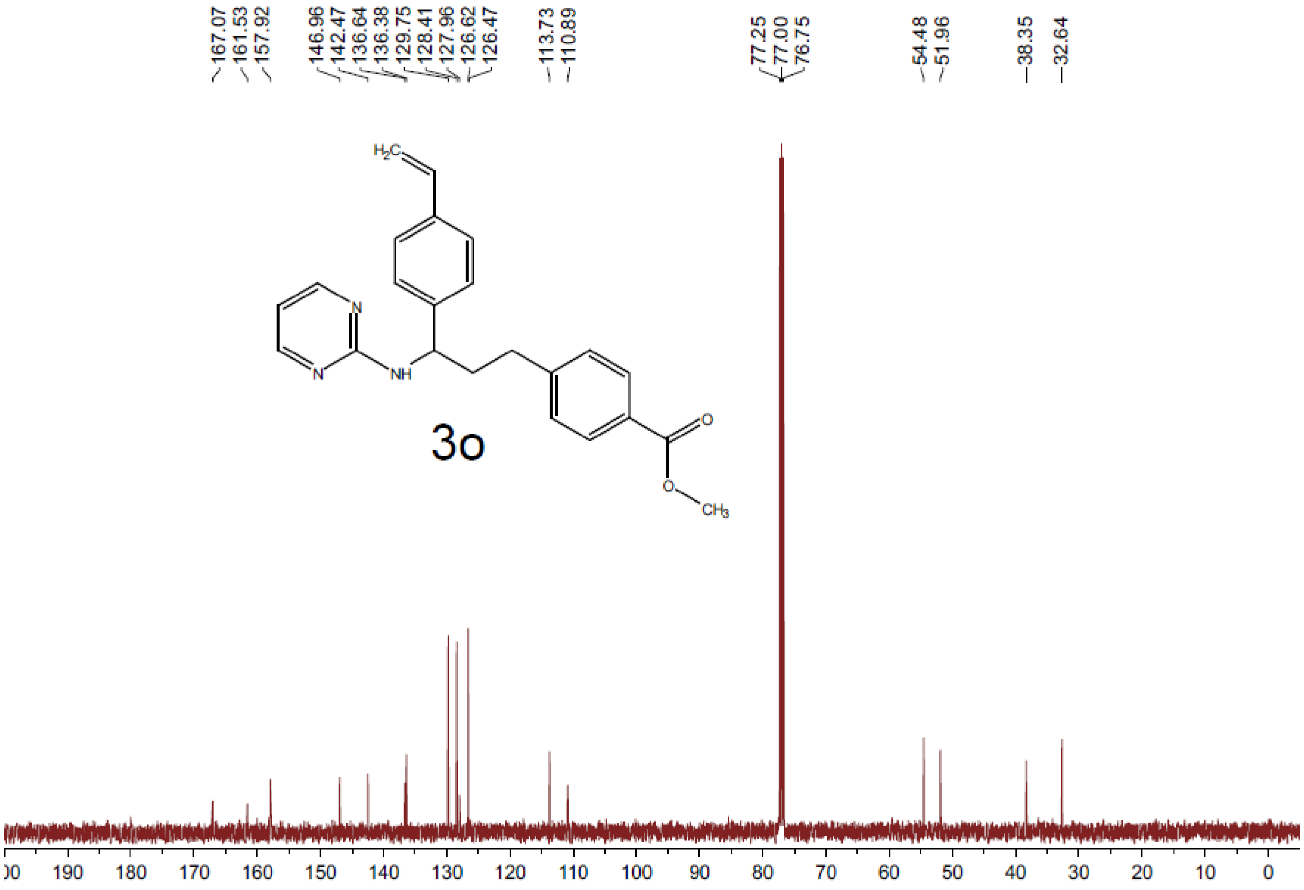
167.07
161.53
157.92
146.96
142.47
136.64
136.38
129.75
128.41
127.96
126.62
126.47
113.73
110.89
77.25
77.00
76.75
54.48
51.96
38.35
32.64
3o
190 180 170 160 150 140 130 120 110 100 90 80 70 60 50 40 30 20 10 0

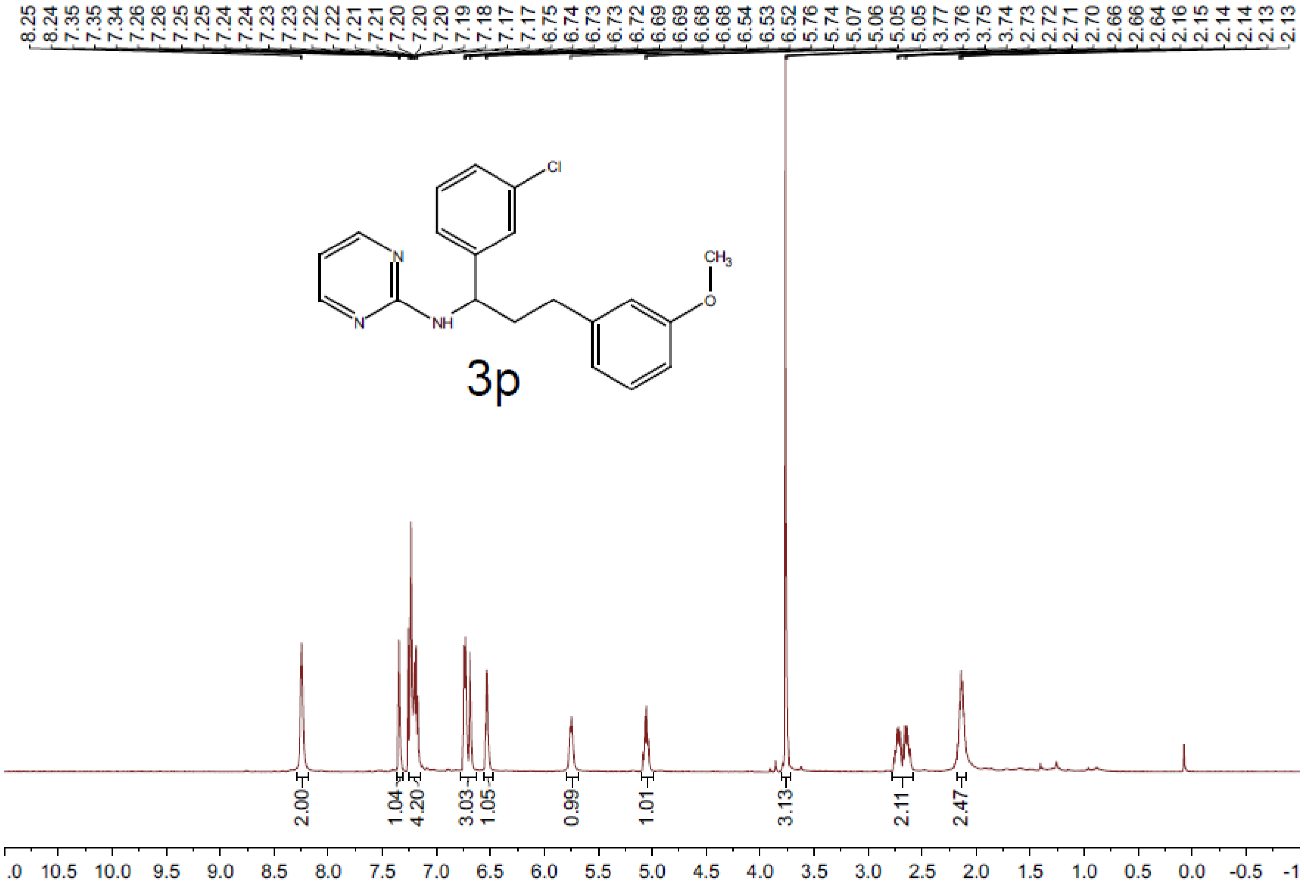
8.25 8.24 7.35 7.34 7.26 7.26 7.25 7.24 7.24 7.23 7.23 7.22 7.22 7.21 7.21 7.20 7.20 7.20 7.19 7.18 7.17 7.17 6.75 6.74 6.73 6.73 6.72 6.69 6.69 6.68 6.68 6.54 6.53 6.52 5.76 5.74 5.07 5.06 5.05 5.05 3.77 3.76 3.75 3.74 2.73 2.72 2.71 2.70 2.66 2.66 2.64 2.16 2.15 2.14 2.14 2.13 2.13
3p
2.00
1.04
4.20
3.03
1.05
0.99
1.01
3.13
2.11
2.47
10.5 10.0 9.5 9.0 8.5 8.0 7.5 7.0 6.5 6.0 5.5 5.0 4.5 4.0 3.5 3.0 2.5 2.0 1.5 1.0 0.5 0.0 -0.5

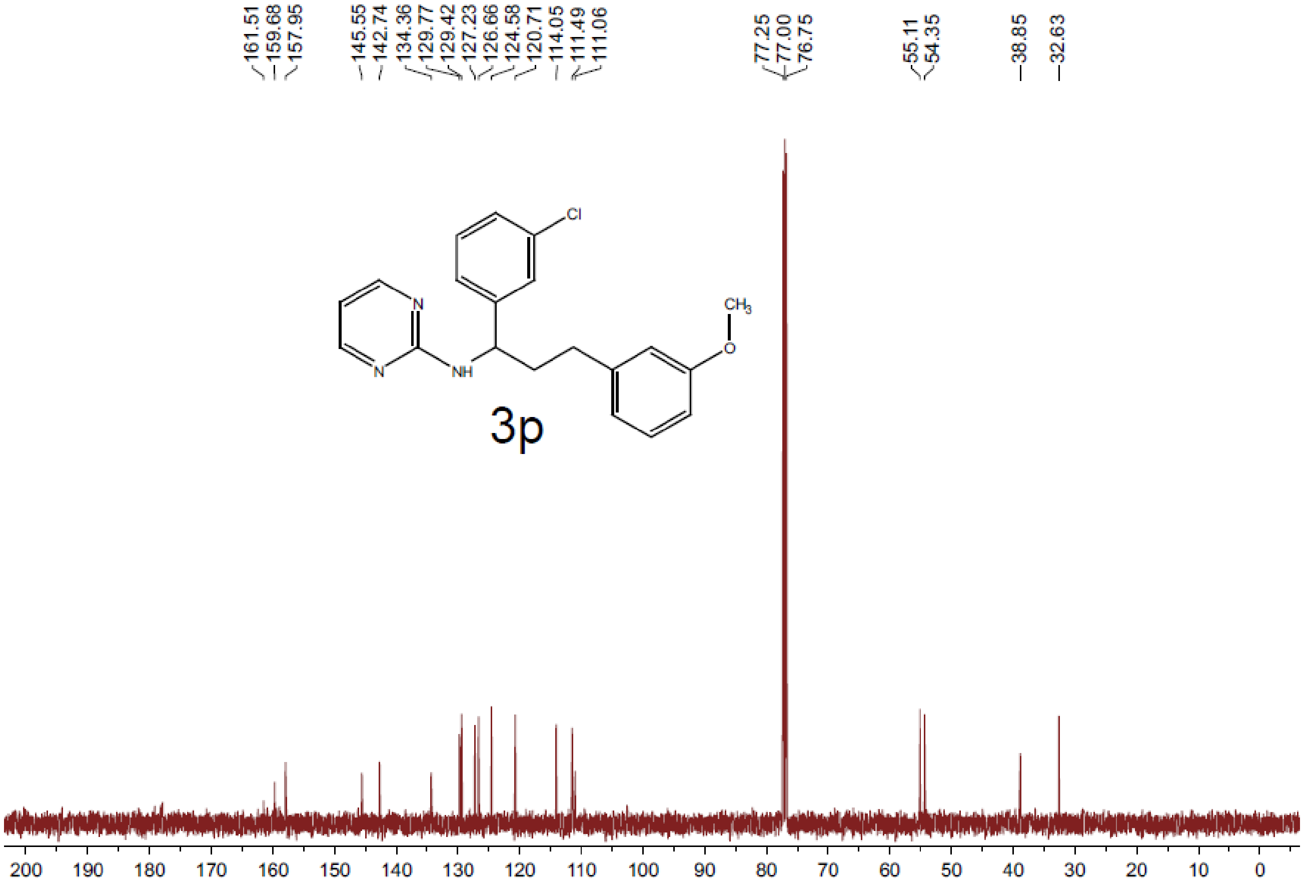
161.51
159.68
157.95
145.55
142.74
134.36
129.77
129.42
127.23
126.66
124.58
120.71
114.05
111.49
111.06
77.25
77.00
76.75
55.11
54.35
38.85
32.63
Cl
CH3
O
N
N
NH
3p
200 190 180 170 160 150 140 130 120 110 100 90 80 70 60 50 40 30 20 10 0

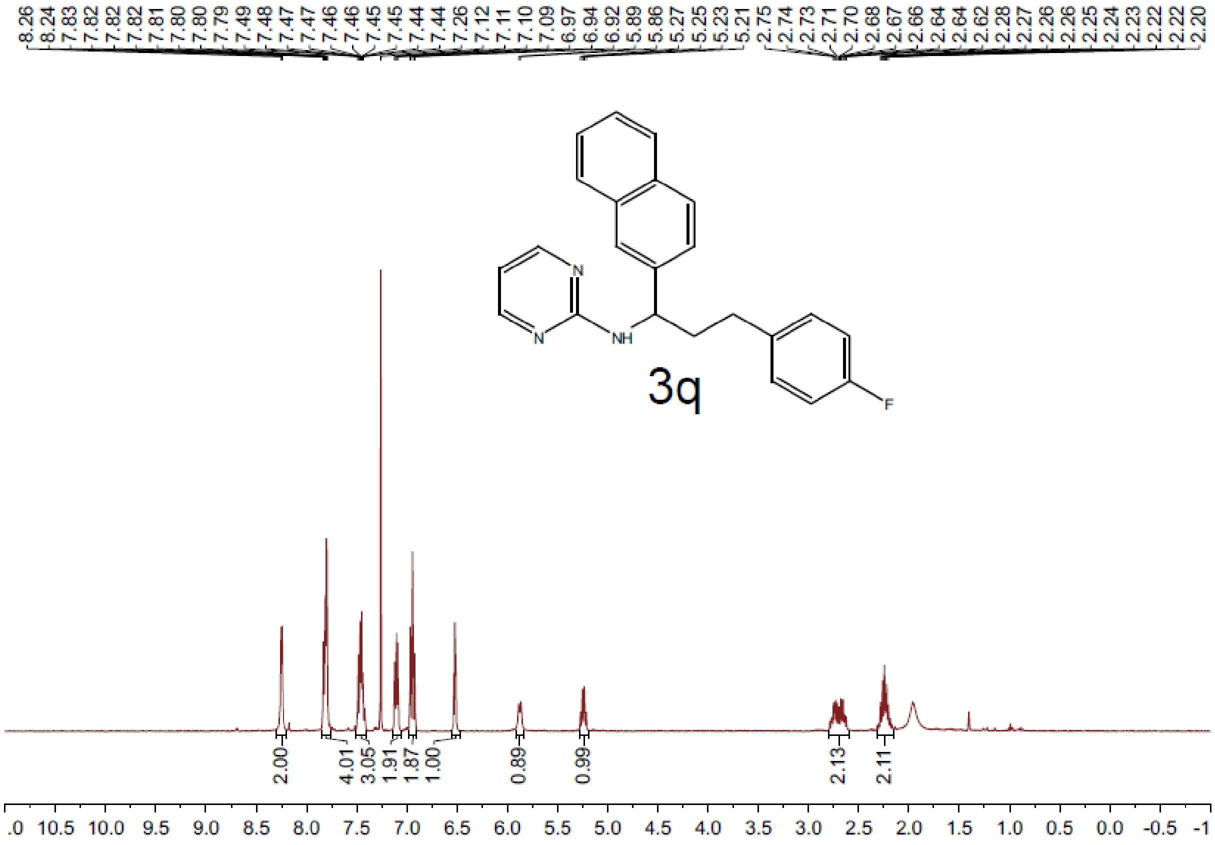
8.26
8.24
7.83
7.82
7.82
7.82
7.81
7.80
7.80
7.79
7.49
7.48
7.47
7.47
7.46
7.46
7.45
7.45
7.44
7.44
7.26
7.12
7.11
7.10
7.09
6.97
6.94
6.92
5.89
5.86
5.27
5.25
5.23
5.21
2.75
2.74
2.73
2.71
2.70
2.68
2.67
2.66
2.64
2.64
2.62
2.28
2.27
2.26
2.26
2.25
2.24
2.23
2.22
2.22
2.20
N
N
NH
F
3q
2.00
4.01
3.05
1.91
1.87
1.00
0.89
0.99
2.13
2.11
.0 10.5 10.0 9.5 9.0 8.5 8.0 7.5 7.0 6.5 6.0 5.5 5.0 4.5 4.0 3.5 3.0 2.5 2.0 1.5 1.0 0.5 0.0 -0.5 -1

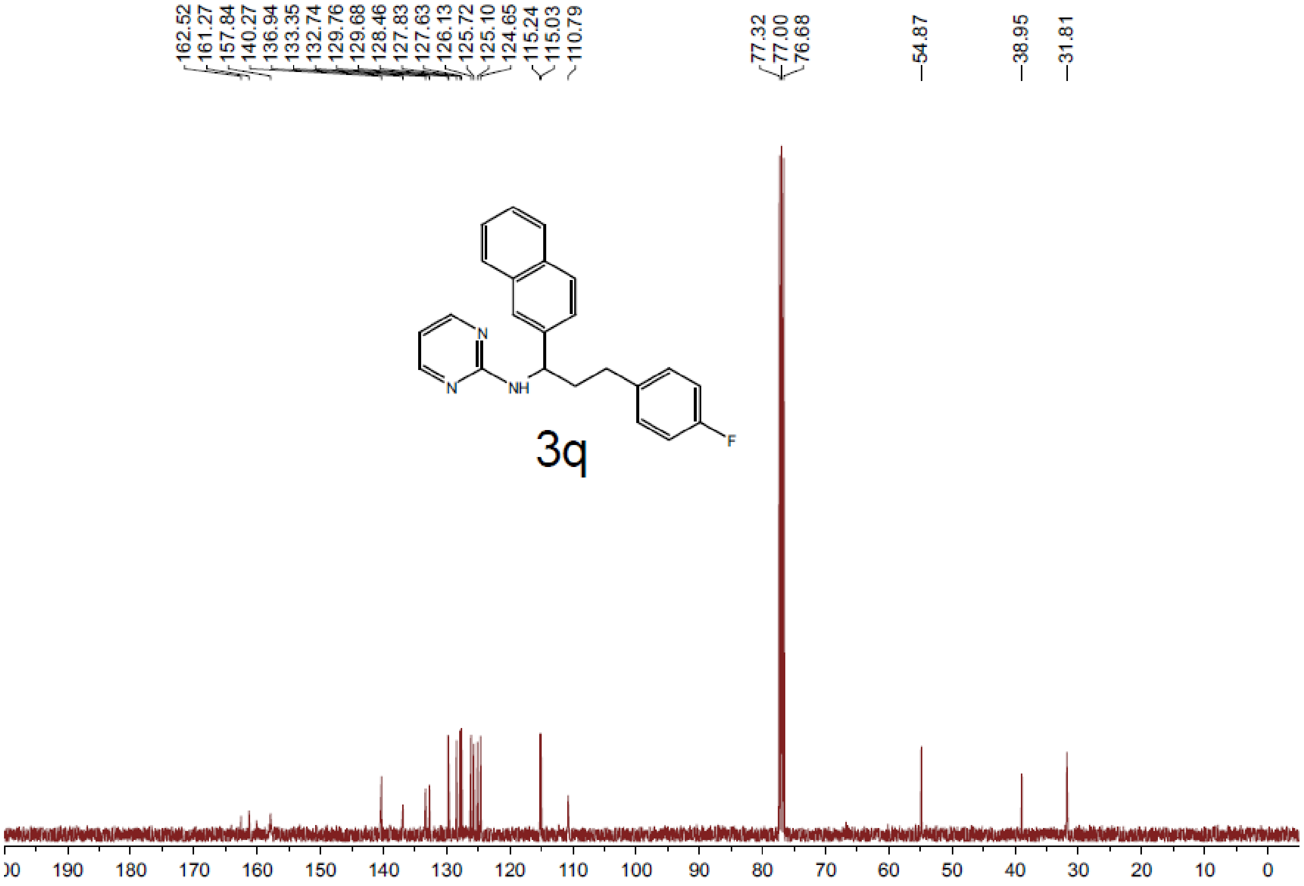
162.52
161.27
157.84
140.27
136.94
133.35
132.74
129.76
129.68
128.46
127.83
127.63
126.13
125.72
125.10
124.65
115.24
115.03
110.79
77.32
77.00
76.68
54.87
38.95
31.81
NH
N
F
3q

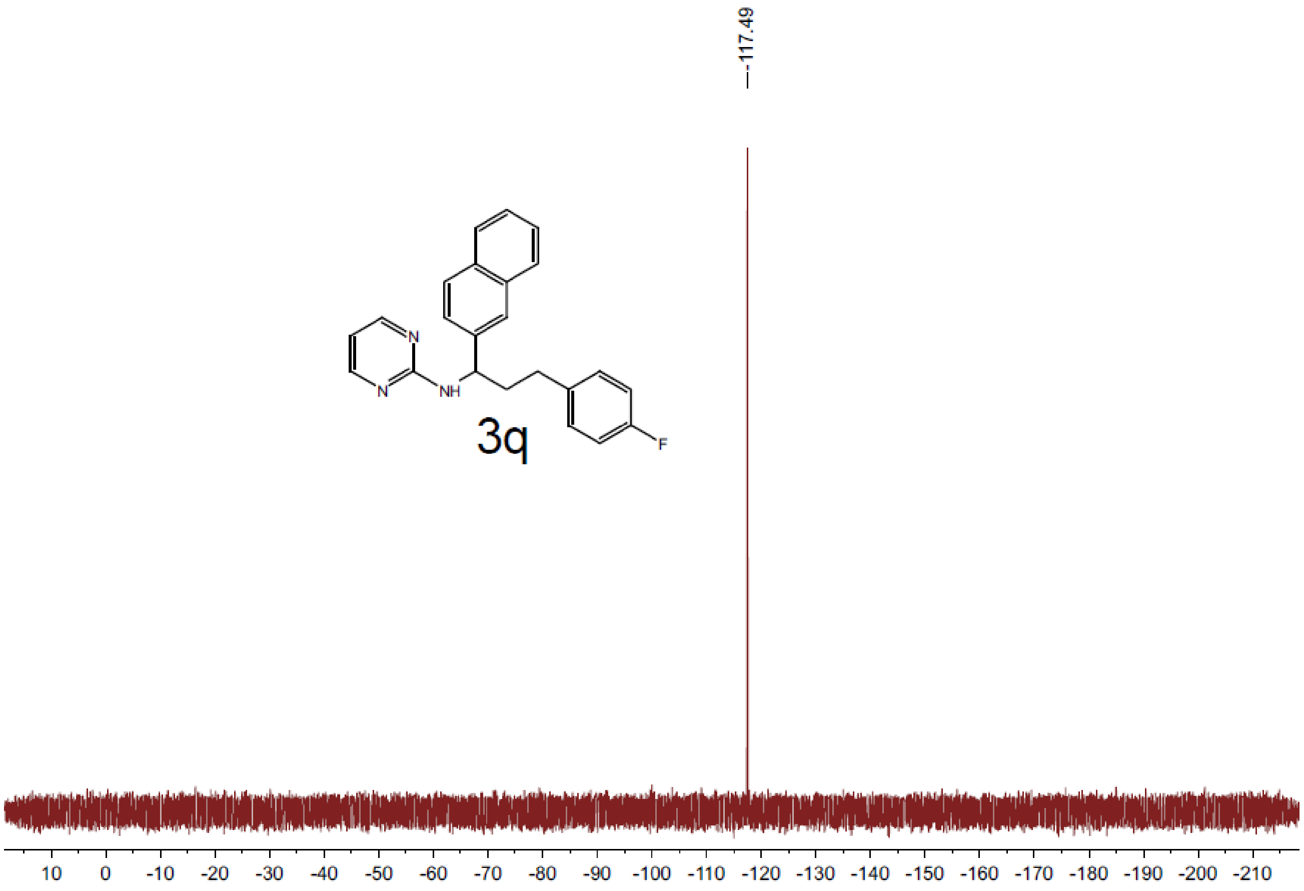
-117.49
NH
N
F
3q

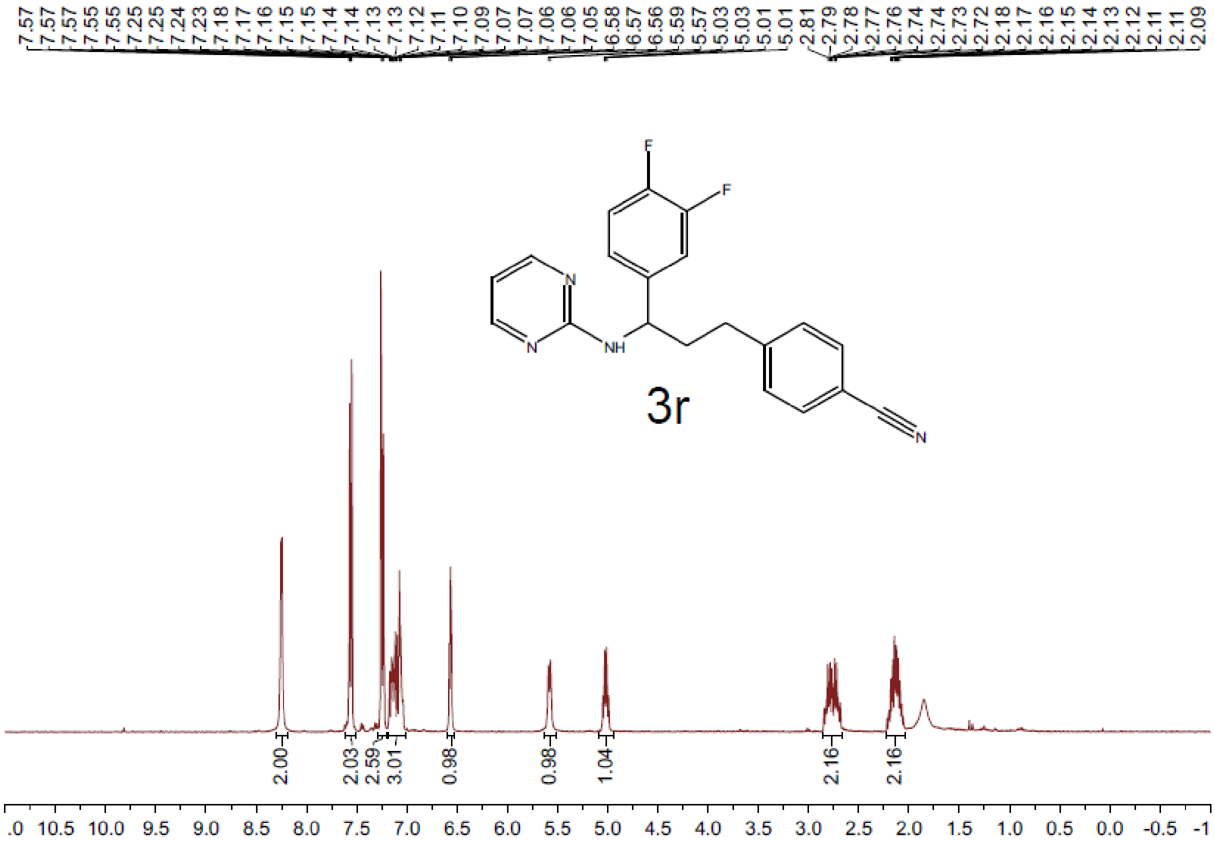

3r

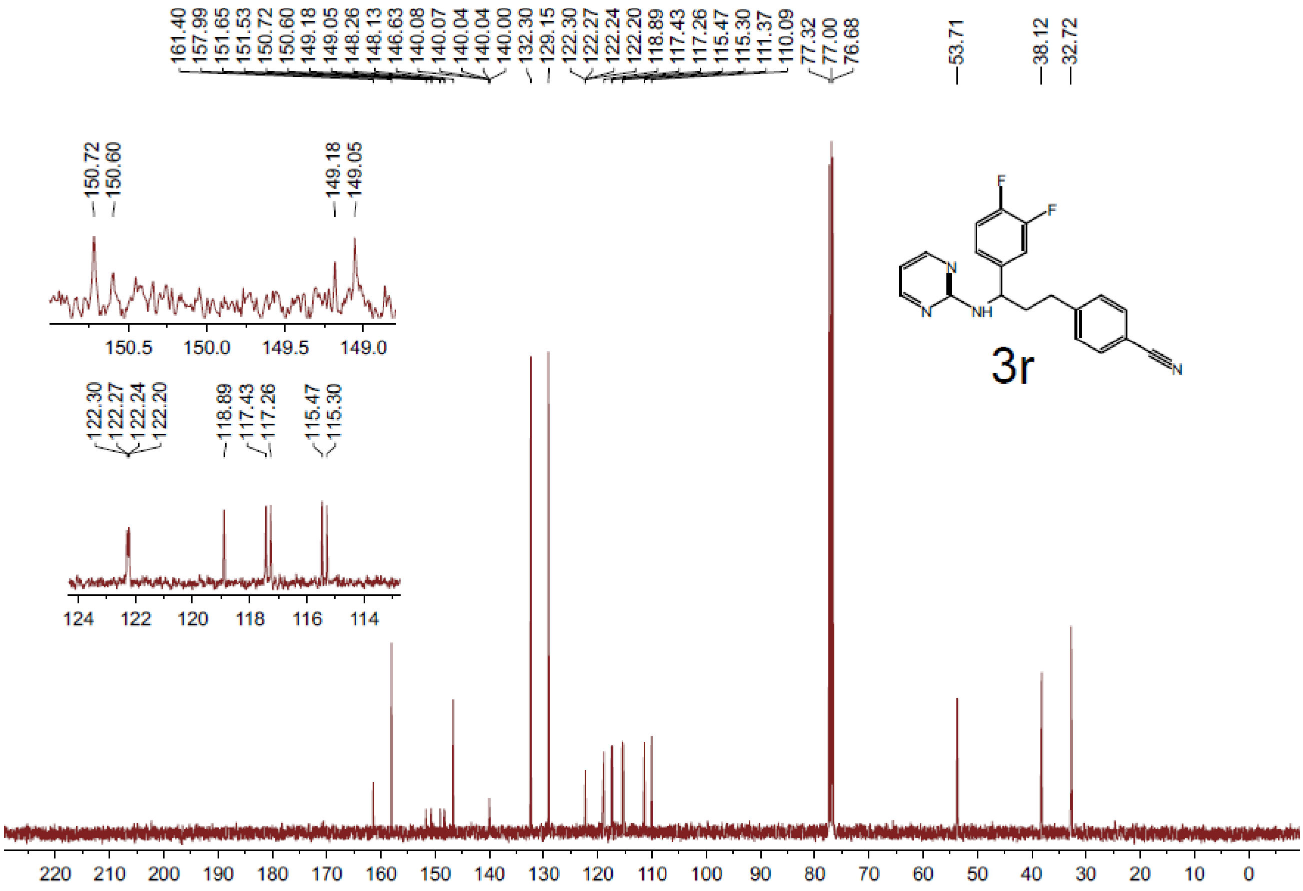

3r

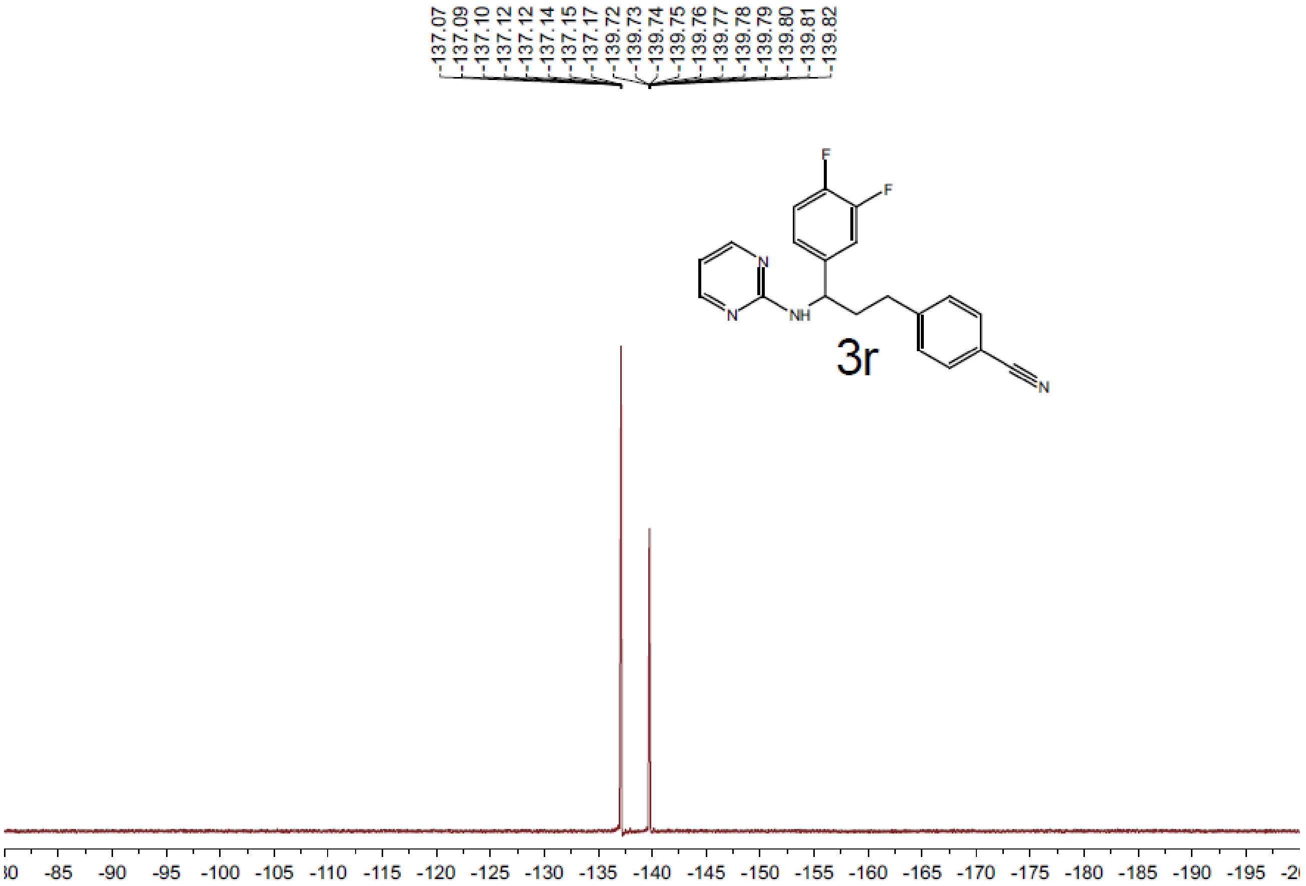
-137.07
-137.09
-137.10
-137.12
-137.12
-137.14
-137.15
-137.17
-139.72
-139.73
-139.74
-139.75
-139.76
-139.77
-139.78
-139.79
-139.80
-139.81
-139.82
F
F
N
N
NH
N
3r
-85 -90 -95 -100 -105 -110 -115 -120 -125 -130 -135 -140 -145 -150 -155 -160 -165 -170 -175 -180 -185 -190 -195

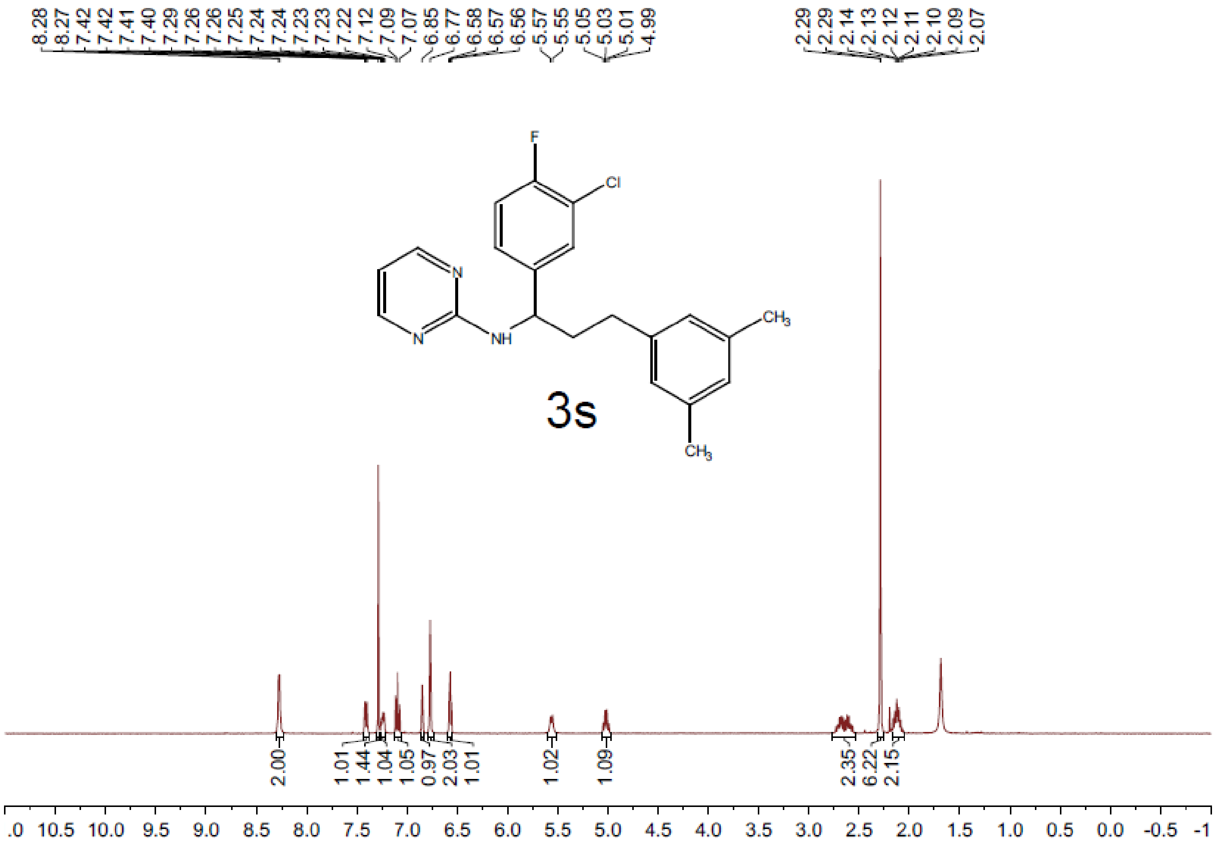
8.28
8.27
7.42
7.42
7.41
7.40
7.29
7.26
7.26
7.25
7.24
7.24
7.23
7.23
7.22
7.12
7.09
7.07
6.85
6.77
6.58
6.57
6.56
5.57
5.55
5.05
5.03
5.01
4.99
2.29
2.29
2.14
2.13
2.12
2.11
2.10
2.09
2.07
F
Cl
N
N
NH
CH3
CH3
3s
2.00
1.01
1.44
1.04
1.05
0.97
2.03
1.01
1.02
1.09
2.35
6.22
2.15
10.5 10.0 9.5 9.0 8.5 8.0 7.5 7.0 6.5 6.0 5.5 5.0 4.5 4.0 3.5 3.0 2.5 2.0 1.5 1.0 0.5 0.0 -0.5

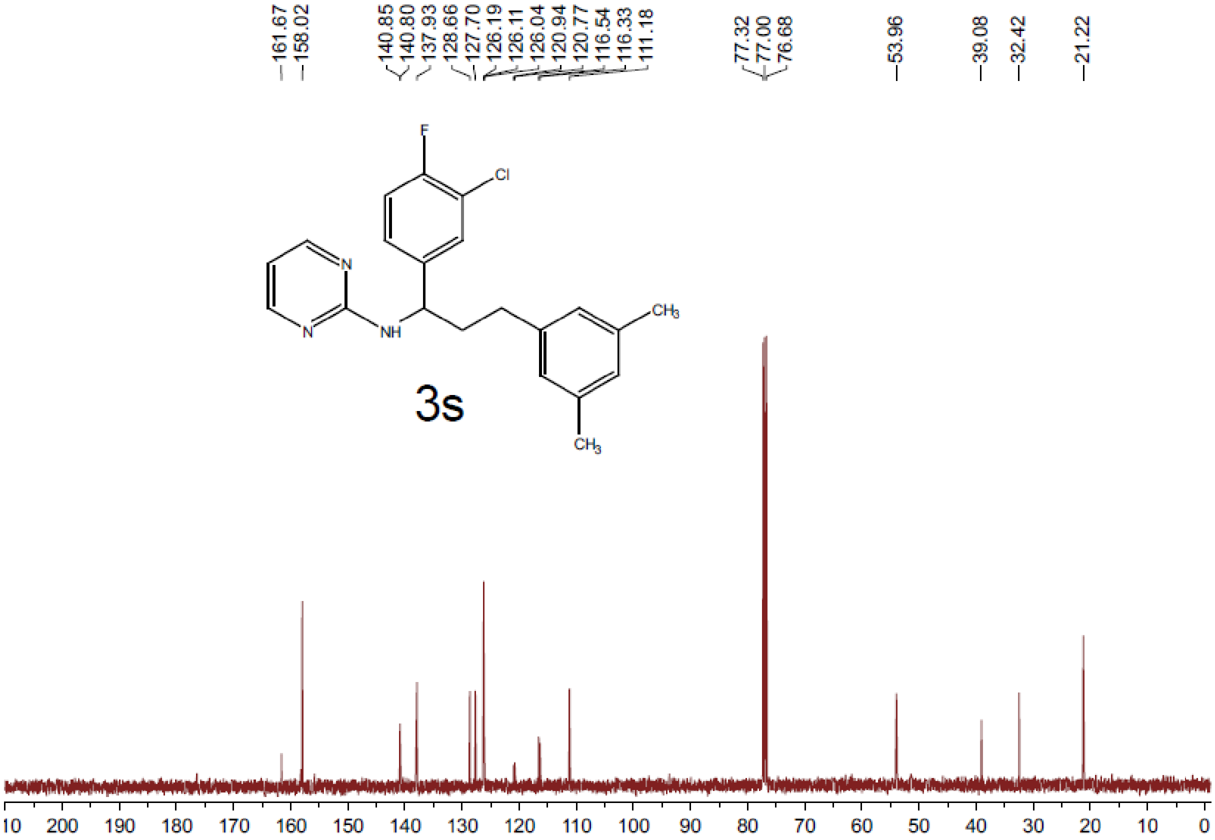
161.67
158.02
140.85
140.80
137.93
128.66
127.70
126.19
126.11
126.04
120.94
120.77
116.54
116.33
111.18
77.32
77.00
76.68
53.96
39.08
32.42
21.22
F
Cl
N
N
NH
CH3
CH3
3s
10 200 190 180 170 160 150 140 130 120 110 100 90 80 70 60 50 40 30 20 10 0

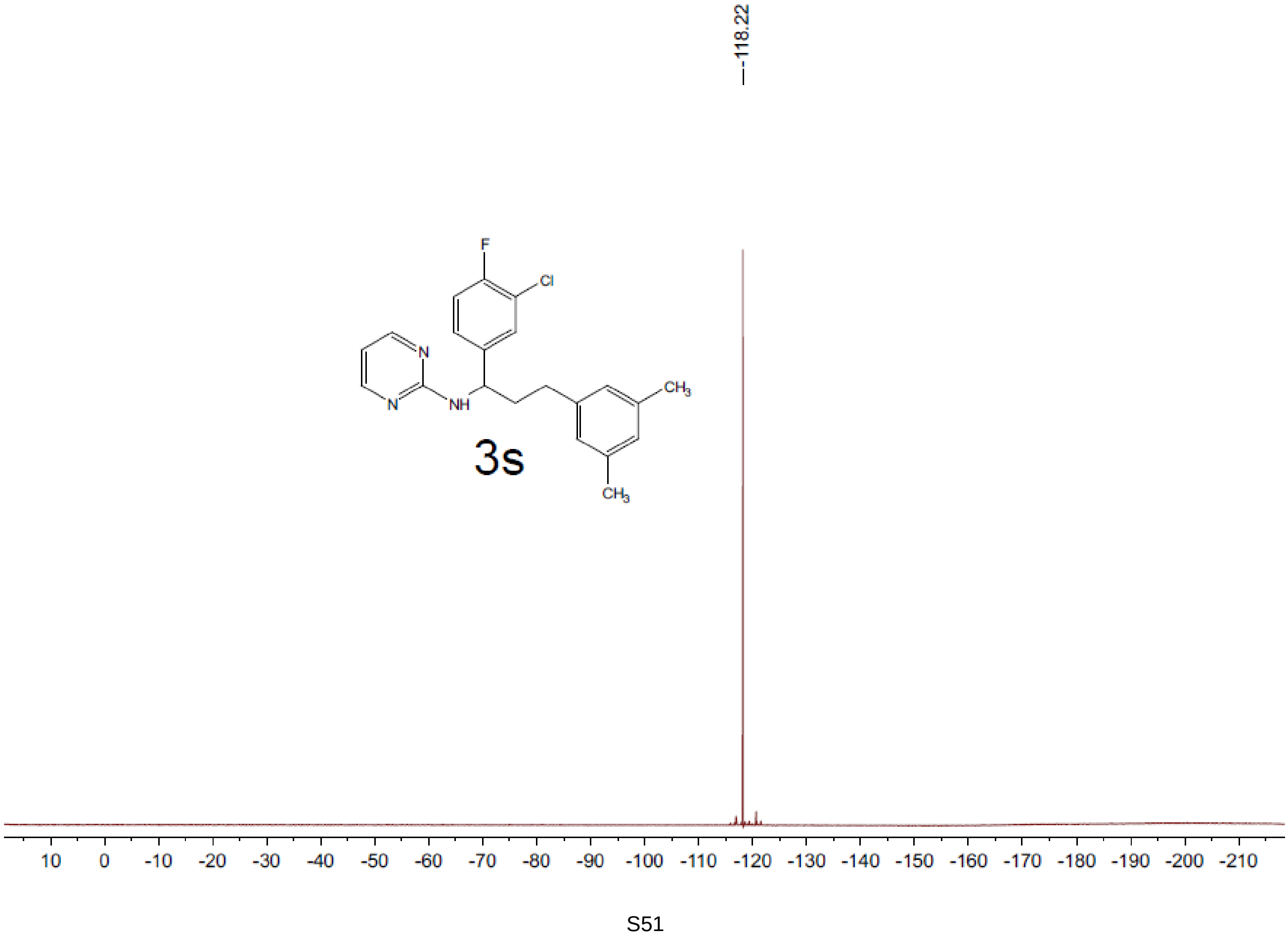
-118.22
F
Cl
N
N
NH
CH3
CH3
3s
10 0 -10 -20 -30 -40 -50 -60 -70 -80 -90 -100 -110 -120 -130 -140 -150 -160 -170 -180 -190 -200 -210

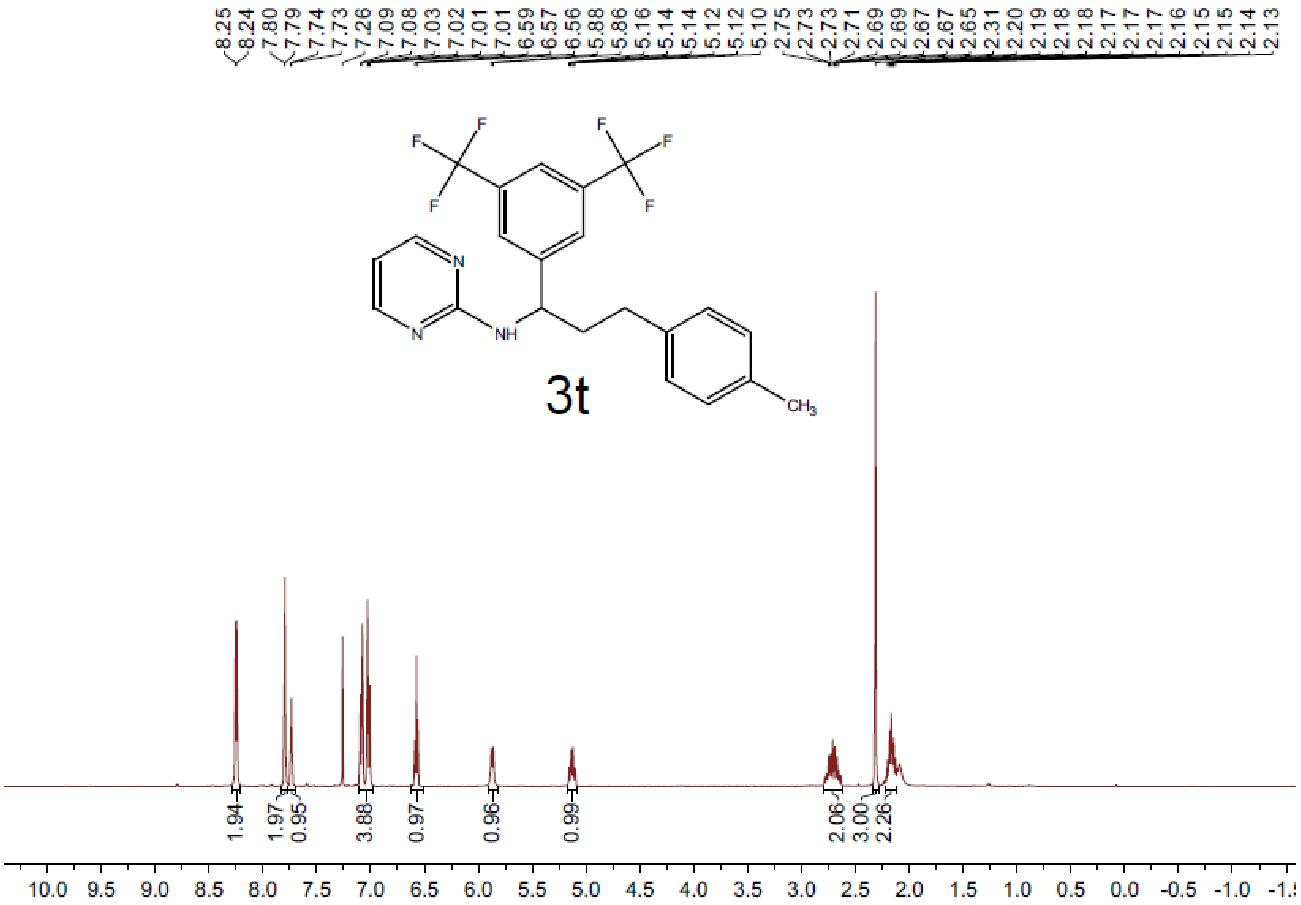
8.25
8.24
7.80
7.79
7.74
7.73
7.26
7.09
7.08
7.03
7.02
7.01
7.01
6.59
6.57
6.56
5.88
5.86
5.16
5.14
5.14
5.12
5.12
5.10
2.75
2.73
2.73
2.71
2.69
2.69
2.67
2.67
2.65
2.31
2.20
2.19
2.18
2.18
2.17
2.17
2.17
2.16
2.15
2.15
2.14
2.13
F
N
N
NH
CH3
3t
1.94
1.97
0.95
3.88
0.97
0.96
0.99
2.06
3.00
2.26
10.0
9.5
9.0
8.5
8.0
7.5
7.0
6.5
6.0
5.5
5.0
4.5
4.0
3.5
3.0
2.5
2.0
1.5
1.0
0.5
0.0
-0.5
-1.0

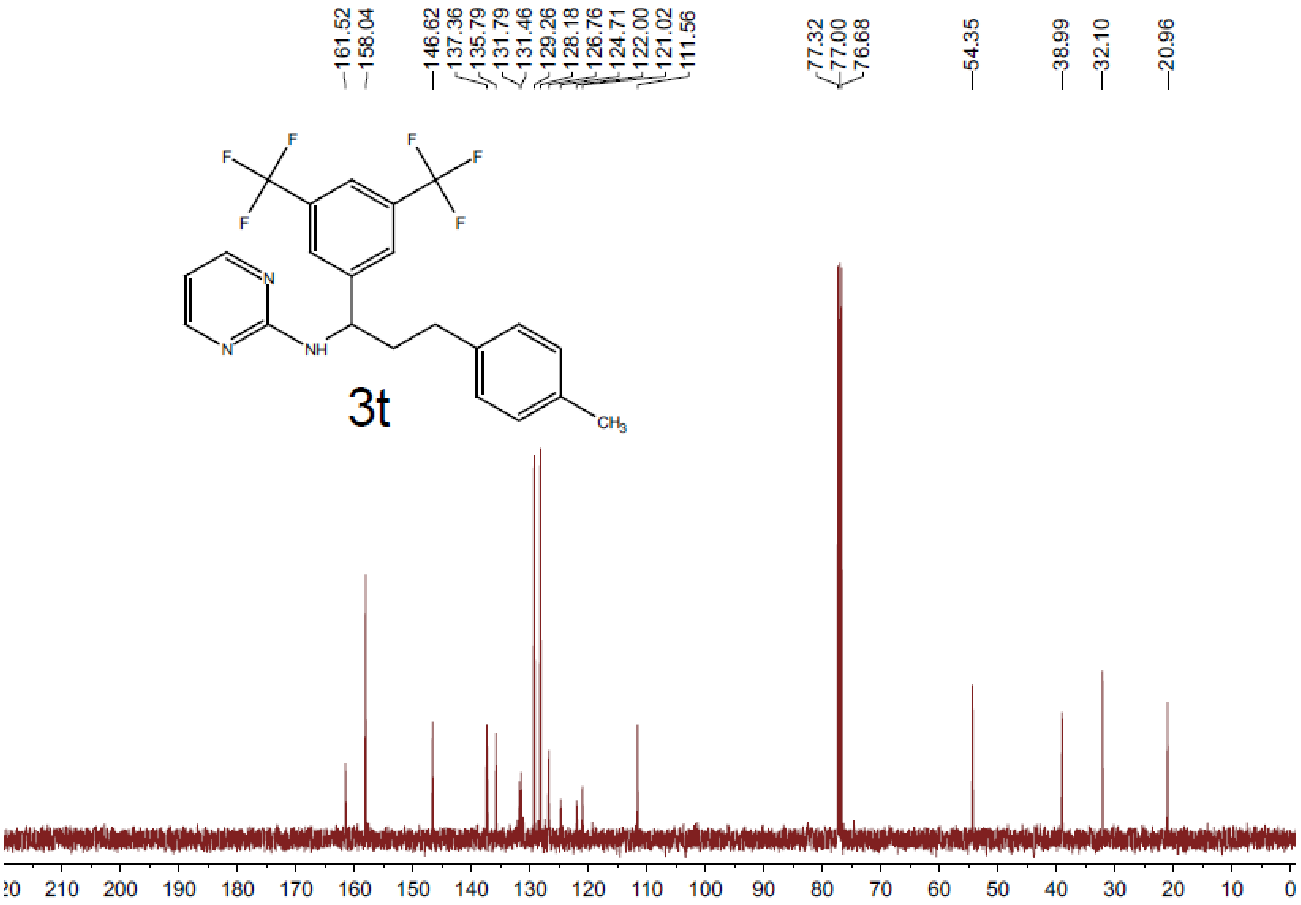
161.52
158.04
146.62
137.36
135.79
131.79
131.46
129.26
128.18
126.76
124.71
122.00
121.02
111.56
77.32
77.00
76.68
54.35
38.99
32.10
20.96
F
N
N
NH
CH3
3t
210
200
190
180
170
160
150
140
130
120
110
100
90
80
70
60
50
40
30
20
10
0

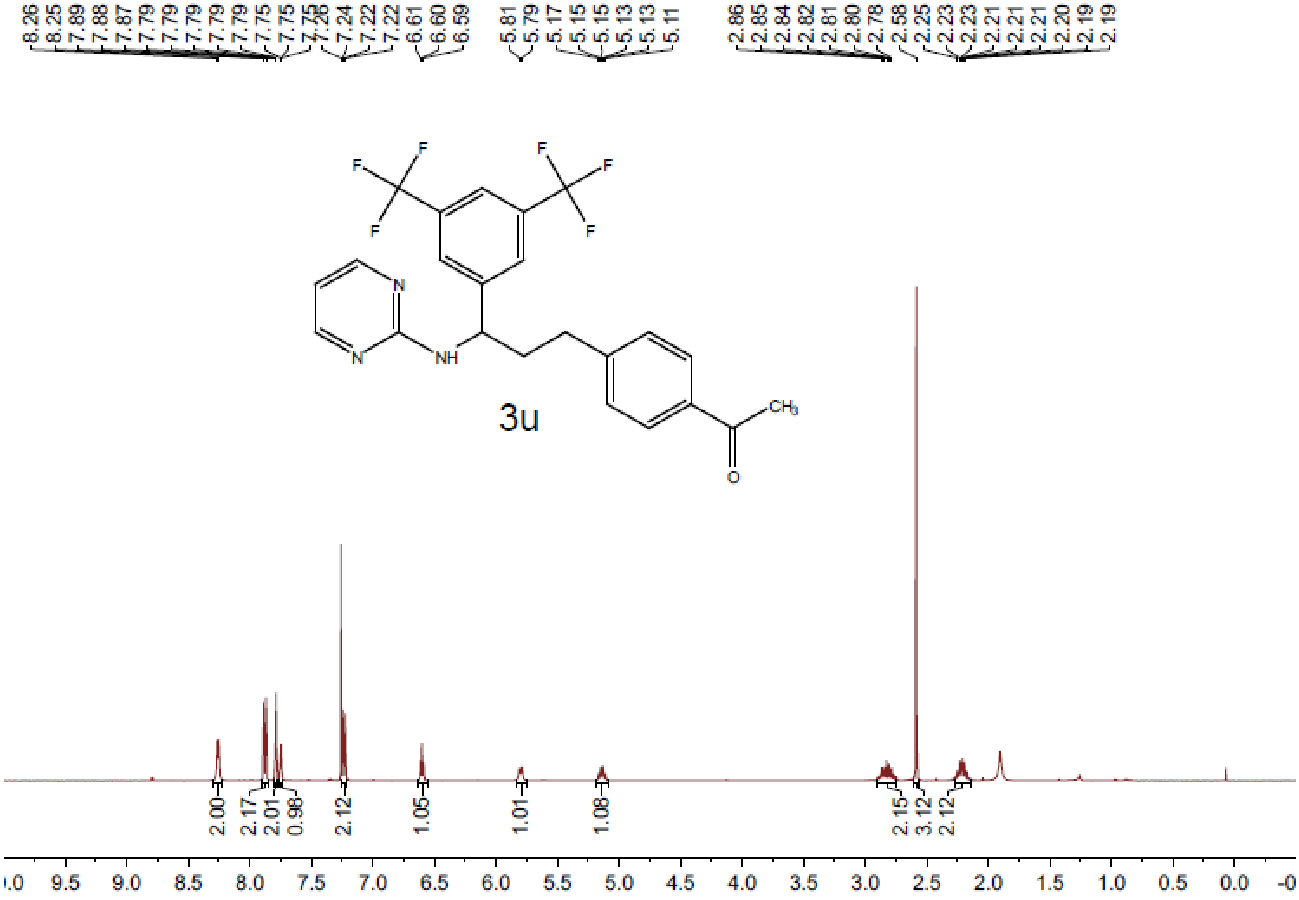
3u
CH3
NH
N
F
O

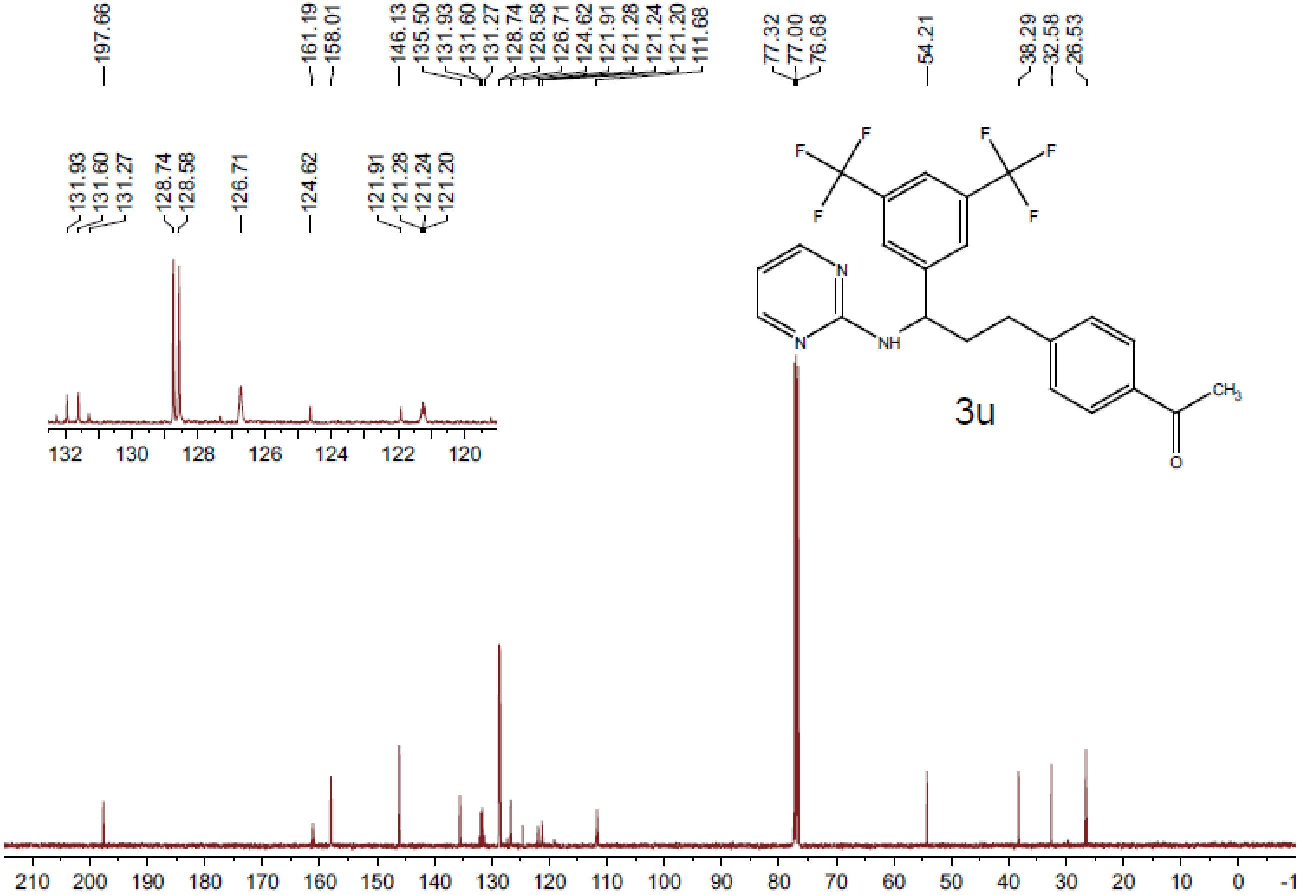
3u
CH3
NH
N
F
O

4a-c were contaminated with tiny amount of side products which can not be removed.

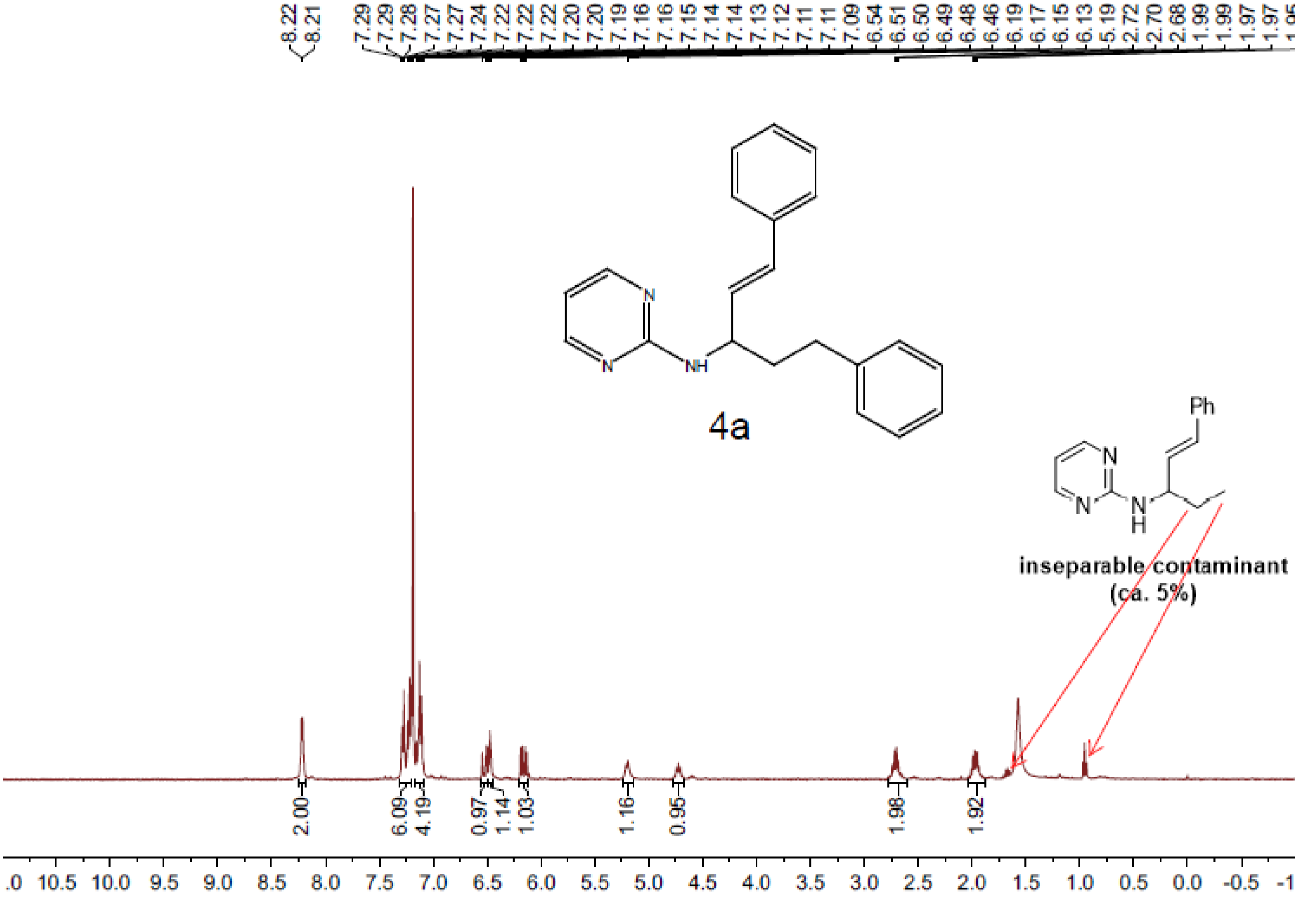

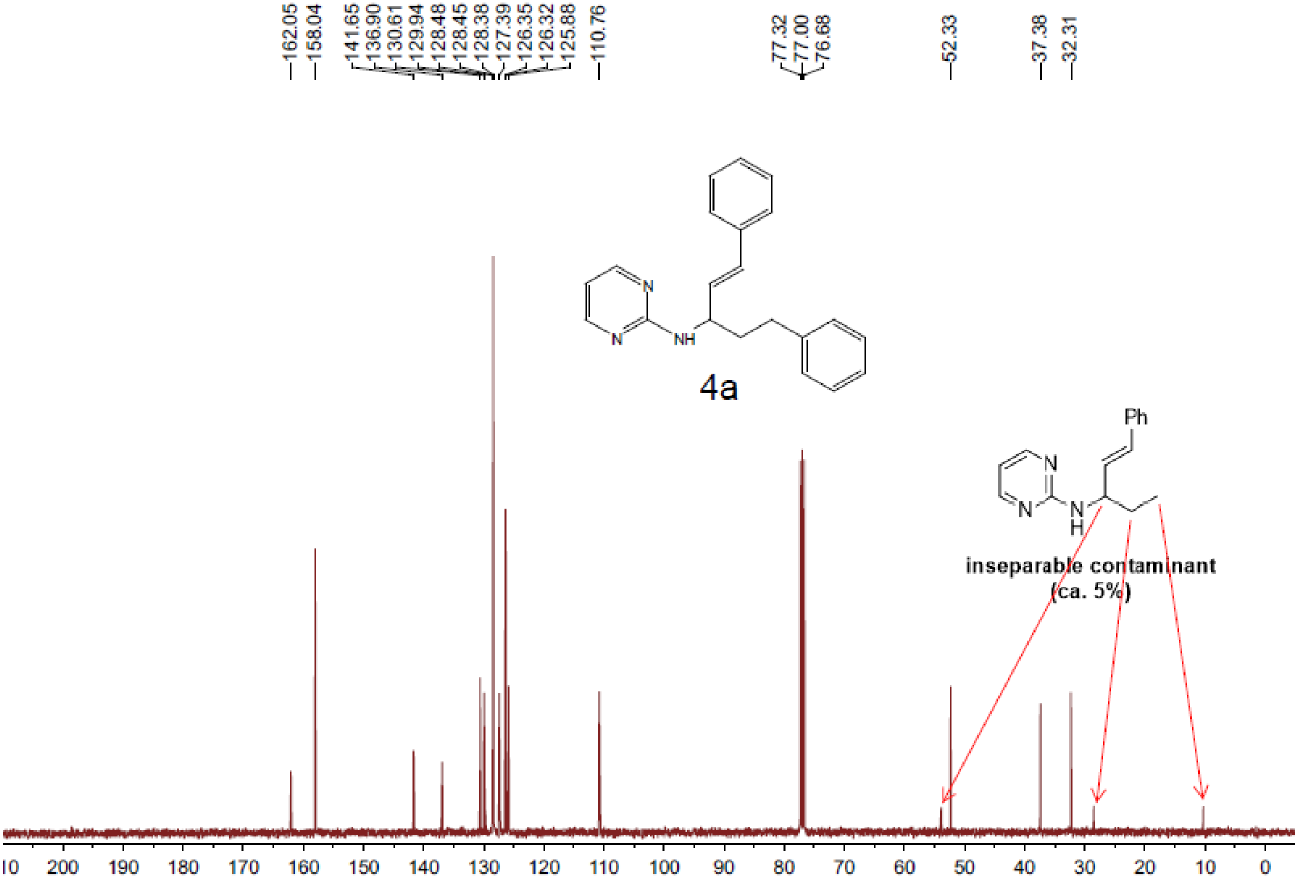

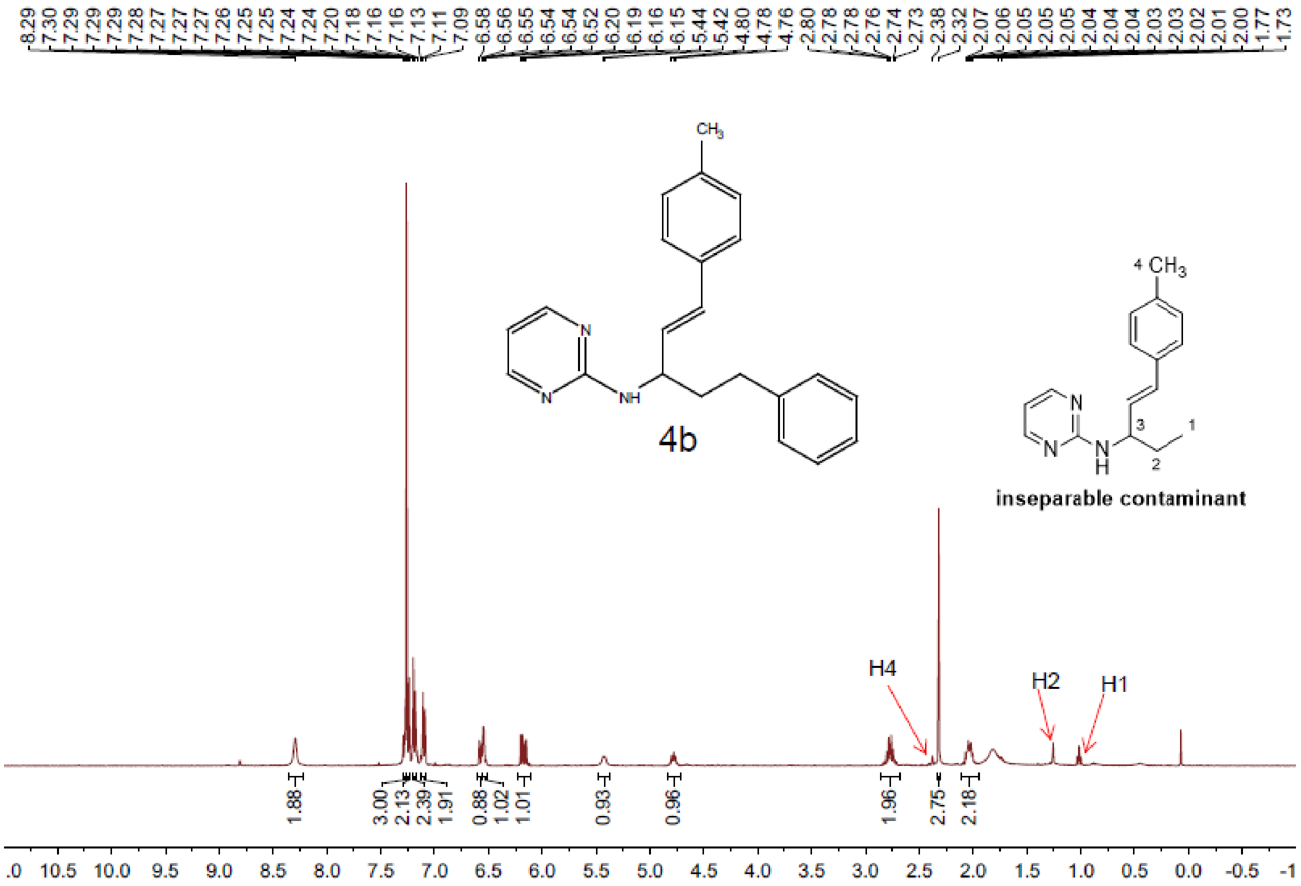

4b
inseparable contaminant
H4
H2
H1

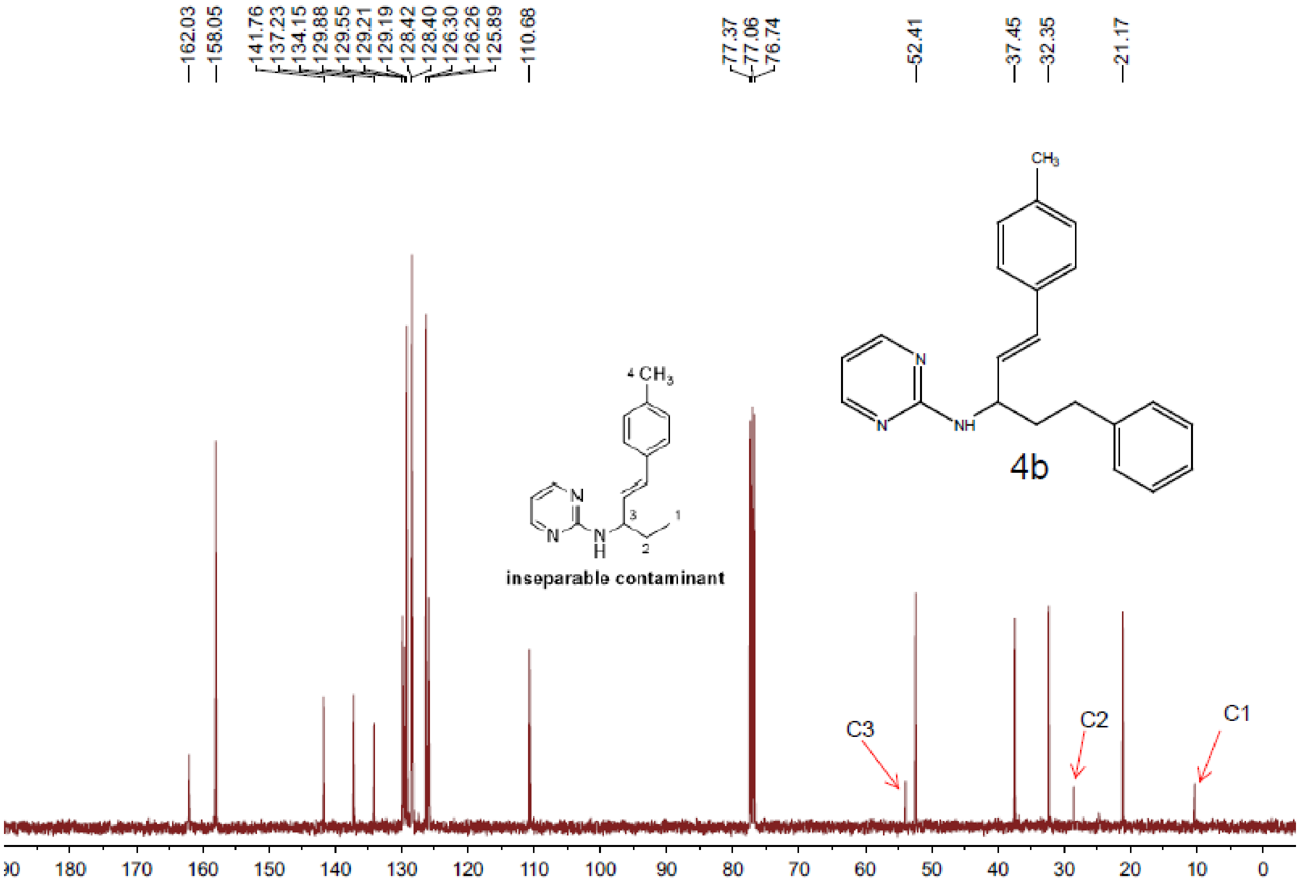

4b
inseparable contaminant
C3
C2
C1

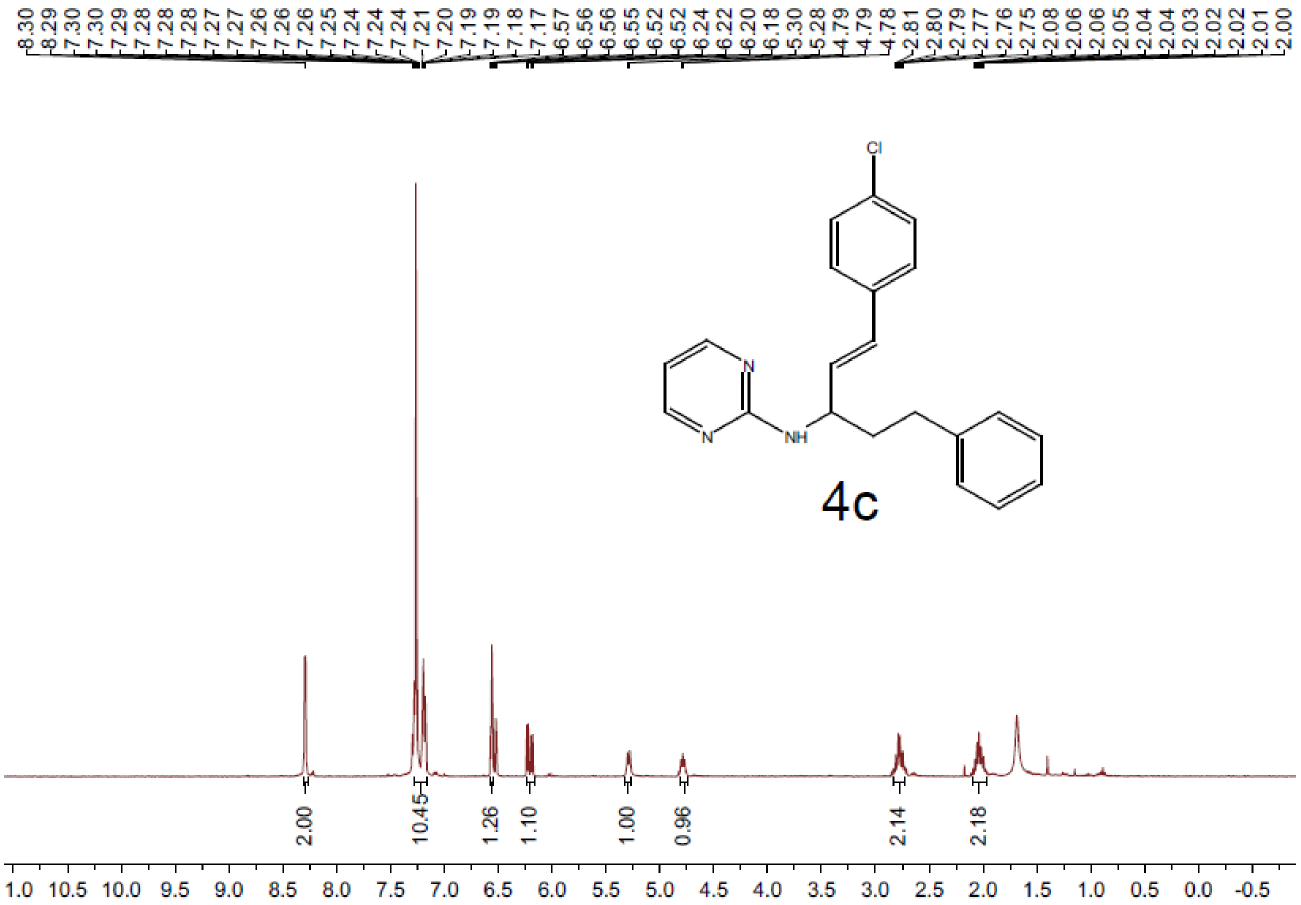
8.30 8.29 7.30 7.30 7.29 7.28 7.28 7.28 7.27 7.27 7.26 7.26 7.26 7.25 7.24 7.24 7.24 7.21 7.20 7.19 7.19 7.18 7.17 6.57 6.56 6.56 6.55 6.52 6.52 6.24 6.22 6.20 6.18 5.30 5.28 4.79 4.79 4.78 2.81 2.80 2.79 2.77 2.76 2.75 2.08 2.06 2.06 2.05 2.04 2.04 2.03 2.02 2.02 2.01 2.00
Cl
N
N
NH
4c
2.00 10.45 1.26 1.10 1.00 0.96 2.14 2.18
1.0 10.5 10.0 9.5 9.0 8.5 8.0 7.5 7.0 6.5 6.0 5.5 5.0 4.5 4.0 3.5 3.0 2.5 2.0 1.5 1.0 0.5 0.0 -0.5

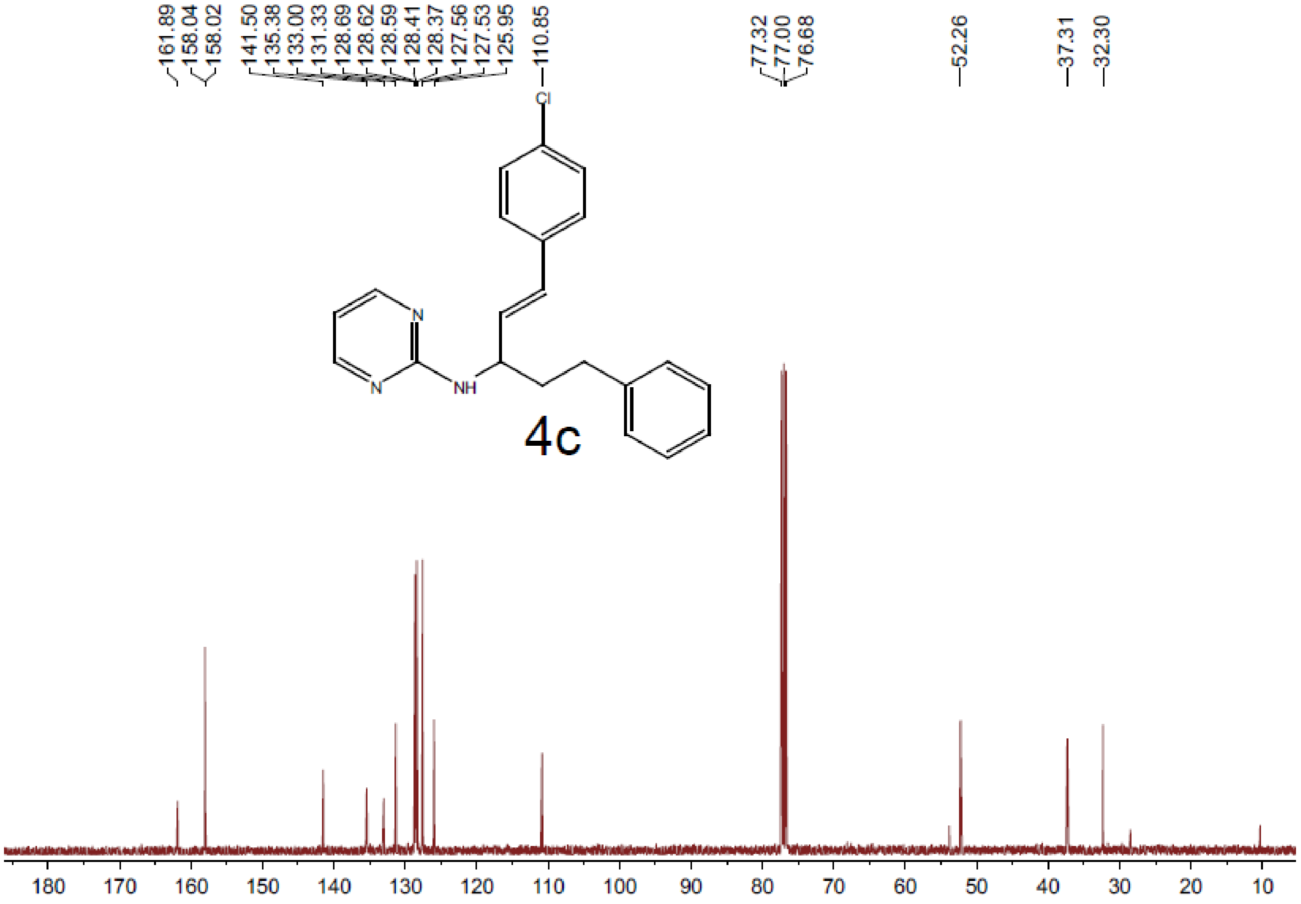
161.89 158.04 158.02 141.50 135.38 133.00 131.33 128.69 128.62 128.59 128.41 128.37 127.56 127.53 125.95 110.85 77.32 77.00 76.68 52.26 37.31 32.30
Cl
N
N
NH
4c
180 170 160 150 140 130 120 110 100 90 80 70 60 50 40 30 20 10

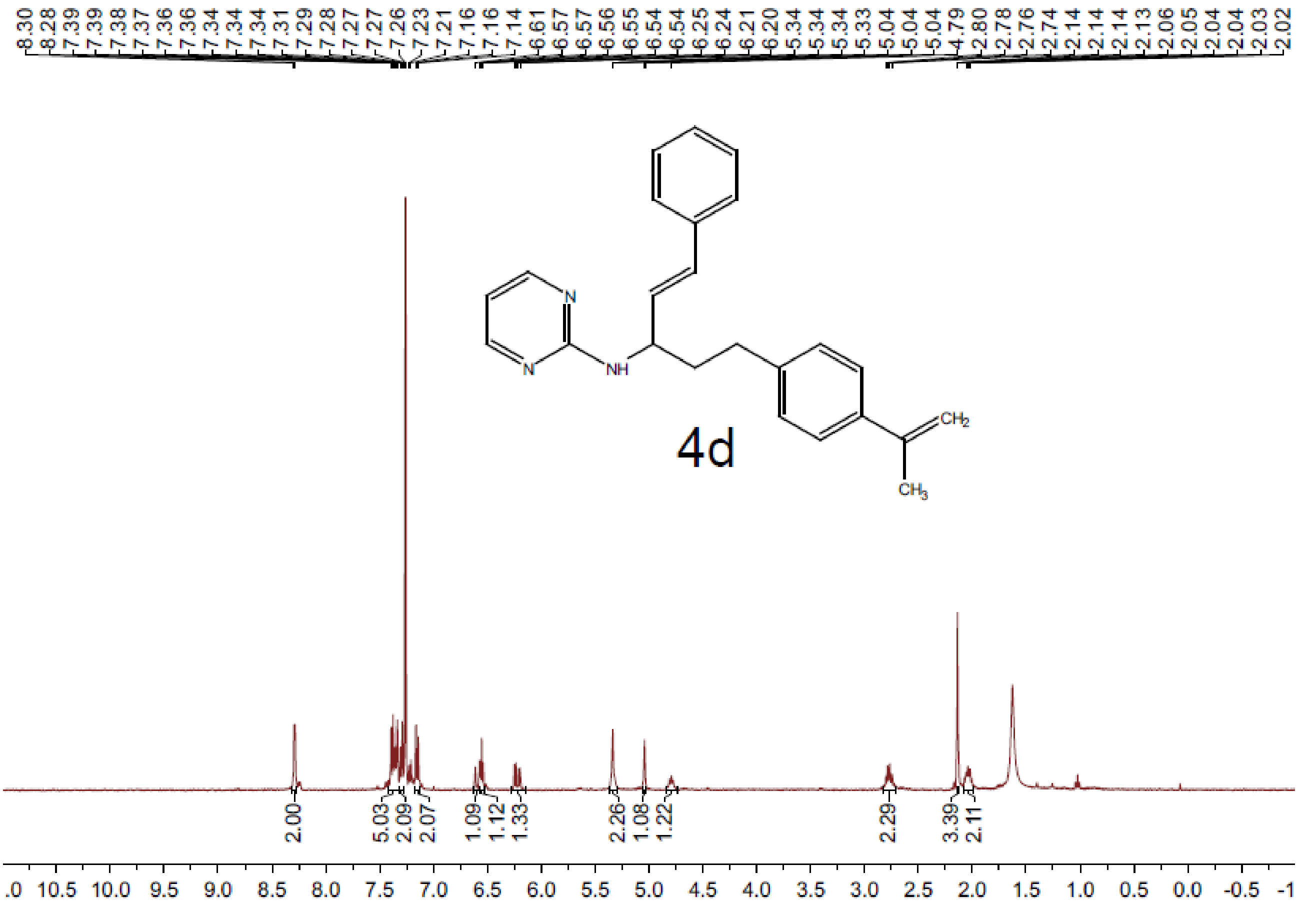
8.30 8.28 7.39 7.39 7.38 7.37 7.36 7.36 7.34 7.34 7.34 7.31 7.29 7.28 7.27 7.27 7.26 7.23 7.21 7.16 7.16 7.14 6.61 6.57 6.57 6.56 6.55 6.54 6.54 6.25 6.24 6.21 6.20 5.34 5.34 5.34 5.33 5.04 5.04 5.04 4.79 2.80 2.78 2.76 2.74 2.14 2.14 2.14 2.13 2.06 2.05 2.04 2.04 2.04 2.03 2.02
N
N
NH
CH2
CH3
4d
2.00
5.03
2.09
2.07
1.09
1.12
1.33
2.26
1.08
1.22
2.29
3.39
2.11
.0 10.5 10.0 9.5 9.0 8.5 8.0 7.5 7.0 6.5 6.0 5.5 5.0 4.5 4.0 3.5 3.0 2.5 2.0 1.5 1.0 0.5 0.0 -0.5 -1

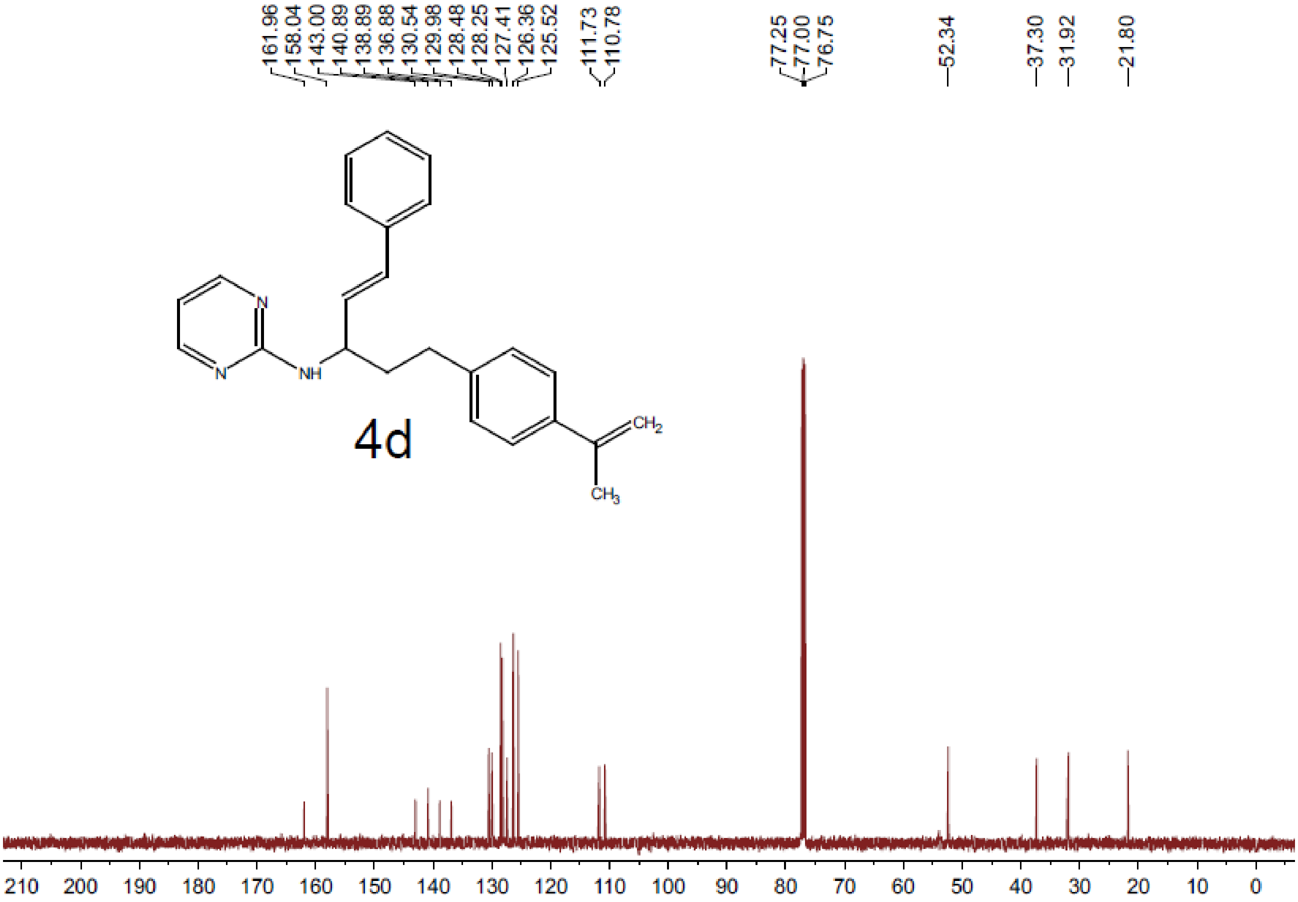
161.96 158.04 143.00 140.89 138.89 136.88 130.54 129.98 128.48 128.25 127.41 126.36 125.52 111.73 110.78
77.25 77.00 76.75
52.34
37.30 31.92
21.80
N
N
NH
CH2
CH3
4d
210 200 190 180 170 160 150 140 130 120 110 100 90 80 70 60 50 40 30 20 10 0

4e seemed to be not very stable and NMR must be taken as soon as possible. Longer waiting time would result in undesired peaks caused by degradation of the structure.

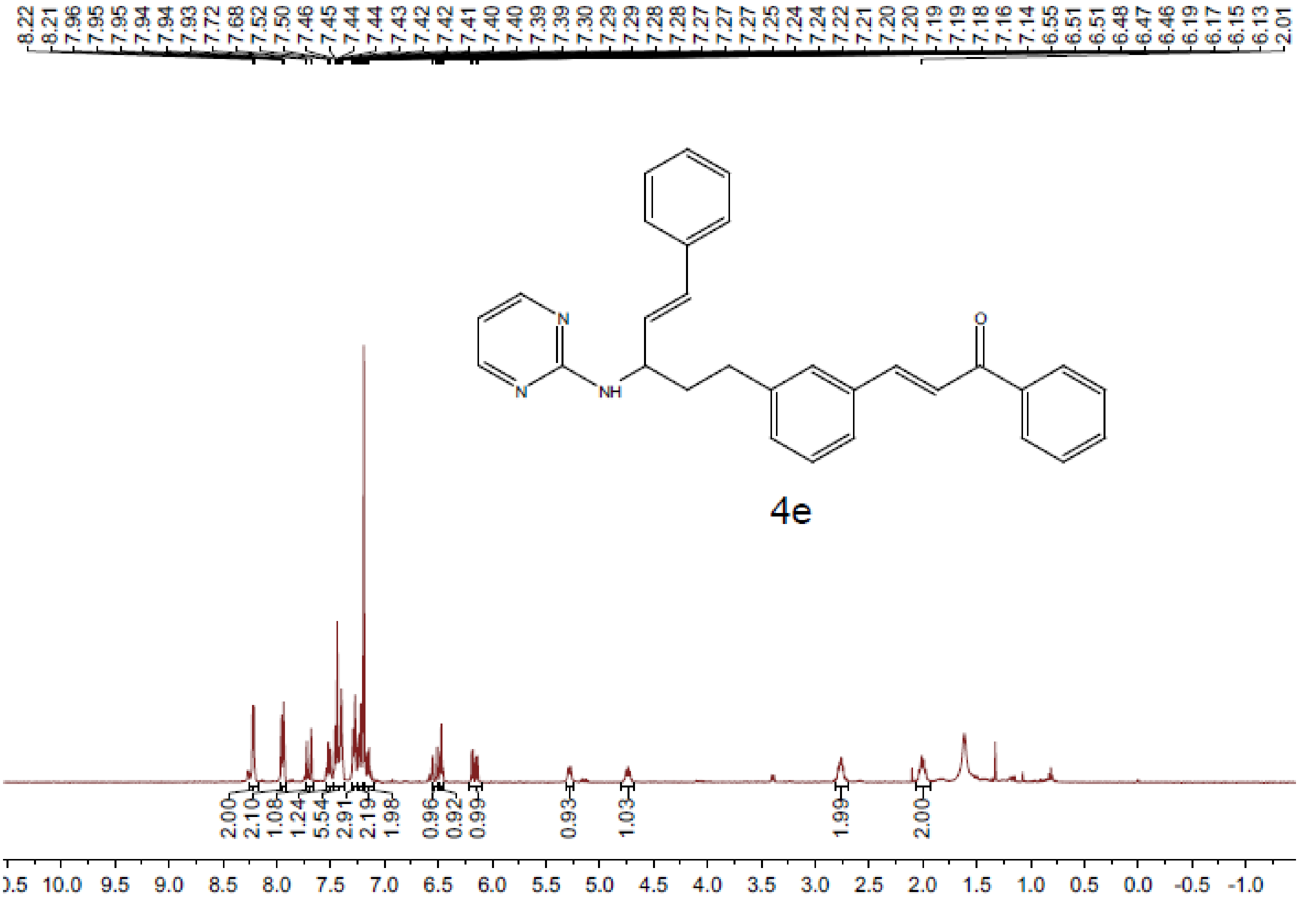

The following is the ^{1}H NMR spectrum for the freshly purified sample of 4e.

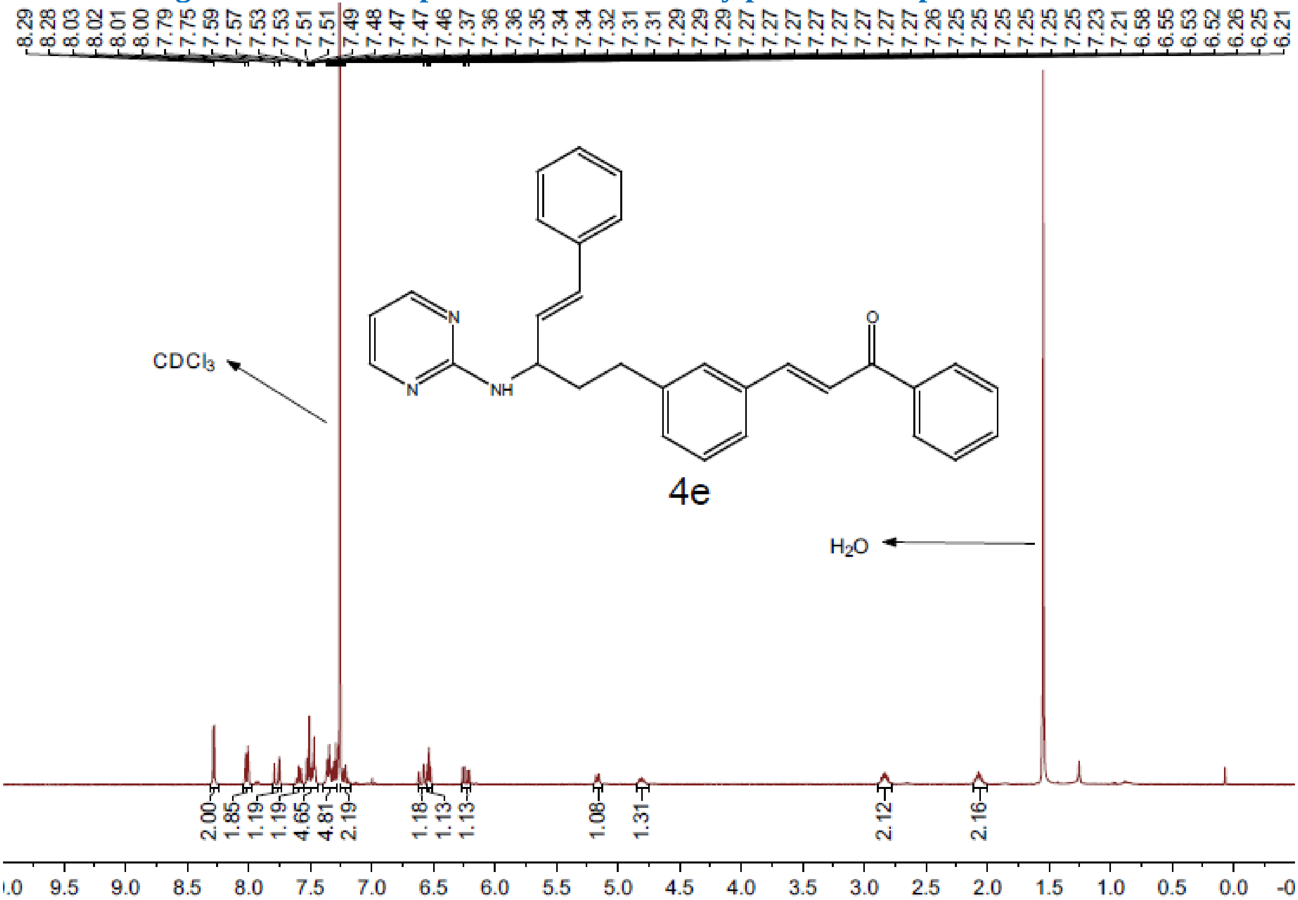

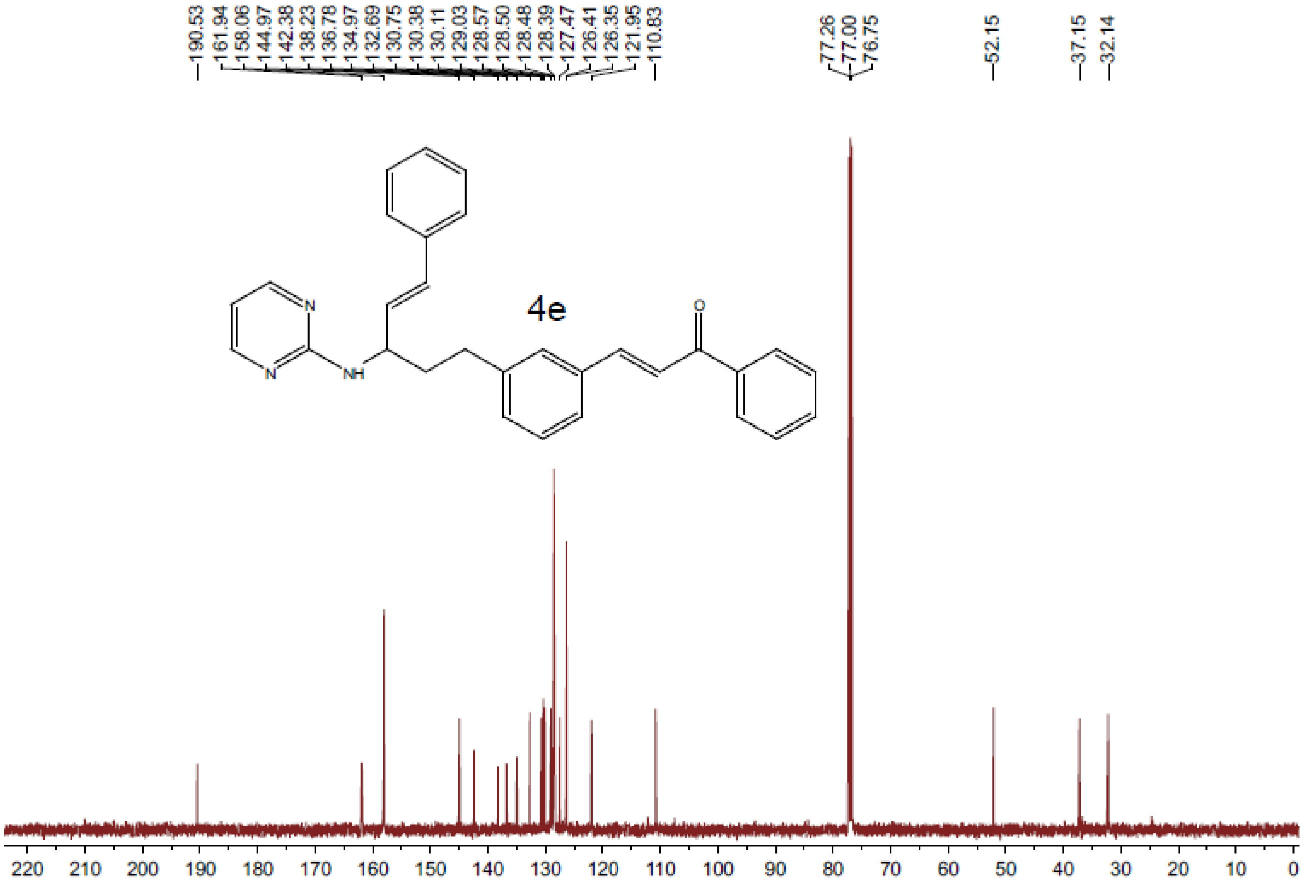
190.53
161.94
158.06
144.97
142.38
138.23
136.78
134.97
132.69
130.75
130.38
130.11
129.03
128.57
128.50
128.48
128.39
127.47
126.41
126.35
121.95
110.83
77.26
77.00
76.75
52.15
37.15
32.14
4e
N
NH
O
220 210 200 190 180 170 160 150 140 130 120 110 100 90 80 70 60 50 40 30 20 10 0

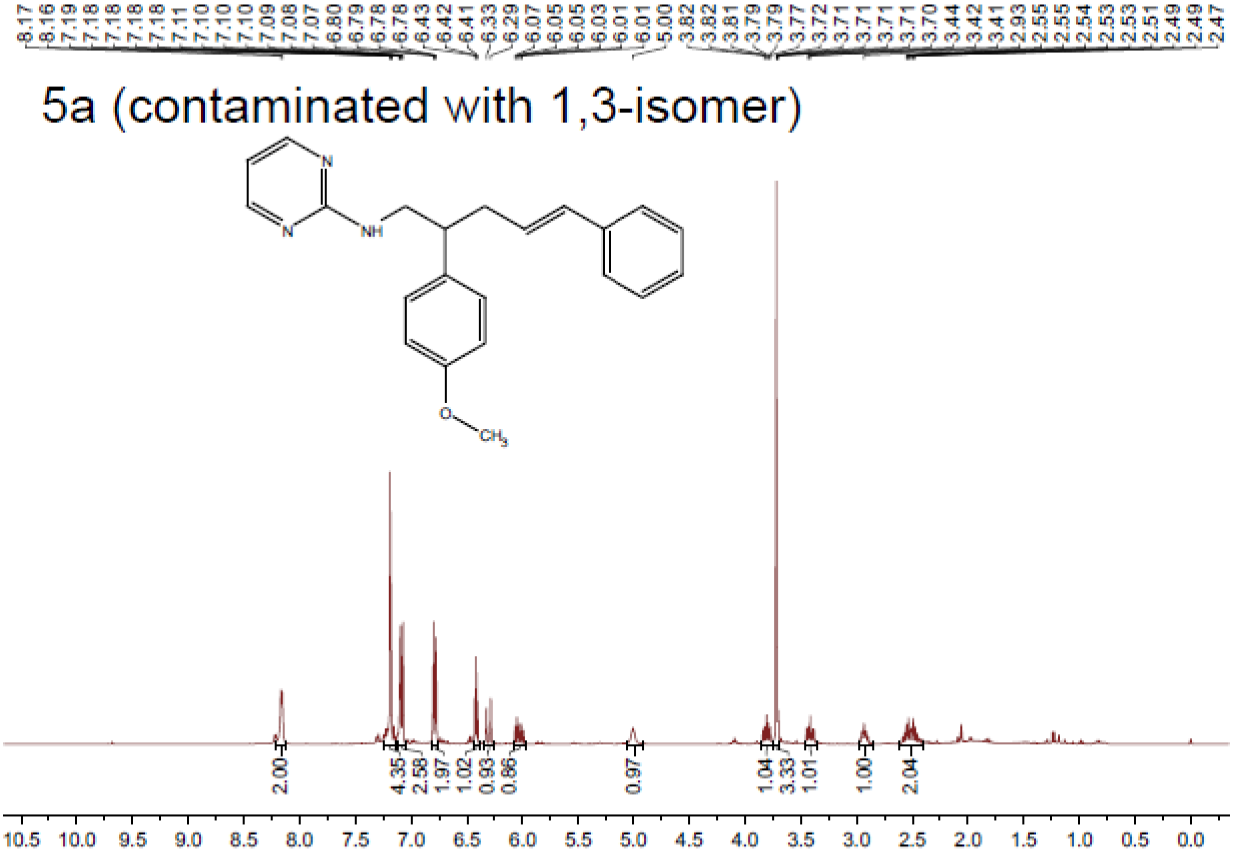
8.17
8.16
7.19
7.18
7.18
7.18
7.18
7.11
7.10
7.10
7.10
7.09
7.08
7.07
6.80
6.79
6.78
6.78
6.43
6.42
6.41
6.33
6.29
6.07
6.05
6.05
6.03
6.01
5.00
3.82
3.82
3.81
3.79
3.79
3.77
3.72
3.71
3.71
3.71
3.71
3.70
3.44
3.42
3.41
2.93
2.55
2.55
2.54
2.53
2.53
2.51
2.49
2.49
2.47
5a (contaminated with 1,3-isomer)
2.00
4.35
2.58
1.97
1.02
0.93
0.86
0.97
1.04
3.33
1.01
1.00
2.04
10.5 10.0 9.5 9.0 8.5 8.0 7.5 7.0 6.5 6.0 5.5 5.0 4.5 4.0 3.5 3.0 2.5 2.0 1.5 1.0 0.5 0.0

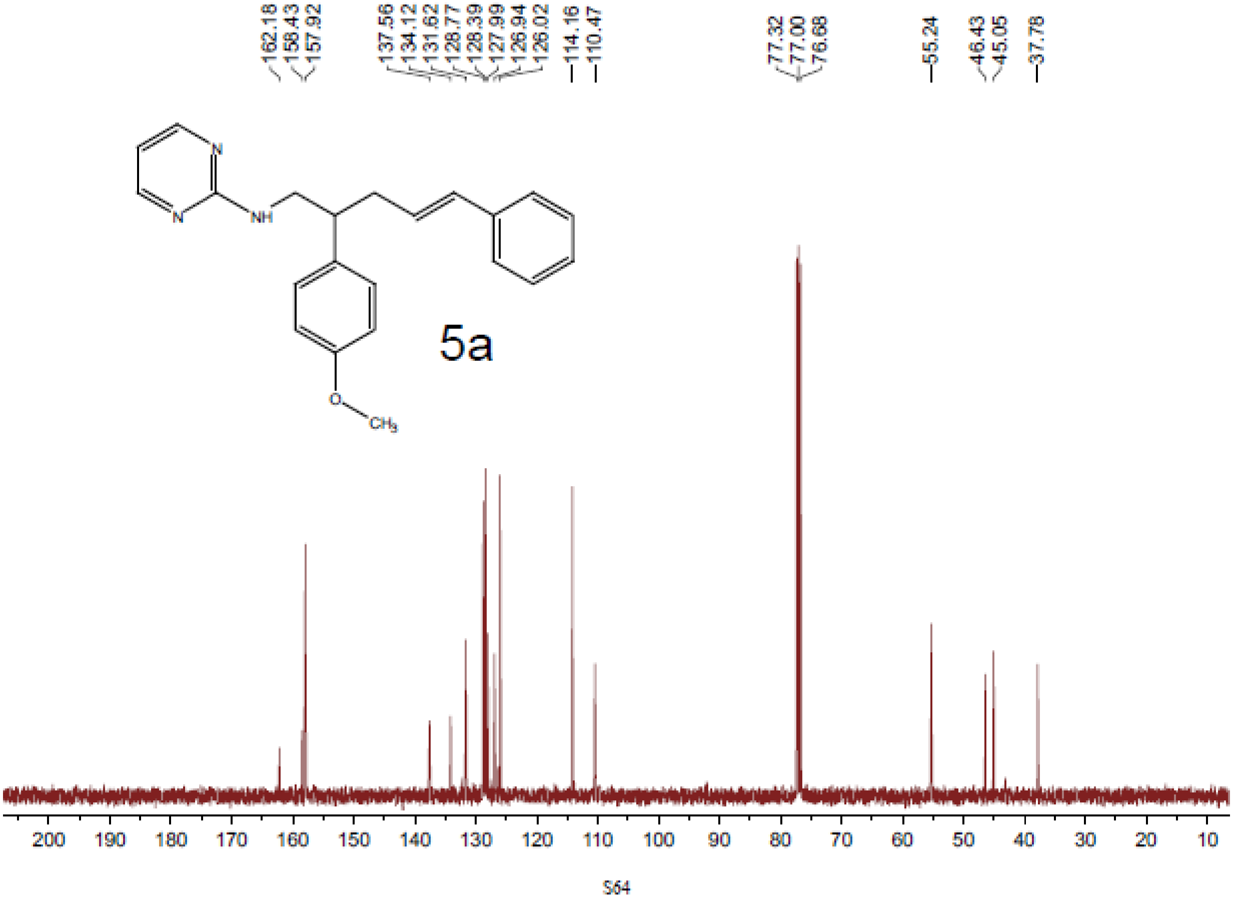
162.18
158.43
157.92
137.56
134.12
131.62
128.77
128.39
127.99
126.94
126.02
114.16
110.47
77.32
77.00
76.68
55.24
46.43
45.05
37.78
5a
200 190 180 170 160 150 140 130 120 110 100 90 80 70 60 50 40 30 20 10
S64

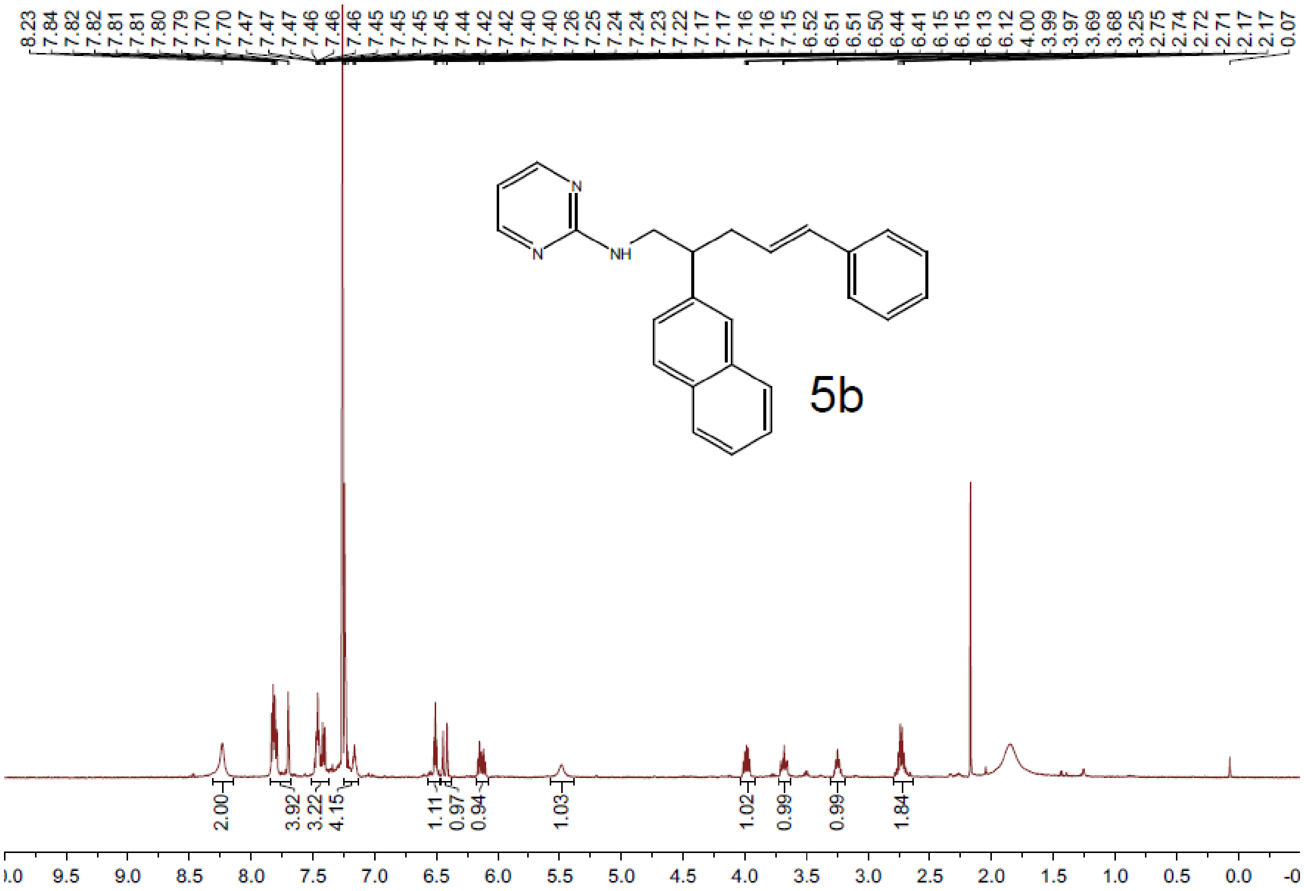
8.23 7.84 7.82 7.81 7.81 7.80 7.79 7.70 7.70 7.47 7.47 7.46 7.46 7.46 7.45 7.45 7.45 7.45 7.44 7.42 7.42 7.40 7.40 7.26 7.25 7.24 7.24 7.23 7.22 7.17 7.17 7.16 7.16 7.15 6.52 6.51 6.51 6.50 6.44 6.41 6.15 6.15 6.13 6.12 4.00 3.99 3.97 3.69 3.68 3.25 2.75 2.74 2.72 2.71 2.17 2.17 0.07
N
N
NH
5b
2.00
3.92
3.22
4.15
1.11
0.97
0.94
1.03
1.02
0.99
0.99
1.84
9.5 9.0 8.5 8.0 7.5 7.0 6.5 6.0 5.5 5.0 4.5 4.0 3.5 3.0 2.5 2.0 1.5 1.0 0.5 0.0

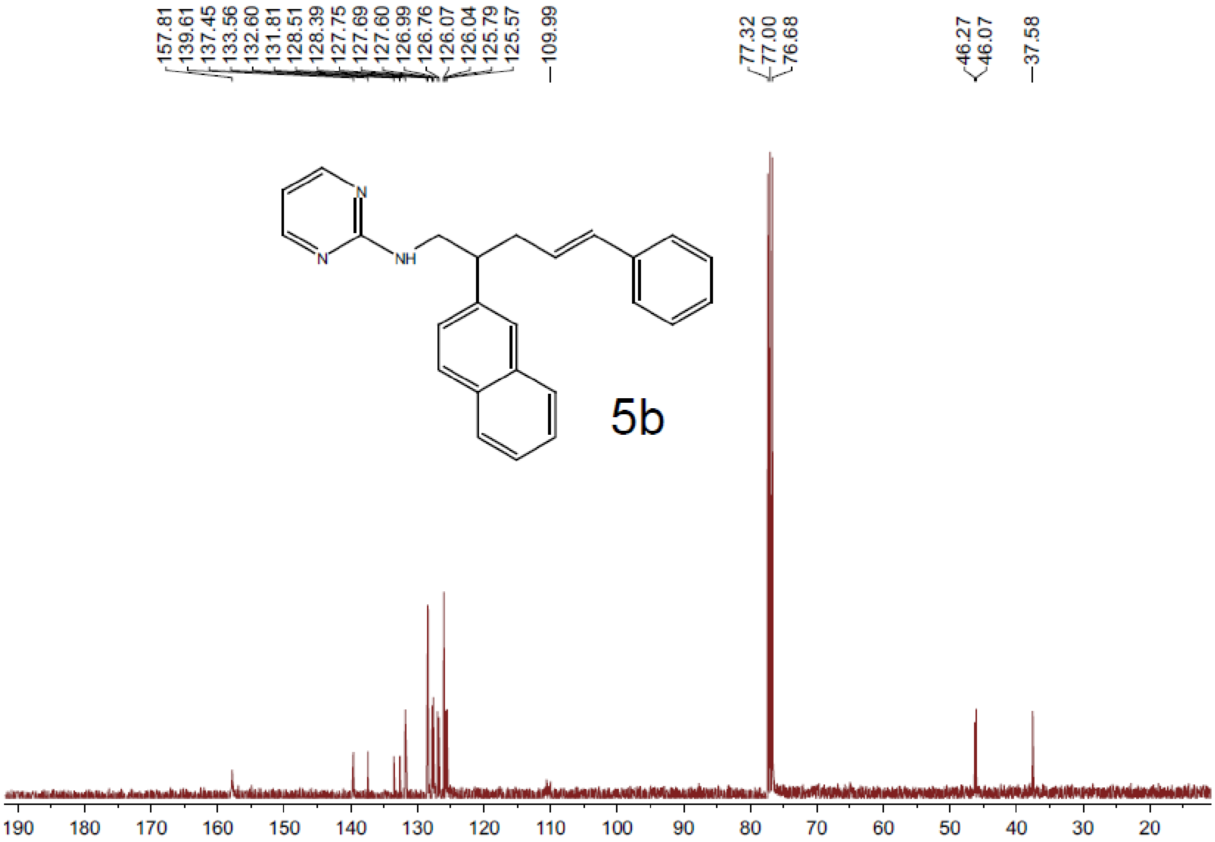
157.81 139.61 137.45 133.56 132.60 131.81 128.51 128.39 127.75 127.69 127.60 126.99 126.76 126.07 126.04 125.79 125.57
109.99
77.32 77.00 76.68
46.27 46.07
37.58
N
N
NH
5b
190 180 170 160 150 140 130 120 110 100 90 80 70 60 50 40 30 20

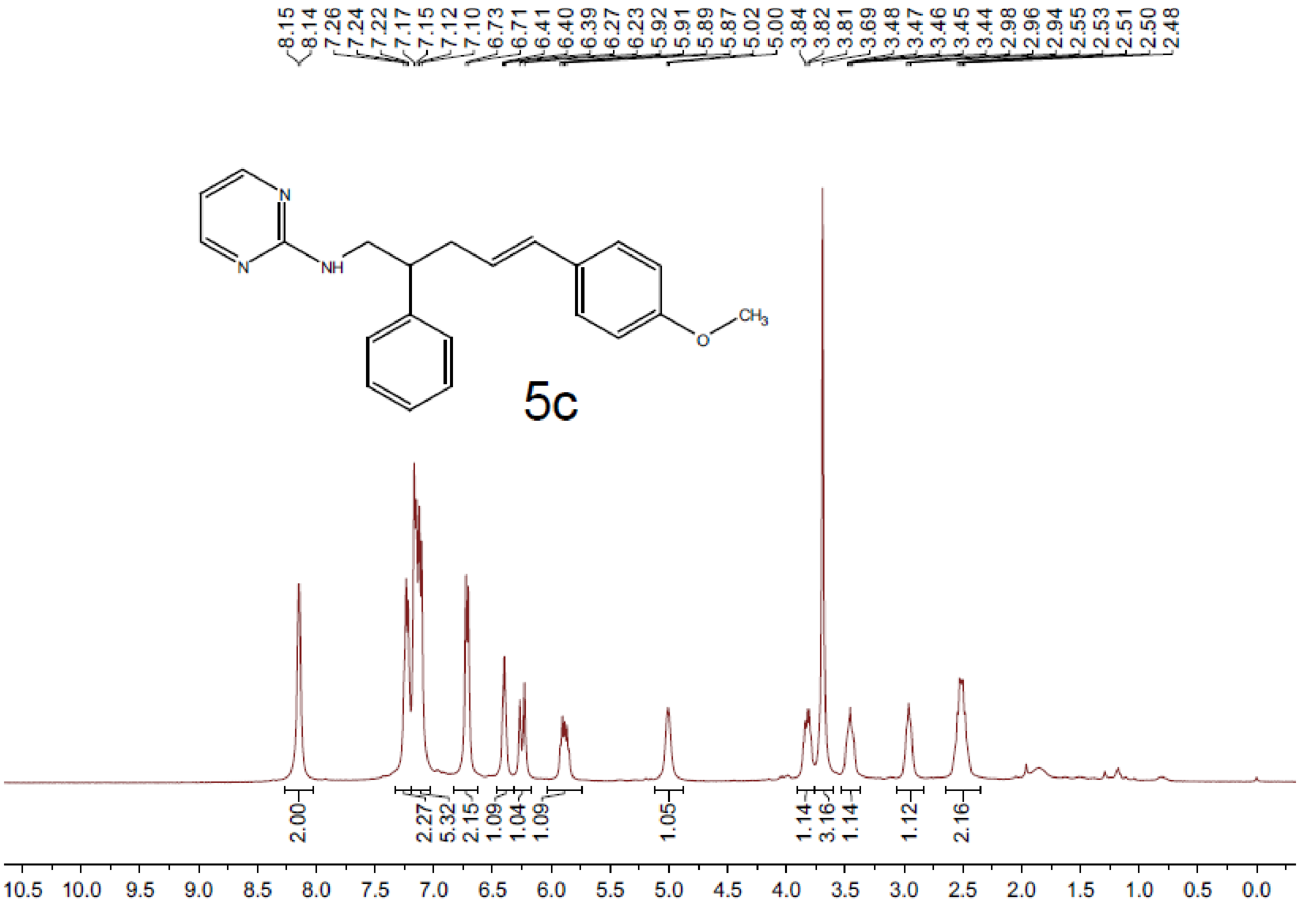

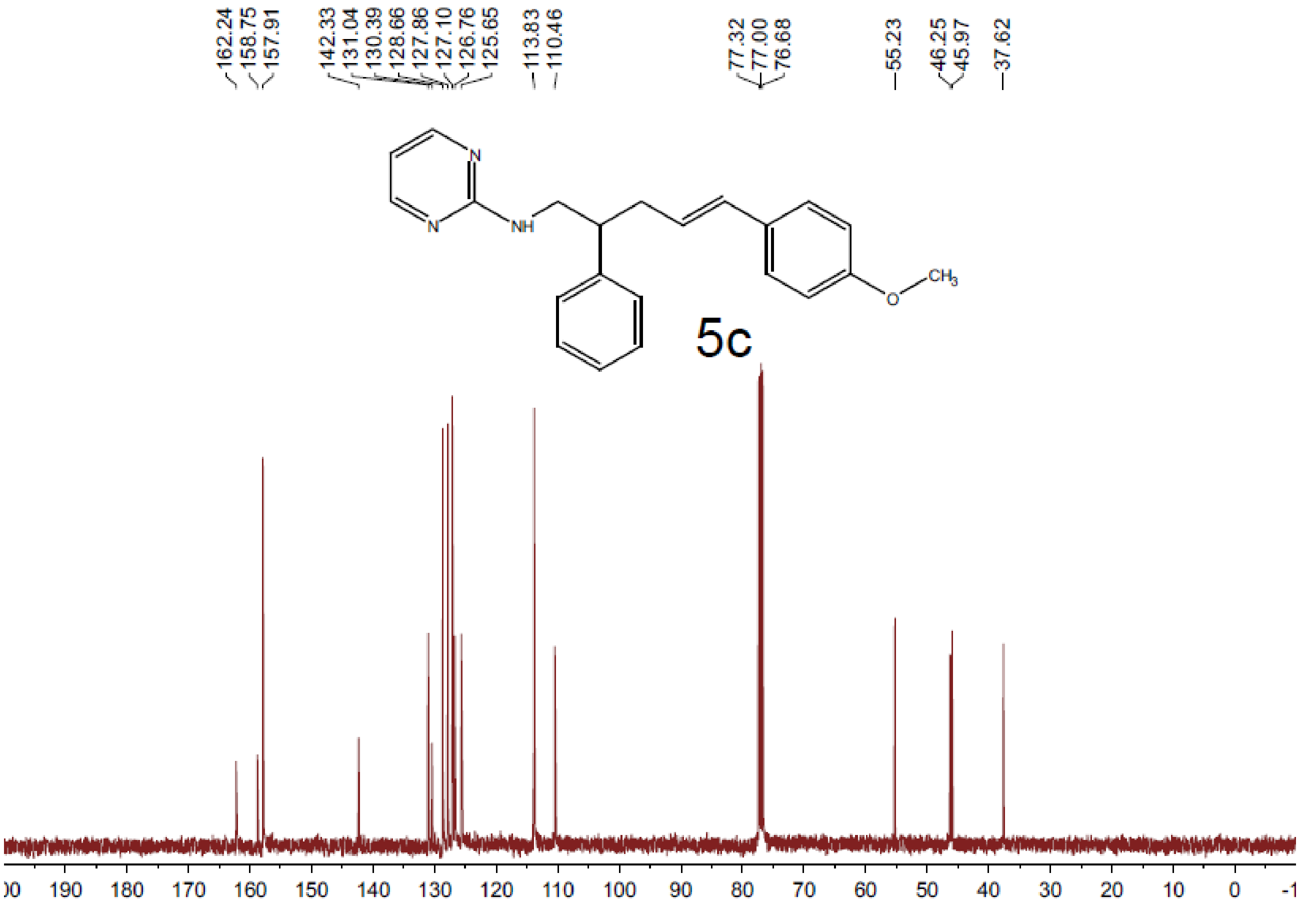

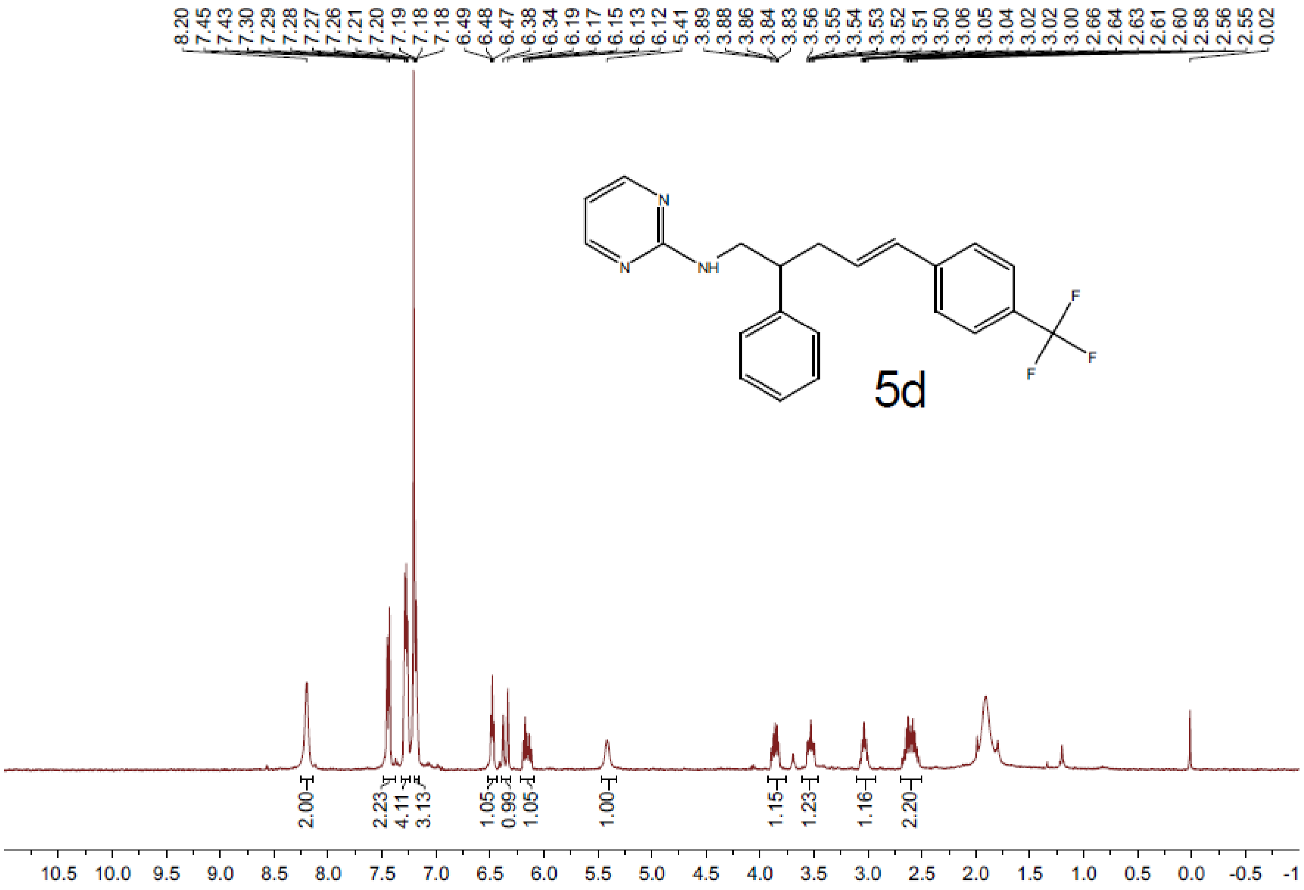
8.20 7.45 7.43 7.30 7.29 7.28 7.27 7.26 7.21 7.20 7.19 7.18 7.18 6.49 6.48 6.47 6.38 6.34 6.19 6.17 6.15 6.13 6.12 5.41 3.89 3.88 3.86 3.84 3.83 3.56 3.55 3.54 3.53 3.52 3.51 3.50 3.06 3.05 3.04 3.02 3.02 3.00 2.66 2.64 2.63 2.61 2.60 2.58 2.56 2.55 0.02
N
N
NH
F
F
F
5d
2.00 2.23 4.11 3.13 1.05 0.99 1.05 1.00 1.15 1.23 1.16 2.20
10.5 10.0 9.5 9.0 8.5 8.0 7.5 7.0 6.5 6.0 5.5 5.0 4.5 4.0 3.5 3.0 2.5 2.0 1.5 1.0 0.5 0.0 -0.5 -1

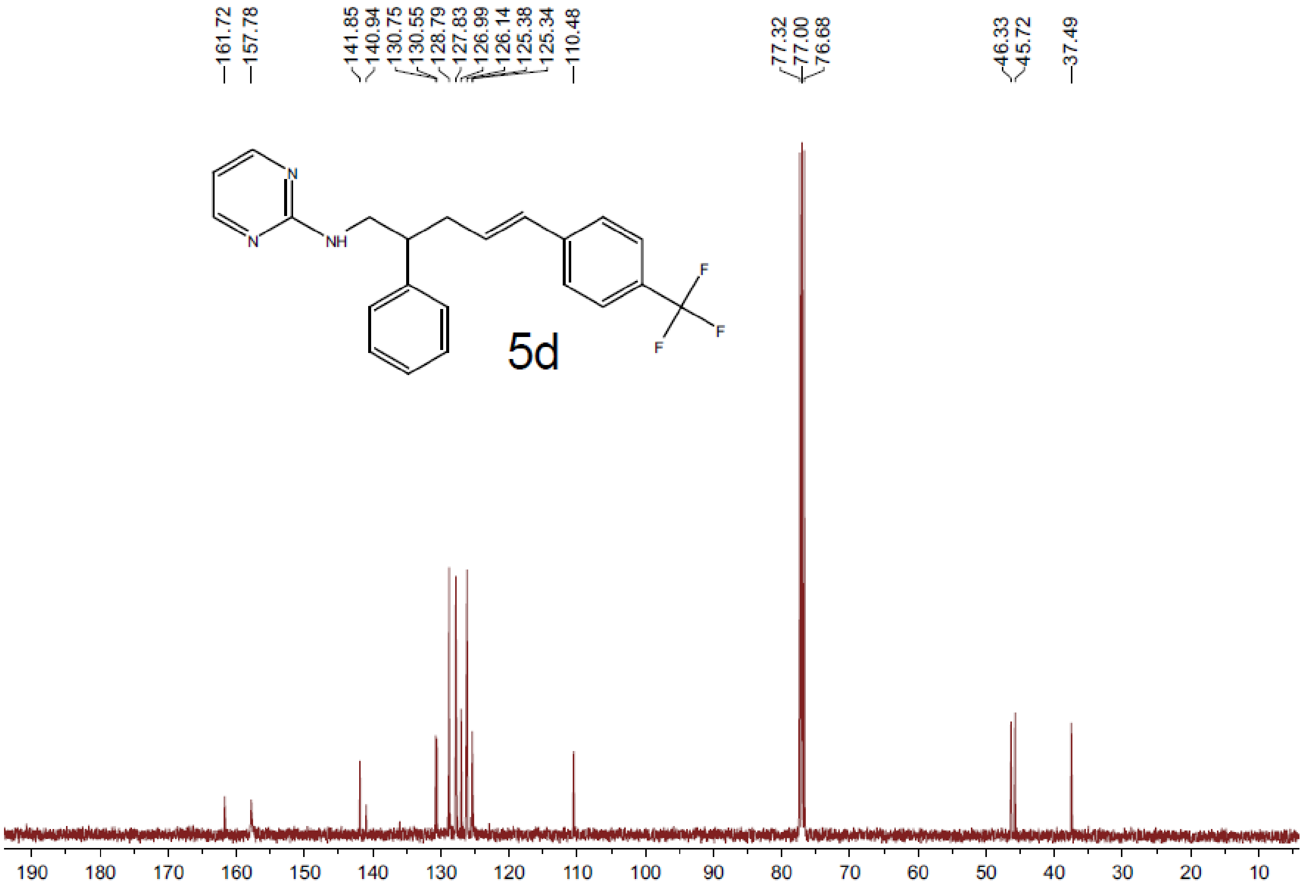
161.72 157.78 141.85 140.94 130.75 130.55 128.79 127.83 126.99 126.14 125.38 125.34 110.48 77.32 77.00 76.68 46.33 45.72 37.49
N
N
NH
F
F
F
5d
190 180 170 160 150 140 130 120 110 100 90 80 70 60 50 40 30 20 10

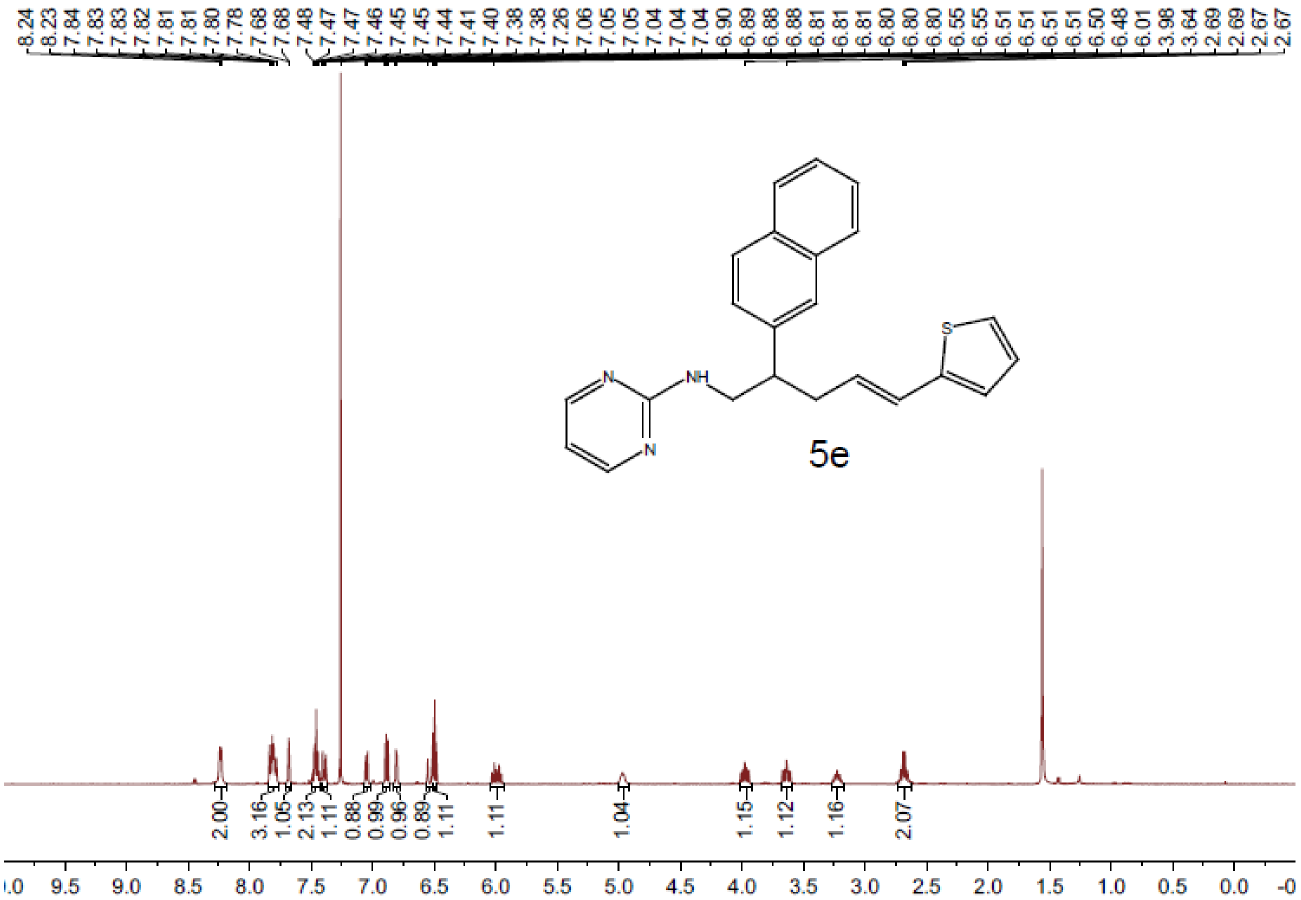

8.24 8.23 7.84 7.83 7.83 7.82 7.81 7.81 7.80 7.78 7.68 7.68 7.48 7.47 7.47 7.46 7.45 7.45 7.44 7.41 7.40 7.38 7.38 7.26 7.06 7.05 7.05 7.04 7.04 7.04 6.90 6.89 6.88 6.88 6.81 6.81 6.81 6.80 6.80 6.80 6.55 6.55 6.51 6.51 6.51 6.51 6.50 6.48 6.01 3.98 3.64 2.69 2.69 2.67 2.67
NH
N
N
S
5e
2.00 3.16 1.05 2.13 1.11 0.88 0.99 0.96 0.89 1.11 1.11 1.04 1.15 1.12 1.16 2.07
9.5 9.0 8.5 8.0 7.5 7.0 6.5 6.0 5.5 5.0 4.5 4.0 3.5 3.0 2.5 2.0 1.5 1.0 0.5 0.0

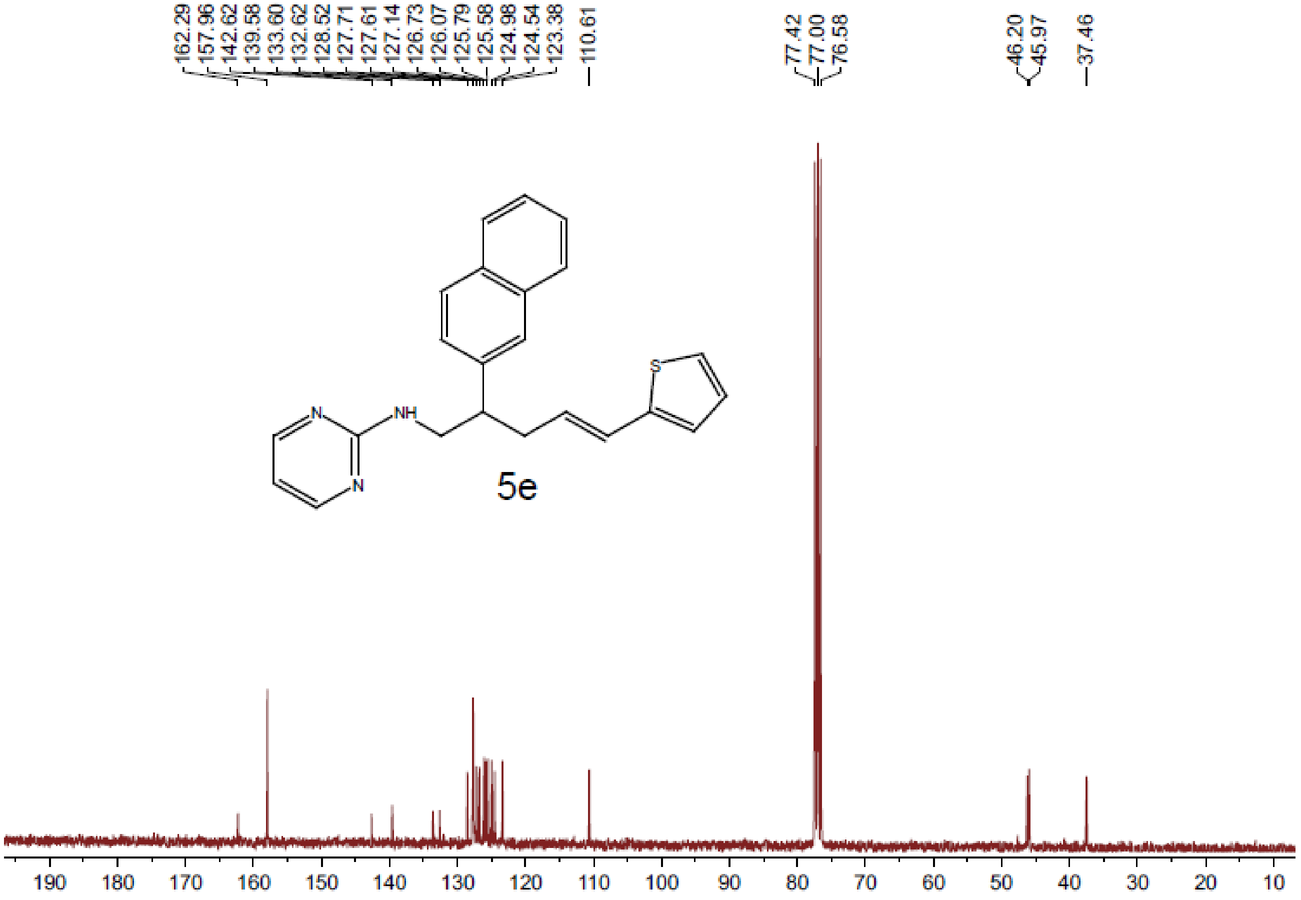

162.29 157.96 142.62 139.58 133.60 132.62 128.52 127.71 127.61 127.14 126.73 126.07 125.79 125.58 124.98 124.54 123.38 110.61
77.42 77.00 76.58
46.20 45.97 37.46
NH
N
N
S
5e
190 180 170 160 150 140 130 120 110 100 90 80 70 60 50 40 30 20 10

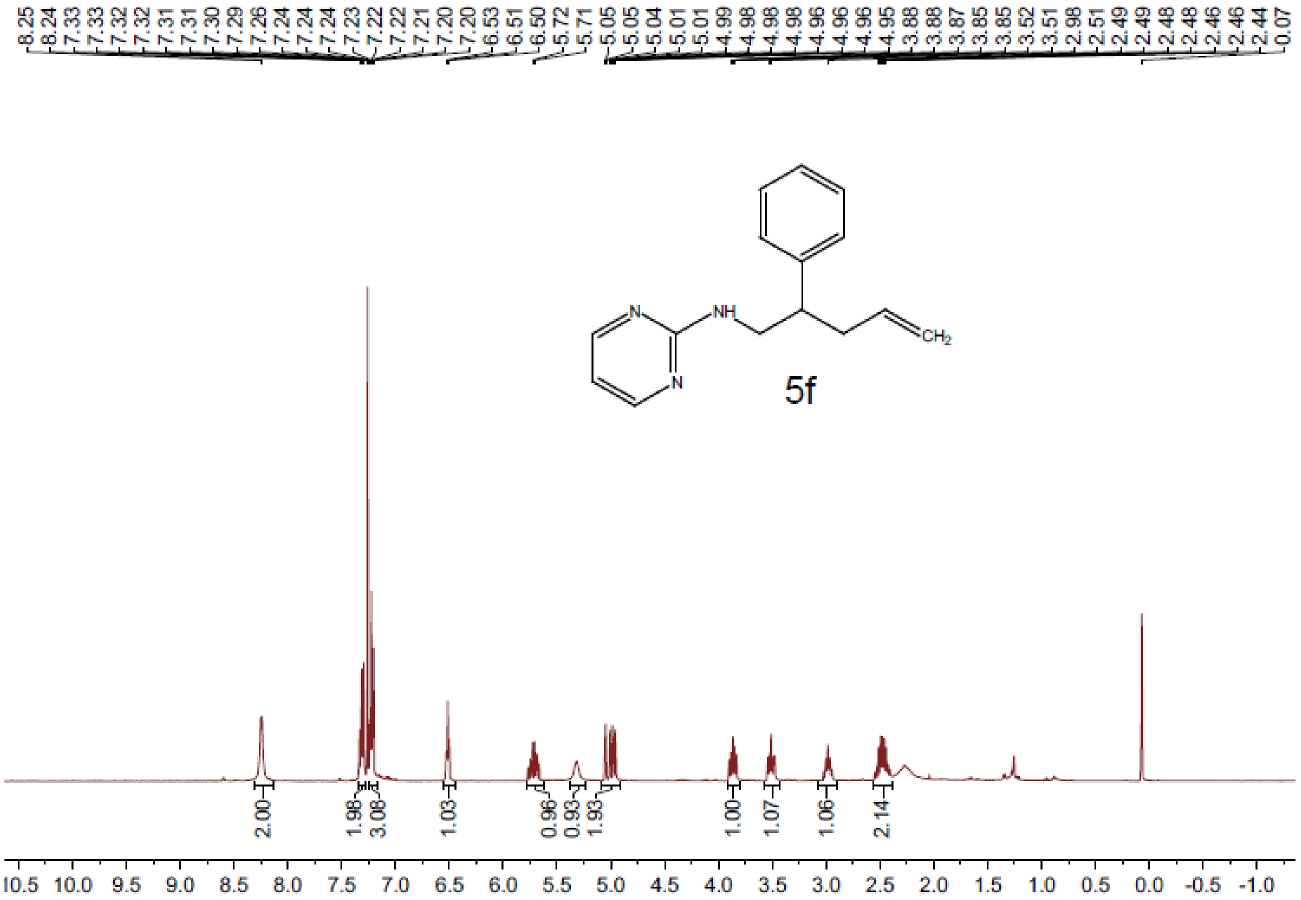

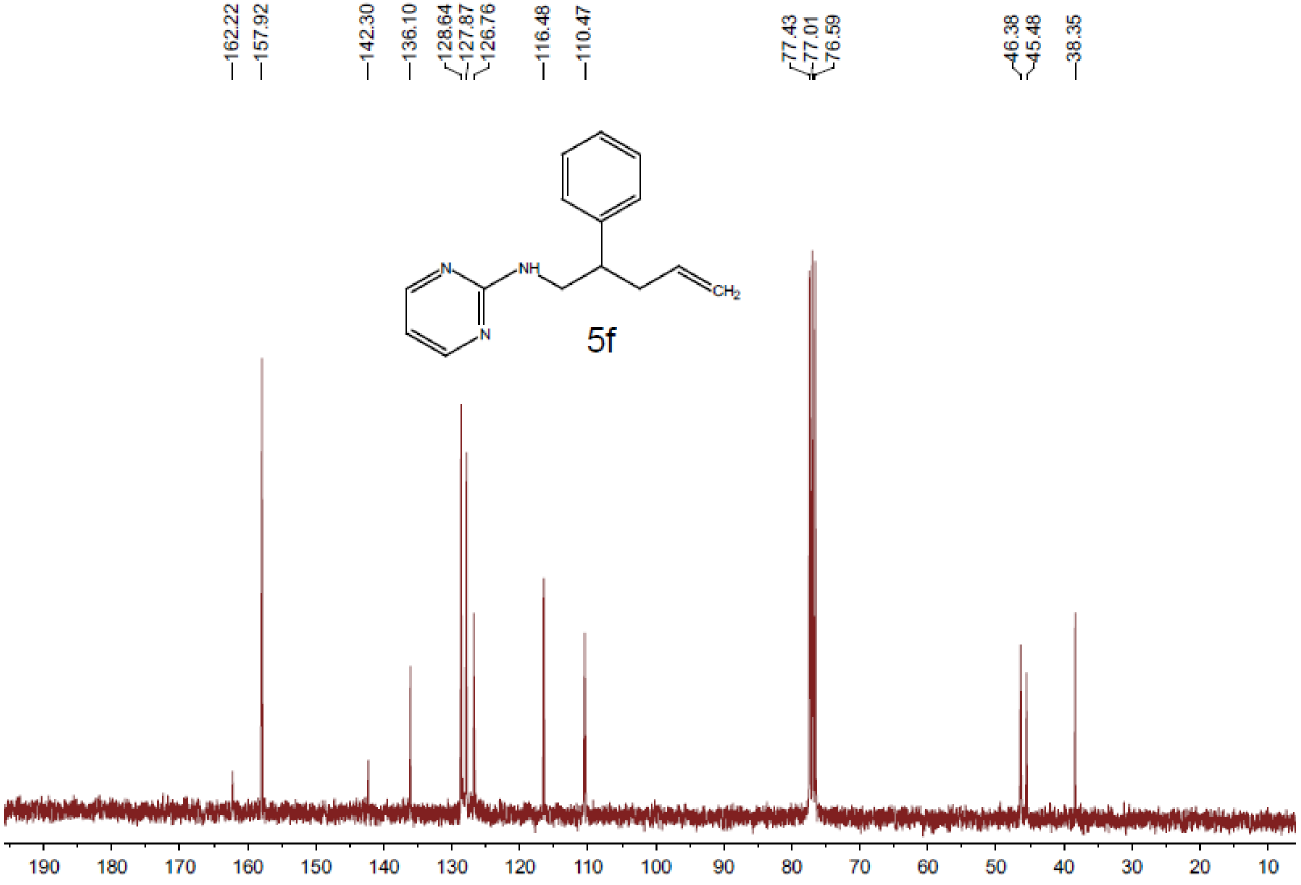

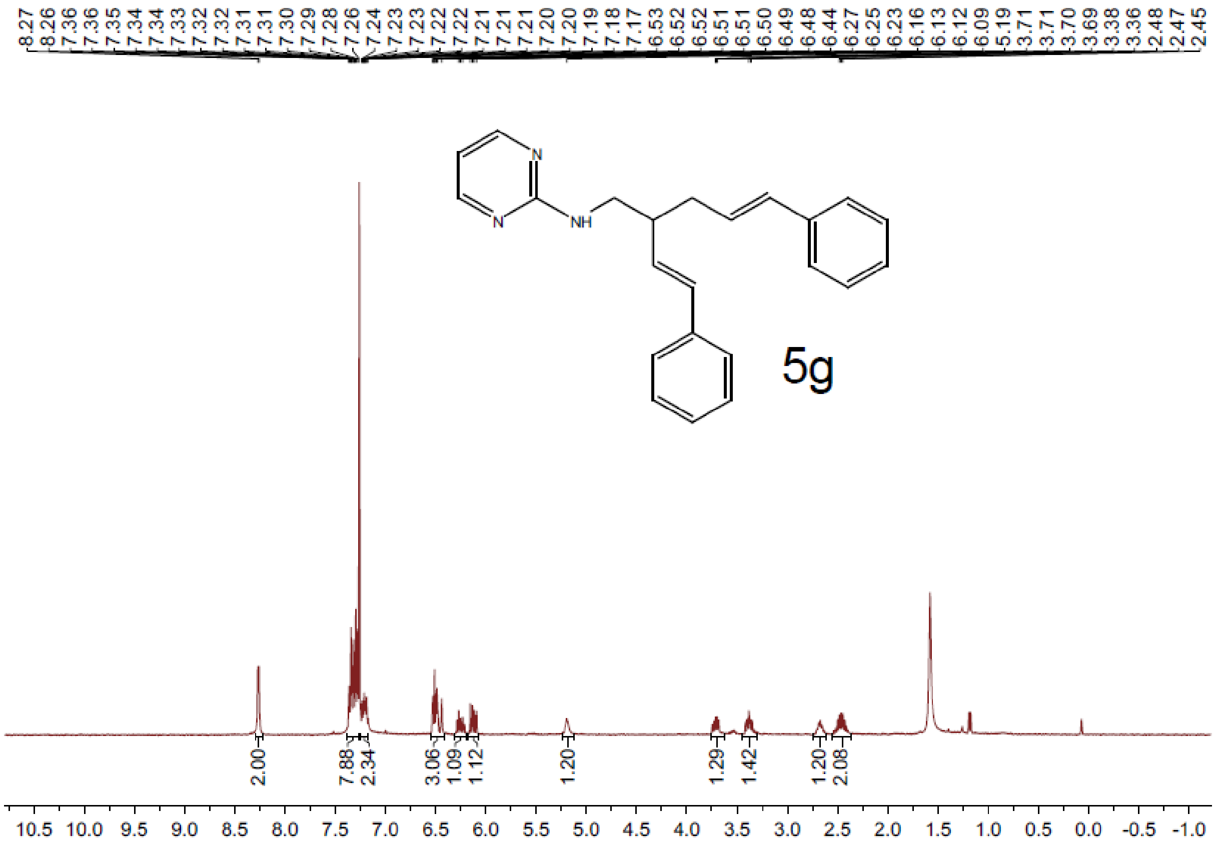
8.27 8.26 7.36 7.36 7.35 7.34 7.34 7.33 7.32 7.32 7.31 7.31 7.30 7.29 7.28 7.26 7.24 7.23 7.23 7.22 7.22 7.21 7.21 7.21 7.20 7.20 7.19 7.18 7.17 6.53 6.52 6.52 6.51 6.51 6.50 6.49 6.48 6.44 6.27 6.25 6.23 6.16 6.13 6.12 6.09 5.19 3.71 3.71 3.70 3.69 3.38 3.36 2.48 2.47 2.45
NH
N
N
5g
2.00 7.88 2.34 3.06 1.09 1.12 1.20 1.29 1.42 1.20 2.08
10.5 10.0 9.5 9.0 8.5 8.0 7.5 7.0 6.5 6.0 5.5 5.0 4.5 4.0 3.5 3.0 2.5 2.0 1.5 1.0 0.5 0.0 -0.5 -1.0

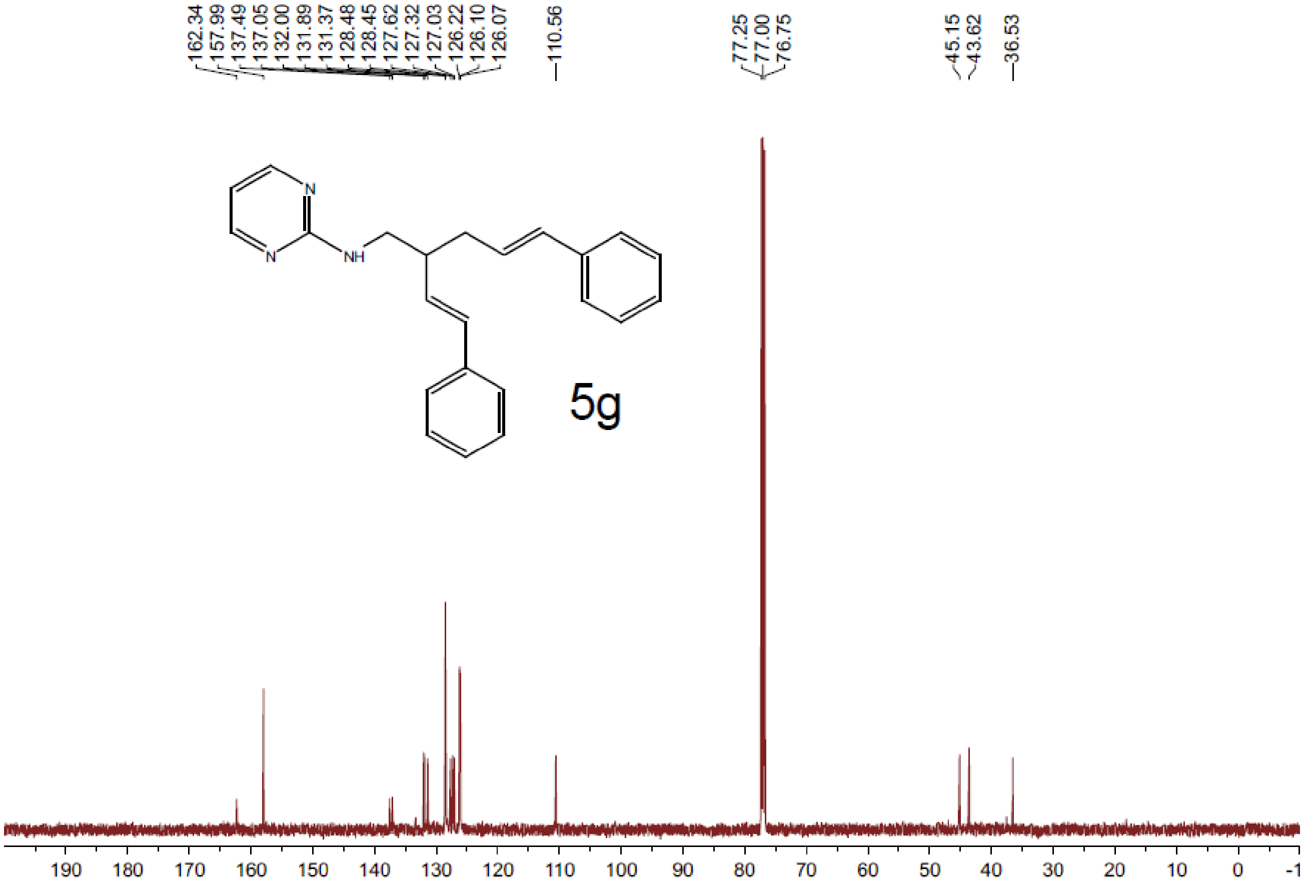
162.34 157.99 137.49 137.05 132.00 131.89 131.37 128.48 128.45 127.62 127.32 127.03 126.22 126.10 126.07 110.56 77.25 77.00 76.75 45.15 43.62 36.53
NH
N
N
5g
190 180 170 160 150 140 130 120 110 100 90 80 70 60 50 40 30 20 10 0 -1

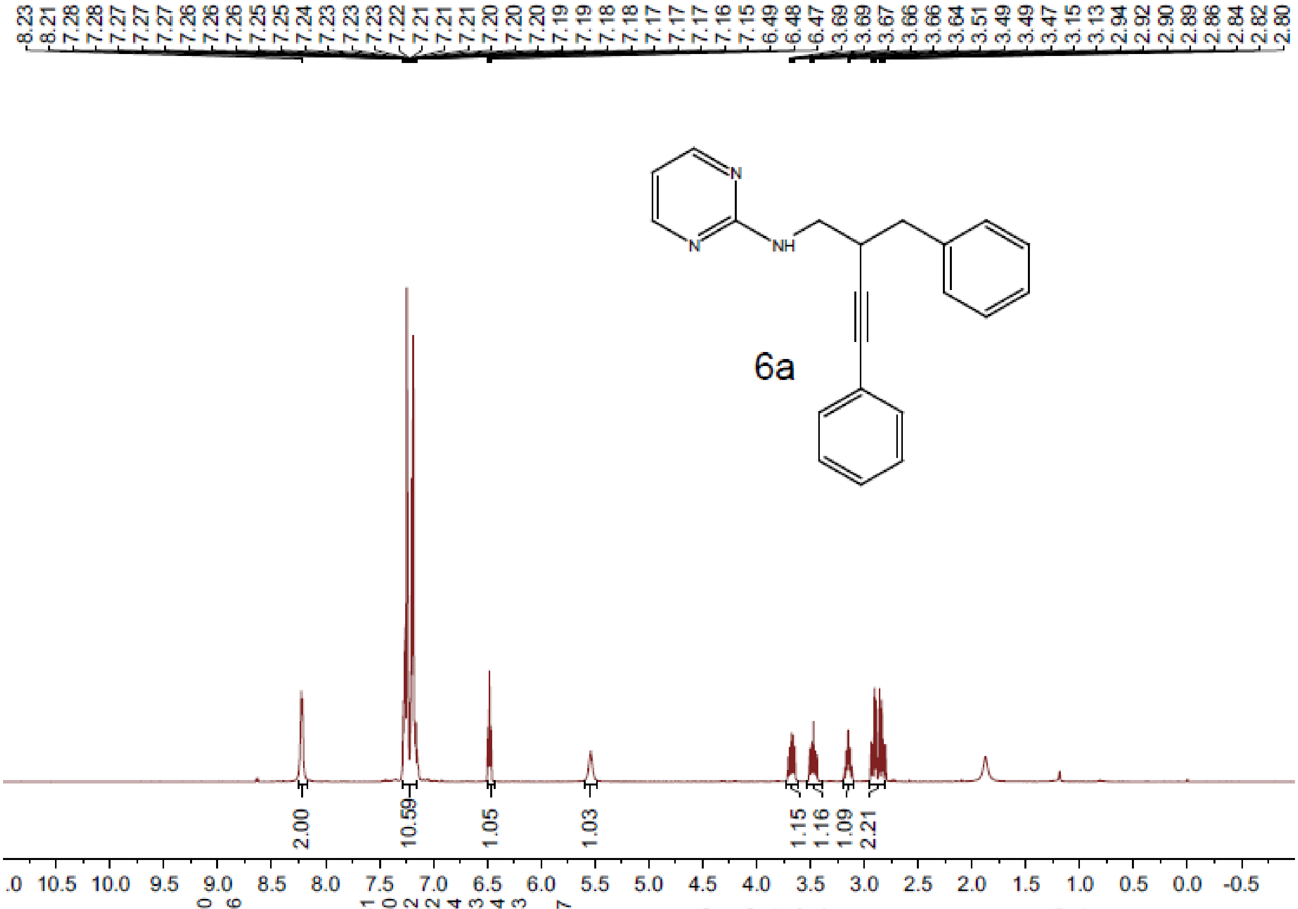
6a
2.00
10.59
1.05
1.03
1.15
1.16
1.09
2.21

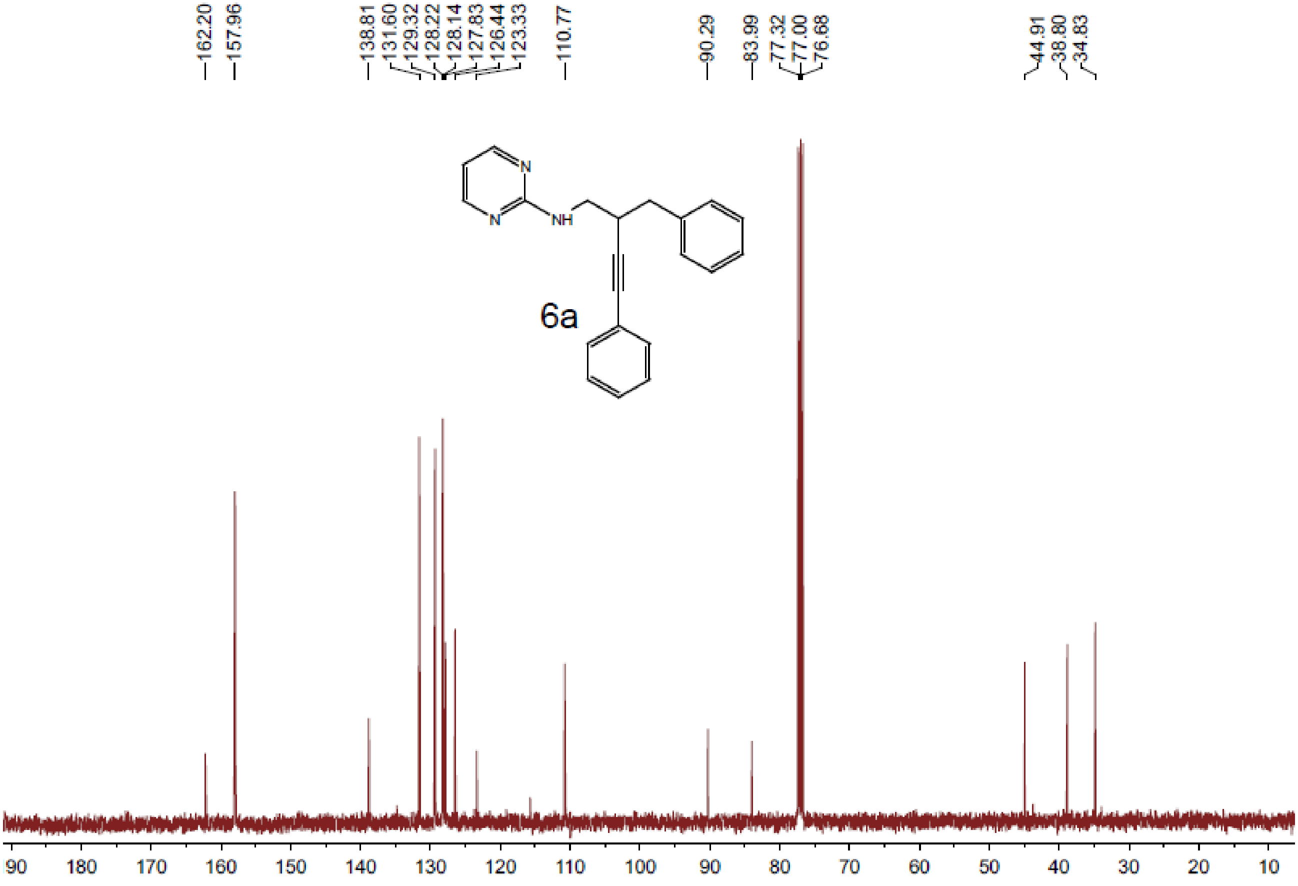
6a
162.20
157.96
138.81
131.60
129.32
128.22
128.14
127.83
126.44
123.33
110.77
90.29
83.99
77.32
77.00
76.68
44.91
38.80
34.83

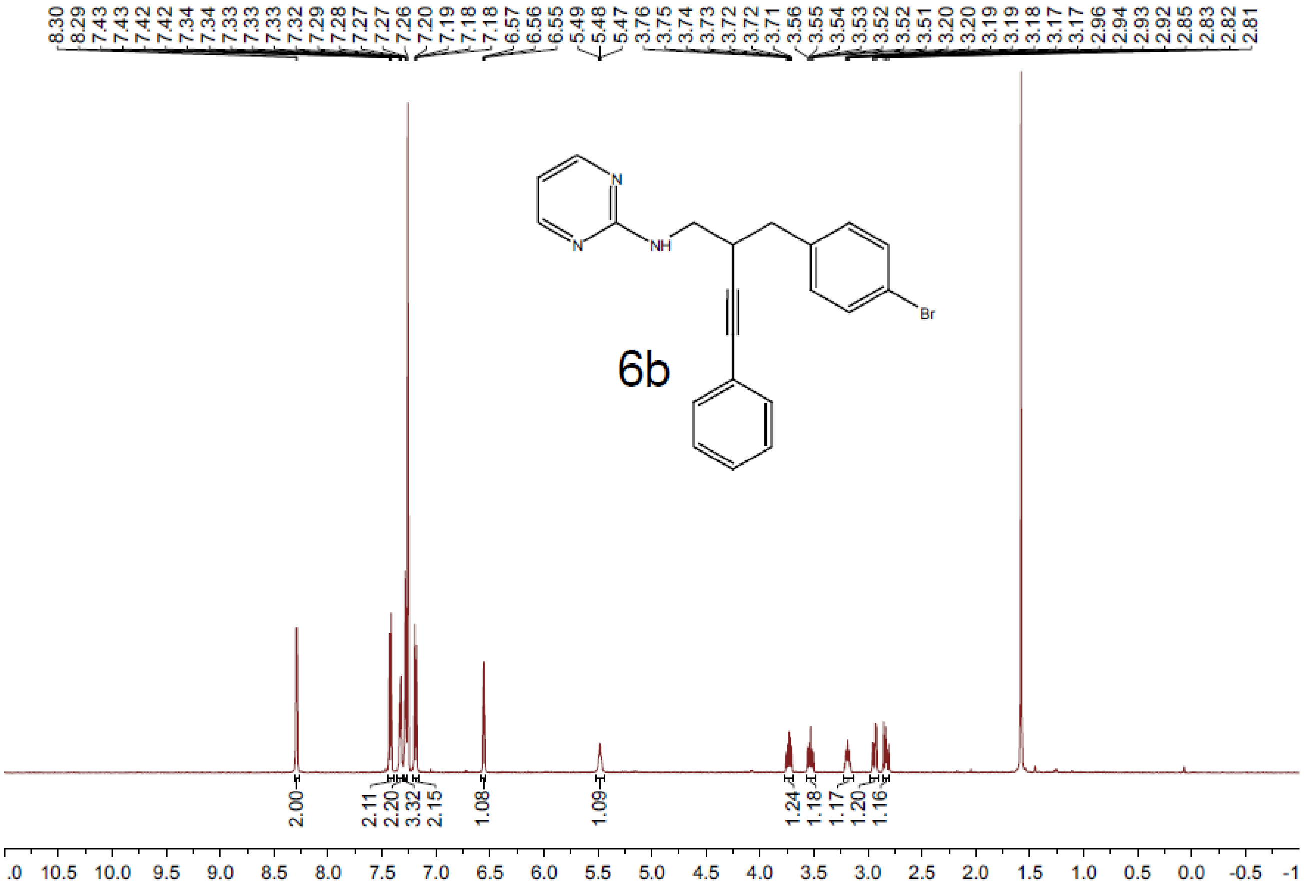
8.30
8.29
7.43
7.43
7.42
7.42
7.34
7.34
7.33
7.33
7.33
7.32
7.29
7.28
7.27
7.27
7.26
7.20
7.19
7.18
7.18
6.57
6.56
6.55
5.49
5.48
5.47
3.76
3.75
3.74
3.73
3.72
3.72
3.71
3.56
3.55
3.54
3.53
3.52
3.52
3.51
3.20
3.20
3.19
3.19
3.18
3.17
2.96
2.94
2.93
2.92
2.85
2.83
2.82
2.81
N
NH
N
Br
6b
2.00
2.11
2.20
3.32
2.15
1.08
1.09
1.24
1.18
1.17
1.20
1.16
.0
10.5
10.0
9.5
9.0
8.5
8.0
7.5
7.0
6.5
6.0
5.5
5.0
4.5
4.0
3.5
3.0
2.5
2.0
1.5
1.0
0.5
0.0
-0.5
-1

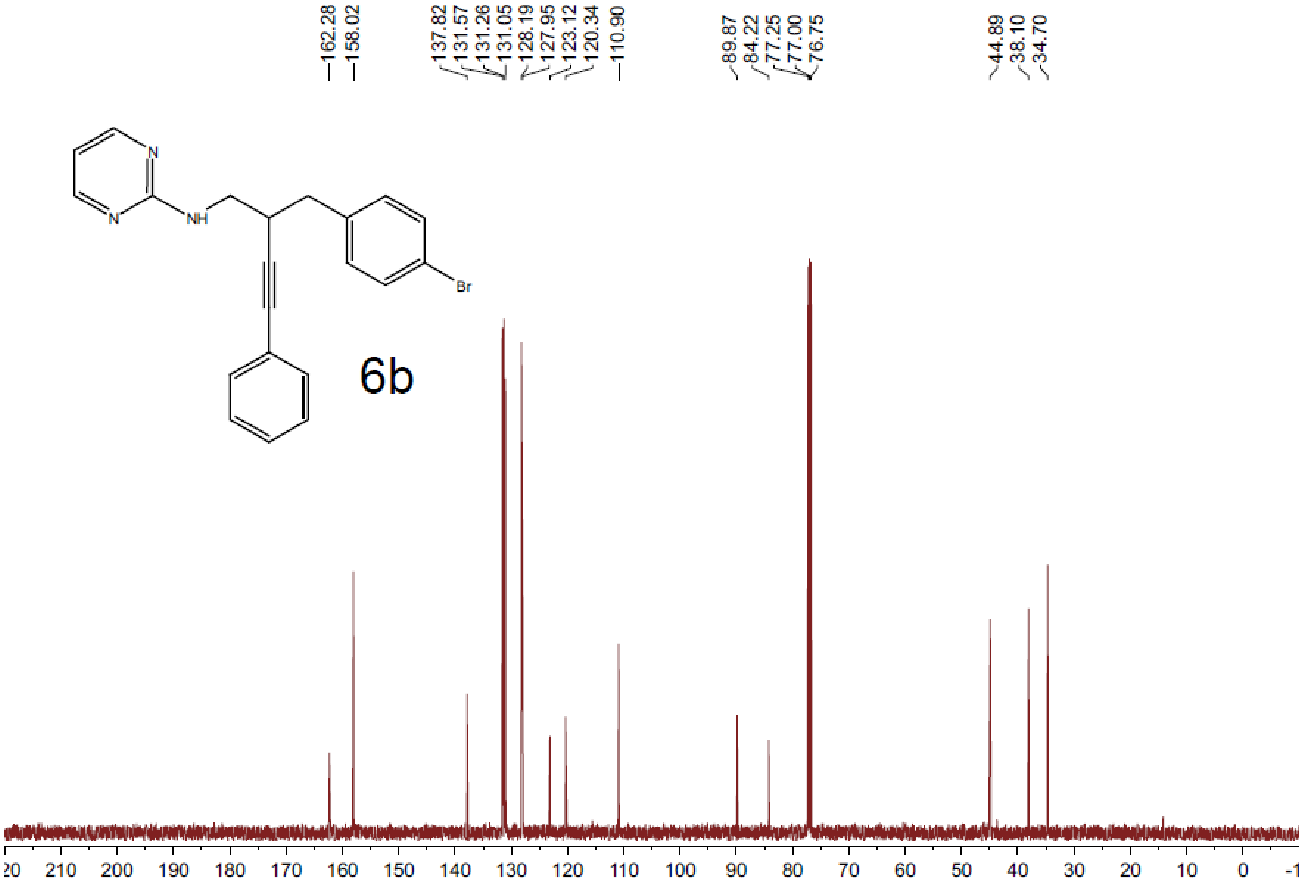
162.28
158.02
137.82
131.57
131.26
131.05
128.19
127.95
123.12
120.34
110.90
89.87
84.22
77.25
77.00
76.75
44.89
38.10
34.70
N
NH
N
Br
6b
20
210
200
190
180
170
160
150
140
130
120
110
100
90
80
70
60
50
40
30
20
10
0
-1

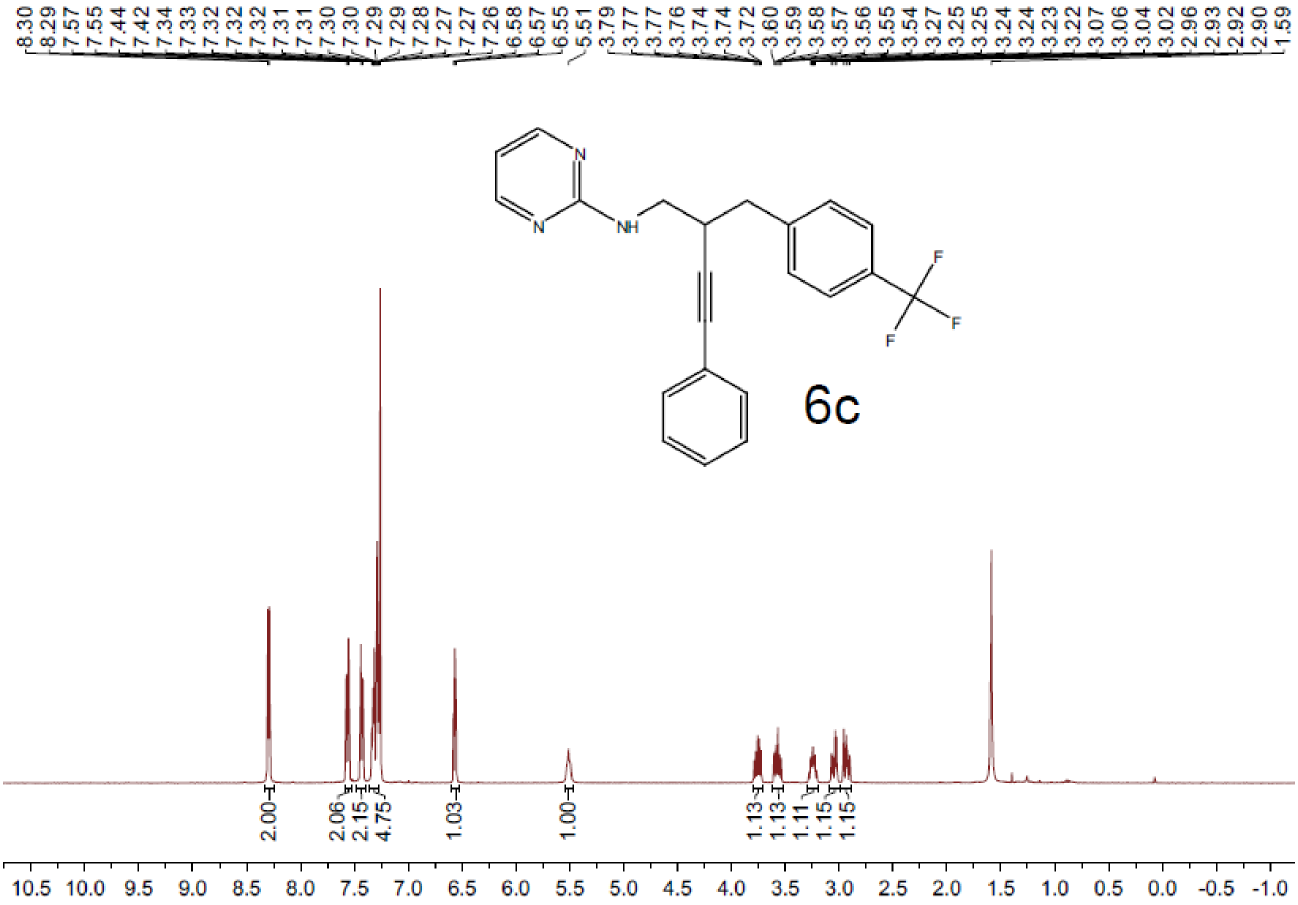
8.30
8.29
7.57
7.55
7.44
7.42
7.34
7.33
7.32
7.32
7.32
7.31
7.31
7.30
7.30
7.29
7.29
7.28
7.27
7.27
7.26
6.58
6.57
6.55
5.51
3.79
3.77
3.77
3.76
3.74
3.74
3.72
3.60
3.59
3.58
3.57
3.56
3.55
3.54
3.27
3.25
3.25
3.24
3.24
3.23
3.22
3.07
3.06
3.04
3.02
2.96
2.93
2.92
2.90
1.59
N
N
NH
F
F
F
6c
2.00
2.06
2.15
4.75
1.03
1.00
1.13
1.13
1.11
1.15
1.15
10.5 10.0 9.5 9.0 8.5 8.0 7.5 7.0 6.5 6.0 5.5 5.0 4.5 4.0 3.5 3.0 2.5 2.0 1.5 1.0 0.5 0.0 -0.5 -1.0

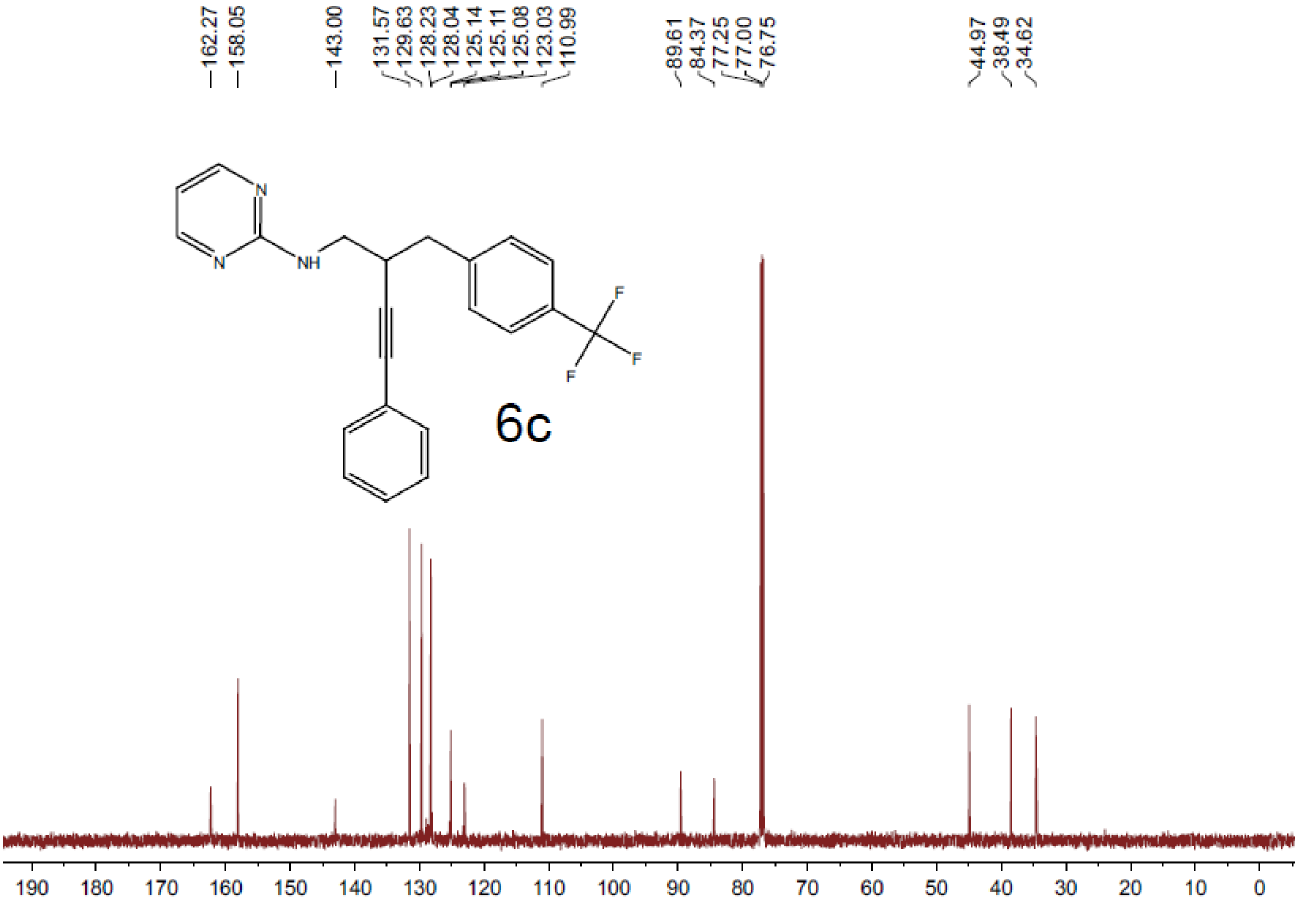
162.27
158.05
143.00
131.57
129.63
128.23
128.04
125.14
125.11
125.08
123.03
110.99
89.61
84.37
77.25
77.00
76.75
44.97
38.49
34.62
N
N
NH
F
F
F
6c
190 180 170 160 150 140 130 120 110 100 90 80 70 60 50 40 30 20 10 0

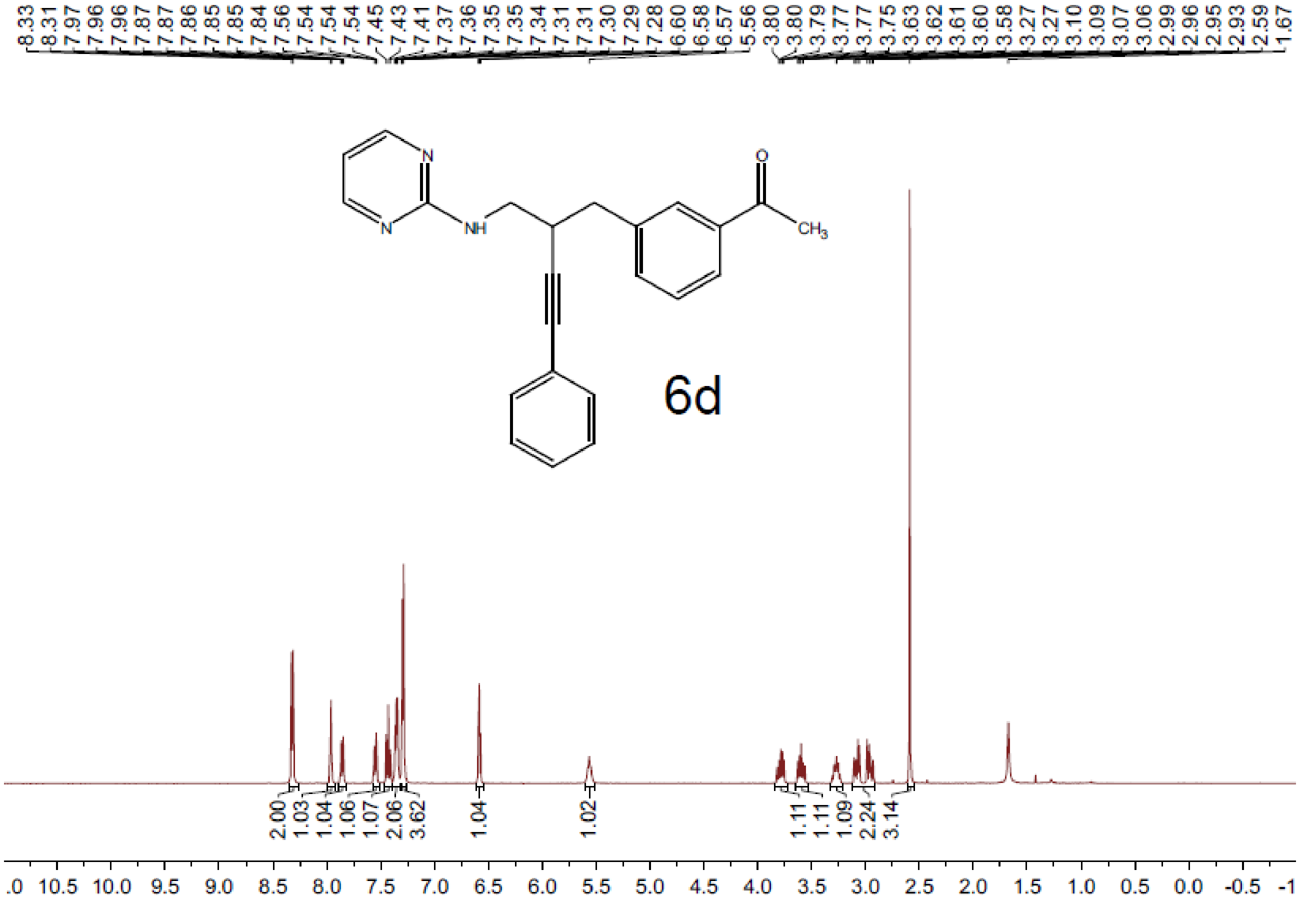

8.33
8.31
7.97
7.96
7.96
7.87
7.87
7.86
7.85
7.85
7.84
7.56
7.54
7.54
7.54
7.45
7.43
7.41
7.37
7.36
7.35
7.35
7.34
7.31
7.31
7.30
7.29
7.28
6.60
6.58
6.57
5.56
3.80
3.80
3.79
3.77
3.77
3.75
3.63
3.62
3.61
3.60
3.58
3.27
3.27
3.10
3.09
3.07
3.06
2.99
2.96
2.95
2.93
2.59
1.67
N
N
NH
O
CH3
6d
2.00
1.03
1.04
1.06
1.07
2.06
3.62
1.04
1.02
1.11
1.11
1.09
2.24
3.14

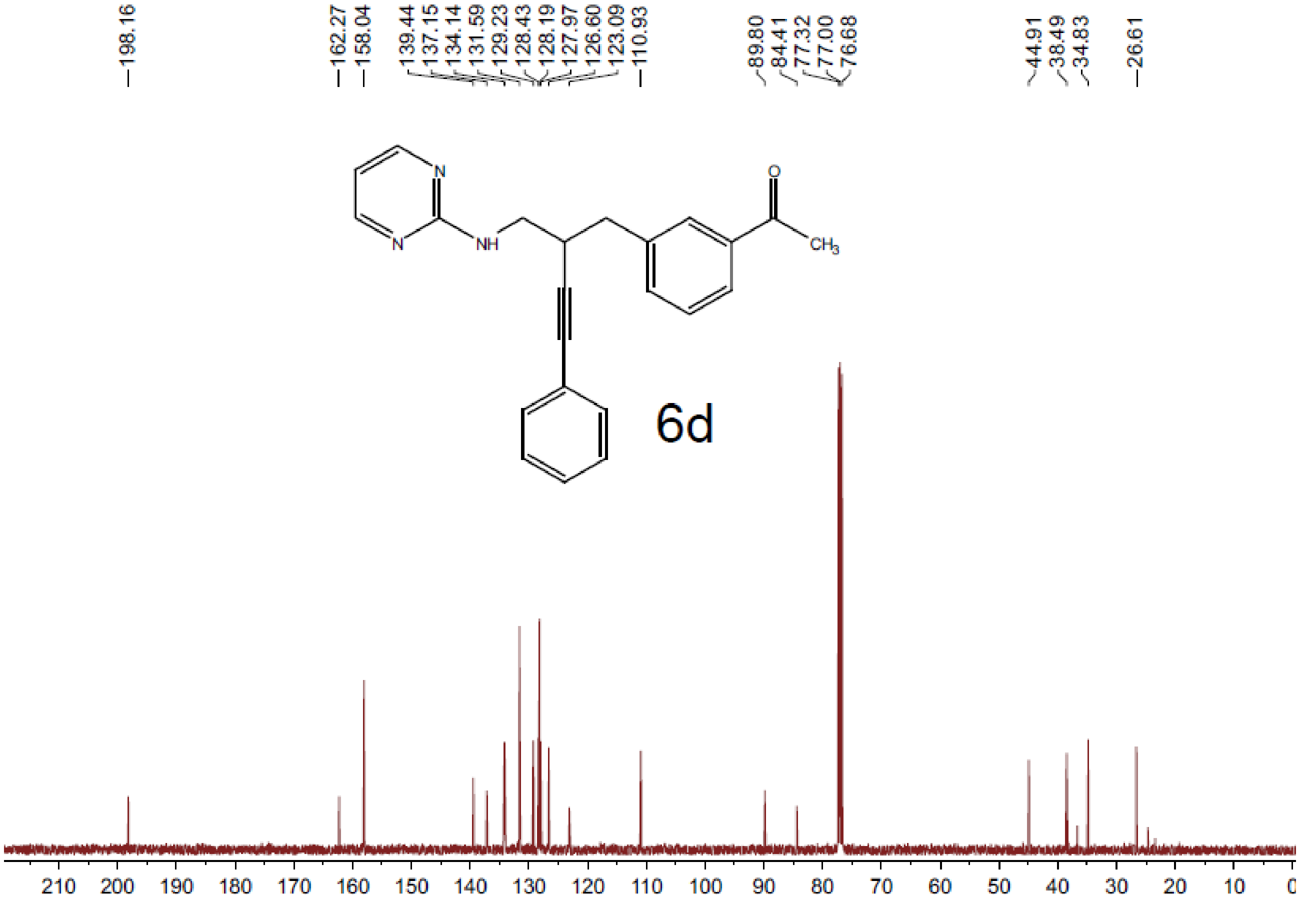

198.16
162.27
158.04
139.44
137.15
134.14
131.59
129.23
128.43
128.19
127.97
126.60
123.09
110.93
89.80
84.41
77.32
77.00
76.68
44.91
38.49
34.83
26.61
N
N
NH
O
CH3
6d

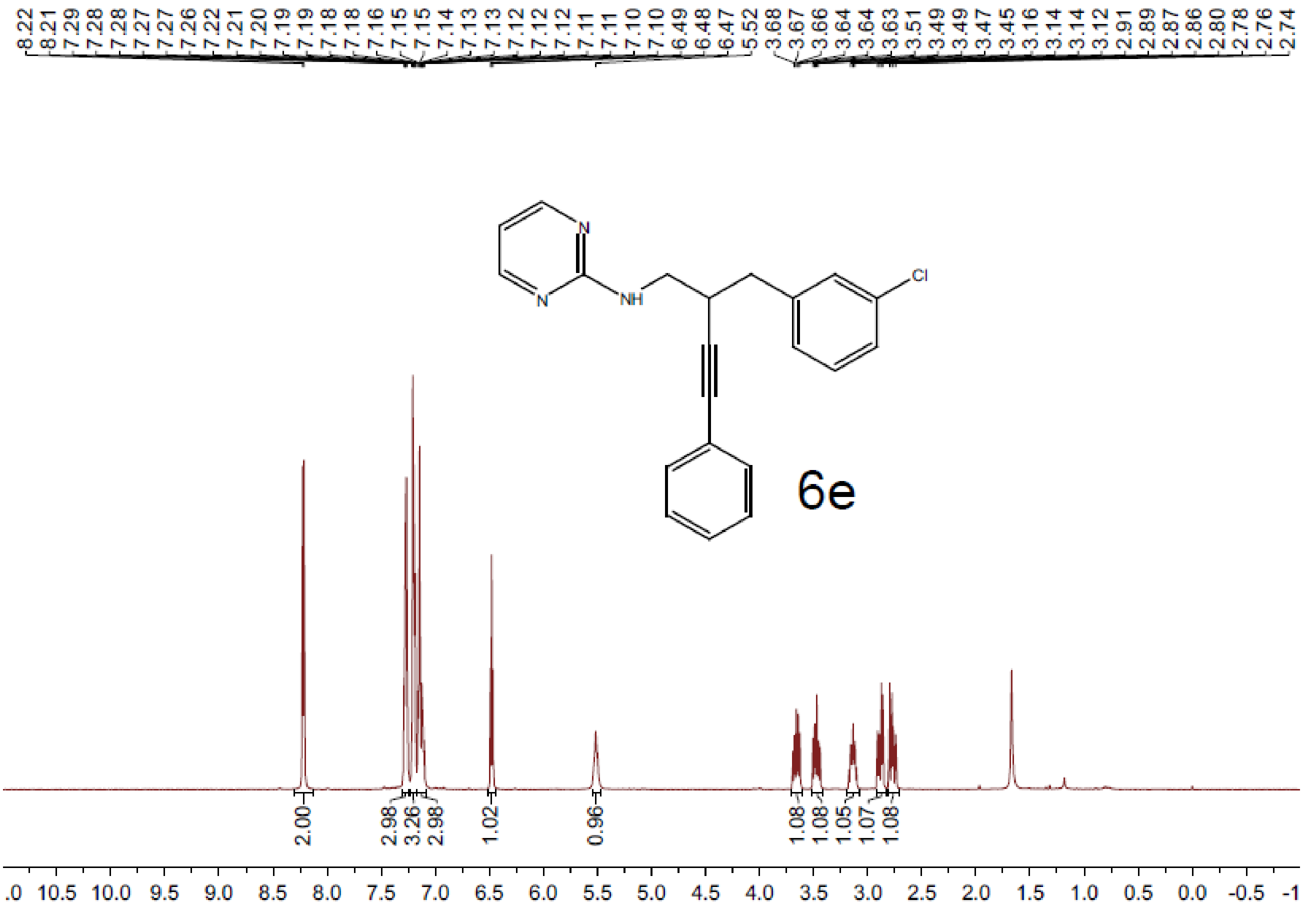
8.22 8.21 7.29 7.28 7.28 7.27 7.27 7.26 7.22 7.21 7.20 7.19 7.19 7.18 7.18 7.16 7.15 7.15 7.14 7.13 7.13 7.12 7.12 7.12 7.11 7.11 7.10 7.10 6.49 6.48 6.47 5.52 3.68 3.67 3.66 3.64 3.64 3.63 3.51 3.49 3.49 3.47 3.45 3.16 3.14 3.14 3.12 2.91 2.89 2.87 2.86 2.80 2.78 2.76 2.74
N
N
NH
Cl
6e
2.00
2.98
3.26
2.98
1.02
0.96
1.08
1.08
1.05
1.07
1.08
.0 10.5 10.0 9.5 9.0 8.5 8.0 7.5 7.0 6.5 6.0 5.5 5.0 4.5 4.0 3.5 3.0 2.5 2.0 1.5 1.0 0.5 0.0 -0.5 -1

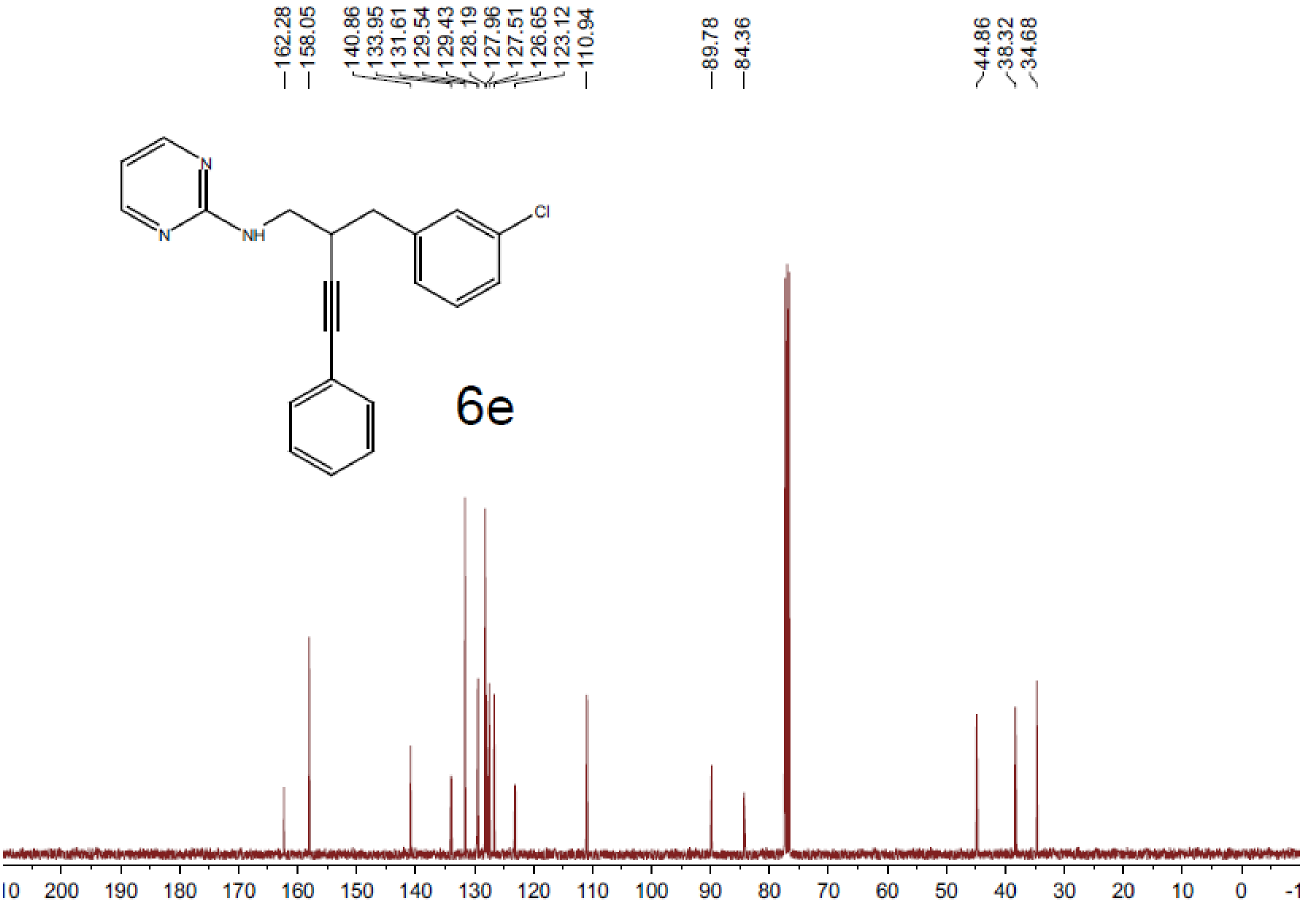
162.28 158.05 140.86 133.95 131.61 129.54 129.43 128.19 127.96 127.51 126.65 123.12 110.94 89.78 84.36 44.86 38.32 34.68
N
N
NH
Cl
6e
10 200 190 180 170 160 150 140 130 120 110 100 90 80 70 60 50 40 30 20 10 0 -1

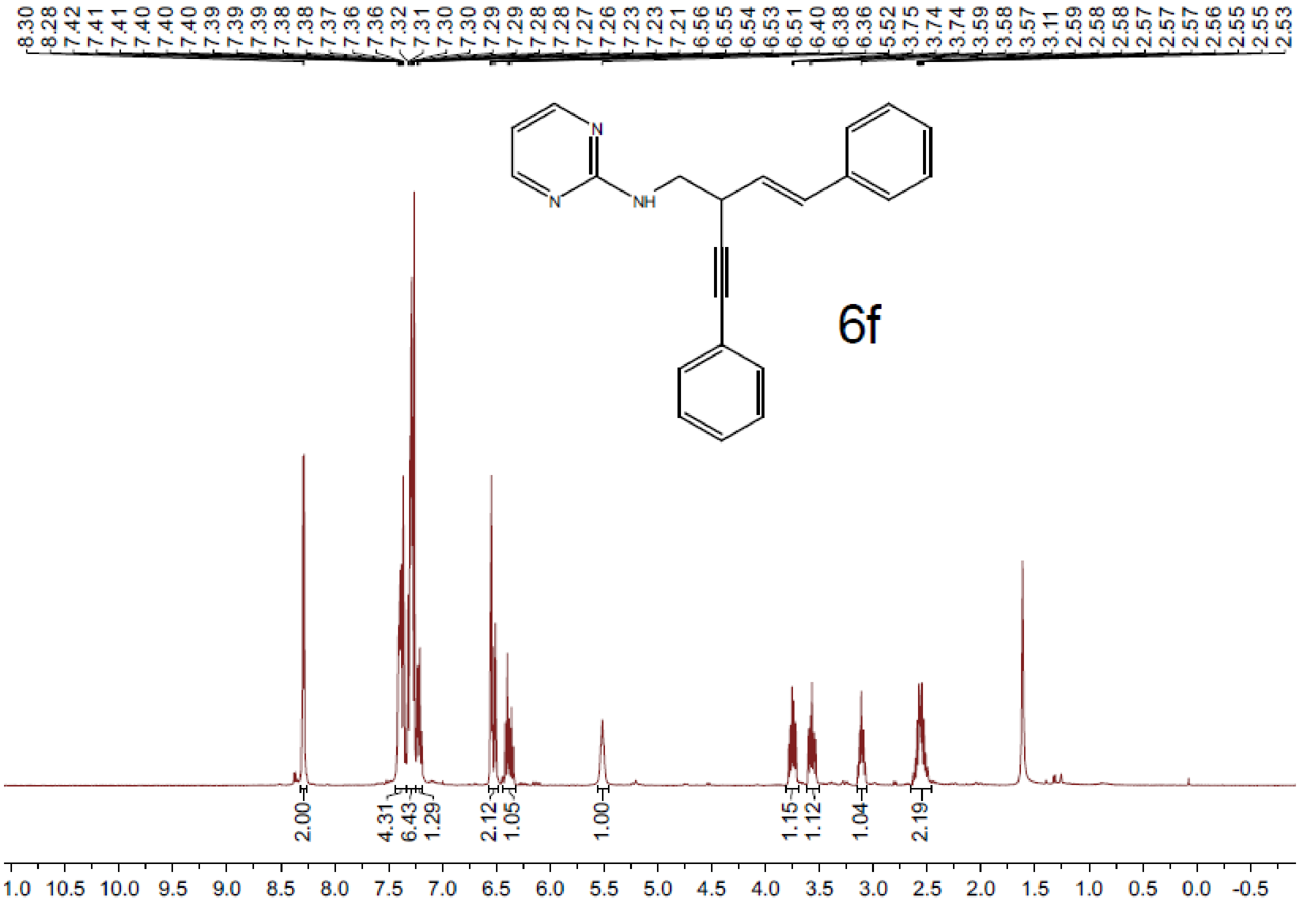
8.30
8.28
7.42
7.41
7.41
7.40
7.40
7.40
7.39
7.39
7.39
7.38
7.38
7.37
7.36
7.36
7.32
7.31
7.30
7.30
7.29
7.29
7.28
7.28
7.27
7.26
7.23
7.23
7.21
6.56
6.55
6.54
6.53
6.51
6.40
6.38
6.36
5.52
3.75
3.74
3.74
3.59
3.58
3.57
3.11
2.59
2.58
2.58
2.57
2.57
2.57
2.56
2.55
2.55
2.53
N
N
NH
6f
2.00
4.31
6.43
1.29
2.12
1.05
1.00
1.15
1.12
1.04
2.19
1.0 10.5 10.0 9.5 9.0 8.5 8.0 7.5 7.0 6.5 6.0 5.5 5.0 4.5 4.0 3.5 3.0 2.5 2.0 1.5 1.0 0.5 0.0 -0.5

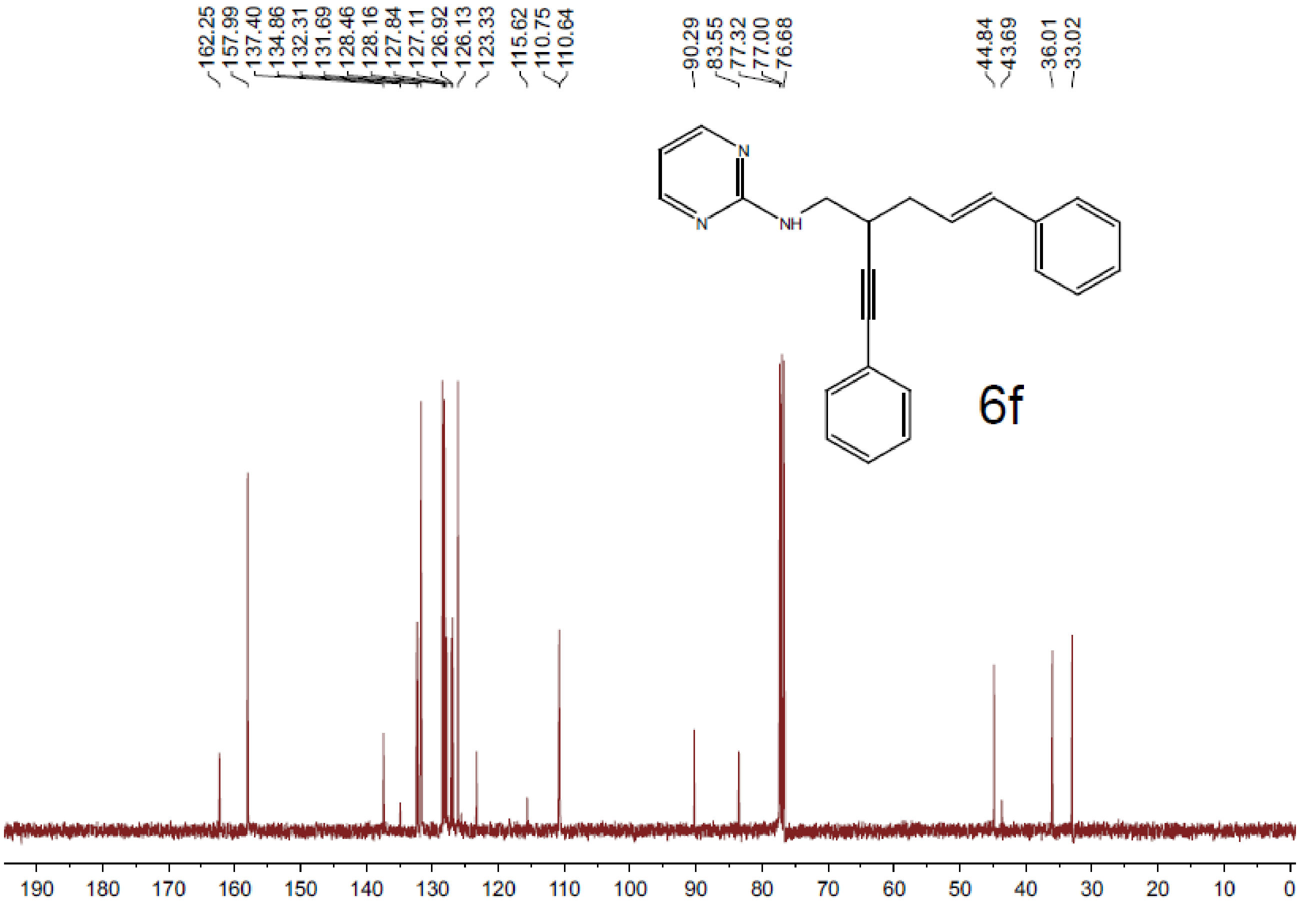
162.25
157.99
137.40
134.86
132.31
131.69
128.46
128.16
127.84
127.11
126.92
126.13
123.33
115.62
110.75
110.64
90.29
83.55
77.32
77.00
76.68
44.84
43.69
36.01
33.02
N
N
NH
6f
190 180 170 160 150 140 130 120 110 100 90 80 70 60 50 40 30 20 10 0

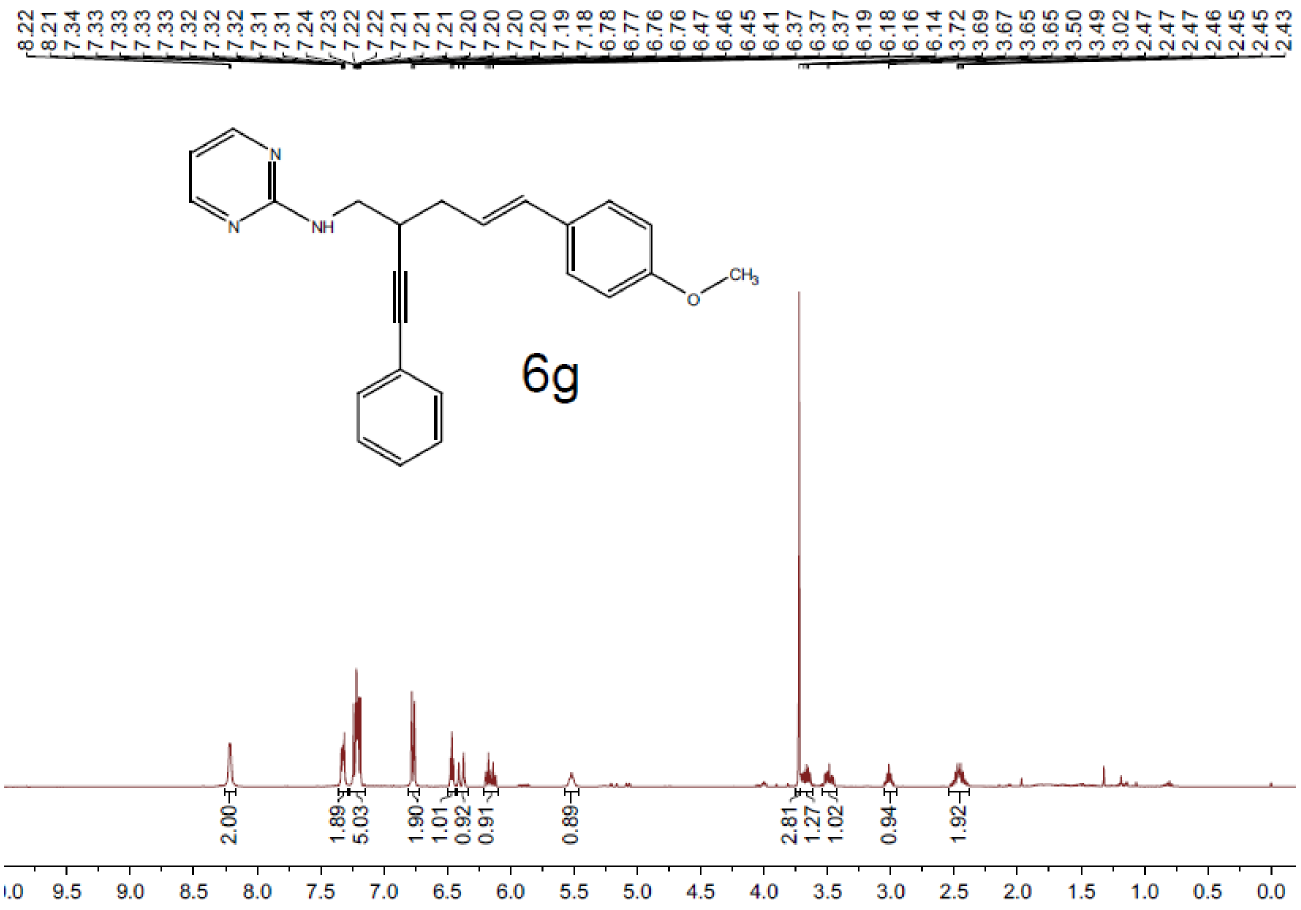
6g

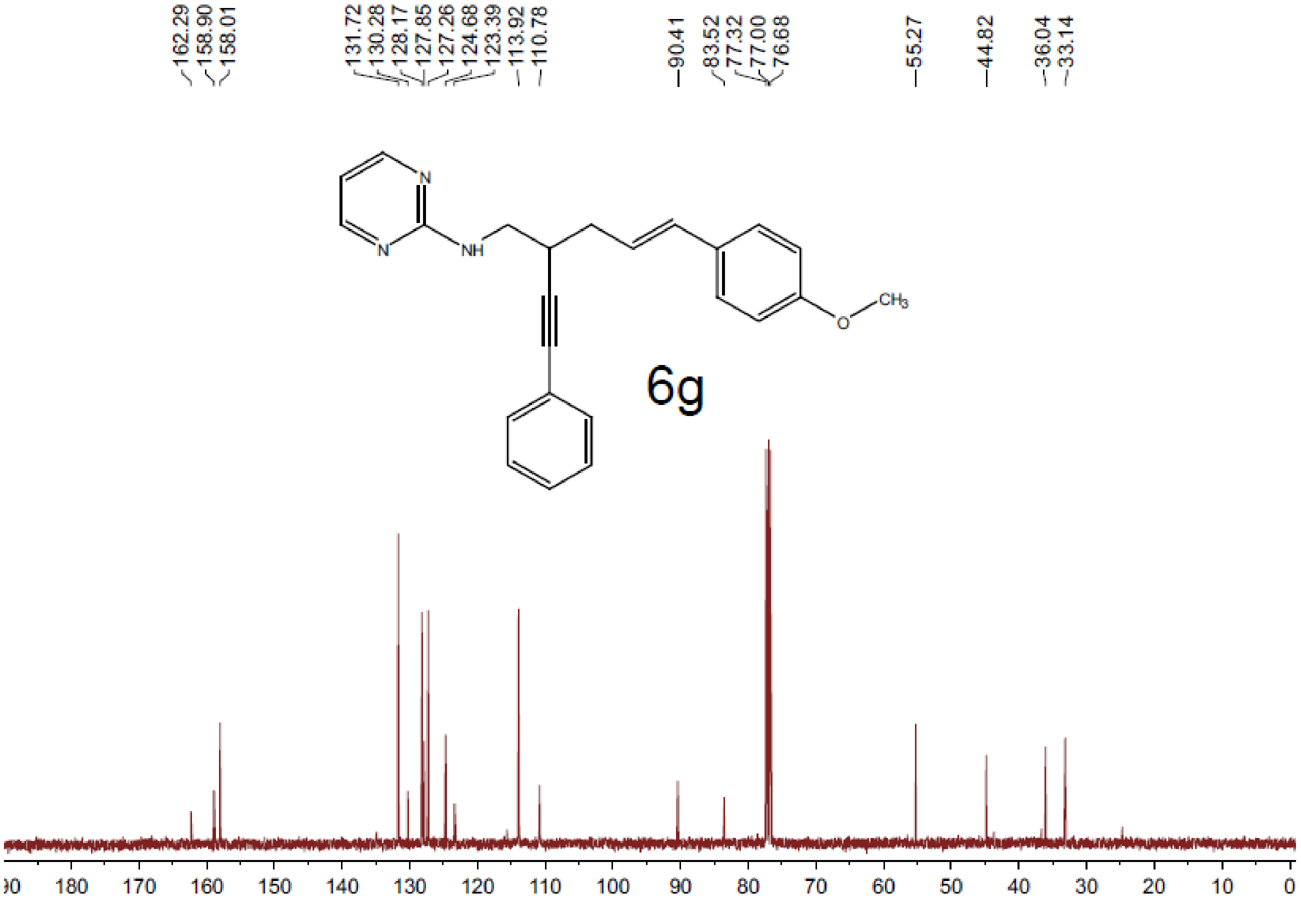
6g

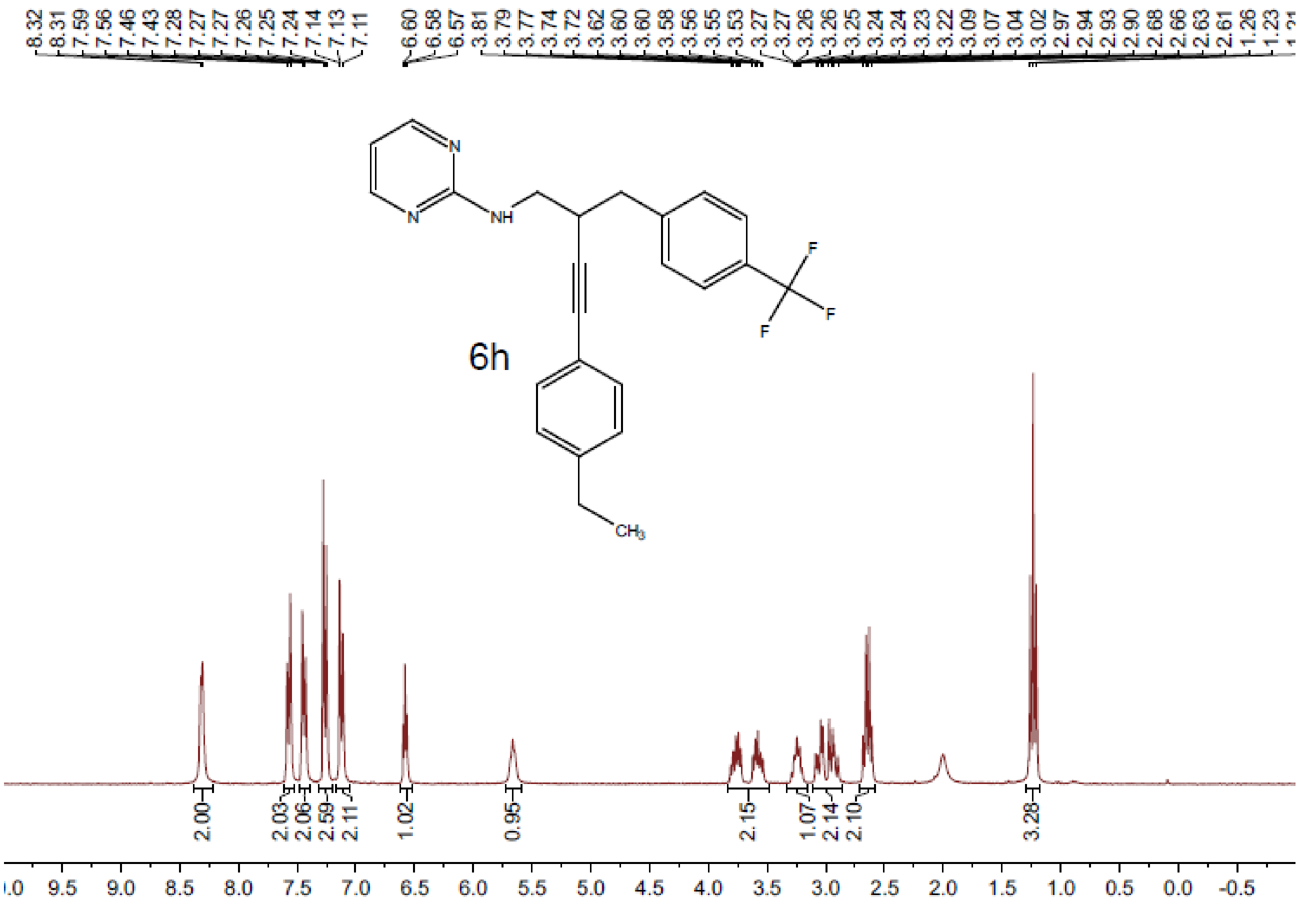
6h
N
NH
F
CH3

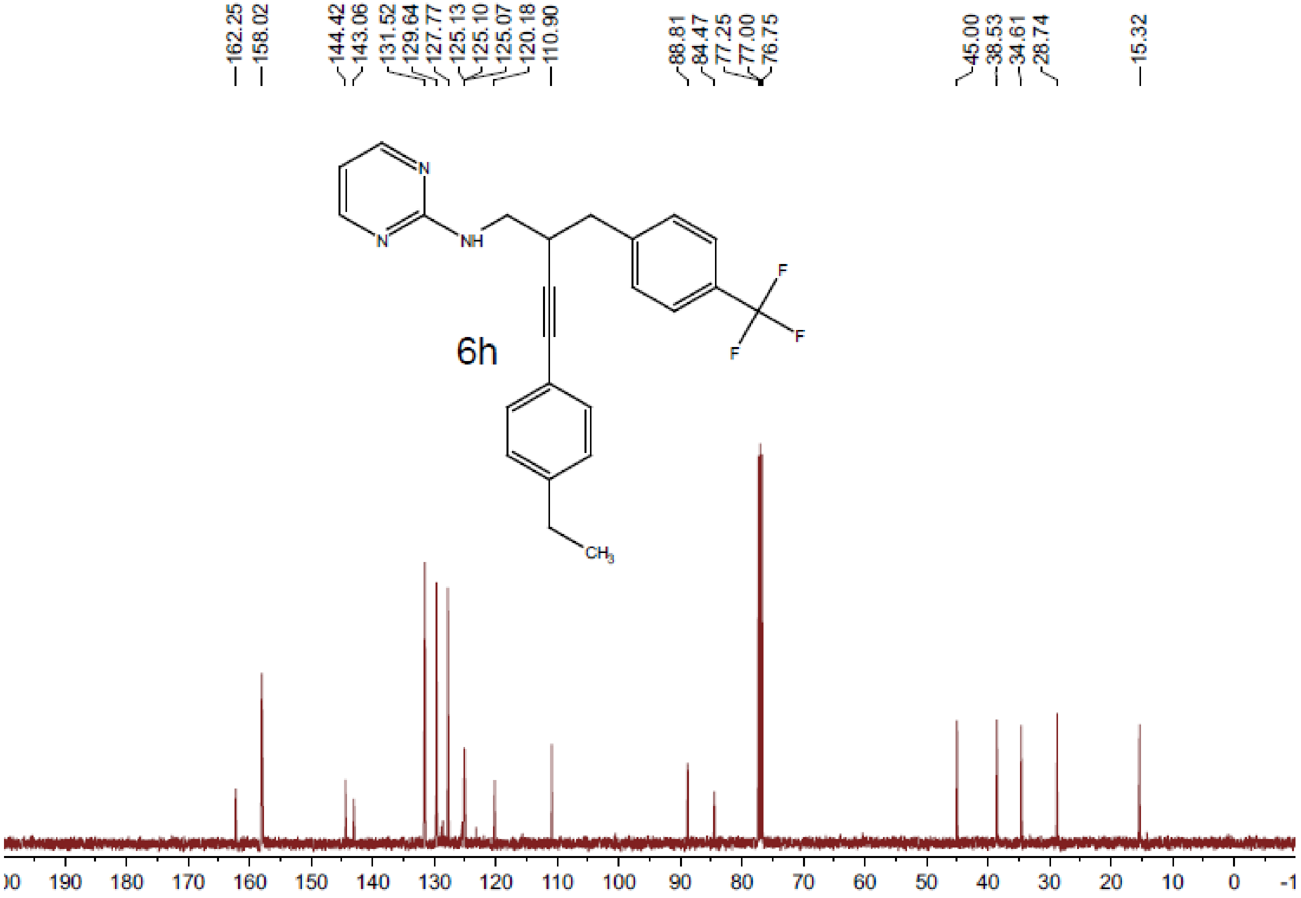
6h
N
NH
F
CH3

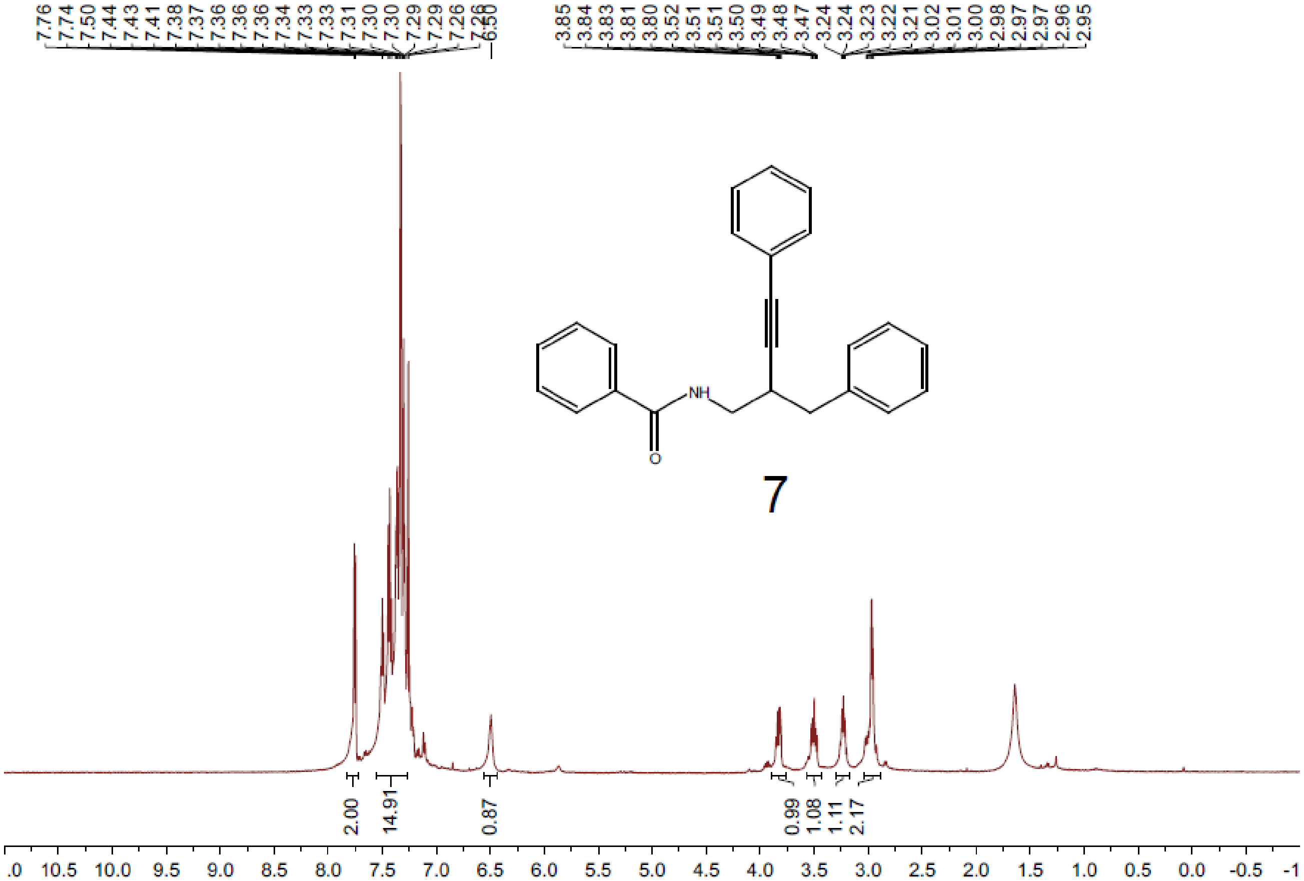
7.76
7.74
7.50
7.44
7.43
7.41
7.38
7.37
7.36
7.36
7.34
7.33
7.33
7.31
7.30
7.30
7.29
7.29
7.26
7.26
6.50
3.85
3.84
3.83
3.81
3.80
3.52
3.51
3.51
3.50
3.49
3.48
3.47
3.24
3.24
3.23
3.22
3.21
3.02
3.01
3.00
2.98
2.97
2.97
2.96
2.95
NH
O
7
2.00
14.91
0.87
0.99
1.08
1.11
2.17
.0 10.5 10.0 9.5 9.0 8.5 8.0 7.5 7.0 6.5 6.0 5.5 5.0 4.5 4.0 3.5 3.0 2.5 2.0 1.5 1.0 0.5 0.0 -0.5 -1

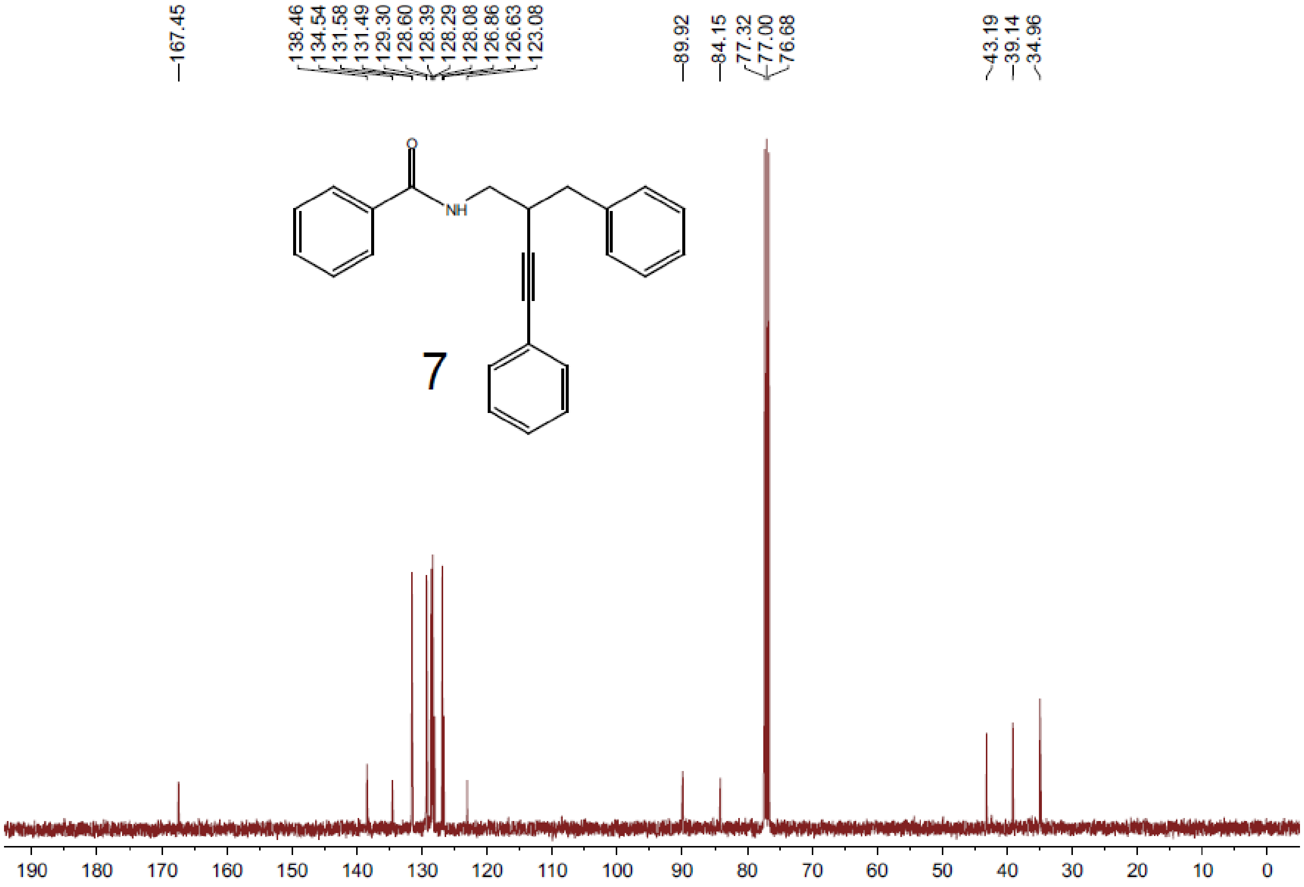
167.45
138.46
134.54
131.58
131.49
129.30
128.60
128.39
128.29
128.08
126.86
126.63
123.08
89.92
84.15
77.32
77.00
76.68
43.19
39.14
34.96
O
NH
7
190 180 170 160 150 140 130 120 110 100 90 80 70 60 50 40 30 20 10 0

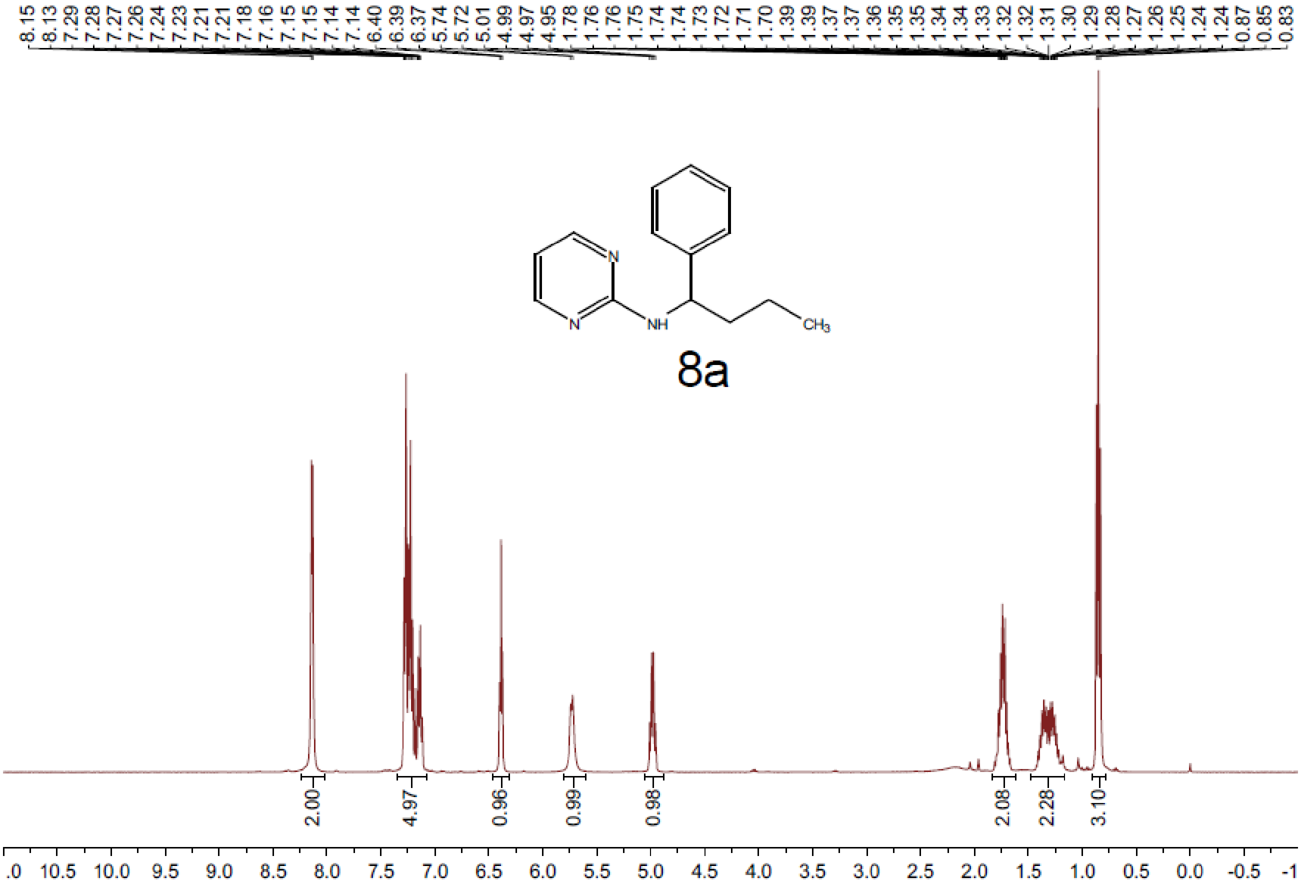
8.15
8.13
7.29
7.28
7.27
7.26
7.24
7.23
7.21
7.21
7.18
7.16
7.15
7.15
7.14
7.14
6.40
6.39
6.37
5.74
5.72
5.01
4.99
4.97
4.95
1.78
1.76
1.76
1.75
1.74
1.74
1.73
1.72
1.71
1.70
1.39
1.39
1.37
1.37
1.36
1.35
1.35
1.34
1.34
1.33
1.32
1.32
1.31
1.30
1.29
1.28
1.27
1.26
1.25
1.24
1.24
0.87
0.85
0.83
N
N
NH
CH3
8a
2.00
4.97
0.96
0.99
0.98
2.08
2.28
3.10

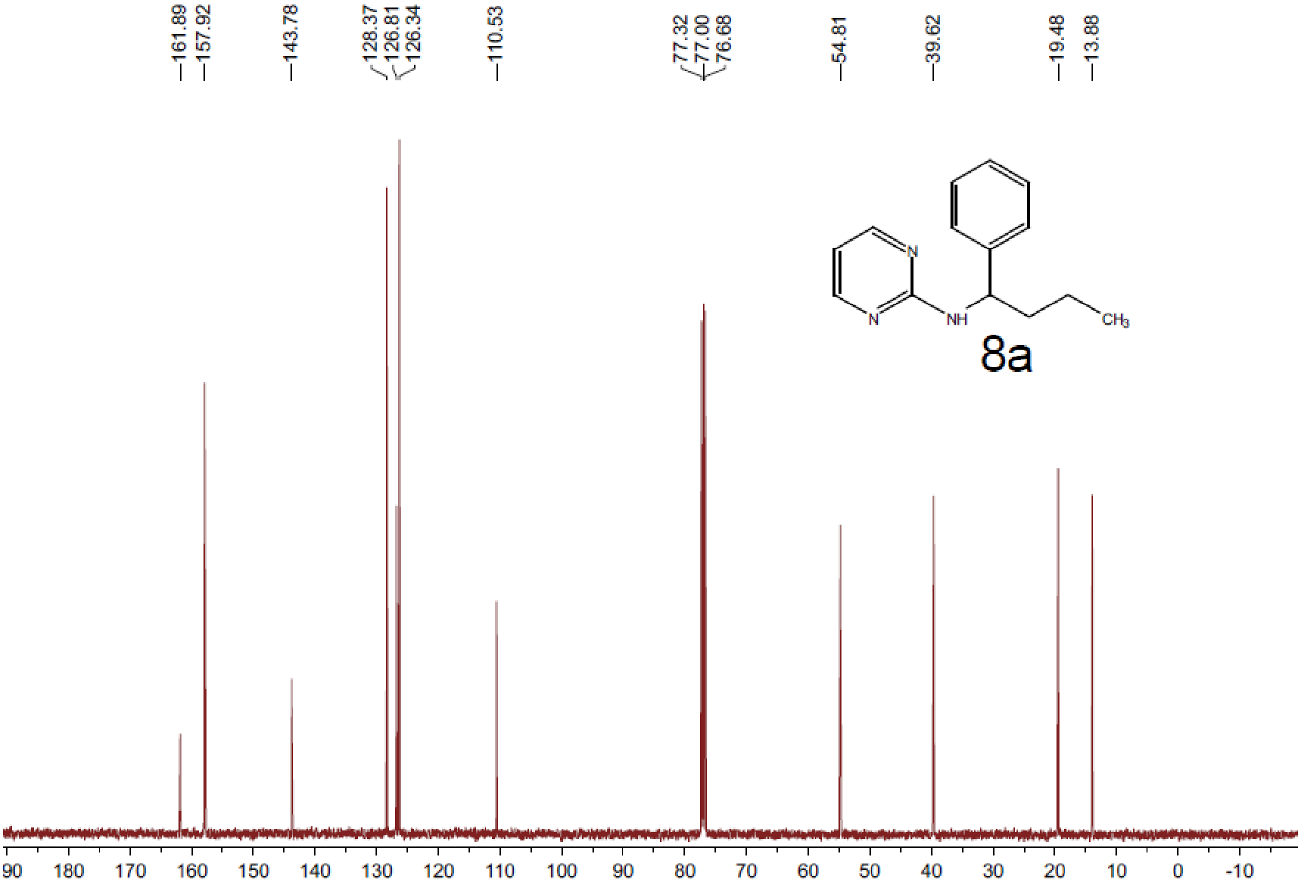
161.89
157.92
143.78
128.37
126.81
126.34
110.53
77.32
77.00
76.68
54.81
39.62
19.48
13.88
N
N
NH
CH3
8a

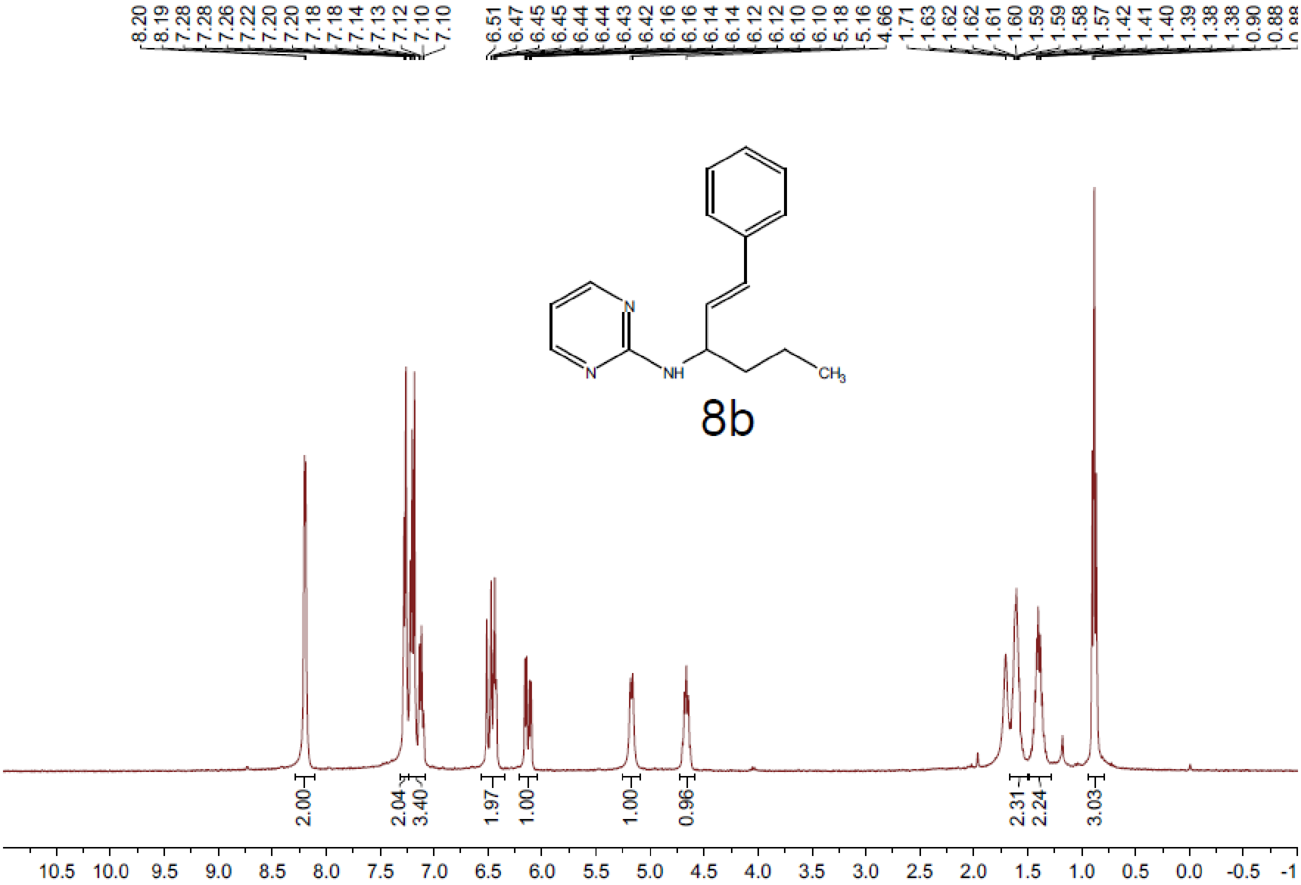
8.20 8.19 7.28 7.26 7.22 7.20 7.20 7.18 7.18 7.14 7.13 7.12 7.10 7.10
6.51 6.47 6.45 6.45 6.44 6.44 6.43 6.42 6.16 6.16 6.14 6.14 6.12 6.12 6.10 6.10 5.18 5.16 4.66
1.71 1.63 1.62 1.62 1.61 1.60 1.59 1.59 1.58 1.57 1.42 1.41 1.40 1.39 1.38 1.38 0.90 0.88 [illegible]
N
N
NH
CH3
8b
2.00 2.04 3.40 1.97 1.00 1.00 0.96 2.31 2.24 3.03
10.5 10.0 9.5 9.0 8.5 8.0 7.5 7.0 6.5 6.0 5.5 5.0 4.5 4.0 3.5 3.0 2.5 2.0 1.5 1.0 0.5 0.0 -0.5 -1

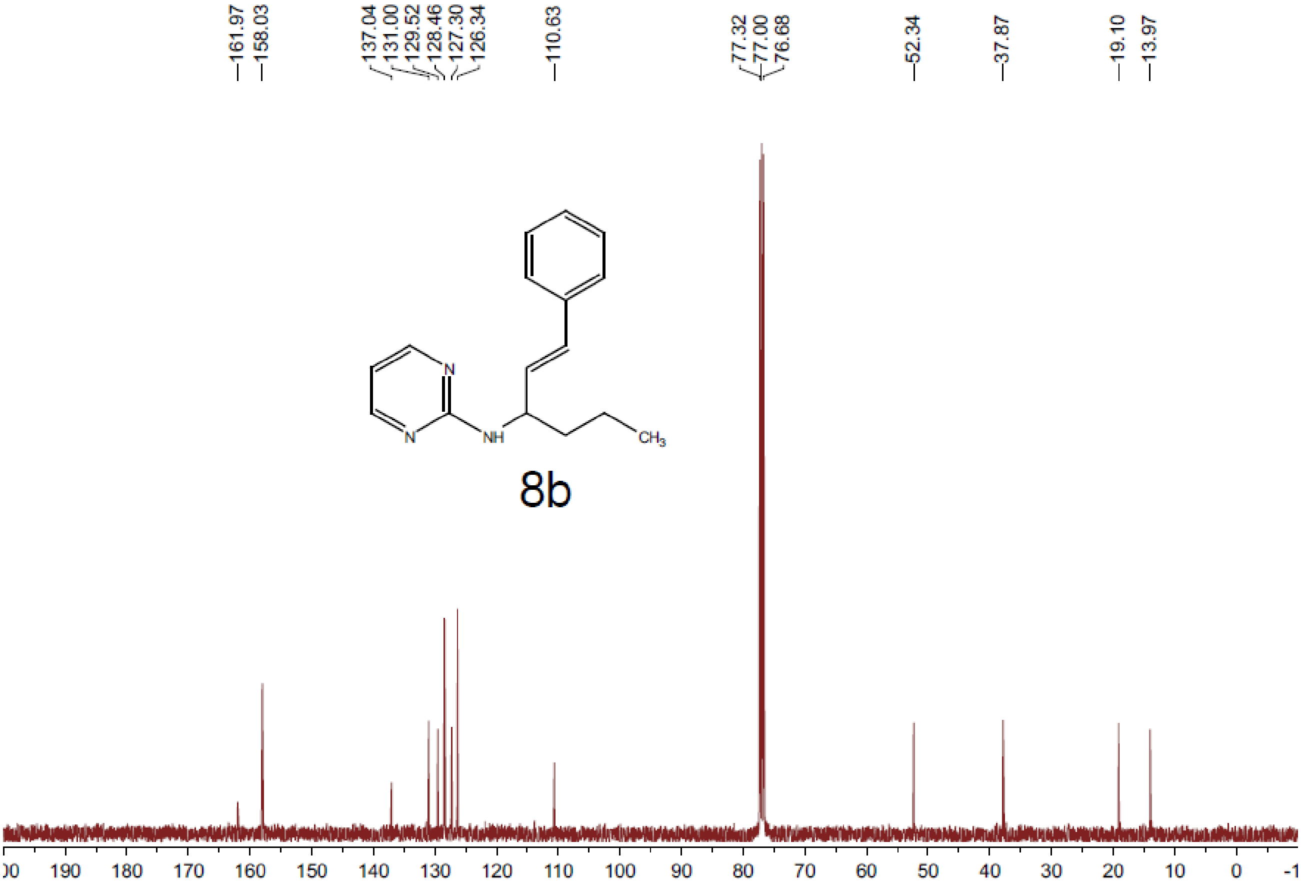
161.97 158.03
137.04 131.00 129.52 128.46 127.30 126.34
110.63
77.32 77.00 76.68
52.34
37.87
19.10 13.97
N
N
NH
CH3
8b
190 180 170 160 150 140 130 120 110 100 90 80 70 60 50 40 30 20 10 0 -1

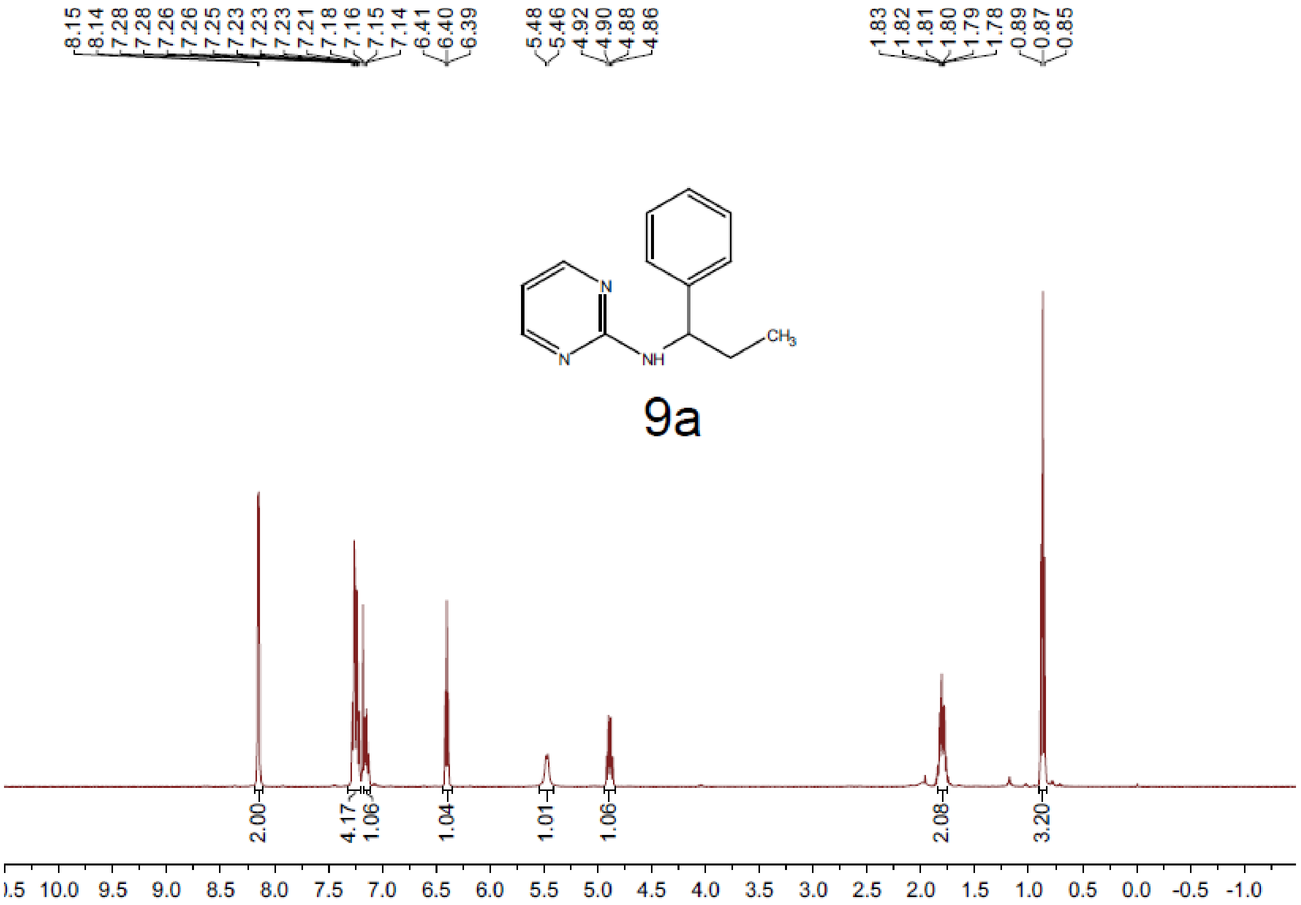
8.15
8.14
7.28
7.28
7.26
7.26
7.25
7.23
7.23
7.23
7.21
7.18
7.16
7.15
7.14
6.41
6.40
6.39
5.48
5.46
4.92
4.90
4.88
4.86
1.83
1.82
1.81
1.80
1.79
1.78
0.89
0.87
0.85
N
N
NH
CH3
9a
2.00
4.17
1.06
1.04
1.01
1.06
2.08
3.20
10.0 9.5 9.0 8.5 8.0 7.5 7.0 6.5 6.0 5.5 5.0 4.5 4.0 3.5 3.0 2.5 2.0 1.5 1.0 0.5 0.0 -0.5 -1.0

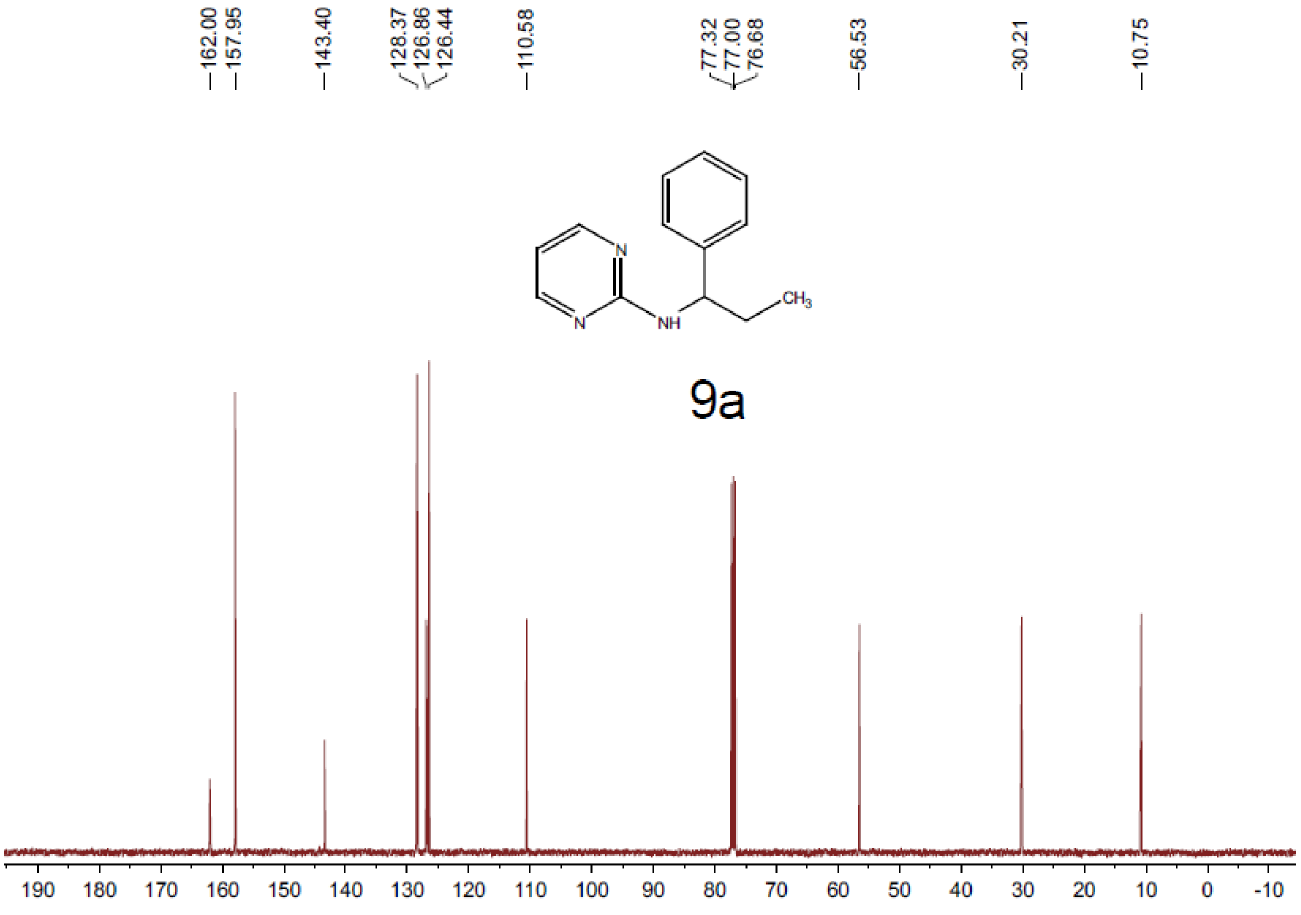
162.00
157.95
143.40
128.37
126.86
126.44
110.58
77.32
77.00
76.68
56.53
30.21
10.75
N
N
NH
CH3
9a
190 180 170 160 150 140 130 120 110 100 90 80 70 60 50 40 30 20 10 0 -10

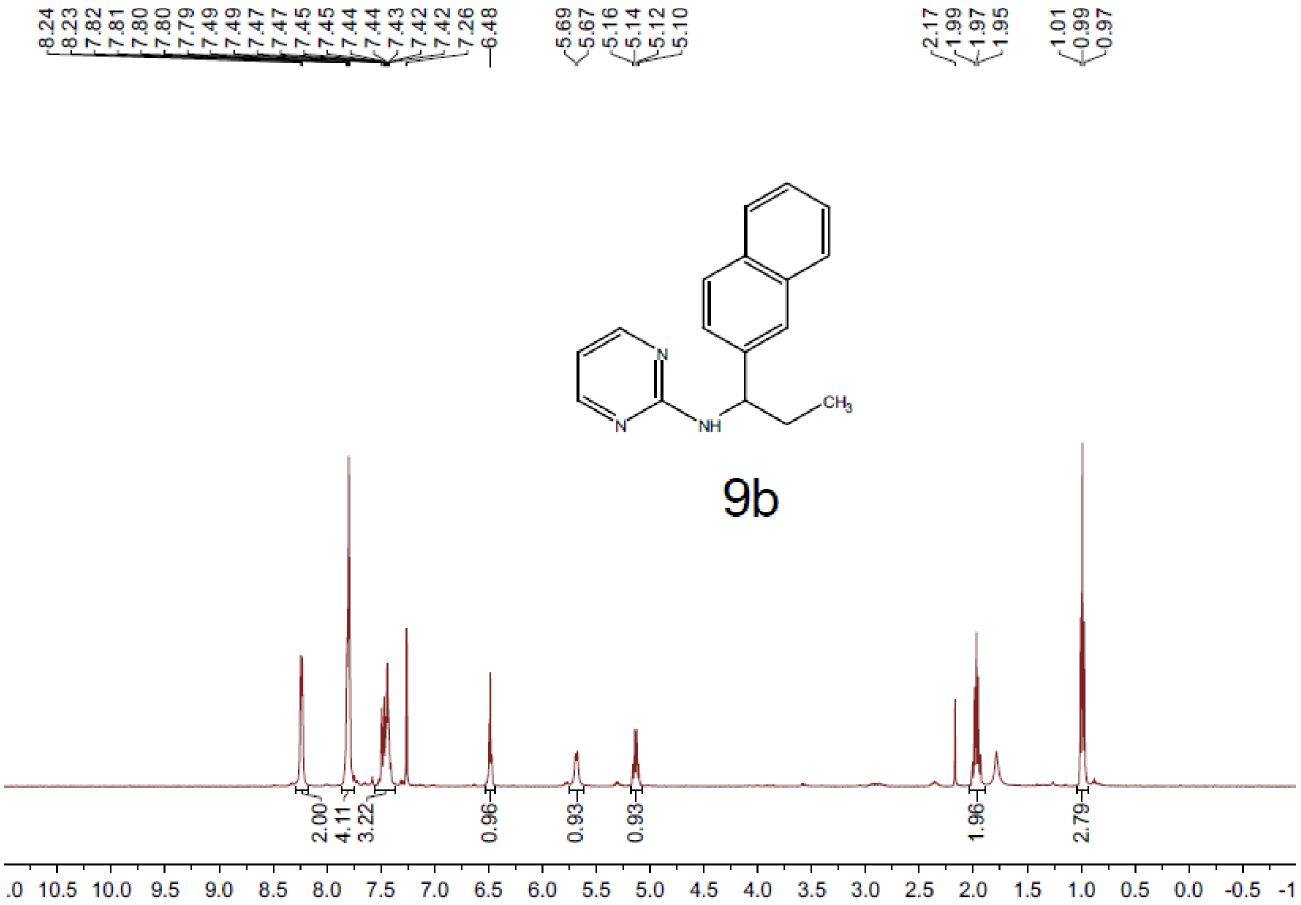
8.24
8.23
7.82
7.81
7.80
7.80
7.79
7.49
7.49
7.47
7.47
7.45
7.45
7.44
7.44
7.43
7.42
7.42
7.26
6.48
5.69
5.67
5.16
5.14
5.12
5.10
2.17
1.99
1.97
1.95
1.01
0.99
0.97
N
N
NH
CH3
9b
2.00
4.11
3.22
0.96
0.93
0.93
1.96
2.79
.0 10.5 10.0 9.5 9.0 8.5 8.0 7.5 7.0 6.5 6.0 5.5 5.0 4.5 4.0 3.5 3.0 2.5 2.0 1.5 1.0 0.5 0.0 -0.5 -1

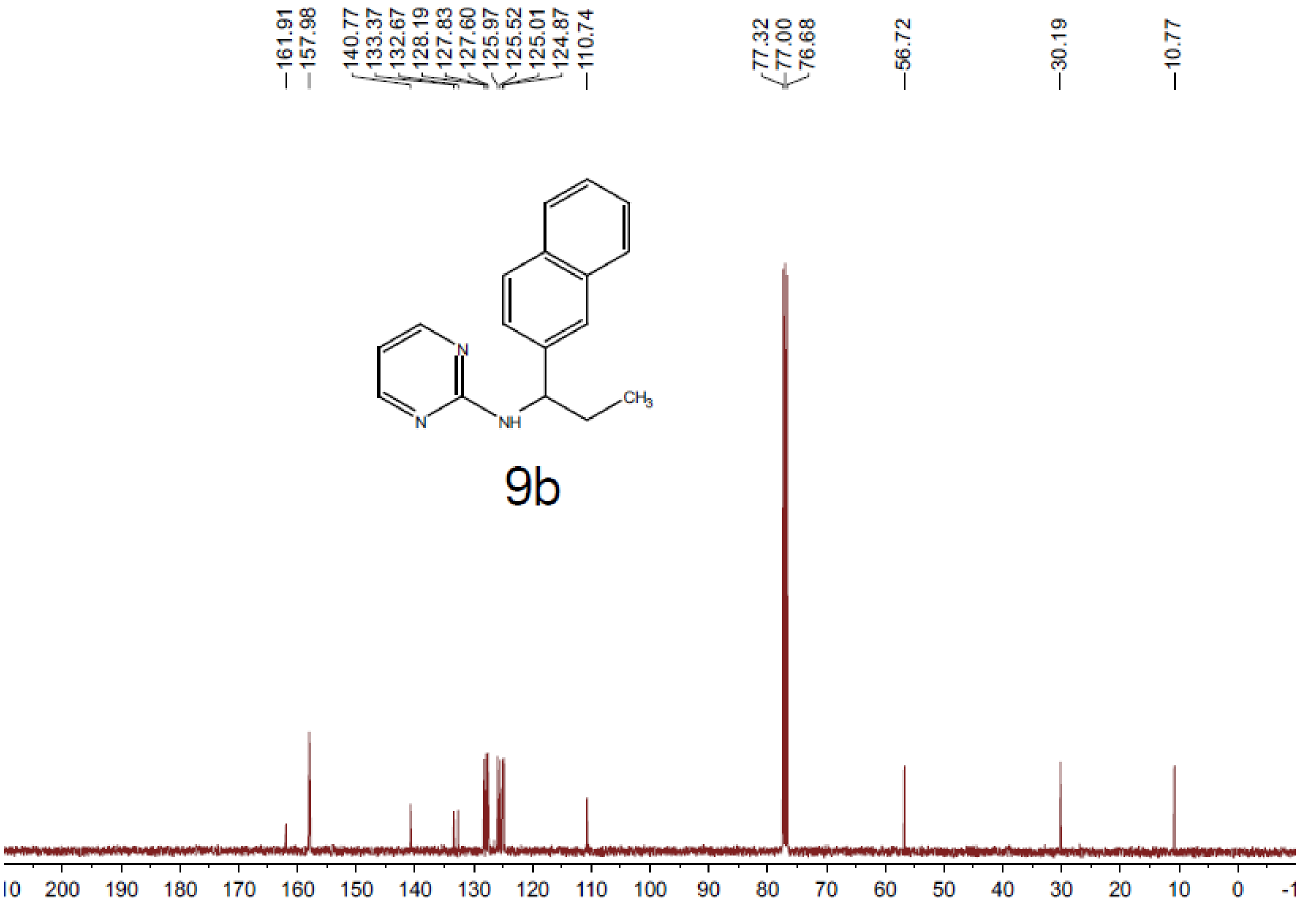
161.91
157.98
140.77
133.37
132.67
128.19
127.83
127.60
125.97
125.52
125.01
124.87
110.74
77.32
77.00
76.68
56.72
30.19
10.77
N
N
NH
CH3
9b
10 200 190 180 170 160 150 140 130 120 110 100 90 80 70 60 50 40 30 20 10 0 -1

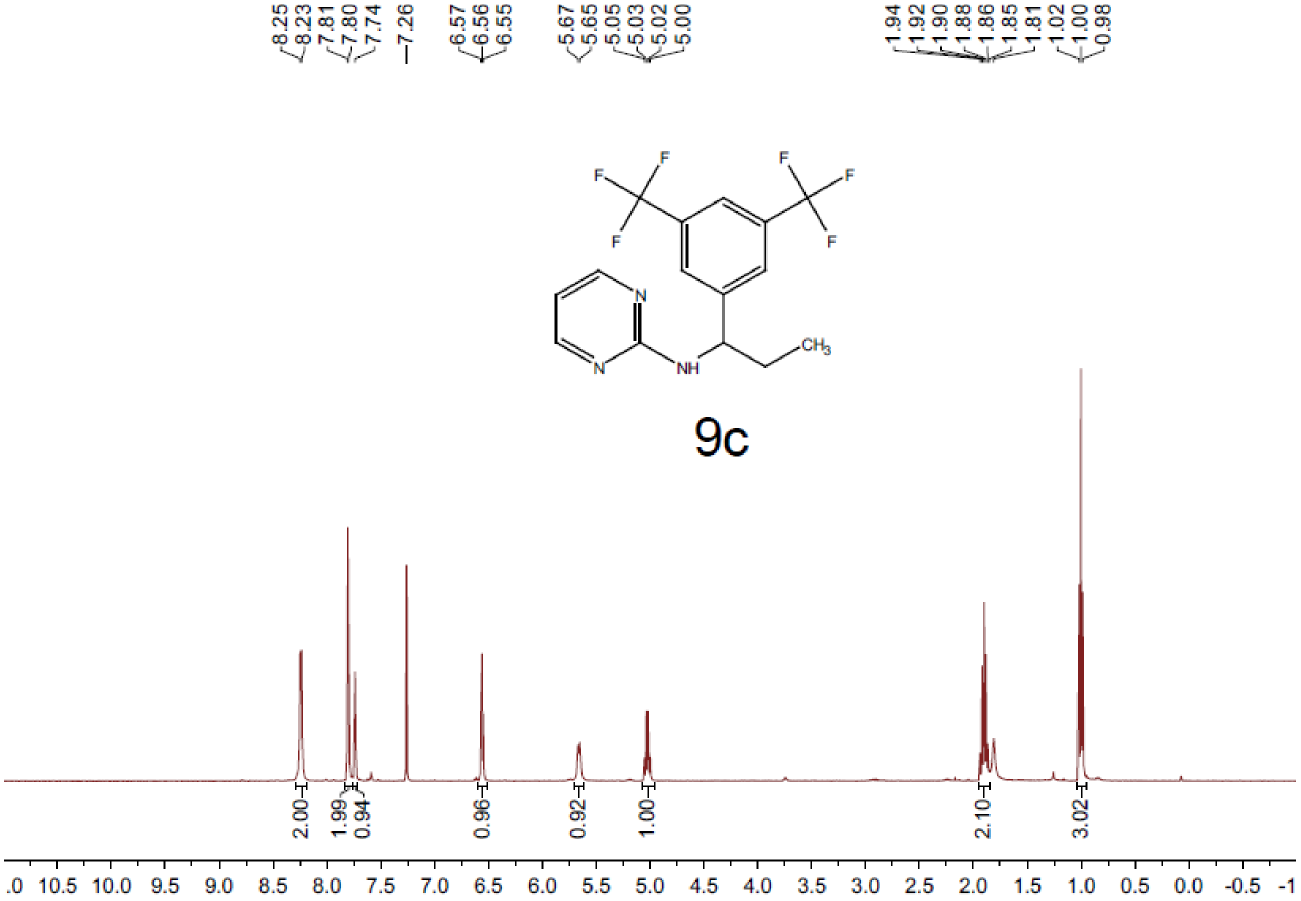
8.25
8.23
7.81
7.80
7.74
7.26
6.57
6.56
6.55
5.67
5.65
5.05
5.03
5.02
5.00
1.94
1.92
1.90
1.88
1.86
1.85
1.81
1.02
1.00
0.98
9c
2.00
1.99
0.94
0.96
0.92
1.00
2.10
3.02
.0 10.5 10.0 9.5 9.0 8.5 8.0 7.5 7.0 6.5 6.0 5.5 5.0 4.5 4.0 3.5 3.0 2.5 2.0 1.5 1.0 0.5 0.0 -0.5 -1

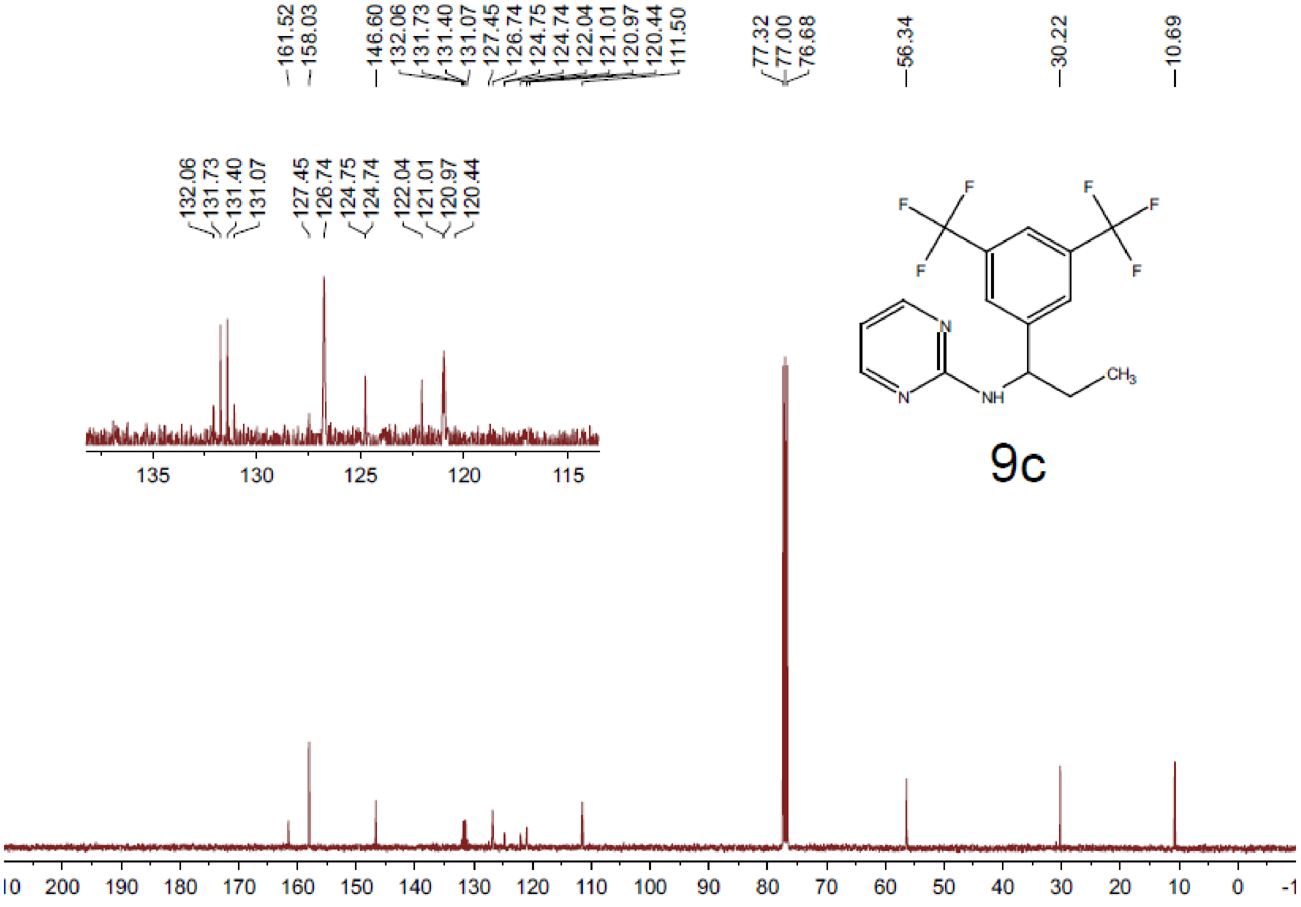
161.52
158.03
146.60
132.06
131.73
131.40
131.07
127.45
126.74
124.75
124.74
122.04
121.01
120.97
120.44
111.50
77.32
77.00
76.68
56.34
30.22
10.69
135 130 125 120 115
9c
10 200 190 180 170 160 150 140 130 120 110 100 90 80 70 60 50 40 30 20 10 0 -1

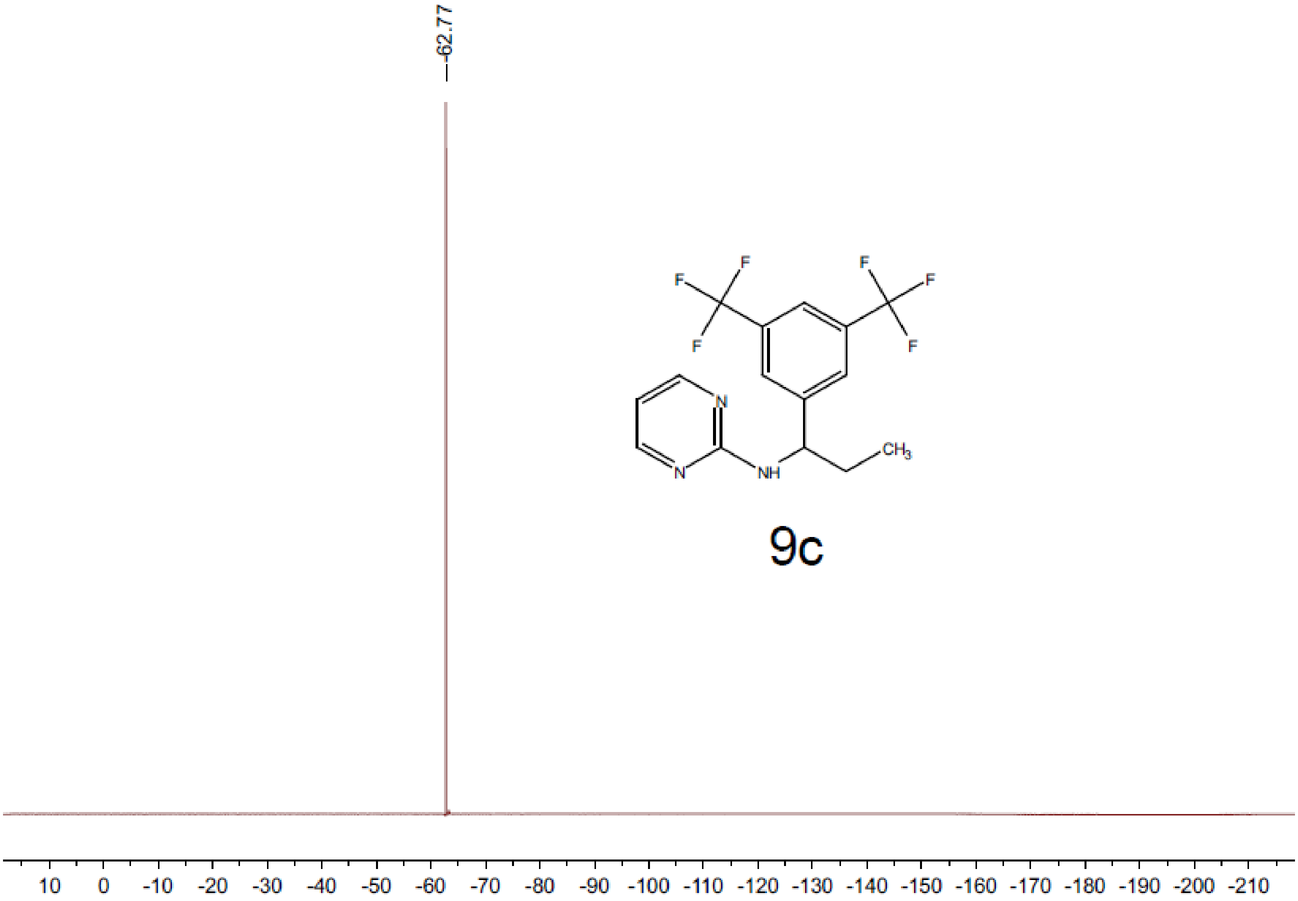

9c

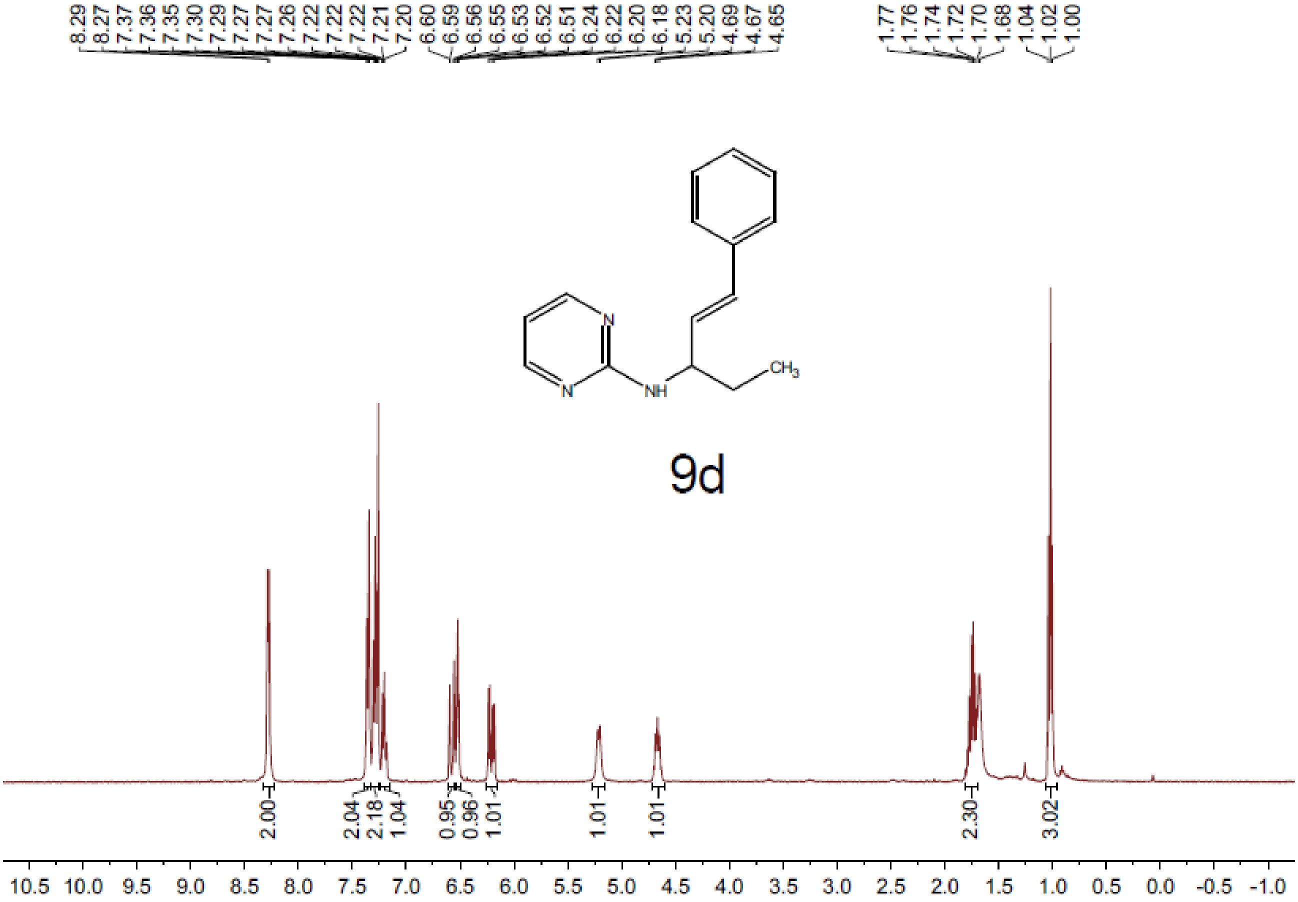

9d

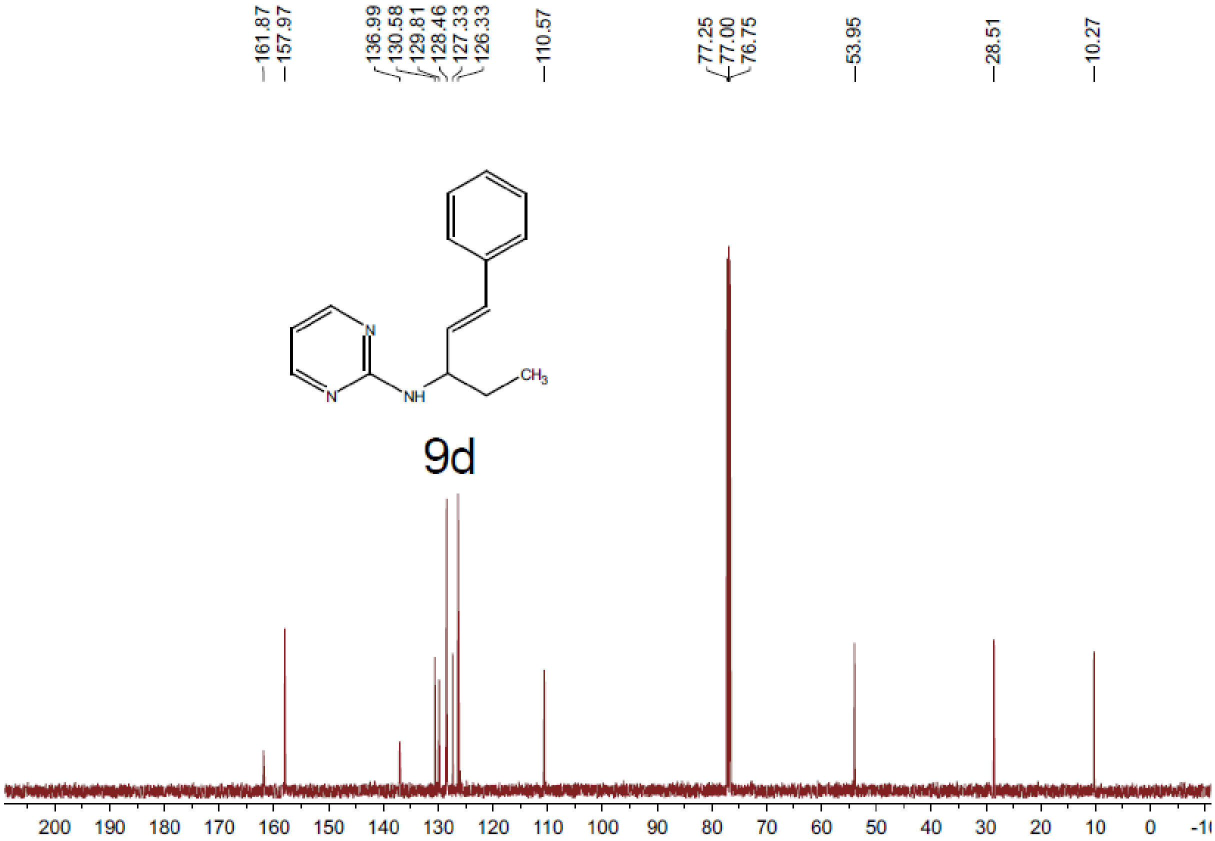
161.87
157.97
136.99
130.58
129.81
128.46
127.33
126.33
110.57
77.25
77.00
76.75
53.95
28.51
10.27
N
N
NH
CH_3
9d
200 190 180 170 160 150 140 130 120 110 100 90 80 70 60 50 40 30 20 10 0

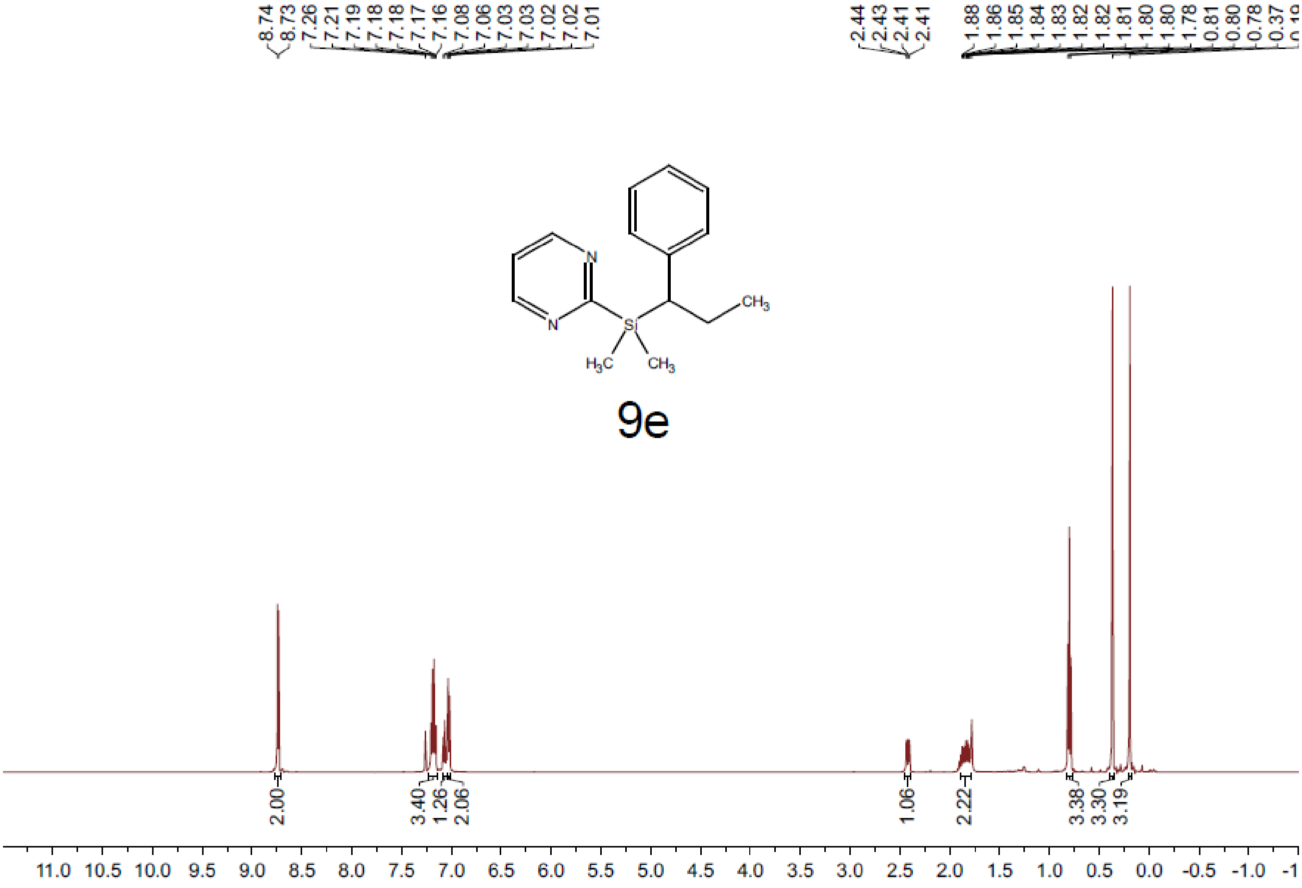
8.74
8.73
7.26
7.21
7.19
7.18
7.18
7.17
7.16
7.08
7.06
7.03
7.03
7.02
7.02
7.01
2.44
2.43
2.41
2.41
1.88
1.86
1.85
1.84
1.83
1.82
1.82
1.81
1.80
1.80
1.78
0.81
0.80
0.78
0.37
N
N
Si
CH_3
H_3C
CH_3
9e
2.00
3.40
1.26
2.06
1.06
2.22
3.38
3.30
3.19
11.0 10.5 10.0 9.5 9.0 8.5 8.0 7.5 7.0 6.5 6.0 5.5 5.0 4.5 4.0 3.5 3.0 2.5 2.0 1.5 1.0 0.5 0.0 -0.5 -1.0

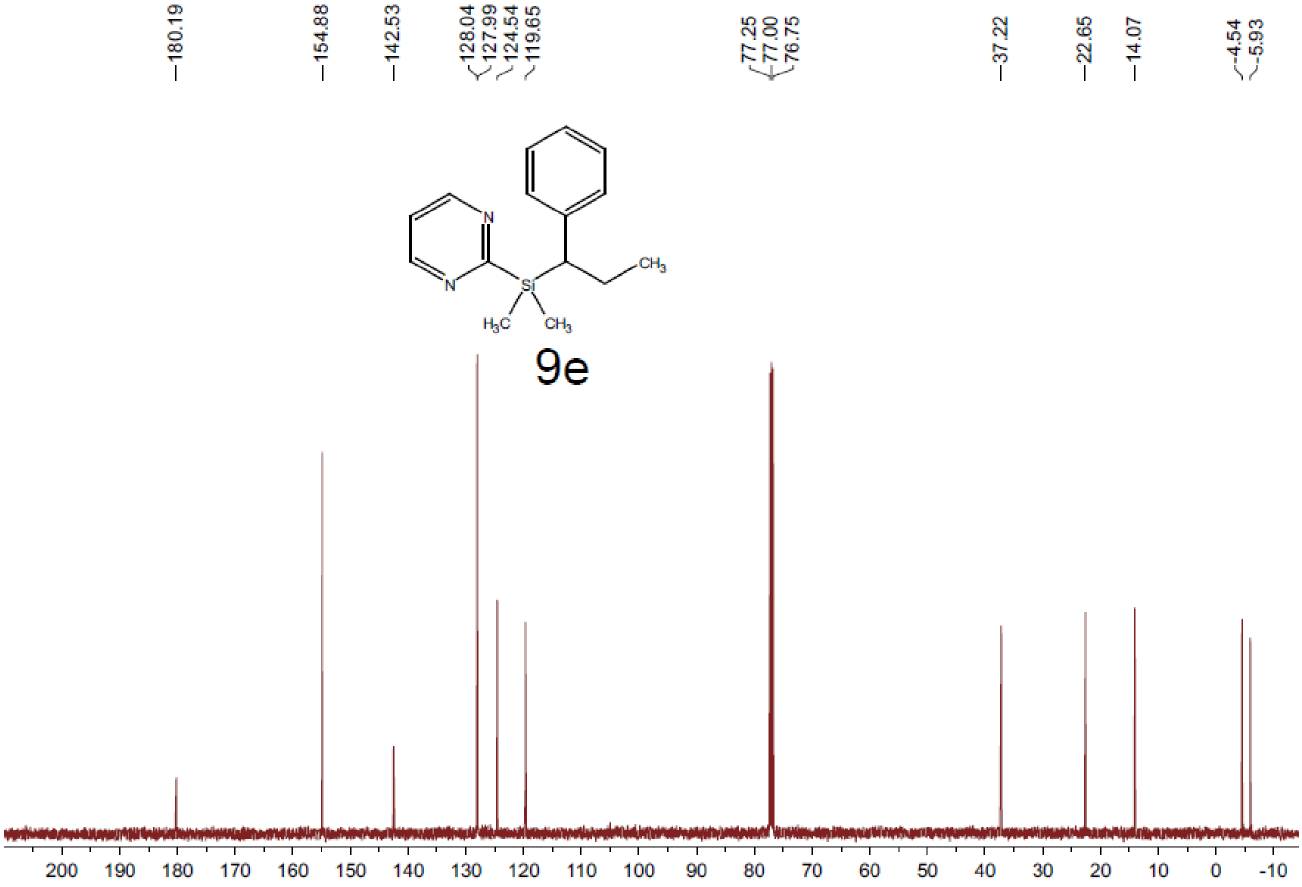
180.19
154.88
142.53
128.04
127.99
124.54
119.65
77.25
77.00
76.75
37.22
22.65
14.07
-4.54
-5.93
9e
200 190 180 170 160 150 140 130 120 110 100 90 80 70 60 50 40 30 20 10 0 -10

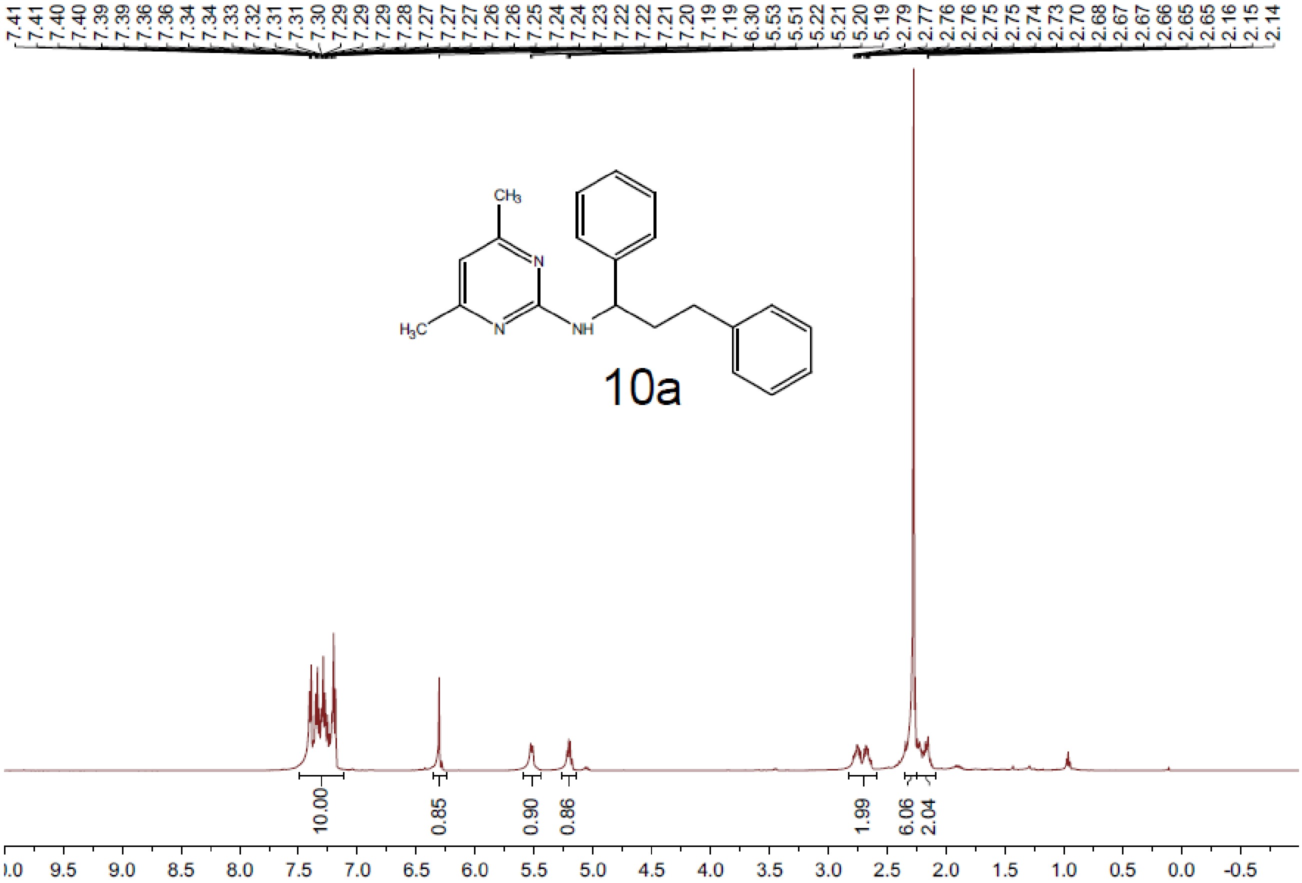
7.41 7.41 7.40 7.40 7.39 7.39 7.36 7.36 7.34 7.34 7.33 7.32 7.31 7.31 7.30 7.29 7.29 7.29 7.28 7.27 7.27 7.26 7.26 7.25 7.24 7.24 7.23 7.22 7.22 7.21 7.20 7.19 7.19 6.30 5.53 5.51 5.22 5.21 5.20 5.19 2.79 2.77 2.76 2.75 2.75 2.74 2.73 2.70 2.68 2.67 2.67 2.66 2.65 2.65 2.16 2.15 2.14
10a
10.00
0.85
0.90
0.86
1.99
6.06
2.04
9.5 9.0 8.5 8.0 7.5 7.0 6.5 6.0 5.5 5.0 4.5 4.0 3.5 3.0 2.5 2.0 1.5 1.0 0.5 0.0 -0.5

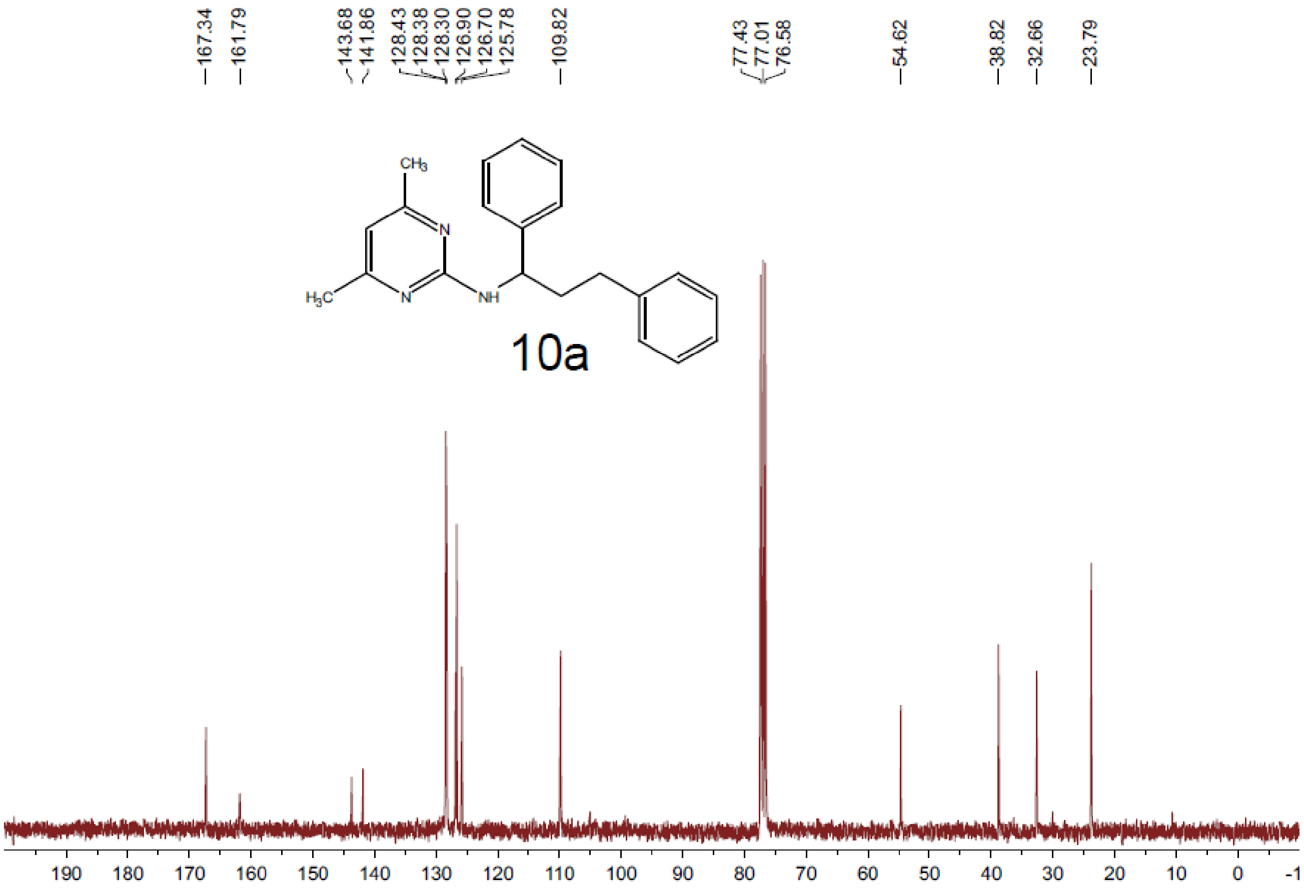
167.34
161.79
143.68
141.86
128.43
128.38
128.30
126.90
126.70
125.78
109.82
77.43
77.01
76.58
54.62
38.82
32.66
23.79
CH3
N
H3C
N
NH
10a
190 180 170 160 150 140 130 120 110 100 90 80 70 60 50 40 30 20 10 0 -1

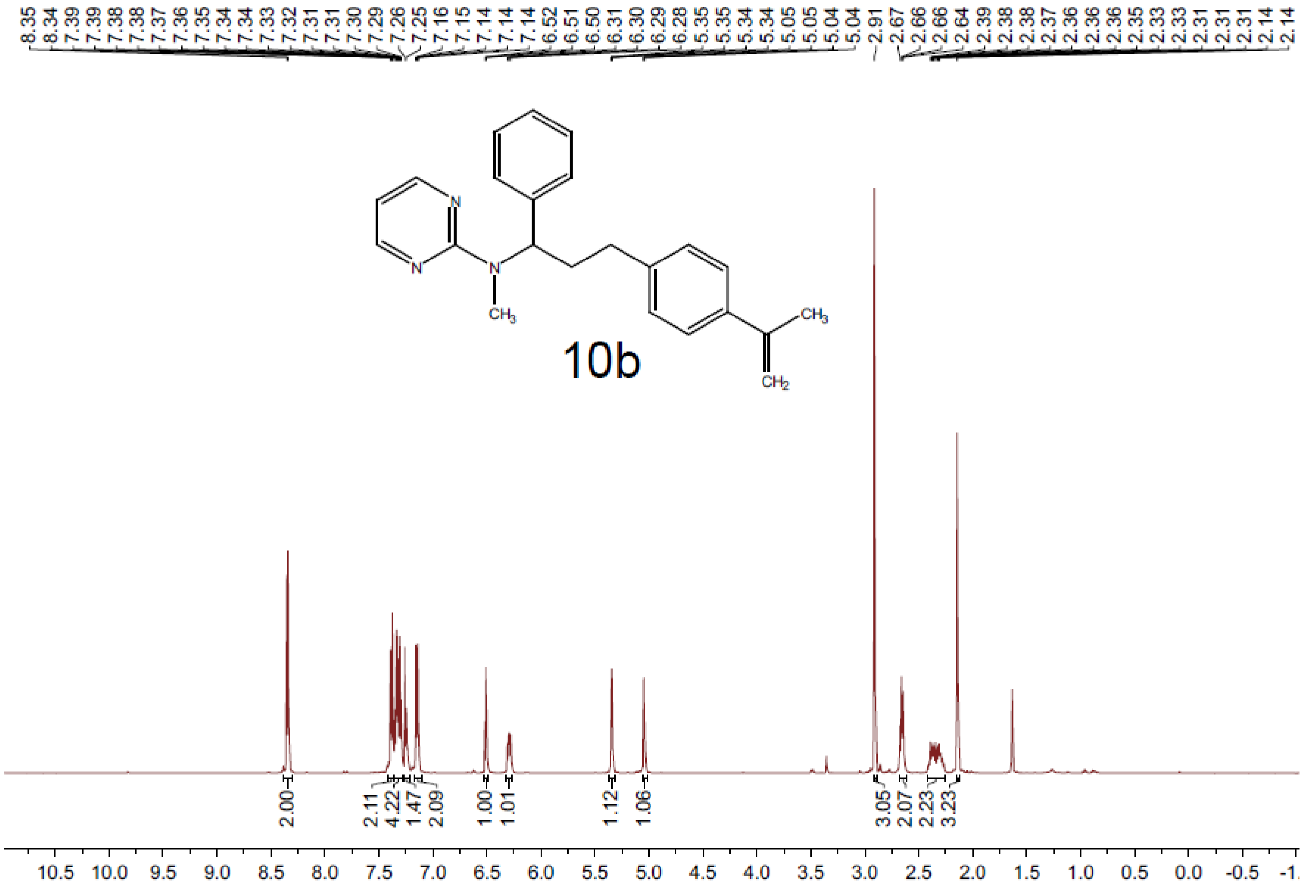
8.35 8.34 7.39 7.39 7.38 7.38 7.37 7.36 7.35 7.34 7.34 7.33 7.32 7.31 7.31 7.30 7.29 7.26 7.25 7.16 7.15 7.14 7.14 6.52 6.51 6.50 6.31 6.30 6.29 6.28 5.35 5.35 5.34 5.34 5.05 5.04 5.04 2.91 2.67 2.66 2.66 2.64 2.39 2.38 2.38 2.37 2.36 2.36 2.35 2.33 2.33 2.31 2.31 2.14 2.14
N
N
N
CH3
CH3
CH2
10b
2.00
2.11
4.22
1.47
2.09
1.00
1.01
1.12
1.06
3.05
2.07
2.23
3.23
10.5 10.0 9.5 9.0 8.5 8.0 7.5 7.0 6.5 6.0 5.5 5.0 4.5 4.0 3.5 3.0 2.5 2.0 1.5 1.0 0.5 0.0 -0.5 -1.

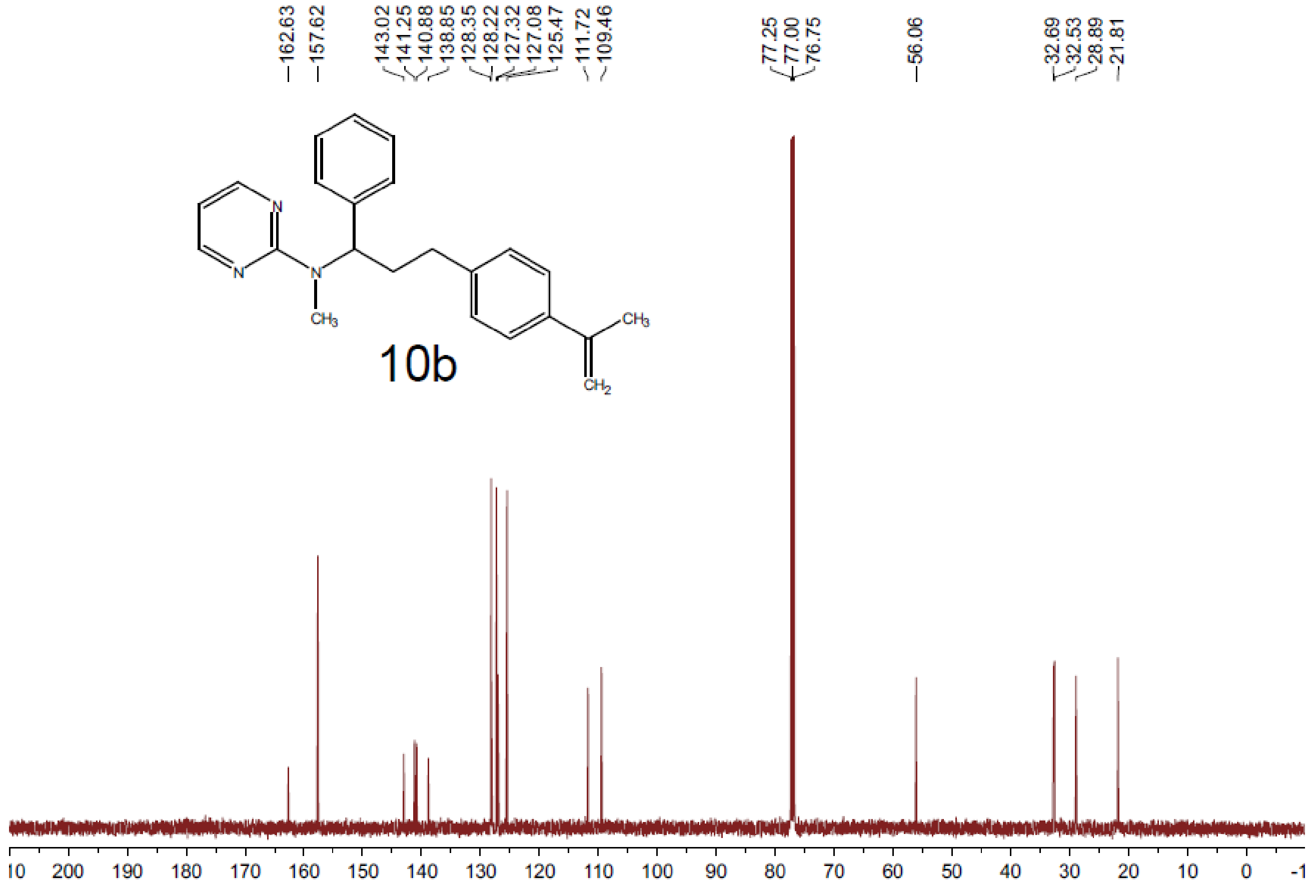
162.63
157.62
143.02
141.25
140.88
138.85
128.35
128.22
127.32
127.08
125.47
111.72
109.46
77.25
77.00
76.75
56.06
32.69
32.53
28.89
21.81
10b

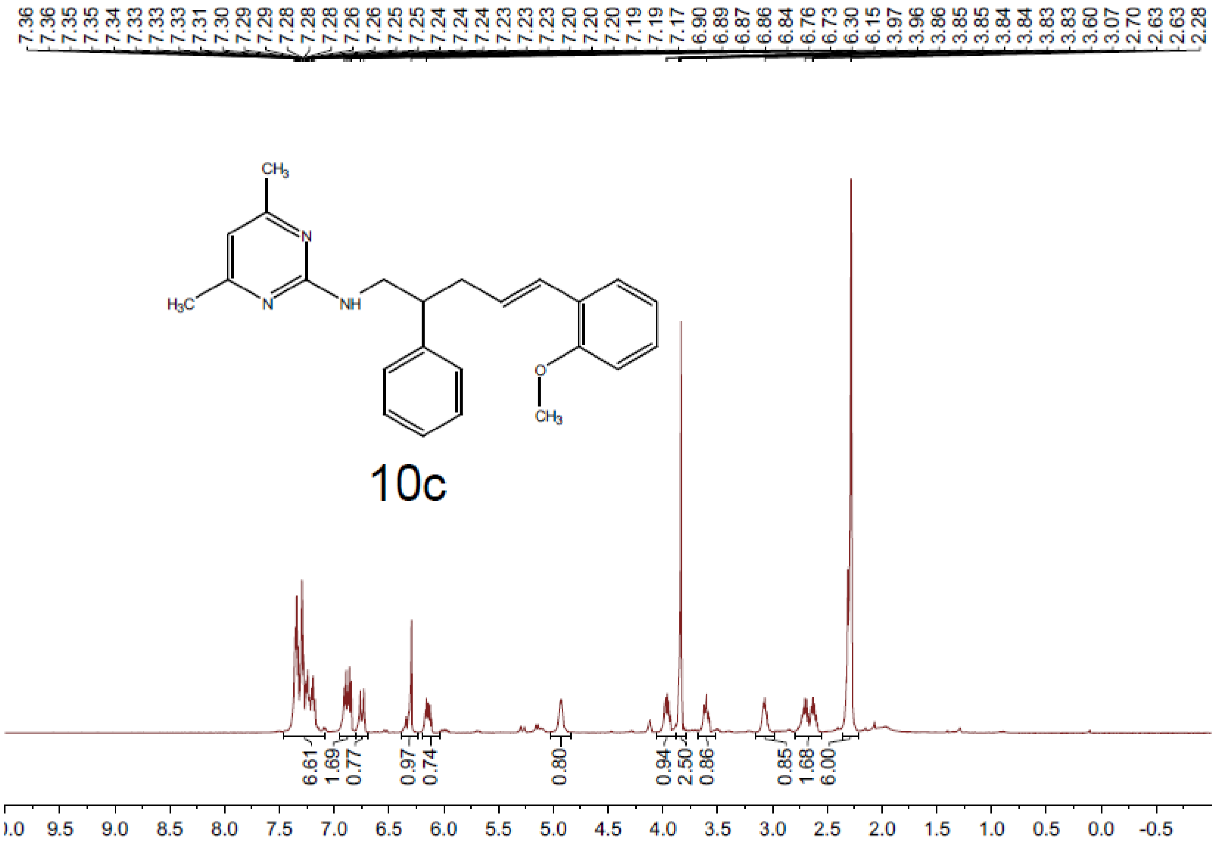
10c
6.61
1.69
0.77
0.97
0.74
0.80
0.94
2.50
0.86
0.85
1.68
6.00

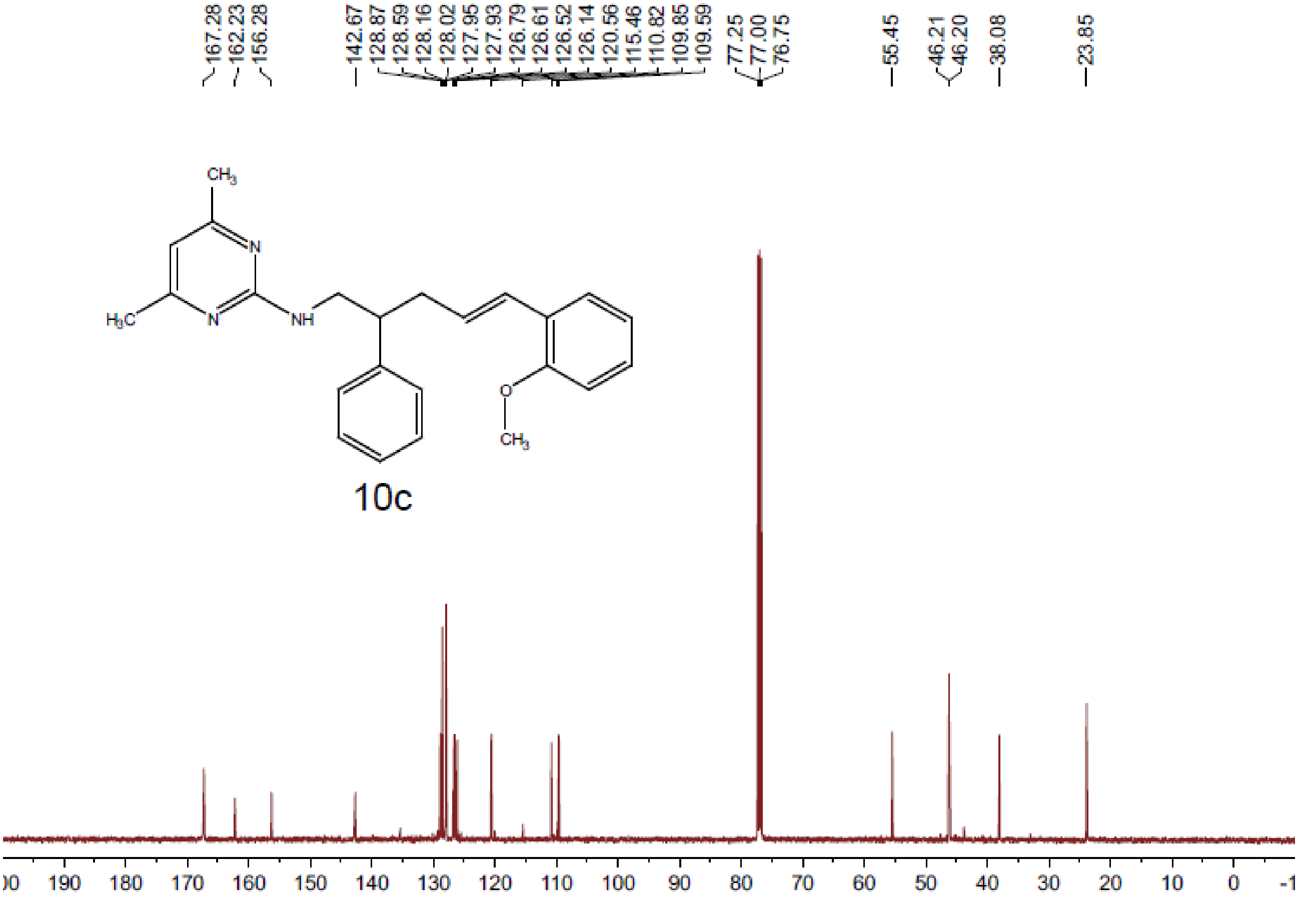

10c

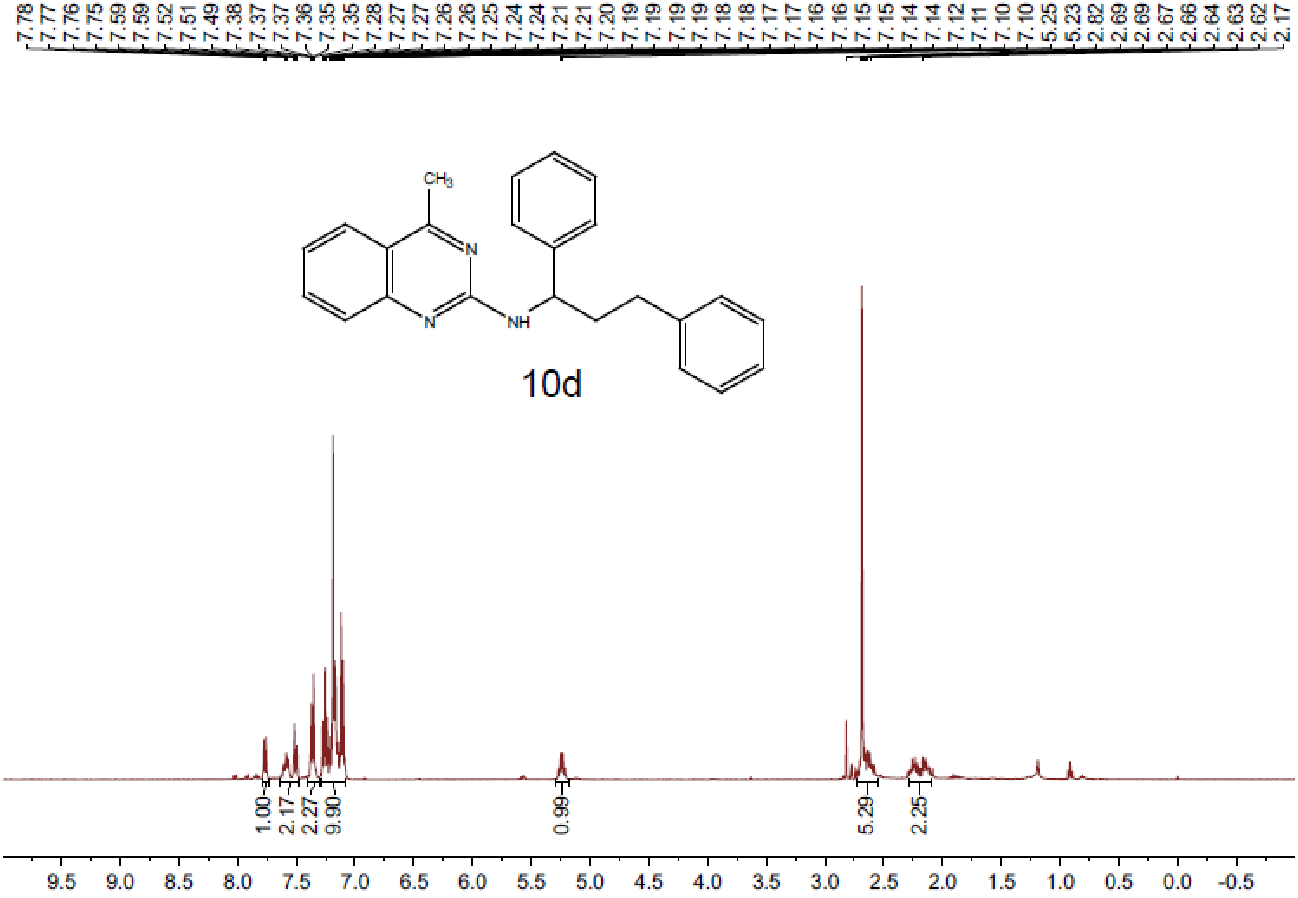

10d

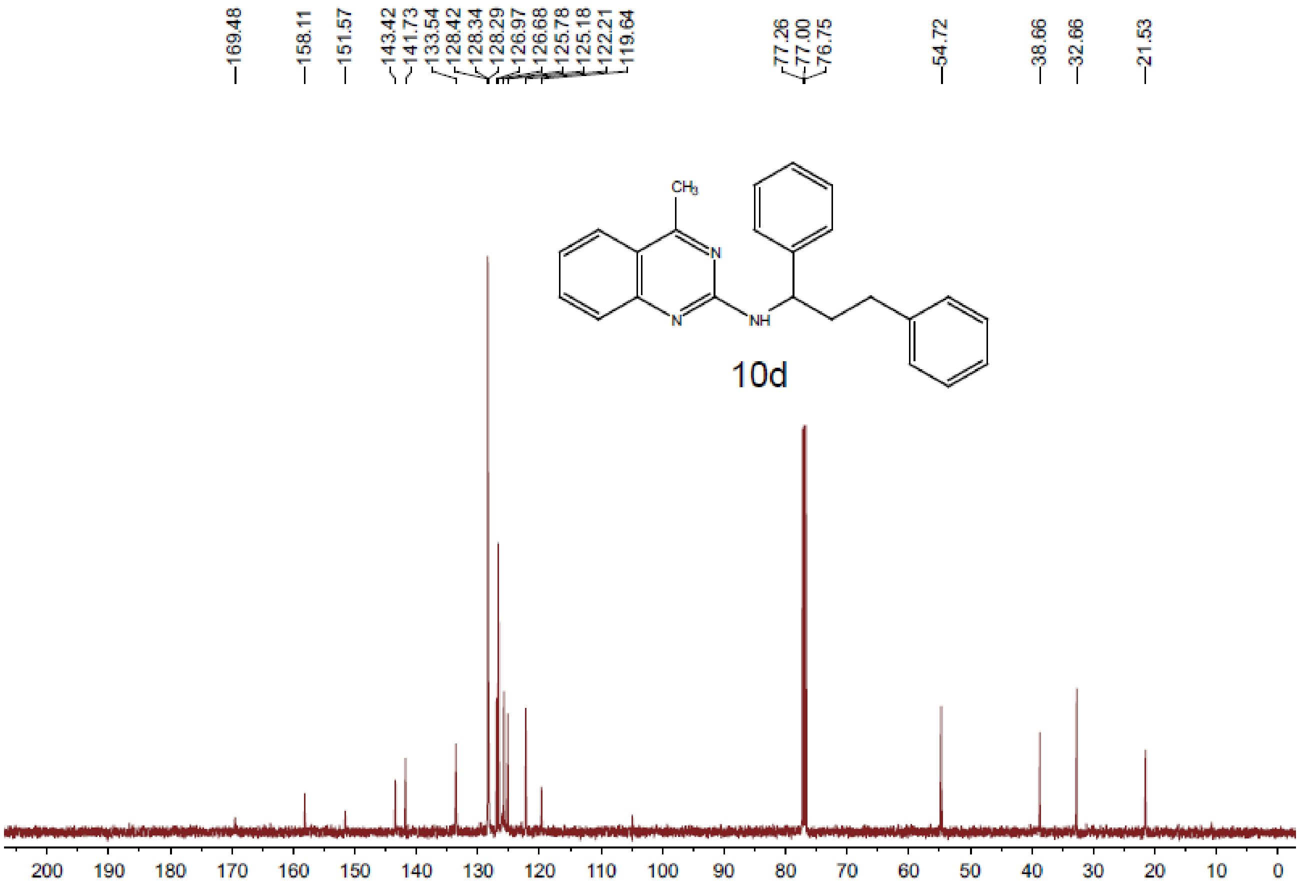
169.48
158.11
151.57
143.42
141.73
133.54
128.42
128.34
128.29
126.97
126.68
125.78
125.18
122.21
119.64
77.26
77.00
76.75
54.72
38.66
32.66
21.53
CH3
N
N
NH
10d
200
190
180
170
160
150
140
130
120
110
100
90
80
70
60
50
40
30
20
10
0

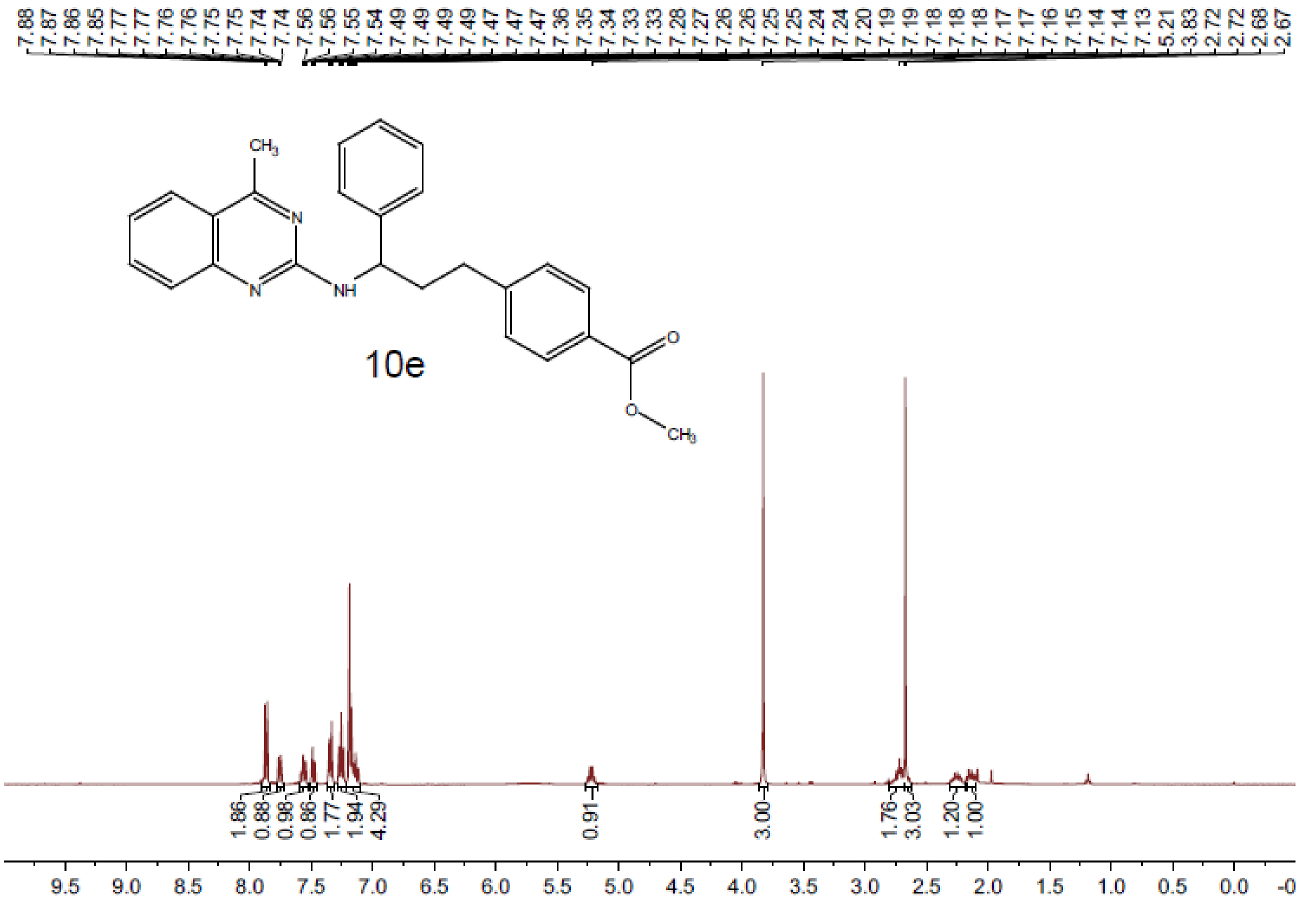
7.88 7.87 7.86 7.85 7.77 7.77 7.76 7.76 7.75 7.75 7.74 7.74 7.56 7.56 7.55 7.54 7.49 7.49 7.49 7.49 7.47 7.47 7.47 7.36 7.35 7.34 7.33 7.33 7.28 7.27 7.26 7.26 7.25 7.25 7.24 7.24 7.20 7.19 7.19 7.18 7.18 7.18 7.17 7.17 7.16 7.15 7.14 7.14 7.13 5.21 3.83 2.72 2.72 2.68 2.67
CH3
N
N
NH
O
O
CH3
10e
1.86
0.88
0.98
0.86
1.77
1.94
4.29
0.91
3.00
1.76
3.03
1.20
1.00
9.5
9.0
8.5
8.0
7.5
7.0
6.5
6.0
5.5
5.0
4.5
4.0
3.5
3.0
2.5
2.0
1.5
1.0
0.5
0.0
-0

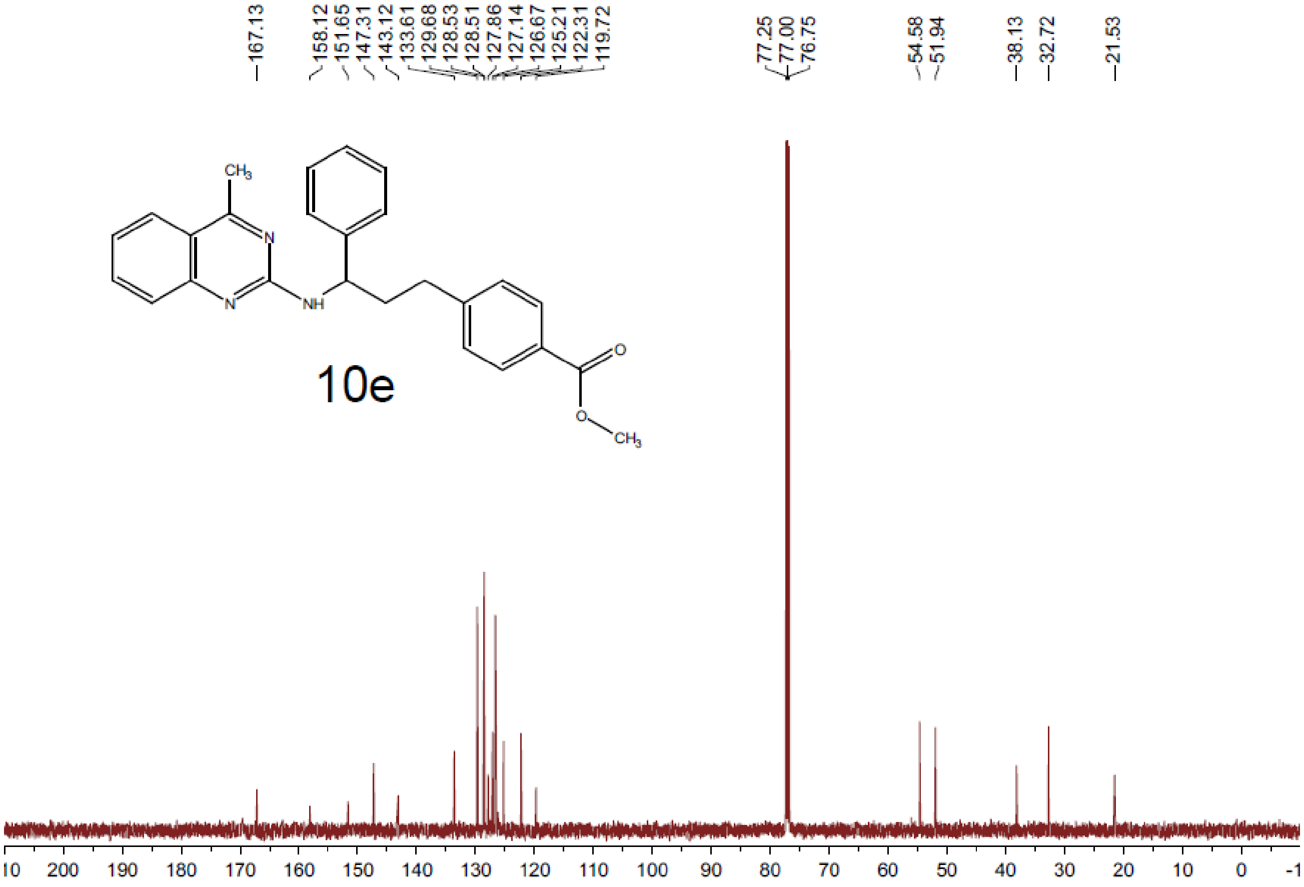

167.13
158.12
151.65
147.31
143.12
133.61
129.68
128.53
128.51
127.86
127.14
126.67
125.21
122.31
119.72
77.25
77.00
76.75
54.58
51.94
38.13
32.72
21.53
CH3
N
NH
O
10e
10
200
190
180
170
160
150
140
130
120
110
100
90
80
70
60
50
40
30
20
10
0
-1
